# Comprehensive functional annotation of susceptibility SNPs prioritized 10 genes for schizophrenia

**DOI:** 10.1038/s41398-019-0398-5

**Published:** 2019-01-31

**Authors:** Hui-Min Niu, Ping Yang, Huan-Huan Chen, Ruo-Han Hao, Shan-Shan Dong, Shi Yao, Xiao-Feng Chen, Han Yan, Yu-Jie Zhang, Yi-Xiao Chen, Feng Jiang, Tie-Lin Yang, Yan Guo

**Affiliations:** 1 Key Laboratory of Biomedical Information Engineering of Ministry of Education, School of Life Science and Technology, Xi’an Jiaotong University, 710049 Xi’an, China; 2 Department of Psychiatry, Hunan Brain Hospital, Changsha, Hunan Province China

## Abstract

Nearly 95% of susceptibility SNPs identified by genome-wide association studies (GWASs) are located in non-coding regions, which causes a lot of difficulty in deciphering their biological functions on disease pathogenesis. Here, we aimed to conduct a comprehensive functional annotation for all the schizophrenia susceptibility loci obtained from GWASs. Considering varieties of epigenomic regulatory elements, we annotated all 22,688 acquired susceptibility SNPs according to their genomic positions to obtain functional SNPs. The comprehensive annotation indicated that these functional SNPs are broadly involved in diverse biological processes. Histone modification enrichment showed that H3K27ac, H3K36me3, H3K4me1, and H3K4me3 were related to the development of schizophrenia. Transcription factors (TFs) prediction, methylation quantitative trait loci (meQTL) analyses, expression quantitative trait loci (eQTL) analyses, and proteomic quantitative trait loci analyses (pQTL) identified 447 target protein-coding genes. Subsequently, differential expression analyses between schizophrenia cases and controls, nervous system phenotypes from mouse models, and protein–protein interaction with known schizophrenia-related pathways and genes were carried out with our target genes. We finaly prioritized 10 target genes for schizophrenia (*CACNA1C, CLU, CSNK2B, GABBR1, GRIN2A, MAPK3, NOTCH4, SRR, TNF*, and *SYNGAP1*). Our results may serve as an encyclopedia of schizophrenia susceptibility SNPs and offer holistic guides for post-GWAS functional experiments.

## Introduction

Schizophrenia is one of the most mysterious and costliest mental disorders with a lifetime risk about 1% [1, 2]. Patients with a diagnosis of schizophrenia live 12–15 years shorter than normal people, and this mortality difference increases in recent decades^1^. Published twin studies found that the heritability of schizophrenia was 73–90% and environmental influence was estimated as 3–19%^2,3^. Basing on the high heritability of schizophrenia, genetic susceptibility factor decipherment would lead us to a better understanding of the genetic basis of schizophrenia.

To date, genome-wide association studies (GWASs) have identified many schizophrenia susceptibility loci. However, most of the disease-associated variants locate in intronic or intergenic regions^4^, which causes difficulties in clarifying their effects on diseases pathogenesis. Also, till now, the majority of functional SNPs remain unrevealed in schizophrenia studies.

Recent years, the increasing epigenomic datasets, including the Encyclopedia of DNA Elements (ENCODE)^5^ and Roadmap Epigenomics Project^6^, make it possible to understand the function of non-coding variants from epigenomic level. It has been reported that the non-coding regions could indirectly participate in the regulation of proximal or distal genes expression by functioning as regulatory elements. Typically, these include the DNA methylation sites, histone modification sites, DNase I hypersensitive sites, transcription factor (TF)-binding sites, as well as the proved enhancer and promoter regions, which are related to the cleavage, transcription, and translation of genes^4,7^. Given these, some functional annotations of GWASs reported SNPs, and the epigenomic contribution have identified a bunch of functional SNPs and extended our understanding in genetic regulation mechanisms^8–10^. For schizophrenia, a comprehensive annotation of GWASs results is needed.

In this study, we obtained schizophrenia-associated SNPs from GWASs and aimed to perform comprehensive functional annotation for all susceptibility loci. At first, we acquired known schizophrenia susceptibility SNPs (referred to as index SNPs) and SNPs in high linkage disequilibrium (LD) with the index SNPs. We also acquired schizophrenia susceptibility SNPs from GWAS summary studies. For SNPs in coding DNA sequences (CDS) regions, we predicted the potential effect of missense SNPs on protein functions. For SNPs in untranslated regions (UTRs), we predicted their potential effects on microRNA binding. For SNPs located in promoters, we predicted whether they have effects on TF binding. For SNPs located in intronic or intergenic region, we detected whether they might regulate enhancer activity through affecting TF-binding ability. For all annotated functional SNPs, histone modification enrichment, methylation quantitative trait loci (meQTL), expression quantitative trait loci (eQTL), and proteomic quantitative trait loci (pQTL) analyses were subsequently performed. In addition, we used multiple ways to validate the correlation of target genes with schizophrenia. Our results may serve as an encyclopedia of schizophrenia susceptibility SNPs and offer guides for post-GWAS functional experiments.

## Materials and methods

### Acquisition of schizophrenia-associated SNPs

**Fig. 1:**
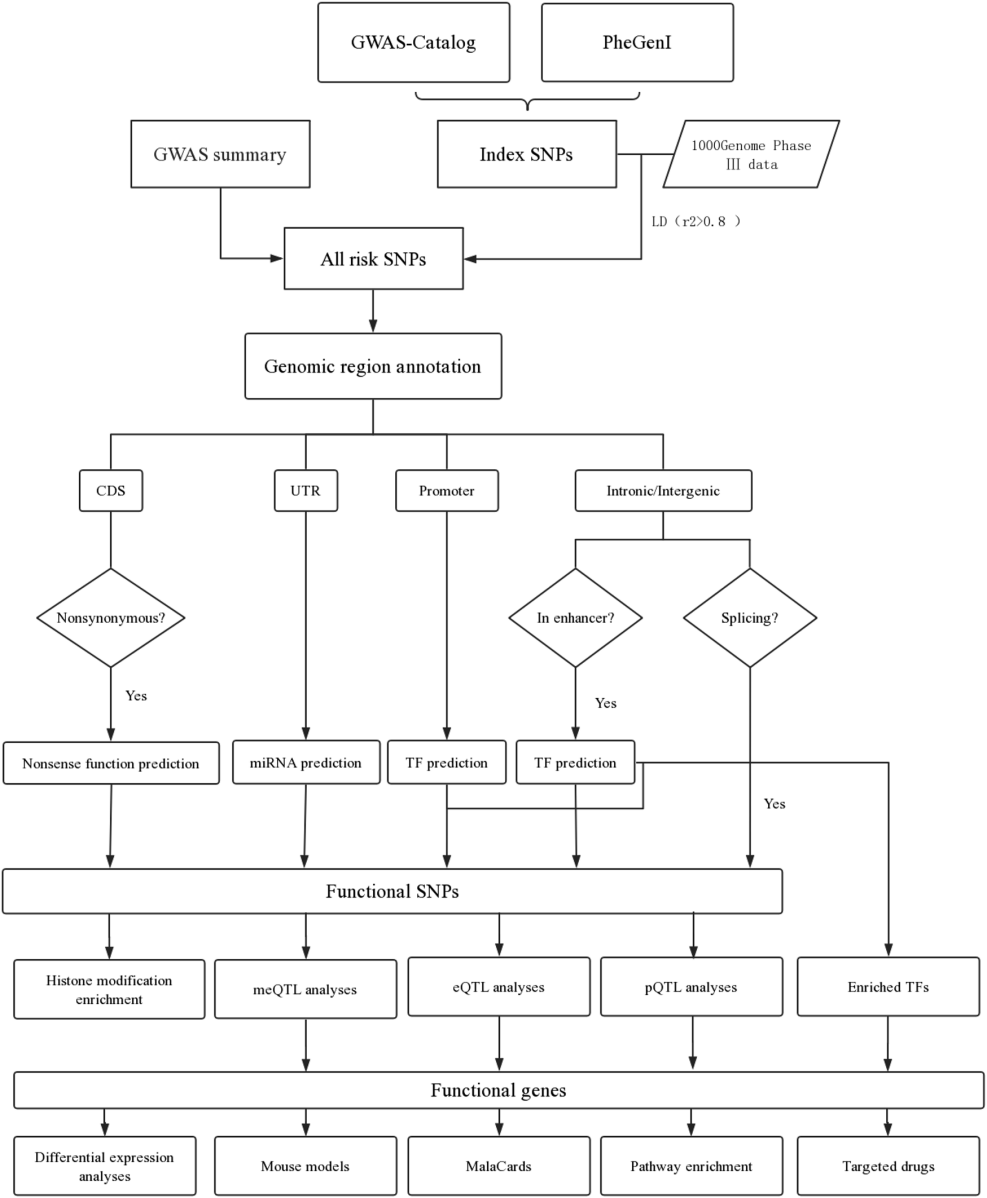
The flow diagram of the analysis strategy. Schizophrenia-associated index SNPs were obtained from public GWASs databases. LD analyses were carried out using the 1000 genome data and GWASs summary data were used to obtain all schizophrenia susceptibility SNPs. Functional annotation was carried out according to the genomic regions of all susceptibility SNPs. For annotated functional SNPs, histone modification enrichment, meQTL, eQTL, and pQTL analyses then were carried out. Various methods were used to validate target genes

Figure 1 shows the analysis strategy of this study. SNPs associated with schizophrenia were obtained from two common resources of SNP–trait associations: GWAS-catalog^11^ and phenotype–genotype integrator (http://www.ncbi.nlm.nih.gov/gap/phegeni). SNPs reported to be associated with schizophrenia with *P* < 5 × 10^−8^ were selected. These were further referred to as index SNPs. Due to the low genomic coverage of microarrays, those true causal variants may not be detected^12^. Therefore, we also obtained the SNPs in strong LD (*r*^2^ ≥ 0.8) with the index SNPs using the 1000 Genomes Phase III data. These were further referred to as LD SNPs. In addition, we also acquired schizophrenia susceptibility SNPs from GWAS summary studies^13,14^ with *P* < 5 × 10^−8^, which were called summary SNPs hereafter. All of the index, LD and summary SNPs were considered as schizophrenia susceptibility SNPs and were subjected to subsequently analyses. Using GencodeV19 reference genome (http://www.gencodegenes.org/releases/), the susceptibility SNPs were annotated with ANNOVAR^15^ to get their genomic region information.

### Functional annotation of schizophrenia-associated SNPs

For SNPs in CDS, we focused on nonsynonymous SNPs. We predicted the potential effect of missense SNPs on protein functions using PROVEAN^16^, SIFT^17^, Polyphen2 (ref. ^18^), and CADD^19^. For SNPs located in 3′-UTR, we predicted the miRNA-binding affinity with the UTR sequences using MirSNP database^20^.

For SNPs located within 1 kb region upstream of transcription start site (TSS), we considered that these SNPs are located in promoters. For SNPs located in intronic or intergenic regions, we used the chromatin 15-states data in brain and blood tissues (Supplementary Table S1) from the Roadmap project (http://www.roadmapepigenomics.org/) to check whether they are located in enhancers). Subsequently, we detected whether the promoter and enhancer SNPs would affect TF binding using the SNP2TFBS database^21^. We also investigated which TFs they were enriched for disruption. The GEO dataset GSE69838 (ref. ^22^) was used to check whether these enriched TFs were differentially expressed during neuronal differentiation of SH-SY5Y cell (0 day vs 11 day).

### Histone modification enrichment

We used histone modification data (including 11 histone marks H3K27ac, H3K27me3, H3K36me3, H3K4me1, H3K4me2, H3K4me3, H3K79me2, H3K9ac, H3K9me, H3K9me3, and H4K20me1) in brain and blood tissues from the Roadmap project (supplementary Table S1) to predict these schizophrenia functional SNPs were enriched in what kind of histone marks using Variant Set Enrichment (VSE)^23^. In the VSE method, a disjoint list of associated variant set (AVS) had be constructed at the first step, in which only one SNP is present in one LD block to avoid inflating test statistics.

### meQTL analyses

We used the meQTL data in the prefrontal cortex from 335 controls and 191 schizophrenia patients reported by Jaffe et al.^24^ to investigate whether the annotated functional SNPs could affect DNA methylation levels.

### eQTL analyses

eQTL results derived from (1) the dorsolateral prefrontal cortex of schizophrenia patients (*N* = 258) and control subjects (*N* = 279) from CommonMind Consortium (CMC)^25^ and (2) whole blood from 3841 samples reported by Bonder et al.^26^ were used to investigate whether the annotated functional SNPs could affect gene expression levels in schizophrenia-related tissues. For functional enhancer SNPs, genes with TSS flanking ±1 Mb around the SNPs were used for investigating eQTL signals in all related tissues. For other functional SNPs, their located genes were used. The Benjamini–Hochberg procedure was executed to account for the multiple testing problems. We also applied independent integrative analysis (Summary-data-based Mendelian Randomization, SMR) approach developed by Zhu et al.^27^ to the GWASs (summary-level data from GWASs summary studies previously mentioned^13,14^) and eQTL data (summary-level data from the Genotype-Tissue Expression (GTEx) v7 project^28^, after integrating 13 brain subregions, and whole blood). After applying a conservative threshold (*P*_HEIDI_ > 0.05 and *P*_eQTL_ < 0.05), genes with FDR *q* < 0.05 in both eQTL and SMR results in brain or blood separately were obtained.

### pQTL analyses

We used the data from blood plasma proteome reported by Suhre et al.^29^ to investigate whether the annotated functional SNPs in blood could affect protein levels. The expression levels of 1124 proteins were quantified in 1000 individuals of the population-based KORA study. For functional enhancer SNPs, all proteins associated with susceptibility SNPs were used. For other functional SNPs, proteins coded by their located genes were used. The Benjamini–Hochberg procedure was executed, and the threshold of FDR *q* < 0.05 was used to filter out non-significant signals.

### Differential expression analyses

For all target genes (obtained from meQTL, eQTL, and pQTL analyses, and enriched TFs) which encode proteins (GencodeV19 annotation data), we further checked whether they were differentially expressed between schizophrenia case and control samples. Meta-analysis using sample-size weighted model in the METAL software^30^ was carried out to combine the results of differential expression analyses from GSE53987 (prefrontal cortex, striatum, and hippocampus from 48 schizophrenia patients and 55 control subjects)^31^, GSE93987 (dorsolateral prefrontal cortex from 102 patients and 106 controls)^32^, and GSE38484 (whole blood from 106 patients and 96 controls)^33^). Effect direction was taken into account in this model. The Benjamini–Hochberg procedure was executed, and the threshold was same as mentioned before.

### Associated genes from mouse models

We used phenotype data in mouse models from two projects, Mouse Genome Database (MGD)^34^ and International Mouse Phenotyping Consortium (IMPC)^35^, to verify whether these target genes deficiency could lead to disorders in nervous system.

### Protein–protein interaction

We also compare our target genes with known schizophrenia-related pathways and genes information reported by MalaCards database (http://www.malacards.org/). For those genes not included, we checked whether they could be related to known schizophrenia pathways or genes through gene–gene interaction using data from MyProteinNet database (http://netbio.bgu.ac.il/myproteinnet/).

### Pathway enrichment

For target genes which had at least two hits in differential expression analyses, mouse models and Malacards, we conducted KEGG pathway enrichment analyses with these genes to detect their related pathways (http://amp.pharm.mssm.edu/Enrichr/).

### Targeted drugs

We used data of targeted drugs from DrugBank (https://www.drugbank.ca/) to investigate whether there were any connection between target genes and approved biotech drugs.

## Results

### Acquisition of schizophrenia-associated SNPs

**Fig. 2:**
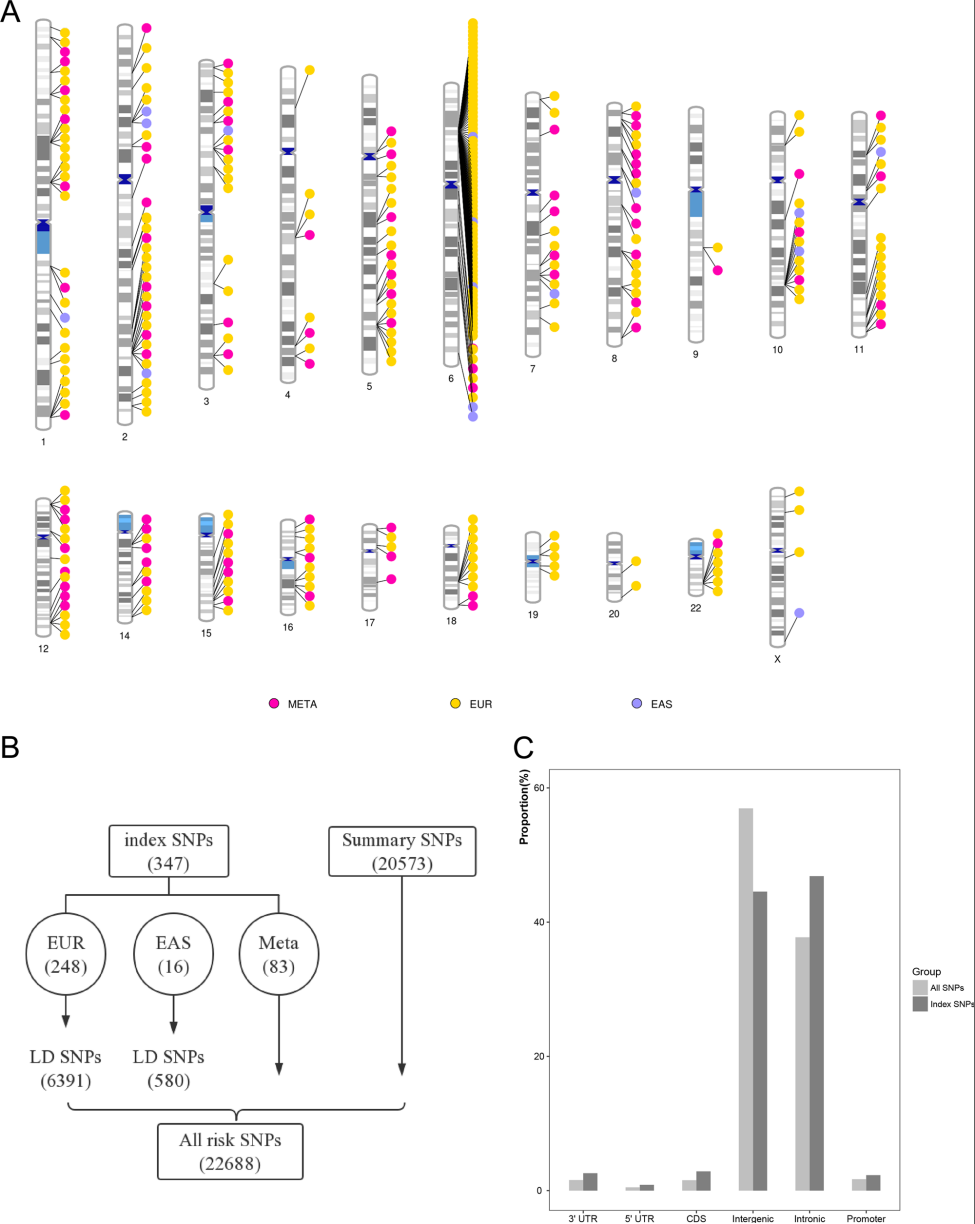
The obtainment and genomic region of schizophrenia susceptibility SNPs. **a** The distribution of the 347 schizophrenia index SNPs in the genome. **b** Schematic of all risk SNPs compilation from available GWASs. EUR European, EAS Asian. **c** Genomic region annotation distribution of the index and all risk SNPs

There were 347 schizophrenia-associated SNPs obtained from GWAS-Catalog and PheGenI in total (Fig. 2a, Supplementary Table S2). Among the index SNPs, 248 SNPs were from European-specific GWAS sample. Subsequently, we acquired 6926 SNPs in high LD with the index SNPs. We also obtained 20,573 susceptibility SNPs from GWAS summary studies (Fig. 2b). All susceptibility SNPs (22,688) were annotated with ANNOVAR and their classifications are shown in Fig. 2c.

### Functional annotation of associated SNPs

We identified 338 susceptibility SNPs located in CDS. Of these SNPs, 161 encoded synonymous substitutions, 172 encoded missense substitutions, 1 frameshift substitution, and 4 still unknown. We explored the potential effects of the missense SNPs using PROVEAN, SIFT, Polyphen2, and CADD. Sixty-six SNPs were predicted to be damaging by at least one algorithm (Supplementary Table S3), including five index SNPs, rs1051061, rs13107325, rs16897515, rs2247870, and rs950169. Seven SNPs were predicted to be damaging by all four algorithms, including rs9257834, rs678, rs4584886, rs34788973, rs13195402, rs13195401, and rs1029871. Moreover, rs13195401, rs13195402, and rs34788973 were all in high LD with index SNPs rs13194053, rs55834529, and rs6932590, which may cause damaging changes in *BTN2A1* and *OR2B2*.

We identified 357 SNPs in 3′-UTR, and 119 SNPs were predicted to be involved in microRNA targets (Supplementary Table S4), including four index SNPs (rs10786736, rs13205911, rs3735025, and rs4702). In addition, another two SNPs, rs1376607 and rs3735026, were in complete LD with the index SNP rs3735025.

**Fig. 3:**
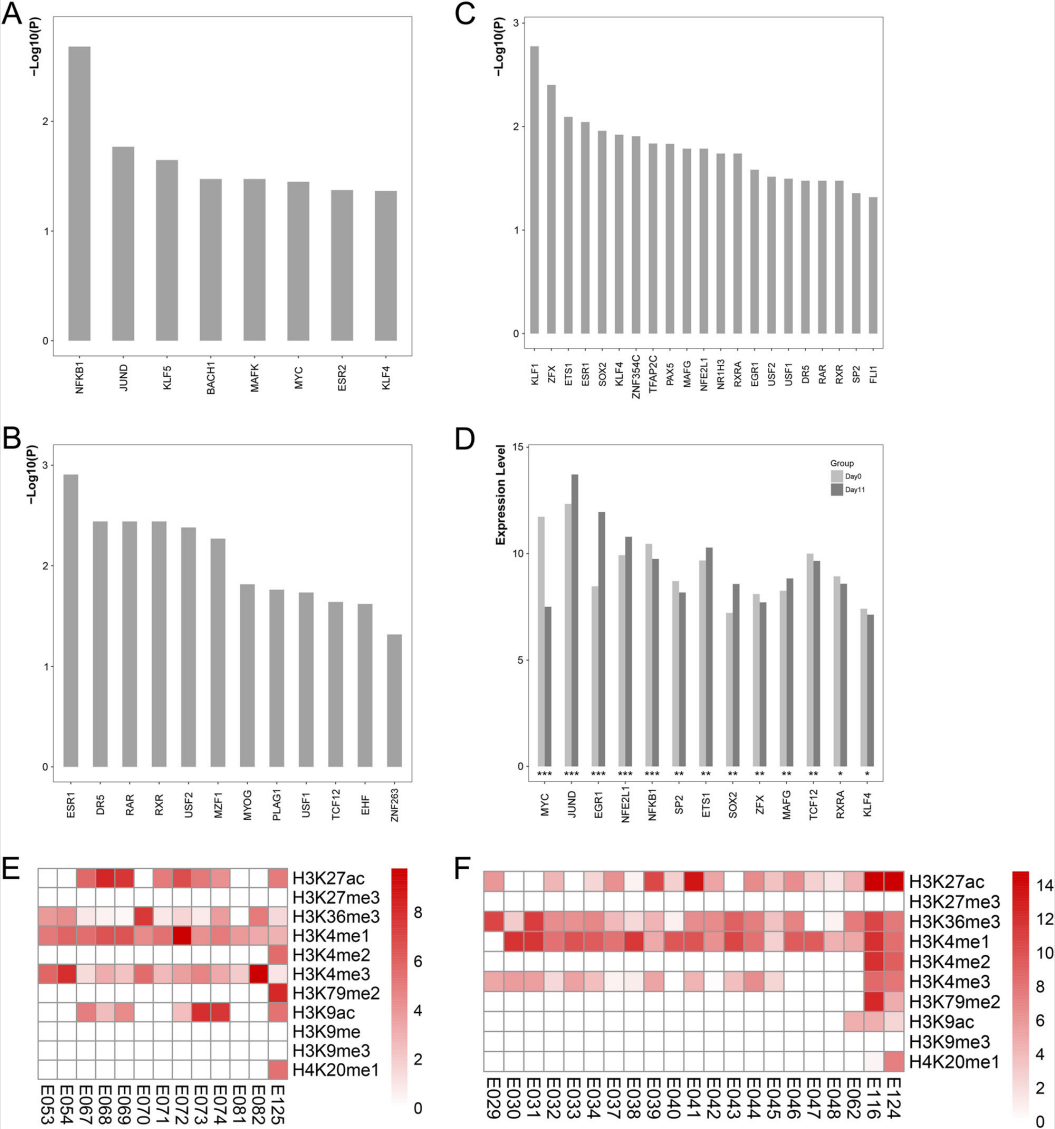
The enrichment of TFs and histone markers. **a** Transcription factor enrichment results for the promoter SNPs. **b** TF enrichment results for the enhancer SNPs in brain tissues. **c** TF enrichment results for the enhancer SNPs in blood tissues. **d** Differential expression analyses results (0 day vs 11 day) of the promoter and enhancer SNPs enriched TFs in human SH-SY5Y cells. *adjust *P* < 0.05, **adjust *P* < 0.01, ***adjust *P* < 0.001. **e** The results of histone modification enrichment for all functional SNPs in brain cell lines. **f** The results of histone modification enrichment for all functional SNPs in blood cell lines. The shallow of red represents the value of −log10 (adjust *P*)

Of all 388 promoter SNPs, 89 SNPs might disrupt TF binding, including index SNP rs796364. For example, the substitution from C to A of rs796364 would affect the binding affinity of NFIC in the upstream of *FTCDNL1* gene. For SNPs in intronic or intergenic region, we identified 2820 and 4694 SNPs located in the enhancer regions of brain and blood cells, respectively. Among them, 659/1028 SNPs in brain/blood might regulate enhancer activity through affecting TF binding. These promoter SNPs were enriched for disruption of eight TFs binding (Fig. 3a) and enhancer SNPs were found to potentially disrupt 12/21 TFs binding in brain/blood (Fig. 3b, c), 34 in total (supplementary Table S5). Comparisons of the gene expression levels during SH-SY5Y cell differentiation detected significant expression changes for 13 TFs (MYC, MAFG, JUND, NFKB1, EGR1, ETS1, KLF4, NFE2L1, RXRA, SOX2, SP2, TCF12, and ZFX; Fig. 3d), suggesting these TFs may have significant functions on nerve cells differentiation or proliferation (Supplementary Table S5).

Finally, we identified 934 functional SNPs in brain and 1300 (rs134885, rs1800629, and rs2747054 were identified in both enhancer and promoter regions and were only counted once) in blood, including 1 splicing SNP, 66 missense SNPs which may damage protein functions, 119 UTR SNPs involved in potential microRNA targets, 89 promoter, and 659/1028 enhancer SNPs which might disrupt TF binding in brain/blood.

### Histone modification enrichment

Histone modification enrichment showed that these functional SNPs were enriched in H3K27ac (active enhancer), H3K36me3 (active elongation), H3K4me1 (active enhancer), and H3K4me3 (active promoter) in most of the brain (Fig. 3e) and blood cell lines (Fig. 3f). All of these histone marks would activate gene expression.

### QTL analyses

According to meQTL results, 516 of 934 annotated functional SNPs in brain were associated with DNA methylation alterations of 224 genes (Supplementary Table S6). For all annotated functional SNPs, we performed SMR and eQTL analyses to find their target genes. One thousand six hundred and one genes in brain tissues and 552 genes in blood tissue were identified in SMR analyses. One hundred and seventy-five genes in CMC and 568 genes in data from Bonder et al. were identified in eQTL analyses (Supplementary Table S6). As a result, 299 genes in blood and 147 genes in brain were overlapped in SMR and eQTL analyses, respectively, 380 genes in total. We also carried out pQTL analyses in blood tissue. Due to the low genotyping density of the current available proteomic data, only 41 functional SNPs were identified and were associated with 19 proteins (supplementary Table S6). Besides, C2, C4A, C4B, MICA, and MICB were identified as the targets of the functional SNPs from both gene and protein expression levels.

### Validation of target genes

**Fig. 4:**
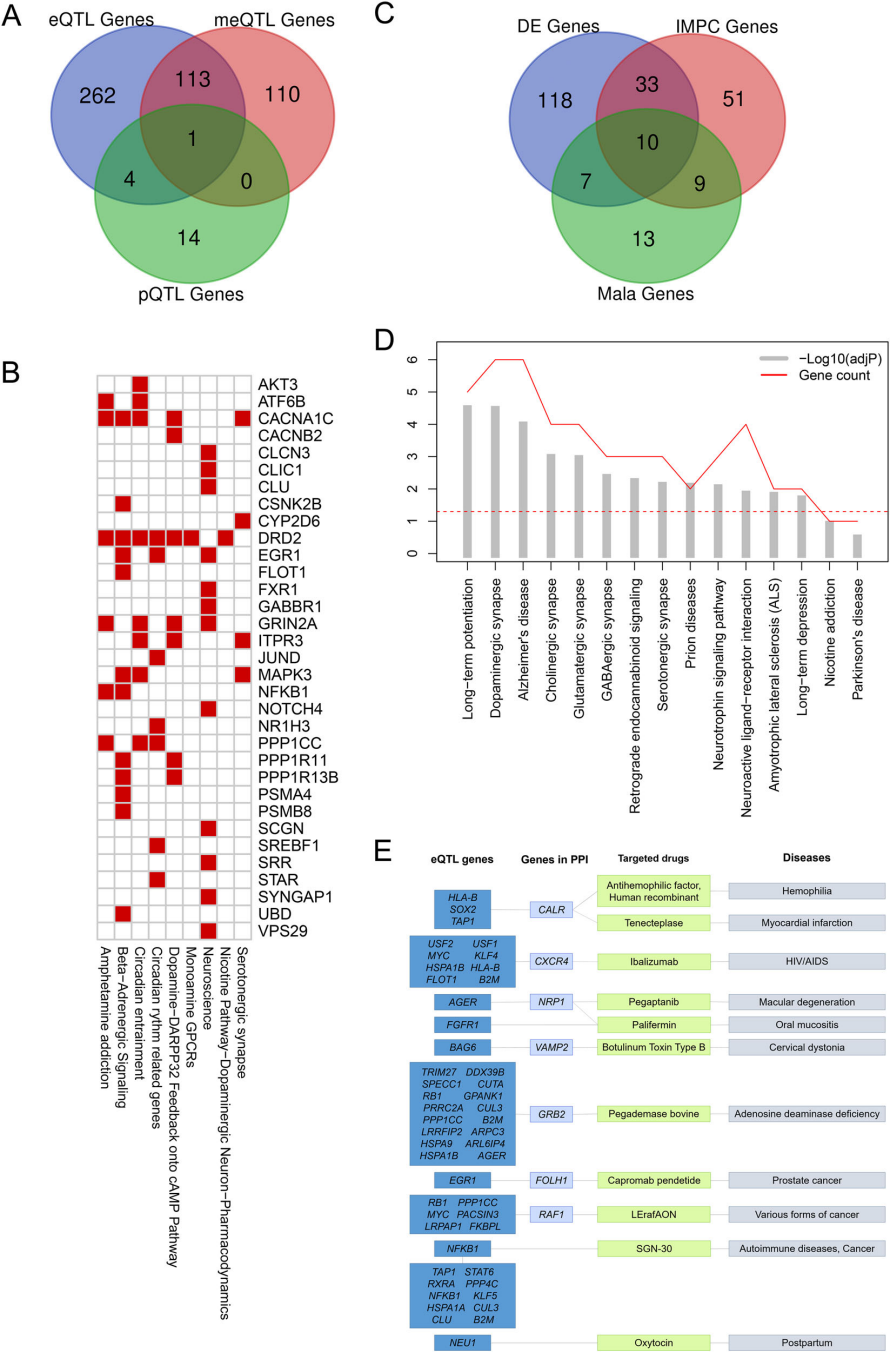
QTLs target genes. **a** The Venn diagram of the target genes from QTLs analyses. **b** The target protein-coding genes (ordinates) assigned to known schizophrenia-related pathways (abscissa). **c** The Venn diagram of the validation of target genes. **d** The enriched schizophrenia-related pathways of 59 target genes which had at least two hits in validations. **e** Connections between target genes (dark blue), genes from PPI (blue), and drugs (green) indicated for other diseases (purple)

Finally, we acquired 538 target genes (Fig. 4a, including 34 enriched TFs, 224 meQTL, 380 eQTL, and 19 pQTL target genes), including 447 protein-coding genes. For these protein-coding genes, 168 genes (37.58%) were differentially expressed between schizophrenia cases and controls (Supplementary Table S7). We also confirmed our results in mouse models and 103 (23.04%) genes were related to nervous system phenotype (Supplementary Table S8). Using the schizophrenia-related pathways data from MalaCards, we found 33 protein-coding genes could be assigned to several signal pathways of schizophrenia (Fig. 4b). Thirteen genes (*C4A, C4B, CACNA1C, CYP2D6, DRD2, GABBR1, GRIN2A, ITIH3, KCTD13, MICB, NOTCH4*, *SRR*, and *TNF*) had been reported related to schizophrenia. Among them, *CACNA1C, CYP2D6, DRD2, GABBR1, GRIN2A, NOTCH4*, and *SRR* were also assigned to related pathways. However, there were still some target genes could not be explained. Using gene–gene interaction data, we found that 221 of the 408 unexplained genes (54.17%) could be related to known schizophrenia pathways through gene–gene interaction (Supplementary Figure S1A) and 65 genes could be related to known schizophrenia-related genes (including 18 genes related to 6 schizophrenia elite genes, Supplementary Figure S1B). Subsequently, we conducted KEGG pathway enrichment analysis for 59 target genes which had at least two hits in above methods (Fig. 4c), and they were enriched in 13 related signal pathways (Fig. 4d). In addition, there were 12 approved drugs targeted to functional genes or schizophrenia-related genes which interacted with functional genes (Fig. 4e).

Notably, we prioritized 10 target genes, *CACNA1C, CLU, CSNK2B, GABBR1, GRIN2A, MAPK3, NOTCH4, SRR, TNF*, and *SYNGAP1*, which were differentially expressed between schizophrenia cases and controls, validated in mouse models and MalaCards directly (Fig. 4c, Table 1, Supplementary Table S9). The correlation with schizophrenia of six among them were already identified. *CLU* and *MAPK3* were only identified as new schizophrenia susceptibility loci by GWASs^13^, but the other two genes (*CSNK2B* and *SYNGAP1*) were not identified yet.

**Table 1:** Analyses results of 10 prior target genes for schizophrenia

Gene	FDR_(DE)_	Pathway	Phenotype in mouse models	QTLs
*CACNA1C*	2.78 × 10^−2^	Amphetamine addiction; beta-adrenergic signaling; circadian entrainment; dopamine-DARPP32 feedback onto cAMP pathway; serotonergic synapse	Abnormal nervous system physiology; nervous; nervous system phenotype	meQTL
*CLU*	2.35 × 10^−3^	Neuroscience	Nervous; nervous system phenotype	meQTL
*CSNK2B*	3.53 × 10^−2^	Beta-adrenergic signaling	Nervous; nervous system phenotype	eQTL, meQTL
*GABBR1*	2.35 × 10^−2^	Neuroscience	Nervous; nervous system phenotype; abnormal somatic nervous system morphology; abnormal sciatic nerve morphology; abnormal nervous system electrophysiology	eQTL, meQTL
*GRIN2A*	3.97 × 10^−4^	Amphetamine addiction; circadian entrainment; dopamine-DARPP32 feedback onto cAMP pathway; neuroscience	Nervous; nervous system phenotype	meQTL
*MAPK3*	4.75 × 10^−2^	Beta-adrenergic signaling; circadian entrainment; serotonergic synapse	Nervous; nervous system phenotype	eQTL
*NOTCH4*	4.63 × 10^−2^	Neuroscience	Nervous; nervous system phenotype	eQTL, meQTL
*SRR*	4.75 × 10^−2^	Neuroscience	Abnormal nervous system physiology; nervous; nervous system phenotype	eQTL, meQTL
*SYNGAP1*	2.78 × 10^−2^	Neuroscience	Nervous; nervous system phenotype	meQTL
*TNF*	2.03 × 10^−2^	—	Abnormal nervous system physiology; nervous; nervous system phenotype	eQTL

Note: DE means differential expression

## Discussion

In this study, we obtained SNPs associated with schizophrenia from the current results of GWASs and conducted functional annotation comprehensively for all susceptibility loci. Different from previous studies, not only the SNPs in exon, UTR, and promoter regions, we also focused on the annotation in intronic and intergenic regions using epigenomic data.

We found 538 target genes, including 447 protein-coding genes. One hundred and sixty-eight (37.58%) of them were differentially expressed between schizophrenia cases and controls, and 103 of them were confirmed related to nervous system phenotype in mouse models, both indicating their important involvement in the development of schizophrenia. Only 33 protein-coding genes could be directly assigned to schizophrenia-related pathways, and 13 were confirmed as schizophrenia-related genes. Furthermore, 54.67% of the rest target protein-coding genes could be related to known schizophrenia-related genes or pathways through gene–gene interaction, which confirmed gene regulatory networks are sufficient to interconnect these genes with disease-related genes^36^.

C2, C4A, C4B, MICA, and MICB were identified as the targets of the functional SNPs from both gene and protein expression levels. *MICB* was found differentially expressed between schizophrenia cases and controls. *C4A*^37^, *C4B*^37^, and *MICB*^38,39^ were previously reported to be associated with schizophrenia. Hakobyan et al.^40^ identified that the hemolytic activity of the C2 complement components was significantly lower in the serum of the schizophrenic patients.

We prioritized 10 target genes for schizophrenia. They were all differentially expressed between schizophrenia cases and controls, validated in mouse models and MalaCards directly. The correlation with schizophrenia of six among them were already identified. *CACNA1C*^41,42^, *GRIN2A*^43^ (interacted with elite gene *AKT1*), *TNF*^44^, and aberrant *SRR*^45^ may contribute to schizophrenia pathogenesis. *GABBR1*^46^ and *NOTCH4*^47^ may confer susceptibility to the development of schizophrenia. Interaction between *COMT* (elite gene) and *NOTCH4* genotypes may predict the treatment response to typical neuroleptics in schizophrenia patients^48^. *CLU* and *MAPK3* were indentified as new loci in GWAS study^13^.

Besides, the other two genes (*SYNGAP1* and *CSNK2B)* were most likely to be schizophrenia susceptibility genes. *SYNGAP1* was assigned to Neuroscience pathway directly, and *CSNK2B* in beta-adrenergic signaling pathway. However, *SYNGAP1* and *CSNK2B* are located in the extended MHC complex on chromosome 6, so it is doubtful those are truly novel associations with schizophrenia. We performed Pearson correlation analyses between *CSNK2B* (or *SYNGAP1*) and *C4A, C4B*^37^ or the other gene *SYNGAP1* (or *CSNK2B*), and did conditional eQTL analyses between each eQTL top SNPs and corresponding target genes (*CSNK2B* and *SYNGAP1*) by adjusting the residual effect of the *C4A* and *C4B* using genotype and expression data (after covariates) of 13 brain subregions and whole blood from the GTEx-v7 project. (1) Pearson correlation analyses results showed that there was no significant correlations between *CSNK2B* and *C4A* or *C4B* in 13/14 or 11/14 tissues, respectively, and there was no significant correlations between *SYNGAP1* and *C4A* or *C4B* in 11/14 or all 14 tissues, respectively. Moreover, we explored the correlations between *CSNK2B* and *SYNGAP1* and found that there was no significant correlations for *CSNK2B* and *SYNGAP1* in 12/14 tissues (supplementary Table S10). (2) In the conditional eQTL analyses, we used the top SNPs obtained in the SMR analyses. As a result, 10 SNPs were used for *CSNK2B*, and 3 SNPs for *SYNGAP1*. We checked if the eQTL association remained significant after adjusting the residual effect of *C4A* and *C4B* (Supplementary Table S10). For *CSNK2B*, conditional eQTL association remained significant (*P* < 0.05) in 21 SNP-tissues pairs (8 SNPs and 10 tissues, respectively, and there were 30 pairs before adjusting the C4 signal). *SYNGAP1* remained significant in 2/3 SNP-tissues pairs. Thus, the observed eQTL associations between corresponding SNPs and *CSNK2B, SYNGAP1* were independent of C4 signal. These analyses results suggested us that *CSNK2B* and *SYNGAP1* were independent from the C4 signal and each other.

There were 12 approved drugs from biotech targeted to functional genes, while Pegademase bovine is used for the treatment of adenosine deaminase, which is involved in the pathophysiology of schizophrenia^49^. Botulinum Toxin Type B is used for dystonia, which is one of the symptoms of schizophrenia. The other 10 drugs were all non-psychiatric medications. We searched for the potential clinical relevance of those non-psychiatric medications in context of schizophrenia. (1) There are four drugs (Tenecteplase, Antihemophilic factor, human recombinant, and Oxytocin) approved for use in blood disorders. Patients with schizophrenia have excess cardiovascular morbidity and mortality^50^. There was high prevalence of psychopathology in children with blood disorders^51^. (2) Four drugs (Ibalizumab, Pegaptanib, Palifermin, and SGN-30) are applied to autoimmune diseases or inflammation. It was found that autoimmune diseases were associated with an increased risk of developing schizophrenia^52^. An important role of peripheral immune-to-brain communication pathways was suggested in schizophrenia, and there was an association between elevated levels of circulating inflammatory cytokines and subsequent risk of psychosis^53^. (3) Three3 medications (SGN-30, LErafAON, and Capromab pendetide) are used for various forms of cancers. Patients with schizophrenia have been found with a reduced risk of cancer^54^. It has been found that patients with schizophrenia have a hyper-opaminergic system and dopamine has the ability to inhibit tumor angiogenesis^55^. Consequently, these non-psychiatric medications may have chance to be applied to schizophrenia, as well to a better understanding of the schizophrenia pathogenesis.

There are still some limitations of this study. Because of the limitation of current available pQTL data, only blood tissue was used in this study. Thus, we may miss many pQTL target genes. Therefore, more pQTL target genes may be obtained if more large-scale data from schizophrenia relevant tissues are available. In addition, we mainly focused on protein-coding genes. Other target genes which encode non-coding RNAs may also play roles in the pathogenesis of schizophrenia. Besides, we did meta-analyses across distinct tissue types firstly because blood was used as a surrogate for brain tissues in schizophrenia studies^56–58^. We aimed to find the genes expressed consistently in these schizophrenia-related tissues. Secondly, meta-analysis applied to different tissues to identify non-tissue specific genes was commonly used^13,59^. This did have some limitations since different tissues have different gene expression patterns, which may introduce heterogeneity.

In conclusion, we acquired SNPs associated with schizophrenia and provided a comprehensive annotation for all susceptibility loci. We identified 934 functional SNPs in brain and 1300 in blood, including 66 missense SNPs, 1 splicing SNP, 119 UTR SNPs, 89 promoter, and 659/1028 enhancer SNPs. These promoter and enhancer SNPs were enriched for disruption of 34 TFs. H3K27ac, H3K36me3, H3K4me1, and H3K4me3 were more likely to be related to the development of schizophrenia. meQTL analyses for the functional SNPs showed that 516 functional SNPs in brain would affect DNA methylation levels of 224 genes. eQTL analyses for the functional SNPs identified 380 target genes. pQTL analyses showed that 19 proteins might be affected by functional SNPs. Finally, we acquired 447 target protein-coding genes. One hundred and sixty-eight of these genes (37.58%) were differentially expressed between schizophrenia cases and controls, and 103 (23.04%) were related to nervous system phenotype in mouse models. Besides, 260 (58.17%) could be correlated with schizophrenia-related pathways or related genes directly or through gene–gene interaction. We prioritized 10 target genes for schizophrenia, *CLU* and *MAPK3* were identified by GWASs, but the other two genes (*CSNK2B* and *SYNGAP1*) were novel. Our results may offer holistic guides for post-GWAS functional experiments.

## Supplementary information

supplementary figure legends

supplementary Figure S1

supplementary Table S1

supplementary Table S2

supplementary Table S3

supplementary Table S4

supplementary Table S5

supplementary Table S6

supplementary Table S7

supplementary Table S8

supplementary Table S9

supplementary Table S10
