# Supplementary material for: Comprehensive functional annotation of susceptibility SNPs prioritized 10 genes for schizophrenia
Source: Transl Psychiatry. 2019 Jan 31;9:56. doi: 10.1038/s41398-019-0398-5 (PMC6355777; doi:10.1038/s41398-019-0398-5)
Supplement: Supplementary file 1 — supplementary figure legends [file 41398_2019_398_MOESM1_ESM.doc]

Figure S1. Gene-gene interaction results of the unexplained target genes. A. 225 target protein-coding genes which were not mapped to known schizophrenia related pathways interact with genes in known schizophrenia pathways. B. 62 target protein-coding genes interact with known schizophrenia related genes, including 6 schizophrenia elite genes.
