# Supplementary figures and images for: Comprehensive functional annotation of susceptibility SNPs prioritized 10 genes for schizophrenia
Source: Transl Psychiatry. 2019 Jan 31;9:56. doi: 10.1038/s41398-019-0398-5 (PMC6355777; doi:10.1038/s41398-019-0398-5)

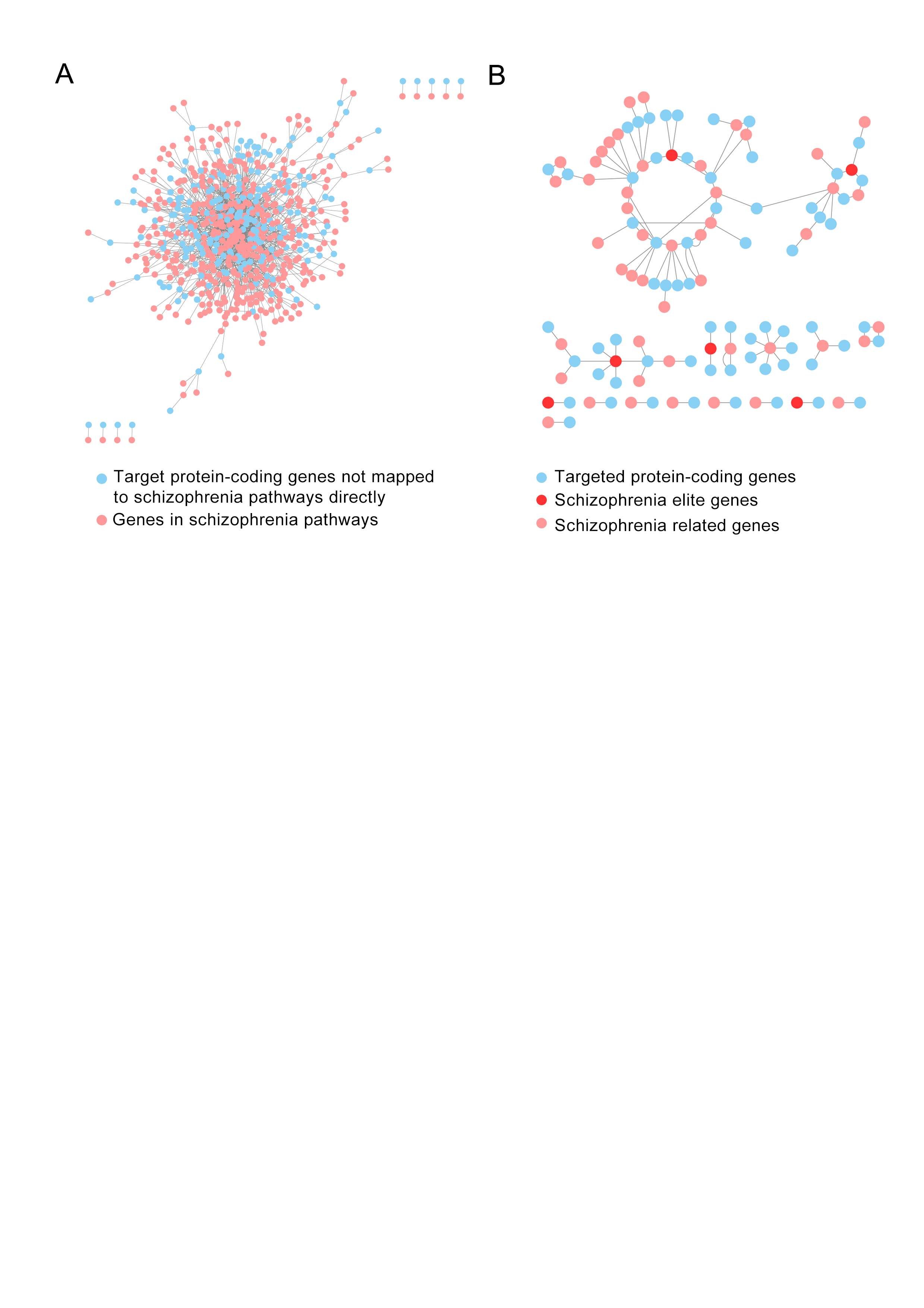

Supplement: Supplementary file 2 — supplementary Figure S1 [file 41398_2019_398_MOESM2_ESM.tif]
