# Supplementary material for: Comprehensive functional annotation of susceptibility SNPs prioritized 10 genes for schizophrenia
Source: Transl Psychiatry. 2019 Jan 31;9:56. doi: 10.1038/s41398-019-0398-5 (PMC6355777; doi:10.1038/s41398-019-0398-5)
Supplement: Supplementary file 3 — supplementary Table S1 [file 41398_2019_398_MOESM3_ESM.doc]

Table S1. Tissues or cells in Roadmap used to obtain the chromatin states information for intronic or intergenic SNPs and do the enrichment of histone modification.

| **ID** | **Description** | **Type** |
| --- | --- | --- |
| E053 | Cortex derived primary cultured neurospheres | Brain |
| E054 | Ganglion Eminence derived primary cultured neurospheres |  |
| E067 | Brain Angular Gyrus |  |
| E068 | Brain Anterior Caudate |  |
| E069 | Brain Cingulate Gyrus |  |
| E070 | Brain Germinal Matrix |  |
| E071 | Brain Hippocampus Middle |  |
| E072 | Brain Inferior Temporal Lobe |  |
| E073 | Brain Dorsolateral Prefrontal Cortex |  |
| E074 | Brain Substantia Nigra |  |
| E081 | Fetal Brain Male |  |
| E082 | Fetal Brain Female |  |
| E125 | NH-A Astrocytes Primary Cells |  |
| E029 | Primary monocytes from peripheral blood | Blood |
| E030 | Primary neutrophils from peripheral blood |  |
| E031 | Primary B cells from cord blood |  |
| E032 | Primary B cells from peripheral blood |  |
| E033 | Primary T cells from cord blood |  |
| E034 | Primary T cells from peripheral blood |  |
| E037 | Primary T helper memory cells from peripheral blood 2 |  |
| E038 | Primary T helper naive cells from peripheral blood |  |
| E039 | Primary T helper naive cells from peripheral blood |  |
| E040 | Primary T helper memory cells from peripheral blood 1 |  |
| E041 | Primary T helper cells PMA-I stimulated |  |
| E042 | Primary T helper 17 cells PMA-I stimulated |  |
| E043 | Primary T helper cells from peripheral blood |  |
| E044 | Primary T regulatory cells from peripheral blood |  |
| E045 | Primary T cells effector/memory enriched from peripheral blood |  |
| E046 | Primary Natural Killer cells from peripheral blood |  |
| E047 | Primary T CD8+ naive cells from peripheral blood |  |
| E048 | Primary T CD8+ memory cells from peripheral blood |  |
| E062 | Primary mononuclear cells from peripheral blood |  |
| E116 | GM12878 Lymphoblastoid Cells |  |
| E124 | Monocytes-CD14+ RO01746 Primary Cells |  |
