# Supplementary material for: Comprehensive functional annotation of susceptibility SNPs prioritized 10 genes for schizophrenia
Source: Transl Psychiatry. 2019 Jan 31;9:56. doi: 10.1038/s41398-019-0398-5 (PMC6355777; doi:10.1038/s41398-019-0398-5)
Supplement: Supplementary file 4 — supplementary Table S2 [file 41398_2019_398_MOESM4_ESM.doc]

Table S2. Details of the 347 schizophrenia related SNPs.

| **SNP** | **Chr** | **hg19_position** | **Ethnic** |
| --- | --- | --- | --- |
| rs78681500 | 2 | 201187316 | EAS |
| rs7757969 | 6 | 112132032 | EAS |
| rs4479915 | 6 | 165075601 | EAS |
| rs2269372 | 23 | 153207545 | EAS |
| rs2073499 | 3 | 50374293 | EAS |
| rs16887244 | 8 | 38031345 | EAS |
| rs1635 | 6 | 28227604 | EAS |
| rs1518395 | 2 | 58208074 | EAS |
| rs11534004 | 7 | 113467444 | EAS |
| rs115070292 | 6 | 29530974 | EAS |
| rs111782145 | 6 | 30873508 | EAS |
| rs11038167 | 11 | 44843134 | EAS |
| rs10883795 | 10 | 104654577 | EAS |
| rs10883765 | 10 | 104456686 | EAS |
| rs1051061 | 2 | 58316814 | EAS |
| rs10489202 | 1 | 167903079 | EAS |
| rs9960767 | 18 | 53155002 | EUR |
| rs9922678 | 16 | 9946319 | EUR |
| rs9841616 | 3 | 181167585 | EUR |
| rs971570 | 6 | 30172513 | EUR |
| rs9665626 | 10 | 104300638 | EUR |
| rs9636107 | 18 | 53200117 | EUR |
| rs9607782 | 22 | 41587556 | EUR |
| rs950169 | 15 | 84706461 | EUR |
| rs9468413 | 6 | 28689672 | EUR |
| rs9461856 | 6 | 33395199 | EUR |
| rs9420 | 11 | 57510294 | EUR |
| rs9398171 | 6 | 108983527 | EUR |
| rs9274623 | 6 | 32635998 | EUR |
| rs9269028 | 6 | 32437531 | EUR |
| rs9268895 | 6 | 32431962 | EUR |
| rs9261290 | 6 | 30038647 | EUR |
| rs885912 | 6 | 30174633 | EUR |
| rs853685 | 6 | 28288785 | EUR |
| rs853681 | 6 | 28296650 | EUR |
| rs853679 | 6 | 28296863 | EUR |
| rs853676 | 6 | 28299687 | EUR |
| rs832190 | 3 | 63842629 | EUR |
| rs832187 | 3 | 63833050 | EUR |
| rs8321 | 6 | 30032522 | EUR |
| rs8082590 | 17 | 17958402 | EUR |
| rs8044995 | 16 | 68189340 | EUR |
| rs8042374 | 15 | 78908032 | EUR |
| rs7951870 | 11 | 46373311 | EUR |
| rs7940866 | 11 | 130817579 | EUR |
| rs79212538 | 5 | 151993104 | EUR |
| rs7914558 | 10 | 104775908 | EUR |
| rs7893279 | 10 | 18745105 | EUR |
| rs7819570 | 8 | 89588626 | EUR |
| rs7801375 | 7 | 131567263 | EUR |
| rs778371 | 2 | 233743109 | EUR |
| rs77502336 | 11 | 123394636 | EUR |
| rs7749823 | 6 | 26158079 | EUR |
| rs7746199 | 6 | 27261324 | EUR |
| rs77149735 | 1 | 243555105 | EUR |
| rs76355118 | 2 | 149412005 | EUR |
| rs7601312 | 2 | 229320093 | EUR |
| rs75968099 | 3 | 36858583 | EUR |
| rs75575209 | 2 | 58138192 | EUR |
| rs7527939 | 1 | 210536025 | EUR |
| rs7523273 | 1 | 207977083 | EUR |
| rs75059851 | 11 | 133822569 | EUR |
| rs7432375 | 3 | 136288405 | EUR |
| rs7405404 | 16 | 13749859 | EUR |
| rs735765 | 6 | 28170297 | EUR |
| rs73396800 | 6 | 32914726 | EUR |
| rs73388146 | 6 | 25170668 | EUR |
| rs73229090 | 8 | 27442127 | EUR |
| rs73191547 | 8 | 10033425 | EUR |
| rs72986630 | 19 | 11849736 | EUR |
| rs72934570 | 18 | 53533189 | EUR |
| rs72700829 | 1 | 150540181 | EUR |
| rs715170 | 18 | 53795514 | EUR |
| rs7085104 | 10 | 104628873 | EUR |
| rs6984242 | 8 | 60700469 | EUR |
| rs6932590 | 6 | 27248931 | EUR |
| rs6918586 | 6 | 26097384 | EUR |
| rs6901575 | 6 | 28250984 | EUR |
| rs6878284 | 5 | 101769726 | EUR |
| rs679087 | 12 | 29917265 | EUR |
| rs67682613 | 6 | 31826705 | EUR |
| rs6704768 | 2 | 233592501 | EUR |
| rs6704641 | 2 | 200164252 | EUR |
| rs6703335 | 1 | 243608967 | EUR |
| rs6670165 | 1 | 177280121 | EUR |
| rs66691851 | 3 | 136154828 | EUR |
| rs6466055 | 7 | 104929064 | EUR |
| rs6461049 | 7 | 2017445 | EUR |
| rs6456834 | 6 | 28700352 | EUR |
| rs6434928 | 2 | 198304577 | EUR |
| rs62402013 | 6 | 26915000 | EUR |
| rs62392365 | 6 | 25371369 | EUR |
| rs62378245 | 5 | 88743962 | EUR |
| rs62200787 | 2 | 185727353 | EUR |
| rs6065094 | 20 | 37453194 | EUR |
| rs6002655 | 22 | 42603814 | EUR |
| rs59979824 | 2 | 193848340 | EUR |
| rs5937157 | 23 | 68377126 | EUR |
| rs5757717 | 22 | 39942234 | EUR |
| rs56205728 | 15 | 40567237 | EUR |
| rs55834529 | 6 | 27072542 | EUR |
| rs55833108 | 10 | 104741583 | EUR |
| rs55661361 | 11 | 124613957 | EUR |
| rs4801131 | 18 | 52752700 | EUR |
| rs4766428 | 12 | 110723245 | EUR |
| rs4711350 | 6 | 33741716 | EUR |
| rs4702 | 15 | 91426560 | EUR |
| rs4687552 | 3 | 52838402 | EUR |
| rs4648845 | 1 | 2387101 | EUR |
| rs4642619 | 8 | 111487468 | EUR |
| rs4523957 | 17 | 2208899 | EUR |
| rs4391122 | 5 | 60598543 | EUR |
| rs4388249 | 5 | 109036066 | EUR |
| rs4330281 | 3 | 17859366 | EUR |
| rs427230 | 20 | 62155111 | EUR |
| rs4129585 | 8 | 143312933 | EUR |
| rs41266839 | 6 | 26409890 | EUR |
| rs3857546 | 6 | 26157762 | EUR |
| rs385492 | 6 | 29649547 | EUR |
| rs3849046 | 5 | 137851192 | EUR |
| rs3800316 | 6 | 27256102 | EUR |
| rs3798869 | 6 | 84328660 | EUR |
| rs3788567 | 22 | 40006035 | EUR |
| rs3768644 | 2 | 72361505 | EUR |
| rs3756766 | 5 | 137673167 | EUR |
| rs3735025 | 7 | 137074844 | EUR |
| rs36068923 | 8 | 111485761 | EUR |
| rs35998080 | 1 | 73278615 | EUR |
| rs35518360 | 4 | 103146890 | EUR |
| rs34706883 | 6 | 27805255 | EUR |
| rs3132565 | 6 | 31102964 | EUR |
| rs3131783 | 6 | 30932068 | EUR |
| rs3131296 | 6 | 32172993 | EUR |
| rs3130356 | 6 | 30336663 | EUR |
| rs301797 | 1 | 8487323 | EUR |
| rs3001723 | 1 | 44037685 | EUR |
| rs2973155 | 5 | 152608619 | EUR |
| rs2949006 | 2 | 200715388 | EUR |
| rs2945232 | 8 | 8098038 | EUR |
| rs2910032 | 5 | 152540354 | EUR |
| rs2905426 | 19 | 19478022 | EUR |
| rs2905424 | 19 | 19473445 | EUR |
| rs2851447 | 12 | 123665113 | EUR |
| rs2844776 | 6 | 30171827 | EUR |
| rs281768 | 2 | 200825240 | EUR |
| rs2799077 | 6 | 28234597 | EUR |
| rs2693698 | 14 | 99719219 | EUR |
| rs2660304 | 1 | 98512127 | EUR |
| rs2596500 | 6 | 31321267 | EUR |
| rs2535627 | 3 | 52845105 | EUR |
| rs2523722 | 6 | 30165273 | EUR |
| rs2523721 | 6 | 30166266 | EUR |
| rs2517614 | 6 | 30163955 | EUR |
| rs2517611 | 6 | 30169327 | EUR |
| rs2517610 | 6 | 30170280 | EUR |
| rs2514218 | 11 | 113392994 | EUR |
| rs2381759 | 2 | 146428031 | EUR |
| rs2373000 | 2 | 37592628 | EUR |
| rs2332700 | 14 | 72417326 | EUR |
| rs2232423 | 6 | 28366151 | EUR |
| rs215411 | 4 | 23423603 | EUR |
| rs211829 | 7 | 110048893 | EUR |
| rs2102949 | 12 | 123676763 | EUR |
| rs210152 | 6 | 33515520 | EUR |
| rs2068012 | 14 | 30190316 | EUR |
| rs2053079 | 19 | 30987423 | EUR |
| rs2021722 | 6 | 30174131 | EUR |
| rs2018916 | 16 | 63700508 | EUR |
| rs2007044 | 12 | 2344960 | EUR |
| rs1936365 | 6 | 28268452 | EUR |
| rs1778508 | 6 | 28229881 | EUR |
| rs17750424 | 6 | 27701122 | EUR |
| rs17749927 | 6 | 27669976 | EUR |
| rs17720293 | 6 | 28214698 | EUR |
| rs17693963 | 6 | 27710165 | EUR |
| rs17691888 | 10 | 18734528 | EUR |
| rs17662626 | 2 | 193984621 | EUR |
| rs17504622 | 5 | 152654479 | EUR |
| rs17194490 | 3 | 2547786 | EUR |
| rs171748 | 5 | 60499131 | EUR |
| rs1702294 | 1 | 98501984 | EUR |
| rs16897515 | 6 | 27278020 | EUR |
| rs16867576 | 5 | 88746331 | EUR |
| rs1679709 | 6 | 28228342 | EUR |
| rs1625579 | 1 | 98502934 | EUR |
| rs1611255 | 6 | 29751005 | EUR |
| rs1538774 | 1 | 243544827 | EUR |
| rs1501357 | 5 | 45364875 | EUR |
| rs1498232 | 1 | 30433951 | EUR |
| rs144447022 | 6 | 29244219 | EUR |
| rs14403 | 1 | 243663893 | EUR |
| rs1419183 | 6 | 28242794 | EUR |
| rs140505938 | 1 | 150031490 | EUR |
| rs1378559 | 23 | 21380266 | EUR |
| rs1367858 | 2 | 201160771 | EUR |
| rs1339227 | 6 | 73155701 | EUR |
| rs133047 | 22 | 41027819 | EUR |
| rs13240464 | 7 | 110898915 | EUR |
| rs13218875 | 6 | 27884012 | EUR |
| rs13217619 | 6 | 28306671 | EUR |
| rs13217239 | 6 | 27254967 | EUR |
| rs13213152 | 6 | 28349698 | EUR |
| rs13212651 | 6 | 27806985 | EUR |
| rs13211507 | 6 | 28257377 | EUR |
| rs13205911 | 6 | 28124114 | EUR |
| rs13199772 | 6 | 27834085 | EUR |
| rs13198716 | 6 | 26582035 | EUR |
| rs13198474 | 6 | 25874423 | EUR |
| rs13194781 | 6 | 27815639 | EUR |
| rs13194504 | 6 | 28630691 | EUR |
| rs13194053 | 6 | 27143883 | EUR |
| rs13107325 | 4 | 103188709 | EUR |
| rs13096210 | 3 | 180599318 | EUR |
| rs12991836 | 2 | 145141541 | EUR |
| rs12966547 | 18 | 52752017 | EUR |
| rs12908161 | 15 | 85207825 | EUR |
| rs12903146 | 15 | 61854663 | EUR |
| rs12887734 | 14 | 104046834 | EUR |
| rs12845396 | 23 | 6029533 | EUR |
| rs12826178 | 12 | 57622371 | EUR |
| rs12823424 | 12 | 2514112 | EUR |
| rs12807809 | 11 | 124606285 | EUR |
| rs12704290 | 7 | 86427626 | EUR |
| rs12691307 | 16 | 29939877 | EUR |
| rs12668848 | 7 | 2020995 | EUR |
| rs1261117 | 18 | 52949657 | EUR |
| rs12474906 | 2 | 28033538 | EUR |
| rs12446487 | 16 | 58671815 | EUR |
| rs12421382 | 11 | 109378071 | EUR |
| rs12360997 | 11 | 24385569 | EUR |
| rs1233578 | 6 | 28712247 | EUR |
| rs12325245 | 16 | 58681393 | EUR |
| rs12148337 | 15 | 70589272 | EUR |
| rs12129573 | 1 | 73768366 | EUR |
| rs11995572 | 8 | 89592083 | EUR |
| rs1198588 | 1 | 98552832 | EUR |
| rs11965538 | 6 | 28239915 | EUR |
| rs11874716 | 18 | 52750688 | EUR |
| rs11740474 | 5 | 153680747 | EUR |
| rs11717383 | 3 | 52287468 | EUR |
| rs117074560 | 6 | 96459651 | EUR |
| rs11693094 | 2 | 185601420 | EUR |
| rs11685299 | 2 | 225391296 | EUR |
| rs11682175 | 2 | 57987593 | EUR |
| rs11532322 | 12 | 123731423 | EUR |
| rs112973353 | 14 | 104537680 | EUR |
| rs11210892 | 1 | 44100084 | EUR |
| rs11191580 | 10 | 104906211 | EUR |
| rs11191419 | 10 | 104612335 | EUR |
| rs1117490 | 6 | 30170510 | EUR |
| rs11139497 | 9 | 84739941 | EUR |
| rs111294930 | 5 | 152177121 | EUR |
| rs1106568 | 4 | 176861301 | EUR |
| rs11027857 | 11 | 24403620 | EUR |
| rs10890032 | 1 | 73789324 | EUR |
| rs10883832 | 10 | 104871279 | EUR |
| rs10791097 | 11 | 130718630 | EUR |
| rs10789369 | 1 | 73824909 | EUR |
| rs10520163 | 4 | 170626552 | EUR |
| rs10503253 | 8 | 4180844 | EUR |
| rs10456362 | 6 | 28221816 | EUR |
| rs10425465 | 19 | 33897934 | EUR |
| rs1023500 | 22 | 42340844 | EUR |
| rs1006737 | 12 | 2345295 | EUR |
| rs999494 | 2 | 73157395 | META |
| rs9859557 | 3 | 180830910 | META |
| rs9292918 | 5 | 45301035 | META |
| rs895526 | 2 | 200162425 | META |
| rs867743 | 8 | 60694647 | META |
| rs8058130 | 16 | 64371163 | META |
| rs8012642 | 14 | 84669481 | META |
| rs796364 | 2 | 200716119 | META |
| rs783540 | 15 | 83254708 | META |
| rs7733403 | 5 | 140154215 | META |
| rs7597593 | 2 | 185533580 | META |
| rs758129 | 15 | 89900887 | META |
| rs758117 | 12 | 2513309 | META |
| rs7438 | 4 | 170642246 | META |
| rs73219805 | 8 | 26272768 | META |
| rs72843506 | 17 | 19946287 | META |
| rs7010876 | 8 | 89264751 | META |
| rs6903570 | 6 | 64866857 | META |
| rs6846161 | 4 | 176866459 | META |
| rs6804239 | 3 | 161780488 | META |
| rs6500596 | 16 | 4470027 | META |
| rs6430491 | 2 | 134840967 | META |
| rs62152284 | 2 | 104984387 | META |
| rs619091 | 11 | 133884137 | META |
| rs61882743 | 11 | 46548754 | META |
| rs61041384 | 12 | 123644043 | META |
| rs5757730 | 22 | 39967430 | META |
| rs56775891 | 18 | 77575613 | META |
| rs56007784 | 17 | 1290950 | META |
| rs4949526 | 1 | 30432219 | META |
| rs4240748 | 12 | 92246786 | META |
| rs3825845 | 15 | 78910258 | META |
| rs3814881 | 16 | 30000901 | META |
| rs3732386 | 3 | 36871993 | META |
| rs35978510 | 1 | 243776117 | META |
| rs35607894 | 14 | 72408638 | META |
| rs35225048 | 15 | 61856263 | META |
| rs35065479 | 17 | 55736735 | META |
| rs323167 | 7 | 78336677 | META |
| rs302321 | 12 | 29928388 | META |
| rs2980436 | 8 | 8092025 | META |
| rs2970610 | 1 | 44097530 | META |
| rs2944823 | 7 | 71795470 | META |
| rs28735056 | 18 | 77622879 | META |
| rs28607014 | 12 | 117708611 | META |
| rs2767713 | 9 | 84799537 | META |
| rs2764766 | 5 | 127213625 | META |
| rs2577831 | 3 | 52628056 | META |
| rs2383377 | 14 | 33257914 | META |
| rs2247870 | 5 | 90151589 | META |
| rs217331 | 6 | 84344689 | META |
| rs2159100 | 12 | 2346393 | META |
| rs214467 | 7 | 110858268 | META |
| rs2057884 | 7 | 104930250 | META |
| rs17687067 | 8 | 17036201 | META |
| rs17630293 | 2 | 201146776 | META |
| rs172531 | 1 | 8495590 | META |
| rs17234749 | 11 | 24381898 | META |
| rs16880831 | 8 | 111561505 | META |
| rs1615350 | 12 | 123650335 | META |
| rs160593 | 6 | 105466332 | META |
| rs13266463 | 8 | 143403693 | META |
| rs13261217 | 8 | 4183057 | META |
| rs13164092 | 5 | 137840860 | META |
| rs12623170 | 2 | 28020157 | META |
| rs12619354 | 2 | 194002782 | META |
| rs12543645 | 8 | 10246325 | META |
| rs12129037 | 1 | 150492114 | META |
| rs1198589 | 1 | 98550411 | META |
| rs1191551 | 14 | 30000405 | META |
| rs11722779 | 4 | 103827488 | META |
| rs11708578 | 3 | 2515894 | META |
| rs113568682 | 5 | 152299754 | META |
| rs112537273 | 8 | 38248306 | META |
| rs112509803 | 7 | 24735004 | META |
| rs11210195 | 1 | 73749283 | META |
| rs11191424 | 10 | 104625886 | META |
| rs111364339 | 10 | 64857872 | META |
| rs10940346 | 5 | 49806042 | META |
| rs10894308 | 11 | 130891895 | META |
| rs10786736 | 10 | 104849116 | META |
| rs10510653 | 3 | 32058559 | META |
| rs10148671 | 14 | 29469373 | META |
