# Supplementary material for: Comprehensive functional annotation of susceptibility SNPs prioritized 10 genes for schizophrenia
Source: Transl Psychiatry. 2019 Jan 31;9:56. doi: 10.1038/s41398-019-0398-5 (PMC6355777; doi:10.1038/s41398-019-0398-5)
Supplement: Supplementary file 5 — supplementary Table S3 [file 41398_2019_398_MOESM5_ESM.doc]

Table S3. The results of variant effect prediction software for the missense SNPs.

| **SNP** | **Gene** | **AA-variant** | **PROVEAN** | **SIFT** | **PolyPhen2** | **CADD** |
| --- | --- | --- | --- | --- | --- | --- |
| rs950169 | *ADAMTSL3* | T1660I |  |  |  | Deleterious |
| rs9394145 | *SYNGAP1* | R31C |  | Damaging |  |  |
| rs9268142 | *C6orf10* | Y463C |  | Damaging |  |  |
| rs9262143 | *PPP1R18* | G339R |  | Damaging | Probably damaging | Deleterious |
| rs9257834 | *OR12D2* | V47F | Deleterious | Damaging | Probably damaging | Deleterious |
| rs7775397 | *C6orf10* | K397Q |  | Damaging | Probably damaging |  |
| rs7757931 | *UBD* | C162F |  | Damaging |  | Deleterious |
| rs707962 | *HLA-DQA1* | I186S | Deleterious |  |  |  |
| rs707949 | *HLA-DQA1* | F179L | Deleterious | Damaging |  | Deleterious |
| rs707910 | *HLA-A* | R68K |  | Damaging |  | Deleterious |
| rs6957894 | *AC110781.3* | G203S | Deleterious |  |  |  |
| rs678 | *ITIH1* | E585V | Deleterious | Damaging | Probably damaging | Deleterious |
| rs62621284 | *SNX19* | P480L |  | Damaging |  | Deleterious |
| rs61742093 | *OR2B2* | I39T | Deleterious | Damaging |  |  |
| rs4584886 | *LRRC48* | R191W | Deleterious | Damaging | Probably damaging | Deleterious |
| rs3803405 | *ALPK3* | G579E |  | Damaging |  |  |
| rs3803403 | *ALPK3* | T414S |  | Damaging |  |  |
| rs3734543 | *BTN2A1* | G437A | Deleterious |  | Possibly damaging |  |
| rs35555795 | *BTN1A1* | P521S |  | Damaging | Possibly damaging |  |
| rs34788973 | *OR2B2* | A300S | Deleterious | Damaging | Probably damaging | Deleterious |
| rs34525648 | *SLC17A2* | I419I |  |  |  | Deleterious |
| rs3134900 | *MICB* | I121M |  |  | Probably damaging |  |
| rs3131787 | *SFTA2* | N37S | Deleterious |  |  |  |
| rs3130743 | *OR2J2* | T218A |  | Damaging |  |  |
| rs3130618 | *GPANK1* | R41L | Deleterious |  | Probably damaging | Deleterious |
| rs3116856 | *OR2J2* | V146A |  |  | Possibly damaging |  |
| rs3116855 | *OR2J2* | Y74H | Deleterious | Damaging |  | Deleterious |
| rs2955367 | *MYO15A* | W718G |  | Damaging |  |  |
| rs2955365 | *MYO15A* | A595T |  | Damaging |  | Deleterious |
| rs2523898 | *MUC22* | R109G |  | Damaging |  |  |
| rs2306899 | *DDHD2* | T186M |  | Damaging | Possibly damaging |  |
| rs2247870 | *GPR98* | V5876I |  |  |  | Deleterious |
| rs2233974 | *C6orf15* | L40F |  |  | Probably damaging |  |
| rs2076484 | *UBD* | L51S | Deleterious |  |  |  |
| rs2075800 | *HSPA1L* | E602K |  | Damaging | Probably damaging | Deleterious |
| rs2072803 | *BTN2A2* | A255P |  | Damaging |  |  |
| rs1801311 | *NDUFA6* | A35V |  | Damaging |  | Deleterious |
| rs1801265 | *DPYD* | R29C |  |  |  | Deleterious |
| rs1799999 | *PPP1R3A* | D905Y |  | Damaging | Probably damaging | Deleterious |
| rs16897515 | *POM121L2* | G644C | Deleterious | Damaging |  | Deleterious |
| rs13216828 | *BTN3A2* | S307N |  | Damaging |  |  |
| rs13195509 | *BTN2A1* | V207M |  |  | Probably damaging | Deleterious |
| rs13195402 | *BTN2A1* | W178C | Deleterious | Damaging | Probably damaging | Deleterious |
| rs13195401 | *BTN2A1* | W178L | Deleterious | Damaging | Probably damaging | Deleterious |
| rs13107325 | *SLC39A8* | A391T |  |  | Possibly damaging | Deleterious |
| rs13072536 | *ITIH4* | I85N | Deleterious |  | Probably damaging | Deleterious |
| rs130079 | *CCHCR1* | G664C |  |  |  | Deleterious |
| rs130076 | *CCHCR1* | R198W | Deleterious |  |  | Deleterious |
| rs130065 | *CCHCR1* | R192W | Deleterious | Damaging |  | Deleterious |
| rs1265754 | *C6orf10* | I150F |  |  |  | Deleterious |
| rs1193851 | *PCNXL3* | S458C |  | Damaging | Probably damaging | Deleterious |
| rs115817940 | *HLA-DRB5* | T106N | Deleterious |  |  |  |
| rs11539157 | *PJA1* | E606D |  |  | Probably damaging |  |
| rs1142323 | *HLA-DQA1* | E63G | Deleterious |  |  |  |
| rs1131165 | *HLA-B* | L17V |  |  |  | Deleterious |
| rs1131156 | *HLA-B* | S14W | Deleterious |  |  | Deleterious |
| rs11177 | *GNL3* | R39Q |  |  |  | Deleterious |
| rs10885 | *PRRC2A* | P2006S |  | Damaging | Probably damaging | Deleterious |
| rs1078112 | *AC110781.3* | V26E | Deleterious |  |  |  |
| rs1059535 | *HLA-A* | A174V | Deleterious | Damaging |  |  |
| rs1051788 | *MICB* | D136N | Deleterious |  | Probably damaging |  |
| rs1051168 | *NMB* | P73T | Deleterious |  |  |  |
| rs1051061 | *VRK2* | I167V |  |  | Possibly damaging | Deleterious |
| rs1046089 | *PRRC2A* | R1740H |  |  | Probably damaging | Deleterious |
| rs1029871 | *NEK4* | P225A | Deleterious | Damaging | Possibly damaging | Deleterious |
| rs1003878 | *C6orf10* | P161L | Deleterious |  |  |  |
