# Supplementary material for: Comprehensive functional annotation of susceptibility SNPs prioritized 10 genes for schizophrenia
Source: Transl Psychiatry. 2019 Jan 31;9:56. doi: 10.1038/s41398-019-0398-5 (PMC6355777; doi:10.1038/s41398-019-0398-5)
Supplement: Supplementary file 6 — supplementary Table S4 [file 41398_2019_398_MOESM6_ESM.doc]

Table S4. MicroRNA target SNPs.

| **SNP** | **Gene** | **miRNA** | **Effect** |
| --- | --- | --- | --- |
| rs9468298 | *ZNF192* | miR-361-5p/3074-3p/2115-3p | create |
|  |  | miR-1252 | decrease |
| rs9393716 | *BTN3A2* | miR-4718 | break |
| rs9379871 | *BTN3A2* | miR-766-3p/3614-3p | break |
|  |  | miR-4639-3p/376a-5p | enhance |
| rs9366655 | *BTN3A2* | miR-661 | enhance |
|  |  | miR-4774-5p | create |
|  |  | miR-4697-3p | break |
| rs9331950 | *CLU* | miR-4263 | break |
|  |  | miR-3658 | decrease |
| rs9301 | *ZNF323* | miR-5700/448 | break |
|  |  | miR-4729 | create |
|  |  | miR-4495 | decrease |
| rs9295759 | *ZNF192* | miR-577 | create |
|  |  | miR-4662a-3p | decrease |
| rs9272988 | *HLA-DQA1* | miR-4733-5p | create |
| rs9272986 | *HLA-DQA1* | miR-138-2-3p | create |
| rs9272979 | *HLA-DQA1* | miR-499b-5p | enhance |
| rs9272975 | *HLA-DQA1* | miR-3143 | break |
| rs9272972 | *HLA-DQA1* | miR-548an/3926/3714 | create |
|  |  | miR-4436b-3p | break |
| rs9272932 | *HLA-DQA1* | miR-620/432-5p/1270 | enhance |
| rs9198 | *PPAPDC1B* | miR-598 | create |
|  |  | miR-4789-3p | decrease |
|  |  | miR-466 | enhance |
| rs880090 | *GMIP* | miR-124-5p | break |
| rs77674559 | *HSPA1B* | miR-524-5p/520d-5p | break |
| rs7750526 | *ZKSCAN3* | miR-552/3165 | break |
|  |  | miR-4524b-3p/3689c/3689b-3p/3689a-3p | decrease |
|  |  | miR-4459 | create |
| rs7626551 | *SFMBT1* | miR-5008-3p | break |
|  |  | miR-4307 | decrease |
| rs7582536 | *RFTN2* | miR-4729 | enhance |
|  |  | miR-106a-3p | create |
| rs7476 | *CREB3L1* | miR-5006-5p/4710 | decrease |
|  |  | miR-4270/125a-3p | enhance |
|  |  | miR-196b-5p/196a-5p | break |
|  |  | miR-1289 | create |
| rs73409643 | *BRD2* | miR-545-3p/3120-3p | decrease |
|  |  | miR-29b-1-5p | break |
|  |  | miR-29a-5p | create |
|  |  | miR-24-2-5p/24-1-5p | enhance |
| rs73208513 | *DMTF1* | miR-hsa-let-7f-1-3p | enhance |
| rs7290134 | *TNFRSF13C* | miR-5008-3p | enhance |
|  |  | miR-3678-3p/3158-5p | break |
|  |  | miR-1205 | create |
| rs72841536 | *BTN3A2* | miR-195-3p/16-2-3p | create |
| rs7237 | *WDR73* | miR-499b-5p/4771 | break |
|  |  | miR-379-5p/3529-5p | create |
| rs7195 | *HLA-DRA* | miR-875-3p/5001-5p/4492 | enhance |
|  |  | miR-4728-3p | break |
|  |  | miR-4446-3p/3652 | decrease |
| rs709937 | *NGEF* | miR-5195-3p | decrease |
|  |  | miR-450b-5p | create |
|  |  | miR-450a-5p | break |
| rs708228 | *CTNND1* | miR-4733-3p | break |
|  |  | miR-1276 | enhance |
|  |  | miR-1276 | decrease |
| rs7047 | *SCAF1* | miR-654-5p/564/541-3p | create |
|  |  | miR-602/4462/3138 | break |
|  |  | miR-4739/1321 | decrease |
| rs696520 | *STAG1* | miR-4709-5p | create |
| rs6910549 | *SLC17A4* | miR-5193 | create |
|  |  | miR-4279/139-5p | break |
| rs6909 | *GATAD2A* | miR-3943/1225-3p | create |
|  |  | miR-2114-3p | break |
|  |  | miR-1276 | enhance |
| rs6826 | *DRG2* | miR-625-5p/4728-5p/3689c/3689b-3p/3689a-3p/3192/30b-3p/149-3p | break |
|  |  | miR-5196-5p/4747-5p/4646-5p/3202/3179/204-3p | create |
|  |  | miR-4763-3p/4716-3p/18a-3p/1207-5p | decrease |
| rs6769789 | *TMEM110* | miR-4769-5p/4654 | create |
|  |  | miR-4734 | break |
| rs62291435 | *FXR1* | miR-5011-5p | break |
| rs6227 | *FURIN* | miR-4274 | break |
|  |  | miR-4261/212-3p/132-3p | create |
| rs60530611 | *BAG4* | miR-491-5p/4745-5p/4481 | break |
|  |  | miR-4758-5p | decrease |
|  |  | miR-4524a-3p | create |
| rs511515 | *BAK1* | miR-4468 | create |
| rs4945816 | *FOXO3* | miR-377-3p | create |
| rs4702 | *FURIN* | miR-1912/1295b-5p | create |
| rs45527431 | *ABT1* | miR-3121-3p | break |
| rs3757150 | *BTN2A1* | miR-4740-3p | create |
| rs3757138 | *BTN3A2* | miR-610 | decrease |
|  |  | miR-556-5p | create |
| rs3735026 | *DGKI* | miR-4796-5p | break |
|  |  | miR-455-3p | create |
|  |  | miR-212-3p/132-3p | decrease |
| rs3735025 | *DGKI* | miR-4709-5p/132-3p | enhance |
|  |  | miR-212-3p | decrease |
| rs3734528 | *HIST1H4A* | miR-9-5p | decrease |
|  |  | miR-5680 | break |
| rs34273322 | *BTN3A1* | miR-708-3p | create |
|  |  | miR-606 | break |
| rs324015 | *STAT6* | miR-5572/4455/3650 | break |
|  |  | miR-4260/187-3p/1268b/1268a | create |
| rs3208733 | *BTN3A1* | miR-217 | decrease |
| rs3208169 | *HLA-DQA1* | miR-944/126-5p | decrease |
|  |  | miR-449b-3p | create |
| rs3189472 | *HLA-C* | miR-95 | break |
| rs3095153 | *SFTA2* | miR-4797-5p/4785 | create |
|  |  | miR-4766-5p/370/2861 | enhance |
|  |  | miR-4524b-3p | break |
|  |  | miR-2861 | decrease |
| rs3047288 | *BTN3A2* | miR-764/552 | break |
|  |  | miR-5095 | create |
| rs28551159 | *BTN3A2* | miR-5581-5p/4297 | break |
|  |  | miR-5001-3p/3194-3p | enhance |
| rs284858 | *C10orf26* | miR-95 | create |
|  |  | miR-761/4690-5p/421/3619-5p/214-3p | break |
|  |  | miR-149-5p | enhance |
| rs284857 | *C10orf26* | miR-376c | create |
| rs284856 | *C10orf26* | miR-4763-3p/211-3p/197-5p/1207-5p | decrease |
|  |  | miR-3910 | create |
|  |  | miR-31-5p | break |
| rs284854 | *C10orf26* | miR-625-5p/4271/4259 | decrease |
|  |  | miR-548u | break |
|  |  | miR-4725-3p/4665-5p | enhance |
| rs2420 | *BRP44* | miR-4646-3p | break |
| rs2298278 | *SUFU* | miR-500a-3p/3130-3p | create |
|  |  | miR-3126-3p | break |
| rs2135551 | *ADAMTSL3* | miR-3667-5p/3153 | decrease |
| rs2106072 | *TRIM26* | miR-4731-5p/4487 | break |
|  |  | miR-1296 | create |
| rs2077586 | *EMX1* | miR-345-3p | decrease |
|  |  | miR-3162-3p/3157-3p | create |
| rs1978 | *BTN3A2* | miR-888-5p | create |
|  |  | miR-216a | break |
| rs1977 | *BTN3A2* | miR-676-3p/202-5p | decrease |
| rs1807494 | *FAM109B* | miR-4506 | break |
| rs17884001 | *AS3MT* | miR-664-3p | enhance |
|  |  | miR-4755-3p/4435 | create |
|  |  | miR-4729 | decrease |
| rs17601029 | *ZNF592* | miR-892b/24-3p | enhance |
|  |  | miR-5003-3p | create |
|  |  | miR-4310 | break |
| rs17310286 | *BNIP3L* | miR-4496/4261/1252 | break |
|  |  | miR-383 | decrease |
| rs17114810 | *SUFU* | miR-620/576-3p/4308/4254/1270 | create |
|  |  | miR-4764-5p/4700-5p/4667-5p/4292 | break |
| rs17114808 | *SUFU* | miR-5586-3p/4635 | enhance |
|  |  | miR-4765 | decrease |
|  |  | miR-194-5p | break |
| rs1710 | *HLA-G* | miR-762/4498 | enhance |
|  |  | miR-593-3p/5001-5p/4417/3663-5p/1587 | decrease |
|  |  | miR-3158-5p | break |
| rs16894108 | *ZSCAN23* | miR-7-2-3p/7-1-3p | break |
|  |  | miR-524-5p/520d-5p/330-3p | create |
| rs1610696 | *HLA-G* | miR-5692a | enhance |
|  |  | miR-559/548y/548w/548o-5p/548n/548j/548i/548h-5p/548d-5p/548c-5p/548b-5p/548au-5p/548as-5p/548ar-5p/548aq-5p/548ap-5p/548am-5p/548ak/548ab/548a-5p | create |
|  |  | miR-548x-3p/548aq-3p/548am-3p/548aj-3p/548ah-3p/548ae | break |
| rs15622 | *MAU2* | miR-578 | create |
|  |  | miR-26a-2-3p/26a-1-3p | enhance |
| rs13964 | *MAU2* | miR-5008-5p/4319/1291/125b-5p/125a-5p | break |
|  |  | miR-4708-5p | decrease |
|  |  | miR-4663/3197 | create |
| rs1376607 | *DGKI* | miR-561-5p | break |
| rs133347 | *WBP2NL* | miR-374b-5p/369-3p | decrease |
|  |  | miR-3145-5p | create |
|  |  | miR-136-5p | break |
| rs13272 | *ATXN7* | miR-3678-3p | create |
|  |  | miR-3668 | break |
| rs13205911 | *ZNF192* | miR-4311 | decrease |
|  |  | miR-3923/302d-5p/302b-5p | create |
| rs12541 | *ESAM* | miR-5195-5p | create |
|  |  | miR-5008-3p | enhance |
| rs11818043 | *SUFU* | miR-542-5p | break |
|  |  | miR-492/3649/3144-5p | create |
|  |  | miR-4749-5p | enhance |
| rs11752496 | *ZKSCAN3* | miR-551b-5p | decrease |
|  |  | miR-548c-3p | enhance |
| rs11535 | *NGEF* | miR-4674 | enhance |
|  |  | miR-4489 | break |
|  |  | miR-4448 | decrease |
| rs1150666 | *ZNF192* | miR-493-5p/4643 | decrease |
|  |  | miR-4789-3p | break |
|  |  | miR-3177-5p | enhance |
| rs1142429 | *HLA-DQA1* | miR-4459 | break |
| rs1142414 | *HLA-DQA1* | miR-4653-5p/3921 | enhance |
|  |  | miR-4303 | create |
| rs1138373 | *HLA-DQA1* | miR-511 | break |
|  |  | miR-4742-5p/1264 | create |
|  |  | miR-4673/4645-5p | enhance |
| rs1131541 | *HLA-DRA* | miR-3614-5p | create |
| rs1130592 | *HLA-C* | miR-767-5p/1301 | decrease |
| rs1130538 | *HLA-C* | miR-7-2-3p/7-1-3p/570-3p | create |
|  |  | miR-4719 | break |
| rs1130148 | *HLA-DQA1* | miR-4784/4689/3150b-3p | create |
|  |  | miR-4653-5p/3921 | enhance |
| rs1130145 | *HLA-DQA1* | miR-891b | create |
|  |  | miR-646/4720-3p/4524b-5p/4524a-5p | break |
| rs1130142 | *HLA-DQA1* | miR-646 | decrease |
|  |  | miR-5588-3p/4720-3p/4524b-5p/4524a-5p | enhance |
|  |  | miR-4433-5p/188-3p | create |
|  |  | miR-3919 | break |
| rs1130126 | *HLA-DQA1* | miR-4791 | decrease |
|  |  | miR-3675-5p | break |
|  |  | miR-3181 | create |
| rs112863520 | *HLA-A* | miR-4728-5p/450a-3p/3150a-3p/149-3p | break |
| rs11235 | *NEK4* | miR-5093 | enhance |
|  |  | miR-5010-3p | break |
|  |  | miR-4720-3p/218-5p/2113 | create |
| rs111727905 | *FOXO3* | miR-4662a-3p | break |
|  |  | miR-4328 | create |
| rs10947436 | *LEMD2* | miR-802/514b-5p/513c-5p/4465/26b-5p/26a-5p/1297 | break |
|  |  | miR-4254 | decrease |
|  |  | miR-412 | enhance |
| rs10883798 | *AS3MT* | miR-4742-3p | create |
| rs10786736 | *NT5C2* | miR-488-5p | create |
|  |  | miR-1238 | decrease |
| rs1065045 | *HLA-DQA1* | miR-5194 | enhance |
|  |  | miR-4651 | decrease |
|  |  | miR-449b-5p/449a/34c-5p/34a-5p | break |
|  |  | miR-3688-5p | create |
| rs1064991 | *HLA-DQA1* | miR-4703-3p/20b-3p/1183 | break |
| rs1064717 | *HLA-DRB1* | miR-5683 | enhance |
|  |  | miR-4695-5p/4459 | create |
| rs1063320 | *HLA-G* | miR-4800-5p/4776-5p | create |
|  |  | miR-152/148b-3p/148a-3p | break |
| rs1061815 | *HLA-A* | miR-92a-2-5p | enhance |
|  |  | miR-608/4651 | decrease |
|  |  | miR-4664-5p/3907/342-5p | create |
|  |  | miR-4483/323a-5p/1293 | break |
| rs1060330 | *WDR82* | miR-873-3p/4677-3p | break |
|  |  | miR-3976 | enhance |
| rs1054930 | *YJEFN3* | miR-644a/3648 | create |
|  |  | miR-4322/4296/4265/3178/2277-5p | break |
| rs1054284 | *GATAD2A* | miR-3120-5p | break |
| rs1051434 | *MPHOSPH9* | miR-4474-3p | create |
|  |  | miR-3187-3p | break |
| rs1049709 | *HLA-C* | miR-764 | break |
|  |  | miR-566 | create |
|  |  | miR-4262/3166/181a-5p | enhance |
|  |  | miR-181d/181b-5p | decrease |
| rs1049633 | *DDR1* | miR-578 | decrease |
|  |  | miR-5193/2355-3p | break |
|  |  | miR-1248 | enhance |
| rs1048645 | *HLA-DQA1* | miR-4726-5p/4640-5p | decrease |
|  |  | miR-3919/3123 | break |
| rs1047997 | *CENPM* | miR-185-3p | break |
| rs1043782 | *BAG4* | miR-4762-3p | decrease |
| rs10430665 | *NT5C2* | miR-558/22-3p | create |
|  |  | miR-5095 | decrease |
| rs1042992 | *BNIP3L* | miR-5688/495 | create |
|  |  | miR-5680/3646 | decrease |
|  |  | miR-23c | enhance |
| rs1041885 | *HLA-DRA* | miR-5694 | break |
|  |  | miR-3156-5p | create |
| rs10282 | *GATAD2A* | miR-4487/3135b | break |
|  |  | miR-1203 | create |
