# Supplementary material for: Comprehensive functional annotation of susceptibility SNPs prioritized 10 genes for schizophrenia
Source: Transl Psychiatry. 2019 Jan 31;9:56. doi: 10.1038/s41398-019-0398-5 (PMC6355777; doi:10.1038/s41398-019-0398-5)
Supplement: Supplementary file 7 — supplementary Table S5 [file 41398_2019_398_MOESM7_ESM.doc]

Table S5. The function of enriched TFs.

1、The TFs enriched by promoter SNPs.

| **TF** | **SNPs** |
| --- | --- |
| BACH1 | rs4932178; rs7007361 |
| ESR2 | rs134885; rs9274657 |
| JUND | rs6933289; rs9272359 |
| KLF4 | rs11057238; rs133376; rs416571; rs9266797 |
| KLF5 | rs133376; rs416571; rs74618856; rs9266797; rs9845788 |
| MAFK | rs4932178; rs7007361 |
| MYC | rs1737082; rs200951 |
| NFKB1 | rs1611204; rs2233960; rs9274652 |

2、The TFs enriched by enhancer SNPs in brain tissues.

| **TF** | **SNPs** |
| --- | --- |
| DR5 | rs886424; rs72936329; rs3129984; rs2395401; rs215406; rs1633086; rs11633450 |
| EHF | rs943476; rs7833728; rs747050; rs72694915; rs7195942; rs487835; rs36053597; rs2368560; rs2295152; rs143734333; rs11903916; rs10954342 |
| ESR1 | rs9487723; rs9481191; rs8101938; rs6903652; rs6748419; rs55689856; rs3759115; rs208818; rs1633086; rs1629068; rs1610721; rs11223650; rs10774033 |
| MYOG | rs72694957; rs6803008; rs2494635; rs204994; rs17454953; rs133382; rs12093576 |
| MZF1 | rs9845120; rs9468368; rs9468350; rs9262617; rs7957096; rs7759803; rs7604700; rs73038442; rs700690; rs6945719; rs6456728; rs59695400; rs5758527; rs5758268; rs4788211; rs4721302; rs3800882; rs3212090; rs3130673; rs3129973; rs2855817; rs2523891; rs2523886; rs2494631; rs2276097; rs2263750; rs2238053; rs1790094; rs1498231; rs1429417; rs139146144; rs134891; rs12531315; rs12272795; rs1145173; rs11191356; rs10797426; rs1016388 |
| PLAG1 | rs871925; rs7832163; rs4730602; rs36043776; rs34269918; rs301792; rs3001723; rs2523893; rs1632909; rs134892; rs1269134; rs1265092; rs1264321 |
| RAR | rs886424; rs72936329; rs3129984; rs2395401; rs215406; rs1633086; rs11633450 |
| RXR | rs886424; rs72936329; rs3129984; rs2395401; rs215406; rs1633086; rs11633450 |
| TCF12 | rs6803008; rs2494635; rs204994; rs17454953; rs133382; rs12093576 |
| USF1 | rs7462788; rs67827555; rs3132089; rs187653; rs11167584 |
| USF2 | rs7462788; rs67827555; rs3132089; rs187653; rs17435276; rs11167584 |
| ZNF263 | rs9893573; rs9264622; rs893808; rs883563; rs76349903; rs73208509; rs72694957; rs72694915; rs67215852; rs6710294; rs6708345; rs6456825; rs61882712; rs60722866; rs59909085; rs5961396; rs59092643; rs57940349; rs56055628; rs55985421; rs4906379; rs4788211; rs4779046; rs4650963; rs4583255; rs4553407; rs36053597; rs35345226; rs34633710; rs34270954; rs34269918; rs324017; rs301792; rs2855802; rs2683634; rs2596473; rs2523599; rs2236951; rs215405; rs2143139; rs210142; rs2057884; rs204993; rs1718682; rs16373; rs1632909; rs150643353; rs1464209; rs139146144; rs134869; rs13236006; rs1264318; rs12573474; rs11779986; rs11688168; rs11623546; rs11589922; rs114326128; rs11372849; rs11191356; rs111659883; rs1016388; rs10132641 |

3、The TFs enriched by enhancer SNPs in blood tissues.

| **TF** | **SNPs** |
| --- | --- |
| DR5 | rs9272495; rs9272255; rs886424; rs72694919; rs3129984; rs1633086; rs11633450 |
| EGR1 | rs9899193; rs9276168; rs9273019; rs9273018; rs9272543; rs9272247; rs883563; rs62253583; rs62253582; rs5757717; rs57321508; rs56189111; rs4719366; rs4369854; rs35346340; rs352139; rs3129986; rs2921705; rs2858328; rs2517578; rs1811371; rs1639108; rs1610719; rs1464209; rs134889; rs12950555; rs12474906; rs11624408; rs11589922; rs113523485; rs111659883 |
| ESR1 | rs9487723; rs9481191; rs9358933; rs61937595; rs501220; rs3759115; rs3132619; rs2945247; rs2280550; rs208818; rs1633086; rs1610721; rs1610622; rs134885; rs12199613 |
| ETS1 | rs9633712; rs9393706; rs9379854; rs9273455; rs6926677; rs4788198; rs4788197; rs3801281; rs2523554; rs2337631; rs2256750; rs1770; rs11779986; rs11589922 |
| FLI1 | rs9633712; rs9393706; rs9379854; rs9273455; rs4788198; rs4788197; rs2523554; rs2256750 |
| KLF1 | rs9295964; rs9271982; rs9271980; rs9271979; rs9271878; rs9271877; rs9271550; rs9271331; rs7749904; rs7349597; rs72694964; rs3135406; rs3134784; rs3132930; rs2955584; rs2916068; rs2337631; rs1890184; rs1611422; rs145294790; rs11975035; rs11768541; rs113523485; rs11057204 |
| KLF4 | rs9295964; rs9271982; rs9271980; rs9271979; rs9271878; rs9271877; rs9271550; rs9271331; rs9264962; rs7749904; rs7349597; rs72694964; rs3135406; rs3134784; rs3132930; rs2916068; rs2370414; rs2337631; rs200327371; rs1894713; rs1611422; rs1610719; rs114132738; rs113523485; rs11057204 |
| MAFG | rs9852677; rs9274563; rs9270739; rs9269094; rs9268659; rs9263886; rs887465; rs876701; rs7928640; rs77034359; rs76451960; rs7615475; rs7484125; rs7252981; rs7096269; rs6906021; rs62244863; rs4759415; rs3800908; rs34349730; rs3132619; rs3130470; rs3130459; rs3129877; rs3096675; rs2571385; rs2395225; rs1737020; rs1727295; rs1611350; rs13217795; rs12891175; rs12155225; rs10617327; rs10207232 |
| NFE2L1 | rs9852677; rs9274563; rs9270739; rs9269094; rs9268659; rs9263886; rs887465; rs876701; rs7928640; rs77034359; rs76451960; rs7615475; rs7484125; rs7252981; rs7096269; rs6906021; rs62244863; rs4759415; rs3800908; rs34349730; rs3132619; rs3130470; rs3130459; rs3129877; rs3096675; rs2571385; rs2395225; rs1737020; rs1727295; rs1611350; rs13217795; rs12891175; rs12155225; rs10617327; rs10207232 |
| NR1H3 | rs9274620; rs9272255; rs9270438; rs3130621; rs2853995; rs2269373; rs200983; rs1790096; rs154069; rs13196986; rs11763870 |
| PAX5 | rs9271968; rs72692895; rs6968335; rs6901903; rs6713113; rs62135552; rs5004276; rs4790884; rs3887382; rs35757884; rs34747231; rs3129983; rs139238973; rs12293624; rs12293621; rs12161096; rs12157344; rs11589801; rs113571835; rs113339491; rs113278154; rs11121178; rs11038864; rs10888569; rs10448063 |
| RAR | rs9272495; rs9272255; rs886424; rs72694919; rs3129984; rs1633086; rs11633450 |
| RXR | rs9272495; rs9272255; rs886424; rs72694919; rs3129984; rs1633086; rs11633450 |
| RXRA | rs9274620; rs9272255; rs9270438; rs3130621; rs2853995; rs2269373; rs200983; rs1790096; rs154069; rs13196986; rs11763870; rs9909895; rs2284990; rs210133; rs1005598 |
| SOX2 | rs9273542; rs9272278; rs9265857; rs760587; rs3131060; rs2394885; rs2195510; rs17598603; rs1658810 |
| SP2 | rs9501082; rs9276168; rs9273175; rs9272543; rs9264962; rs883563; rs7751220; rs7544145; rs73210236; rs72687376; rs7126343; rs6925852; rs66772001; rs59092643; rs585522; rs5751220; rs57321508; rs56189111; rs4711107; rs352139; rs34130214; rs3129986; rs301792; rs2916073; rs2916068; rs2858328; rs256013; rs2524080; rs2370414; rs1894713; rs1811371; rs1610719; rs1464209; rs146399873; rs134889; rs13214169; rs12885251; rs12671113; rs1265178; rs12386951; rs11864819; rs11372849; rs10950413; rs1005599 |
| TFAP2C | rs9288280; rs55928150; rs3759115; rs3756766; rs375245; rs3129056; rs3129055; rs3129012; rs3128992; rs2745977; rs16832165; rs1633022; rs1632909; rs12576115; rs10846491; rs1059612 |
| USF1 | rs7614727; rs67827555; rs3132089; rs2523673; rs187653; rs154072 |
| USF2 | rs7614727; rs67827555; rs3132089; rs187653; rs17435276; rs154072 |
| ZFX | rs9895335; rs9501079; rs9272195; rs9272194; rs9271990; rs9271989; rs9271988; rs9268636; rs73068054; rs6466031; rs6002598; rs5751241; rs4898465; rs4711163; rs4424923; rs35324223; rs3212024; rs3130943; rs3130522; rs3129879; rs3129012; rs3096680; rs2581819; rs2565160; rs256017; rs216221; rs2012423; rs1824850; rs1790119; rs133348; rs13076193; rs12817892; rs12677360; rs12671113; rs1265947; rs11713763; rs11705236; rs1117488; rs11078400; rs10786701 |
| ZNF354C | rs9895335; rs9273164; rs9272462; rs9272249; rs9272226; rs9271965; rs9271738; rs9271293; rs9269873; rs9268097; rs9264587; rs9264586; rs805303; rs73490813; rs73206924; rs72849267; rs6584540; rs6485685; rs5758659; rs4932177; rs4434205; rs3131095; rs3114661; rs2948293; rs2534675; rs2235251; rs208810; rs2073526; rs186176074; rs177567; rs13030538; rs12447860; rs12176317; rs11745933; rs11689851; rs112940468; rs11191560 |

4、The function of enriched and differential expressed TFs during SH-SY5Y cell differentiation.

| **TF** | **Function** |
| --- | --- |
| JUND | Programmed neuronal cell death, plastic adaptations in the mature brain[^1^](#_ENREF_1). |
| NFKB1 | Involved in neurogenesis and brain pathology, stimulating adult axoneogenesis and treating central nervous system (CNS) axonopathies[^2^](#_ENREF_2). |
| ETS1 | Regulators for the mouse neurochondrin gene[^3^](#_ENREF_3). |
| KLF4 | Nerve Growth Factor-responsive immediate-early genes in neuronal differentiation[^4^](#_ENREF_4). |
| NFE2L1 | Expressed in cultured neurospheres[^5^](#_ENREF_5). |
| SOX2 | TF in neural stem cells and keeps the cells immature and proliferative[^6^](#_ENREF_6); contributed to the proliferative state of oligodendrocyte progenitor cells[^7^](#_ENREF_7). |
| SP2 | Conditional deletion of the SP2 disrupts the cell cycle in cortical neural stem cells (NSCs) and perturbs neurogenesis[^8^](#_ENREF_8). |
| TCF12 | A novel factor in mesodiencephalic dopaminergic neuronal development[^9^](#_ENREF_9); involved in the control of proliferating NSCs and progenitor cells[^10^](#_ENREF_10). |

1. Schlingensiepen KH*, et al*. THE ROLE OF JUN TRANSCRIPTION FACTOR EXPRESSION AND PHOSPHORYLATION IN NEURONAL DIFFERENTIATION, NEURONAL CELL-DEATH, AND PLASTIC ADAPTATIONS IN-VIVO. *Cellular And Molecular Neurobiology* 1994; **14**(5)**:** 487-505.

2. Haenold R*, et al*. NF-kappa B controls axonal regeneration and degeneration through cell-specific balance of RelA and p50 in the adult CNS. *Journal Of Cell Science* 2014; **127**(14)**:** 3052-3065.

3. Dateki M, Mochizuki R, Yanai K, Fukamizu A. Identification of the mouse neurochondrin promoter region and the responsible region for cell type specific gene regulation. *Neuroscience Letters* 2004; **356**(2)**:** 107-110.

4. Dijkmans TF*, et al*. Identification of new Nerve Growth Factor-responsive immediate-early genes. *Brain Research* 2009; **1249:** 19-33.

5. Molofsky AV*, et al*. Expression Profiling of Aldh1l1-Precursors in the Developing Spinal Cord Reveals Glial Lineage-Specific Genes and Direct Sox9-Nfe2l1 Interactions. *Glia* 2013; **61**(9)**:** 1518-1532.

6. Caglayan D, Lundin E, Kastemar M, Westermark B, Ferletta M. Sox21 inhibits glioma progression in vivo by forming complexes with Sox2 and stimulating aberrant differentiation. *International Journal Of Cancer* 2013; **133**(6)**:** 1345-1356.

7. Zhao C*, et al*. Sox2 Sustains Recruitment of Oligodendrocyte Progenitor Cells following CNS Demyelination and Primes Them for Differentiation during Remyelination. *The Journal of Neuroscience* 2015; **35**(33)**:** 11482-11499.

8. Liang HX*, et al*. Neural development is dependent on the function of specificity protein 2 in cell cycle progression. *Development* 2013; **140**(3)**:** 552-561.

9. Mesman S, Smidt MP. Tcf12 Is Involved in Early Cell-Fate Determination and Subset Specification of Midbrain Dopamine Neurons. *Frontiers In Molecular Neuroscience* 2017; **10**.

10. Uittenbogaard M, Chiaramello A. Expression of the bHLH transcription factor Tcf12 (ME1) gene is linked to the expansion of precursor cell populations during neurogenesis. *Brain Research Gene Expression Patterns* 2002; **1**(2)**:** 115-121.
