# Supplementary material for: Comprehensive functional annotation of susceptibility SNPs prioritized 10 genes for schizophrenia
Source: Transl Psychiatry. 2019 Jan 31;9:56. doi: 10.1038/s41398-019-0398-5 (PMC6355777; doi:10.1038/s41398-019-0398-5)
Supplement: Supplementary file 8 — supplementary Table S6 [file 41398_2019_398_MOESM8_ESM.pdf]

Table S6. QTLs analyses results of functional SNPs.

1、The most significant meQTL analyses results.

Gene	SNP	P_value	FDR	beta
MAD1L1	rs6950330	8.69E-116	2.57E-111	-0.10
HLA-C	rs9264586	8.65E-98	1.17E-93	-0.22
LSM1	rs11783967	7.76E-95	8.96E-91	-0.24
GABBR1	rs7757931	6.45E-94	7.03E-90	-0.18
HLA-DQB1	rs9274657	4.25E-85	2.71E-81	0.20
SNX19	rs2276097	4.77E-83	2.71E-79	-0.11
HAPLN4	rs735273	4.90E-78	2.06E-74	0.06
ZKSCAN8	rs9468298	5.47E-78	2.27E-74	0.06
BAG6	rs805302	4.67E-77	1.87E-73	-0.08
HLA-G	rs1710	6.08E-75	2.16E-71	0.06
SRR	rs2224770	7.00E-63	1.33E-59	0.14
HLA-F	rs1633086	3.97E-58	5.87E-55	-0.22
ZSCAN16-AS1	rs2275508	3.88E-57	5.38E-54	0.13
YJEFN3	rs1054930	3.96E-57	5.49E-54	0.11
FES	rs35346340	6.16E-56	7.97E-53	0.06
HLA-B	rs9266230	1.46E-55	1.85E-52	-0.10
GPSM3	rs204994	1.87E-54	2.23E-51	-0.04
MYO15A	rs2955365	2.15E-51	2.16E-48	-0.05
OR2J2	rs3116856	1.63E-50	1.56E-47	-0.05
ZSCAN12	rs7750526	1.03E-49	9.39E-47	-0.14
ZFP57	rs416571	1.41E-49	1.28E-46	0.23
NT5C2	rs35525740	1.89E-49	1.71E-46	-0.05
BRD2	rs176249	5.82E-49	5.11E-46	0.05
HLA-DQA1	rs707949	3.66E-48	3.06E-45	-0.10
OGFOD2	rs4275659	4.56E-47	3.57E-44	-0.03
IGSF9B	rs11223650	7.42E-47	5.74E-44	-0.10
LOC554223	rs1610645	5.62E-45	3.87E-42	0.10
FAM109B	rs6002597	6.60E-44	4.23E-41	0.02
DRG2	rs11078410	3.94E-41	2.11E-38	-0.03
C10orf32-ASMT	rs12219246	2.43E-40	1.24E-37	0.06
TRMT61A	rs12880077	1.64E-38	7.47E-36	-0.04
MICB	rs2596473	9.13E-38	3.97E-35	0.03
NAGA	rs4822088	1.15E-36	4.63E-34	0.03
TSNARE1	rs4976982	1.68E-35	6.25E-33	0.09
HLA-DRA	rs2395179	2.08E-35	7.72E-33	-0.03
PLCH2	rs16824398	2.15E-35	7.97E-33	0.04
MUC22	rs9501045	2.50E-35	9.22E-33	-0.08
NEK4	rs34017441	2.37E-33	7.60E-31	0.03
TRIM8	rs10786701	5.09E-32	1.48E-29	-0.07
BAK1	rs511515	7.96E-31	2.12E-28	-0.03

Gene	SNP	P_value	FDR	beta
GLT8D1	rs11177	1.79E-29	4.32E-27	-0.10
BCL11B	rs35604463	1.92E-27	3.99E-25	0.03
PRRC2A	rs3130621	7.82E-27	1.55E-24	0.03
BTN3A2	rs9379859	8.66E-27	1.71E-24	0.06
PPT2	rs3130281	2.54E-26	4.85E-24	0.03
ITIH4	rs13072536	3.97E-26	7.47E-24	0.06
TMEM110-MUSTN1	rs6769789	8.40E-26	1.54E-23	0.03
CNNM2	rs10786736	2.64E-25	4.67E-23	-0.05
HIST1H2BJ	rs9348746	3.73E-25	6.51E-23	-0.02
WHSC1L1	rs2016875	2.58E-24	4.22E-22	0.04
GPANK1	rs3130618	7.71E-24	1.22E-21	0.05
C6orf10	rs1003878	1.77E-23	2.72E-21	-0.21
FTCDNL1	rs796364	2.11E-23	3.21E-21	-0.04
C6orf15	rs2523893	3.25E-23	4.87E-21	-0.01
NT5DC2	rs1961958	3.05E-22	4.23E-20	0.03
ITIH1	rs678	3.49E-22	4.81E-20	-0.05
HCFC1	rs2266886	4.96E-22	6.74E-20	0.02
LEMD2	rs2182658	6.90E-22	9.25E-20	0.02
C2orf82	rs2675956	9.93E-22	1.31E-19	-0.03
HIST1H2BL	rs2747054	1.68E-21	2.18E-19	0.02
MPHOSPH9	rs1727334	7.50E-21	9.23E-19	0.06
PITPNM2	rs1727295	1.15E-20	1.39E-18	-0.02
NFATC3	rs9922594	1.64E-20	1.96E-18	0.03
NMB	rs11630887	2.33E-20	2.75E-18	-0.03
PEX10	rs10797426	2.39E-20	2.82E-18	0.03
LY6G5C	rs3130618	2.53E-20	2.97E-18	0.06
OR12D2	rs9257834	1.14E-19	1.27E-17	-0.03
PPP1R13B	rs2223937	1.80E-19	1.96E-17	0.02
WBP2NL	rs5758527	1.81E-19	1.97E-17	-0.01
SHISA8	rs9620001	2.45E-19	2.64E-17	0.05
TCF20	rs5758605	2.87E-18	2.80E-16	-0.04
PSORS1C1	rs2233960	4.96E-18	4.74E-16	-0.03
BAG5	rs28362583	6.06E-18	5.74E-16	0.03
TRIM26	rs885912	1.00E-17	9.29E-16	-0.03
CACNA1C	rs1016388	1.19E-17	1.10E-15	0.02
CCHCR1	rs1265092	1.41E-17	1.30E-15	-0.02
GRIN2A	rs9926303	1.89E-17	1.71E-15	0.02
NOTCH4	rs6457513	3.81E-16	3.04E-14	-0.03
GATAD2A	rs2905435	4.51E-16	3.57E-14	0.02
SUGP1	rs2315022	2.07E-15	1.53E-13	0.01
TMTC1	rs302317	3.51E-15	2.53E-13	-0.04
CENPM	rs5996096	5.40E-15	3.82E-13	0.01
DPCR1	rs3095153	5.51E-15	3.89E-13	-0.03

Gene	SNP	P_value	FDR	beta
HIST1H2AJ	rs2747054	5.89E-15	4.15E-13	0.01
CSNK2B	rs805302	9.99E-15	6.87E-13	-0.07
CHRNA2	rs2565065	1.36E-14	9.21E-13	-0.04
NMUR2	rs112345465	1.91E-14	1.28E-12	-0.04
TCF19	rs1265092	2.31E-14	1.53E-12	0.03
CACNB2	rs6482406	4.37E-14	2.81E-12	0.04
CDC25C	rs16373	6.37E-14	4.02E-12	0.03
POU5F1	rs887465	7.19E-14	4.51E-12	-0.04
CLU	rs9331950	1.30E-13	7.91E-12	0.02
UBD	rs7757931	1.42E-13	8.61E-12	-0.04
POM121L2	rs16897515	1.93E-13	1.15E-11	0.02
KLC1	rs59092643	2.13E-13	1.27E-11	-0.02
INO80E	rs4788211	2.44E-13	1.44E-11	0.01
MLN	rs1318691	3.34E-13	1.95E-11	0.02
NGEF	rs2675956	4.46E-13	2.57E-11	-0.02
RERE	rs301792	6.23E-13	3.53E-11	-0.01
MGAT3	rs9306336	1.01E-12	5.61E-11	-0.01
FLOT1	rs1059612	1.52E-12	8.25E-11	-0.03
FURIN	rs4702	1.68E-12	9.05E-11	-0.01
SPATS2L	rs3769446	2.10E-12	1.12E-10	0.09
HLA-DQA2	rs5021453	2.11E-12	1.13E-10	-0.03
SFTA2	rs3131787	2.33E-12	1.23E-10	-0.02
NAB2	rs324017	2.59E-12	1.37E-10	0.02
TNFRSF13C	rs9620001	4.27E-12	2.19E-10	-0.02
PRRT1	rs3130281	4.34E-12	2.23E-10	-0.02
CDK2AP1	rs4553407	5.71E-12	2.90E-10	0.03
C16orf92	rs4788211	7.63E-12	3.81E-10	0.01
HHAT	rs3765879	2.11E-11	9.97E-10	-0.02
DGKZ	rs876701	2.32E-11	1.09E-09	-0.01
GNL3	rs34017441	2.99E-11	1.39E-09	0.01
GTF2H4	rs3095153	3.61E-11	1.66E-09	0.04
IER3	rs3094125	5.58E-11	2.51E-09	-0.13
CUL3	rs3768886	6.75E-11	3.00E-09	0.04
ZNF165	rs3734573	7.25E-11	3.21E-09	0.01
PPM1M	rs11717383	1.23E-10	5.29E-09	0.01
DOC2A	rs4788211	1.58E-10	6.70E-09	-0.02
PPP1CC	rs12305489	1.70E-10	7.16E-09	-0.02
PPT2-EGFL8	rs3130281	1.89E-10	7.92E-09	0.01
TMEM243	rs73210240	2.20E-10	9.16E-09	-0.04
ABCB9	rs883562	2.71E-10	1.11E-08	0.03
CYP2D7	rs5758605	3.71E-10	1.50E-08	-0.02
SDCCAG8	rs1058305	3.73E-10	1.50E-08	-0.01
DDR1	rs1049633	3.85E-10	1.55E-08	0.02

Gene	SNP	P_value	FDR	beta
SGK223	rs2948286	4.00E-10	1.60E-08	-0.02
WDR73	rs11630887	7.70E-10	2.97E-08	0.02
MAN2A1	rs3819904	7.71E-10	2.98E-08	0.02
SEMA3B	rs6776145	9.09E-10	3.48E-08	0.02
SLCO4C1	rs3114661	9.18E-10	3.51E-08	0.02
LHFPL3	rs7807853	1.00E-09	3.81E-08	0.02
LST1	rs1800629	1.22E-09	4.57E-08	-0.02
RENBP	rs2269373	1.46E-09	5.41E-08	-0.01
ARL6IP4	rs4275659	1.46E-09	5.43E-08	-0.01
ANKRD44	rs1429417	1.68E-09	6.17E-08	0.01
WBP1L	rs284858	2.45E-09	8.79E-08	-0.01
GOLGA6L4	rs62029580	3.78E-09	1.32E-07	0.02
APOM	rs1046089	4.39E-09	1.52E-07	0.04
LOC100131289	rs111770007	5.19E-09	1.78E-07	0.02
HLA-H	rs1736937	8.79E-09	2.91E-07	0.02
FAM53C	rs16373	1.32E-08	4.25E-07	0.02
PLEKHO1	rs72694953	1.81E-08	5.72E-07	-0.04
KDM3B	rs16373	2.29E-08	7.11E-07	0.02
IMMP2L	rs37716	2.29E-08	7.12E-07	-0.02
ETF1	rs55985421	3.57E-08	1.08E-06	0.01
DPYD	rs1801265	3.89E-08	1.17E-06	-0.02
GIGYF2	rs6710294	6.33E-08	1.83E-06	-0.01
C6orf48	rs3115674	1.45E-07	3.96E-06	0.02
PPP1R16B	rs6129108	1.48E-07	4.02E-06	0.01
CYP17A1	rs12219246	1.85E-07	4.98E-06	0.01
ZKSCAN3	rs7750526	2.33E-07	6.15E-06	-0.01
HSPA1B	rs2075800	2.57E-07	6.74E-06	-0.01
LINC00336	rs511515	3.98E-07	1.01E-05	-0.02
PBX2	rs204993	4.66E-07	1.17E-05	0.02
LTA	rs1800629	6.55E-07	1.60E-05	0.02
ATXN7	rs7627690	7.81E-07	1.88E-05	-0.01
GALNT10	rs11740474	8.01E-07	1.92E-05	0.01
SREBF2	rs9607850	8.56E-07	2.04E-05	0.01
CYP2D6	rs28579115	8.87E-07	2.11E-05	0.03
BAG4	rs16887244	9.70E-07	2.29E-05	-0.01
ARHGAP1	rs61884292	9.76E-07	2.30E-05	0.01
RNF5	rs3134940	1.03E-06	2.42E-05	-0.01
3-Sep	rs5758527	1.18E-06	2.75E-05	-0.01
MAU2	rs2315022	1.30E-06	3.00E-05	0.01
TWF2	rs1060330	1.49E-06	3.39E-05	-0.02
ATG13	rs6485685	1.57E-06	3.56E-05	0.01
HSPA1L	rs2075800	1.79E-06	4.02E-05	0.01
C2orf47	rs281771	1.82E-06	4.08E-05	-0.01

**Gene**	**SNP**	**P_value**	**FDR**	**beta**
DNAH1	rs7622851	2.20E-06	4.86E-05	0.01
DRD2	rs17602038	2.87E-06	6.20E-05	0.01
BTN2A2	rs9393716	4.31E-06	8.99E-05	0.06
BTNL2	rs2001097	4.59E-06	9.51E-05	-0.02
PRRG2	rs57940349	5.56E-06	1.13E-04	-0.02
ZFYVE21	rs11846838	7.11E-06	1.42E-04	0.02
PBRM1	rs11177	1.22E-05	2.32E-04	0.01
C6orf47	rs805302	1.36E-05	2.55E-04	-0.01
TYW5	rs281771	1.84E-05	3.36E-04	0.01
SLC7A6	rs1971546	2.09E-05	3.76E-04	-0.01
HIST1H3I	rs200949	2.40E-05	4.26E-04	0.01
CCDC134	rs9607850	2.54E-05	4.48E-04	0.00
BTN2A1	rs3757150	2.60E-05	4.57E-04	-0.01
GPM6A	rs7673823	2.68E-05	4.70E-04	0.01
SATB2	rs6748419	2.72E-05	4.76E-04	0.01
CACNA2D2	rs2236950	3.16E-05	5.45E-04	0.01
HCG27	rs887465	3.19E-05	5.49E-04	-0.01
FGFR1	rs17182225	3.88E-05	6.56E-04	-0.01
SCGN	rs7771468	3.98E-05	6.71E-04	-0.01
SCAND2P	rs11630887	5.62E-05	9.13E-04	-0.02
SLC17A2	rs34525648	7.07E-05	1.12E-03	0.01
VARS2	rs3131787	9.41E-05	1.45E-03	0.01
EMX1	rs2077586	9.48E-05	1.45E-03	0.04
FXR1	rs1805589	1.07E-04	1.62E-03	-0.02
SYNGAP1	rs9394145	1.08E-04	1.63E-03	0.01
AGPAT1	rs3130281	1.34E-04	1.97E-03	0.01
SMG6	rs2224770	1.35E-04	1.98E-03	0.00
HIST1H3C	rs28360595	1.82E-04	2.59E-03	0.03
ZSCAN31	rs9468350	1.86E-04	2.64E-03	0.01
CNTN4	rs9845120	2.07E-04	2.90E-03	0.01
HIST1H2AC	rs198811	2.22E-04	3.09E-03	-0.03
LRRC48	rs4584886	2.28E-04	3.16E-03	0.00
KCTD13	rs4424923	2.31E-04	3.20E-03	0.01
ALPK3	rs17601029	2.60E-04	3.54E-03	-0.02
FOXP1	rs6803008	2.62E-04	3.57E-03	0.01
MOG	rs416571	2.87E-04	3.86E-03	-0.01
PLA2G15	rs1971546	3.45E-04	4.53E-03	-0.01
HIST1H1B	rs200949	3.46E-04	4.55E-03	0.00
AGER	rs204993	3.80E-04	4.93E-03	0.01
PRR12	rs57940349	3.91E-04	5.05E-03	0.02
HIST1H4K	rs2747054	4.06E-04	5.22E-03	0.01
HSPA1A	rs2075800	4.25E-04	5.43E-03	-0.02
HIST1H1A	rs28360595	4.57E-04	5.79E-03	-0.02

Gene	SNP	P_value	FDR	beta
MIR29C	rs7537216	5.16E-04	6.44E-03	-0.01
ZNF592	rs17600551	5.28E-04	6.56E-03	-0.01
ZSCAN23	rs16894108	5.42E-04	6.71E-03	-0.01
SRPK2	rs3801281	6.10E-04	7.43E-03	-0.01
PPP1R18	rs9262142	6.14E-04	7.48E-03	-0.01
CILP2	rs1054930	6.29E-04	7.64E-03	0.01
HSPA9	rs256013	6.48E-04	7.83E-03	0.00
BTN3A1	rs34273322	6.51E-04	7.86E-03	-0.01
BNIP3L	rs3808581	6.53E-04	7.88E-03	0.01
NAA10	rs4898465	7.15E-04	8.53E-03	-0.01
PPP4C	rs34855001	7.62E-04	9.00E-03	0.01
PLCL1	rs1464211	8.37E-04	9.76E-03	-0.01

2、The most significant eQTL analyses results.

(1) The most significant SMR analyses results in whole blood from GTEx.

Gene	topSNP	p_eQTL	p_SMR	p_HEIDI	adjp_SMR
C4B	rs3135394	5.27E-19	3.69E-12	0.08	2.47E-09
TRIM27	rs3131067	9.47E-25	4.17E-11	0.06	1.40E-08
ZFP57	rs885945	9.09E-46	1.88E-10	NA	1.21E-07
LINC00243	rs1049633	2.33E-12	2.92E-09	0.08	6.53E-07
CYP21A1P	rs1265947	1.11E-11	4.48E-09	0.07	6.96E-07
GPN3	rs11065647	2.79E-43	5.19E-09	0.61	6.96E-07
GATAD2A	rs4808203	1.40E-20	3.44E-08	0.21	3.84E-06
C4A	rs2854277	3.45E-14	4.40E-08	0.24	9.47E-06
RP11-73M18.8	rs11846404	3.87E-15	1.09E-07	0.10	1.04E-05
CYP21A2	rs2854277	3.73E-10	1.50E-07	0.63	1.26E-05
MAPK3	rs4424923	2.15E-20	2.44E-07	NA	1.82E-05
SNX19	rs62621284	4.59E-89	1.18E-07	NA	1.90E-05
KLC1	rs11846404	7.58E-13	3.73E-07	0.25	2.50E-05
TRIM38	rs34525648	8.91E-09	8.07E-07	0.10	4.92E-05
PRRC2A	rs2857694	4.83E-11	9.47E-07	0.51	5.29E-05
CCHCR1	rs2853999	2.19E-09	7.11E-07	0.71	5.39E-05
RPL23AP1	rs885945	6.30E-12	7.50E-07	NA	5.39E-05
RP11-890B15.3	rs62621284	7.13E-23	1.61E-06	NA	8.01E-05
HLA-S	rs1131165	5.27E-15	2.27E-06	0.12	1.05E-04
PSMA4	rs905739	2.33E-10	4.28E-06	NA	1.61E-04
TMTC1	rs302317	2.92E-18	4.73E-06	NA	1.61E-04
AC013264.2	rs10931779	9.61E-11	4.31E-06	0.11	1.92E-04
C2orf47	rs1658810	2.46E-08	4.10E-06	NA	1.92E-04
C2	rs9269102	3.32E-07	5.43E-06	0.69	2.18E-04
FAM216A	rs11065647	1.52E-10	5.53E-06	0.87	2.18E-04
PLCL1	rs3731570	2.60E-14	7.26E-06	0.07	2.34E-04
FLOT1	rs2394978	7.89E-07	6.52E-06	0.37	2.36E-04
FTCDNL1	rs281771	6.58E-08	7.35E-06	NA	2.36E-04
LY6G5B	rs805303	4.96E-08	6.75E-06	NA	2.36E-04
PBX2	rs204990	5.83E-07	7.40E-06	0.34	2.36E-04
HLA-G	rs3129984	1.38E-06	9.70E-06	0.42	2.83E-04
FAM109B	rs1807494	1.01E-10	1.07E-05	NA	2.86E-04
MICB	rs9266209	4.47E-08	1.07E-05	0.31	2.86E-04
CYP2D8P1	rs134889	2.73E-09	1.20E-05	0.24	3.09E-04
NT5C2	rs77420391	3.45E-07	1.30E-05	0.69	3.22E-04
HCG4P3	rs885945	2.74E-08	1.17E-05	NA	3.44E-04
TBX6	rs4424923	1.88E-09	1.55E-05	NA	3.59E-04
POU5F1	rs6457375	5.85E-08	1.64E-05	0.12	3.66E-04
SETD8	rs10772999	2.24E-07	1.78E-05	0.46	3.86E-04

Gene	topSNP	p_eQTL	p_SMR	p_HEIDI	adjp_SMR
AKAP10	rs72843506	1.07E-18	1.66E-05	NA	4.30E-04
DUS2	rs56303487	1.18E-20	2.11E-05	NA	4.42E-04
ALMS1	rs11903916	7.73E-11	2.20E-05	NA	4.46E-04
YPEL3	rs4788198	4.64E-08	2.30E-05	NA	4.53E-04
SREBF1	rs9895335	2.06E-14	2.41E-05	0.13	4.61E-04
FES	rs35346340	2.54E-09	2.61E-05	NA	4.87E-04
DDX39B	rs113239947	4.06E-09	2.87E-05	0.27	5.19E-04
RP11-325F22.2	rs7807853	3.19E-09	2.94E-05	0.70	5.19E-04
CDK2AP1	rs10846491	5.09E-09	3.04E-05	0.08	5.22E-04
HLA-F-AS1	rs1611296	3.08E-07	3.29E-05	0.23	5.38E-04
TMEM110	rs13072536	2.50E-10	3.27E-05	0.11	5.38E-04
MUSTN1	rs7638524	1.69E-12	2.40E-05	0.06	5.53E-04
MAP3K11	rs1193851	3.35E-10	3.76E-05	NA	5.98E-04
TSSK6	rs62135552	2.13E-08	3.84E-05	0.09	5.98E-04
GABBR1	rs3129055	3.32E-07	4.11E-05	0.11	6.19E-04
PRMT7	rs1971546	4.59E-10	4.25E-05	NA	6.20E-04
CCDC134	rs9607850	6.02E-09	5.36E-05	NA	7.65E-04
PRSS16	rs3757150	1.09E-05	5.60E-05	0.40	7.82E-04
HLA-H	rs885945	2.77E-06	6.56E-05	0.23	8.32E-04
IER3	rs1639108	1.78E-05	6.10E-05	0.43	8.32E-04
MEI1	rs1047997	4.46E-07	6.70E-05	NA	8.32E-04
RP1-265C24.5	rs9295759	2.35E-06	6.54E-05	0.07	8.32E-04
TEF	rs9611488	7.99E-08	6.38E-05	NA	8.32E-04
ZBTB12	rs1131165	1.46E-06	6.65E-05	0.68	8.32E-04
INO80E	rs4788198	1.00E-06	7.82E-05	NA	9.52E-04
FTSJ2	rs1476887	4.73E-06	8.64E-05	0.24	1.03E-03
RP11-499P20.2	rs6482406	3.94E-07	1.01E-04	NA	1.19E-03
ARL6IP4	rs941306	5.32E-07	1.11E-04	0.19	1.26E-03
SFMBT1	rs62253583	1.13E-06	1.11E-04	0.51	1.26E-03
AC103965.1	rs220333	2.72E-07	8.53E-05	0.27	1.32E-03
GMIP	rs880090	1.12E-07	1.20E-04	0.14	1.33E-03
HLA-DMA	rs116131925	2.00E-05	1.23E-04	0.16	1.33E-03
RERE	rs302719	2.77E-07	1.23E-04	NA	1.33E-03
RP1-257I20.14	rs5758645	2.21E-06	1.27E-04	0.58	1.35E-03
FKBPL	rs3130281	3.46E-05	1.30E-04	0.65	1.36E-03
RNASEH2C	rs1193851	1.12E-07	1.41E-04	NA	1.46E-03
CLP1	rs708228	1.54E-06	1.70E-04	NA	1.72E-03
CSNK2B	rs2857694	1.36E-05	1.81E-04	0.59	1.72E-03
DDX39BP2	rs3094724	2.27E-05	1.74E-04	0.21	1.72E-03
HLA-T	rs3129012	4.48E-05	1.81E-04	0.88	1.72E-03
HLA-U	rs1063320	3.24E-07	1.77E-04	0.06	1.72E-03
GOLGA6L5	rs12905223	1.27E-06	1.34E-04	0.34	1.77E-03
ALMS1P	rs11903916	5.99E-07	1.95E-04	NA	1.79E-03

Gene	topSNP	p_eQTL	p_SMR	p_HEIDI	adjp_SMR
TRIM10	rs1059535	2.87E-05	1.93E-04	0.27	1.79E-03
IGSF9B	rs73036062	1.37E-05	1.98E-04	NA	1.79E-03
BAG6	rs2857694	1.65E-05	2.01E-04	0.88	1.80E-03
SKIV2L	rs389883	1.05E-05	2.06E-04	NA	1.81E-03
BAG5	rs4906336	1.60E-05	2.24E-04	NA	1.95E-03
TDRD9	rs10132641	8.33E-06	2.29E-04	NA	1.97E-03
ABCB9	rs7296418	7.94E-06	2.40E-04	0.16	2.02E-03
ZNF391	rs7750526	6.61E-05	2.41E-04	0.69	2.02E-03
HIST1H4H	rs61534839	1.04E-06	2.58E-04	NA	2.10E-03
IFITM4P	rs416571	4.02E-06	2.61E-04	NA	2.10E-03
MAU2	rs62135552	4.83E-06	2.60E-04	NA	2.10E-03
XXbac-BPG181B23.7	rs9266244	7.35E-07	1.84E-04	0.43	2.12E-03
NT5DC2	rs13076193	2.20E-09	1.90E-04	0.06	2.15E-03
RP11-245J9.4	rs7615475	5.85E-06	2.85E-04	0.27	2.25E-03
ZSCAN9	rs9885928	1.88E-05	2.98E-04	0.22	2.32E-03
CLIC1	rs3115674	1.40E-04	3.09E-04	0.58	2.35E-03
LCAT	rs7204208	6.22E-09	3.06E-04	NA	2.35E-03
STK19	rs74618856	3.45E-05	3.18E-04	0.80	2.40E-03
GUSBP2	rs9348746	2.80E-05	3.26E-04	NA	2.43E-03
HLA-A	rs416571	5.40E-06	2.56E-04	NA	2.55E-03
GDPD3	rs4424923	1.30E-05	3.57E-04	NA	2.61E-03
MOG	rs3117289	8.66E-05	3.58E-04	NA	2.61E-03
SH3RF1	rs4434205	1.06E-07	3.63E-04	NA	2.62E-03
RP11-282O18.3	rs11057238	1.39E-05	4.20E-04	0.09	2.96E-03
AGER	rs3095240	1.43E-04	4.46E-04	0.82	2.99E-03
NMB	rs1051168	1.40E-05	4.39E-04	0.72	2.99E-03
RP11-350N15.5	rs72630609	1.25E-05	4.32E-04	NA	2.99E-03
TCF19	rs2233960	9.19E-06	4.39E-04	0.36	2.99E-03
ZSCAN2	rs62021208	4.69E-06	4.43E-04	0.82	2.99E-03
HLA-C	rs114475699	4.77E-08	3.57E-04	NA	3.02E-03
DDAH2	rs3130281	1.94E-04	4.79E-04	0.65	3.18E-03
TUBB	rs2523598	1.09E-04	4.87E-04	0.34	3.20E-03
HCG4P5	rs885945	4.29E-05	3.87E-04	NA	3.21E-03
HCG27	rs114475699	8.52E-08	3.99E-04	0.57	3.26E-03
XXbac-BPG299F13.17	rs9266244	7.07E-06	4.16E-04	NA	3.36E-03
AGPAT1	rs204993	5.98E-05	5.18E-04	NA	3.37E-03
HIST1H2AK	rs7739915	2.42E-04	5.33E-04	0.72	3.44E-03
VARS2	rs2853999	1.47E-04	4.85E-04	NA	3.68E-03
BTN2A2	rs2073528	1.66E-04	5.79E-04	NA	3.70E-03
ZSCAN12P1	rs1150666	7.63E-05	5.92E-04	0.48	3.74E-03
PCCB	rs9845788	2.10E-05	6.15E-04	0.35	3.85E-03
PEMT	rs9895335	3.73E-06	6.35E-04	0.90	3.94E-03
MPHOSPH9	rs941306	3.72E-05	6.46E-04	0.36	3.97E-03

Gene	topSNP	p_eQTL	p_SMR	p_HEIDI	adjp_SMR
RP5-821D11.7	rs9607850	1.87E-05	6.65E-04	NA	4.05E-03
LY6G6D	rs805303	1.74E-04	6.83E-04	NA	4.12E-03
RP11-285J16.1	rs73068054	2.81E-05	6.05E-04	NA	4.22E-03
HCG18	rs6914215	1.37E-04	7.13E-04	0.78	4.27E-03
HLA-DOB	rs6906021	9.26E-05	7.58E-04	NA	4.45E-03
ATP6V1G2	rs2736176	1.60E-04	7.66E-04	NA	4.46E-03
BAG4	rs9198	4.97E-05	7.96E-04	0.90	4.59E-03
C2orf69	rs1658810	2.37E-04	8.01E-04	NA	4.59E-03
RP11-10A14.5	rs2948293	1.37E-05	7.10E-04	0.33	4.73E-03
ZKSCAN4	rs3131095	5.78E-04	8.49E-04	0.84	4.82E-03
HLA-K	rs886424	4.82E-04	8.86E-04	NA	4.99E-03
SDAD1P1	rs3808573	3.06E-05	9.01E-04	NA	5.03E-03
APOPT1	rs58033365	1.89E-04	9.62E-04	NA	5.18E-03
COL11A2	rs9268084	4.08E-04	9.62E-04	NA	5.18E-03
DESI1	rs9607850	4.37E-05	9.53E-04	NA	5.18E-03
MAD1L1	rs6975354	7.08E-05	9.66E-04	0.21	5.18E-03
SLC44A4	rs622871	2.62E-04	9.41E-04	NA	5.18E-03
RP11-73M18.7	rs11846404	2.16E-04	9.99E-04	NA	5.27E-03
RPS12P26	rs35346340	7.65E-05	9.98E-04	NA	5.27E-03
ITPR3	rs10947436	1.12E-04	1.03E-03	NA	5.37E-03
U91328.19	rs13204572	6.60E-04	1.05E-03	NA	5.44E-03
CALHM2	rs10430665	3.86E-04	1.06E-03	0.69	5.44E-03
HLA-DPB2	rs3134940	5.23E-04	1.09E-03	0.73	5.56E-03
MRPS21	rs55963510	5.22E-05	1.11E-03	NA	5.61E-03
HIST1H2BD	rs9393698	9.80E-05	1.15E-03	NA	5.76E-03
ZNF747	rs4788198	2.00E-04	1.15E-03	NA	5.76E-03
MICA	rs9266209	3.10E-04	1.18E-03	NA	5.86E-03
SLC12A4	rs7204208	5.46E-06	1.21E-03	NA	5.96E-03
TOM1L2	rs9895335	2.68E-05	1.23E-03	0.52	5.99E-03
HSPA1B	rs389883	2.58E-04	1.25E-03	NA	6.06E-03
ABHD16A	rs3130621	7.17E-04	1.32E-03	NA	6.25E-03
HIST1H4E	rs9393698	1.26E-04	1.31E-03	NA	6.25E-03
OR2H2	rs3094064	7.44E-04	1.31E-03	NA	6.25E-03
ETF1	rs11745933	8.24E-05	1.35E-03	NA	6.33E-03
HLA-DPB1	rs114132738	1.06E-04	1.35E-03	NA	6.33E-03
ATXN7	rs7627690	1.59E-04	1.48E-03	0.43	6.82E-03
NDUFA6	rs5758645	2.70E-04	1.47E-03	NA	6.82E-03
HIST1H2BF	rs9393698	1.69E-04	1.52E-03	NA	6.88E-03
HIST1H4K	rs35501037	1.09E-03	1.52E-03	NA	6.88E-03
PPT2	rs2280774	1.27E-04	1.52E-03	NA	6.88E-03
AIF1	rs2857694	4.63E-04	1.57E-03	NA	7.06E-03
ZDHHC5	rs682503	1.64E-04	1.59E-03	NA	7.10E-03
HLA-F	rs1610589	4.87E-04	1.65E-03	0.95	7.14E-03

Gene	topSNP	p_eQTL	p_SMR	p_HEIDI	adjp_SMR
NEK1	rs4434205	3.14E-05	1.65E-03	NA	7.14E-03
PRR12	rs10417980	1.25E-04	1.62E-03	NA	7.14E-03
RNF39	rs3094724	6.37E-04	1.65E-03	NA	7.14E-03
RNF5	rs6914215	4.97E-04	1.65E-03	NA	7.14E-03
TRANK1	rs73068054	1.68E-04	1.71E-03	NA	7.33E-03
GNL3	rs1029871	8.18E-05	1.78E-03	NA	7.60E-03
NPIPB11	rs4424923	3.25E-04	1.86E-03	NA	7.88E-03
LST1	rs1131165	5.48E-04	1.88E-03	0.88	7.88E-03
SPCS1	rs34017441	7.77E-05	1.88E-03	NA	7.88E-03
GNA12	rs10263703	4.63E-04	2.07E-03	NA	8.63E-03
HCG20	rs1610696	9.16E-04	2.17E-03	NA	8.80E-03
RP11-73M18.9	rs3212090	7.04E-04	2.16E-03	NA	8.80E-03
VWA7	rs805303	8.77E-04	2.13E-03	NA	8.80E-03
Z83851.3	rs5758605	5.03E-04	2.19E-03	NA	8.83E-03
ZBTB22	rs10947436	4.52E-04	2.24E-03	NA	8.98E-03
SNORD35A	rs7047	5.06E-04	2.27E-03	NA	9.05E-03
3-Sep	rs7290134	2.78E-04	2.29E-03	NA	9.08E-03
TRPV4	rs3026445	3.28E-04	2.35E-03	NA	9.28E-03
RP5-874C20.3	rs7776351	1.17E-03	2.38E-03	NA	9.34E-03
LY6G5C	rs2075800	5.03E-04	1.88E-03	NA	9.40E-03
HIST1H1T	rs198811	6.32E-04	2.54E-03	NA	9.90E-03
HLA-W	rs3129055	8.36E-04	2.65E-03	NA	1.02E-02
NDUFA13	rs880090	3.60E-04	2.65E-03	NA	1.02E-02
XXbac-BPG252P9.10	rs3094628	1.78E-03	2.68E-03	NA	1.02E-02
ALG1L13P	rs2948293	2.09E-04	2.25E-03	NA	1.03E-02
ZSCAN16-AS1	rs2275508	1.08E-03	2.75E-03	NA	1.05E-02
APH1A	rs11589922	4.93E-04	2.81E-03	NA	1.06E-02
ELMO3	rs6499157	8.89E-05	2.86E-03	NA	1.08E-02
PGBD1	rs200983	2.02E-03	2.87E-03	NA	1.08E-02
RP11-731C17.2	rs696520	8.72E-04	2.92E-03	NA	1.09E-02
HIST1H4I	rs66972160	1.05E-03	2.98E-03	NA	1.10E-02
ITIH3	rs4687663	5.07E-04	3.11E-03	NA	1.15E-02
MDC1	rs1063320	5.04E-04	3.25E-03	NA	1.18E-02
MED19	rs708228	7.63E-04	3.29E-03	NA	1.18E-02
NDUFA6-AS1	rs6002597	7.31E-04	3.28E-03	NA	1.18E-02
ZNF204P	rs9468350	2.15E-03	3.28E-03	NA	1.18E-02
SLC4A1AP	rs4666014	2.06E-04	3.48E-03	NA	1.25E-02
ATP13A1	rs7252981	9.56E-04	3.53E-03	NA	1.25E-02
RP11-347C12.2	rs4424923	9.59E-04	3.54E-03	NA	1.25E-02
CTRL	rs6499157	1.76E-04	3.65E-03	NA	1.29E-02
CTA-989H11.1	rs134889	1.17E-03	3.71E-03	NA	1.30E-02
TCF20	rs2143139	1.19E-03	3.73E-03	NA	1.30E-02
RP3-477M7.5	rs302719	8.07E-04	3.75E-03	NA	1.30E-02

Gene	topSNP	p_eQTL	p_SMR	p_HEIDI	adjp_SMR
PRRT1	rs9273595	2.35E-03	3.78E-03	NA	1.30E-02
SFXN2	rs10883798	2.03E-03	3.90E-03	NA	1.33E-02
ZNF785	rs4424923	1.12E-03	3.89E-03	NA	1.33E-02
SPECC1	rs72843506	2.56E-04	3.31E-03	NA	1.38E-02
RP11-196G18.3	rs11589922	9.61E-04	4.10E-03	NA	1.40E-02
DFNA5	rs79210963	8.33E-04	4.27E-03	NA	1.44E-02
LY6G6C	rs2395174	1.33E-03	4.28E-03	NA	1.44E-02
HLA-J	rs885945	1.47E-03	3.67E-03	NA	1.46E-02
SRR	rs11078018	1.16E-03	4.40E-03	NA	1.47E-02
ABCF1	rs3129055	1.80E-03	4.46E-03	NA	1.49E-02
MNT	rs11078018	1.21E-03	4.52E-03	NA	1.50E-02
LRRFIP2	rs6769400	1.21E-03	4.56E-03	NA	1.51E-02
DGKZ	rs876701	1.90E-03	4.62E-03	NA	1.52E-02
CTA-250D10.23	rs133348	1.09E-03	4.64E-03	NA	1.52E-02
WBP2NL	rs8135801	1.34E-03	4.70E-03	NA	1.53E-02
ATF6B	rs3096690	3.68E-03	4.83E-03	NA	1.55E-02
GTF2H4	rs3094125	3.52E-03	4.81E-03	NA	1.55E-02
RP11-524F11.1	rs6826	6.46E-04	4.91E-03	NA	1.57E-02
CUTA	rs209474	1.08E-03	4.94E-03	NA	1.57E-02
MICD	rs1611350	2.62E-03	5.00E-03	NA	1.57E-02
PAN2	rs61937595	1.96E-03	5.01E-03	NA	1.57E-02
PPP1R18	rs6457375	2.24E-03	5.01E-03	NA	1.57E-02
SF3B1	rs700664	1.51E-03	5.06E-03	NA	1.57E-02
U91328.22	rs13196986	2.45E-03	5.06E-03	NA	1.57E-02
RILPL2	rs883563	1.46E-03	5.17E-03	NA	1.59E-02
ZNF184	rs13215020	3.47E-03	5.16E-03	NA	1.59E-02
CFB	rs9269102	3.62E-03	5.21E-03	NA	1.59E-02
C7orf50	rs7783715	1.25E-03	5.25E-03	NA	1.60E-02
LGALS9B	rs72843506	5.88E-04	4.80E-03	NA	1.70E-02
MIEF2	rs7219320	6.06E-04	4.83E-03	NA	1.70E-02
OR2B6	rs7739915	4.09E-03	5.63E-03	NA	1.71E-02
HIST1H1E	rs3757150	4.00E-03	5.66E-03	NA	1.71E-02
C12orf65	rs11057238	1.88E-03	5.70E-03	NA	1.71E-02
RCN3	rs7047	2.22E-03	5.81E-03	NA	1.74E-02
HLA-DRA	rs9271562	2.79E-03	5.85E-03	NA	1.74E-02
DPEP3	rs7204208	5.89E-04	5.87E-03	NA	1.74E-02
USP32P3	rs72843506	6.48E-04	5.03E-03	NA	1.74E-02
HLA-V	rs1610622	1.55E-03	6.01E-03	NA	1.77E-02
NEU1	rs6908927	3.79E-03	6.02E-03	NA	1.77E-02
XXbac-BPG299F13.14	rs389883	1.21E-03	5.51E-03	NA	1.83E-02
ACAD8	rs73034295	3.28E-03	6.27E-03	NA	1.83E-02
RP11-62H7.2	rs1878561	1.20E-03	5.76E-03	NA	1.88E-02
C6orf25	rs4249311	3.45E-03	6.57E-03	NA	1.91E-02

Gene	topSNP	p_eQTL	p_SMR	p_HEIDI	adjp_SMR
WBP1L	rs284854	1.84E-03	6.12E-03	NA	1.91E-02
C11orf31	rs682503	2.12E-03	6.66E-03	NA	1.91E-02
TRIM26	rs3129012	4.58E-03	6.63E-03	NA	1.91E-02
HCG9	rs1610720	3.77E-03	6.75E-03	NA	1.93E-02
ABT1	rs9348746	3.37E-03	6.85E-03	NA	1.94E-02
IRF3	rs57940349	1.90E-03	6.79E-03	NA	1.94E-02
RP5-1115A15.1	rs301792	1.93E-03	6.84E-03	NA	1.94E-02
SIPA1	rs1193851	1.92E-03	6.99E-03	NA	1.97E-02
NAGA	rs5758605	3.06E-03	7.09E-03	NA	1.99E-02
FCGR1C	rs72694955	2.25E-03	7.13E-03	NA	1.99E-02
NCR3	rs9269102	5.25E-03	7.17E-03	NA	1.99E-02
CHST12	rs7783715	2.20E-03	7.32E-03	NA	2.01E-02
NIF3L1	rs1658810	4.53E-03	7.33E-03	NA	2.01E-02
TMEM161A	rs2965199	3.43E-03	7.30E-03	NA	2.01E-02
NHP2L1	rs5758605	3.32E-03	7.51E-03	NA	2.05E-02
ZNF592	rs3803405	1.61E-03	7.61E-03	NA	2.07E-02
KAT5	rs1193851	2.29E-03	7.75E-03	NA	2.10E-02
LTA	rs2905737	2.35E-03	7.79E-03	NA	2.11E-02
ANP32E	rs72694955	2.63E-03	7.86E-03	NA	2.11E-02
EIF2B1	rs11057238	3.13E-03	7.96E-03	NA	2.12E-02
HIST1H2BH	rs61534839	2.49E-03	8.02E-03	NA	2.13E-02
DPEP2	rs6499157	1.22E-03	8.16E-03	NA	2.15E-02
TMEM180	rs11818043	3.77E-03	8.35E-03	NA	2.19E-02
HIST1H2AL	rs34130214	6.46E-03	8.45E-03	NA	2.20E-02
PPM1M	rs2276834	2.86E-03	8.44E-03	NA	2.20E-02
RP11-261C10.3	rs12751210	2.75E-03	8.60E-03	NA	2.23E-02
RPL32P1	rs9273595	6.25E-03	8.60E-03	NA	2.23E-02
B3GALT4	rs707962	5.38E-03	8.75E-03	NA	2.23E-02
NRM	rs1610622	2.90E-03	8.74E-03	NA	2.23E-02
PSMD6-AS2	rs13272	3.55E-03	8.74E-03	NA	2.23E-02
TRIM39	rs12665531	2.94E-03	8.75E-03	NA	2.23E-02
EGFL8	rs2523554	4.63E-03	8.84E-03	NA	2.24E-02
HIST1H2BN	rs4711163	5.83E-03	8.85E-03	NA	2.24E-02
RAD9B	rs3026445	3.25E-03	8.89E-03	NA	2.24E-02
PPP1R2P1	rs616187	4.31E-03	8.96E-03	NA	2.25E-02
MEF2C-AS1	rs62378245	4.62E-03	9.06E-03	NA	2.26E-02
VAMP3	rs11121178	3.13E-03	9.03E-03	NA	2.26E-02
FOXP1	rs9842406	3.35E-03	9.18E-03	NA	2.28E-02
BATF2	rs1193851	3.06E-03	9.23E-03	NA	2.28E-02
SYNGAP1	rs9276915	3.68E-03	9.28E-03	NA	2.29E-02
ATPAF2	rs4584886	2.32E-03	9.48E-03	NA	2.33E-02
MSH5	rs2858310	4.20E-03	9.65E-03	NA	2.36E-02
ARPC3	rs3026445	3.79E-03	9.80E-03	NA	2.38E-02

Gene	topSNP	p_eQTL	p_SMR	p_HEIDI	adjp_SMR
DDHD2	rs2016875	4.10E-03	9.86E-03	NA	2.38E-02
TM2D2	rs9198	4.17E-03	9.81E-03	NA	2.38E-02
TULP3	rs68089646	3.79E-03	9.85E-03	NA	2.38E-02
OGFOD2	rs4148863	5.31E-03	9.91E-03	NA	2.38E-02
SPATS2L	rs3769456	4.82E-03	9.94E-03	NA	2.38E-02
NELFE	rs204990	7.72E-03	9.97E-03	NA	2.38E-02
RP11-677M14.3	rs12293624	4.06E-03	1.00E-02	NA	2.38E-02
RFXANK	rs880090	3.74E-03	1.01E-02	NA	2.39E-02
XXbac-BPG249D20.9	rs28744244	4.07E-03	1.02E-02	NA	2.40E-02
PAQR5	rs28620094	3.84E-03	1.03E-02	NA	2.42E-02
ZSCAN16	rs34130214	8.14E-03	1.04E-02	NA	2.42E-02
WBP1	rs11903916	3.98E-03	1.04E-02	NA	2.43E-02
RP3-370M22.8	rs11705236	5.05E-03	1.07E-02	NA	2.47E-02
HLA-DPA1	rs9270665	6.26E-03	1.08E-02	NA	2.47E-02
TAPSAR1	rs34434863	4.88E-03	1.08E-02	NA	2.47E-02
ZNRD1	rs2106072	8.74E-03	1.07E-02	NA	2.47E-02
HIST1H3E	rs2073526	5.00E-03	1.10E-02	NA	2.50E-02
NFKBIL1	rs12665531	4.22E-03	1.10E-02	NA	2.50E-02
PSMB8	rs4947350	6.74E-03	1.11E-02	NA	2.51E-02
RP11-571M6.8	rs324017	5.62E-03	1.11E-02	NA	2.51E-02
RP4-756G23.5	rs5758645	5.89E-03	1.12E-02	NA	2.51E-02
ATF4	rs5757717	5.69E-03	1.12E-02	NA	2.51E-02
RP11-425L10.1	rs7130141	6.53E-03	1.12E-02	NA	2.51E-02
DEPDC1B	rs4604142	7.24E-03	1.14E-02	NA	2.54E-02
NR1H2	rs7047	5.99E-03	1.16E-02	NA	2.57E-02
CNGB1	rs12447860	5.95E-03	1.17E-02	NA	2.58E-02
PRR3	rs3129986	9.22E-03	1.17E-02	NA	2.58E-02
TMX2	rs708228	5.61E-03	1.18E-02	NA	2.59E-02
INHBE	rs61937595	6.70E-03	1.21E-02	NA	2.63E-02
SDCCAG8	rs12751210	4.78E-03	1.21E-02	NA	2.64E-02
RPS18	rs176249	5.14E-03	1.22E-02	NA	2.65E-02
SNRNP35	rs1790119	5.66E-03	1.23E-02	NA	2.66E-02
MICE	rs1633081	7.44E-03	1.24E-02	NA	2.67E-02
RFT1	rs7638524	4.88E-03	1.24E-02	NA	2.67E-02
ERCC8	rs4604142	8.08E-03	1.25E-02	NA	2.67E-02
ALDH16A1	rs7047	6.72E-03	1.26E-02	NA	2.68E-02
C6orf48	rs1046089	9.00E-03	1.28E-02	NA	2.68E-02
CTSW	rs1193851	5.15E-03	1.28E-02	NA	2.68E-02
RGL2	rs9271562	7.88E-03	1.28E-02	NA	2.68E-02
RP11-894P9.1	rs1799796	7.95E-03	1.27E-02	NA	2.68E-02
SERPING1	rs708228	6.24E-03	1.27E-02	NA	2.68E-02
WDR81	rs8076939	5.20E-03	1.27E-02	NA	2.68E-02
RP4-778K6.3	rs6968335	5.69E-03	1.30E-02	NA	2.71E-02

Gene	topSNP	p_eQTL	p_SMR	p_HEIDI	adjp_SMR
SGK223	rs1878561	3.10E-03	9.86E-03	NA	2.71E-02
SLURP1	rs72687376	5.91E-03	1.31E-02	NA	2.73E-02
C16orf93	rs4788198	7.69E-03	1.33E-02	NA	2.73E-02
C1orf116	rs12749612	4.24E-03	1.32E-02	NA	2.73E-02
HIST1H4J	rs4711163	9.51E-03	1.33E-02	NA	2.73E-02
TAPBP	rs511515	5.73E-03	1.33E-02	NA	2.73E-02
HCG4P11	rs1610622	5.75E-03	1.35E-02	NA	2.74E-02
MED25	rs7047	7.32E-03	1.34E-02	NA	2.74E-02
PHF1	rs4947350	8.62E-03	1.35E-02	NA	2.74E-02
TNFRSF13C	rs133348	6.00E-03	1.34E-02	NA	2.74E-02
FAM83G	rs11078410	4.02E-03	1.38E-02	NA	2.80E-02
SLC47A1	rs72843506	2.91E-03	1.08E-02	NA	2.83E-02
HIST1H2AC	rs1179087	7.32E-03	1.41E-02	NA	2.85E-02
HLA-B	rs2233960	2.89E-03	1.13E-02	NA	2.88E-02
TAP2	rs1770	6.71E-03	1.44E-02	NA	2.90E-02
ATG13	rs12574918	7.48E-03	1.47E-02	NA	2.92E-02
CDH1	rs7187202	6.16E-03	1.46E-02	NA	2.92E-02
CTSK	rs7544145	6.72E-03	1.47E-02	NA	2.92E-02
RP1-34B20.4	rs9393698	7.35E-03	1.47E-02	NA	2.92E-02
FGFR1	rs17435276	5.25E-03	1.48E-02	NA	2.93E-02
LYPLA2P1	rs756139	7.70E-03	1.49E-02	NA	2.95E-02
HSPA1A	rs616187	8.75E-03	1.50E-02	NA	2.96E-02
HIST2H2AC	rs72694965	7.99E-03	1.51E-02	NA	2.97E-02
DOPEY1	rs4470825	7.78E-03	1.57E-02	NA	3.07E-02
GPANK1	rs2233974	1.21E-02	1.57E-02	NA	3.07E-02
SAPCD1	rs3095240	1.23E-02	1.57E-02	NA	3.07E-02
ZNF165	rs1150666	1.01E-02	1.59E-02	NA	3.08E-02
BTN3A3	rs61534839	7.54E-03	1.62E-02	NA	3.09E-02
EHMT2	rs1131156	9.71E-03	1.61E-02	NA	3.09E-02
GOLPH3L	rs7544145	7.76E-03	1.62E-02	NA	3.09E-02
HCG4P7	rs9257834	7.63E-03	1.63E-02	NA	3.09E-02
HIST1H2AH	rs34130214	1.36E-02	1.64E-02	NA	3.09E-02
HIST1H4C	rs3757150	1.34E-02	1.64E-02	NA	3.09E-02
SNX32	rs1193851	7.54E-03	1.64E-02	NA	3.09E-02
TRIM8	rs11191356	8.61E-03	1.61E-02	NA	3.09E-02
UBE3B	rs3026445	8.10E-03	1.63E-02	NA	3.09E-02
XXbac-BPG252P9.9	rs1632855	1.32E-02	1.61E-02	NA	3.09E-02
NAB2	rs324017	9.64E-03	1.65E-02	NA	3.10E-02
MARCKSL1P1	rs34747231	1.26E-02	1.66E-02	NA	3.12E-02
DAXX	rs210133	1.03E-02	1.68E-02	NA	3.14E-02
LEMD2	rs116131925	1.30E-02	1.68E-02	NA	3.14E-02
HIST1H1B	rs7750526	1.37E-02	1.71E-02	NA	3.16E-02
NEAT1	rs1193851	8.03E-03	1.71E-02	NA	3.16E-02

Gene	topSNP	p_eQTL	p_SMR	p_HEIDI	adjp_SMR
RP11-434P11.2	rs11903916	8.45E-03	1.72E-02	NA	3.16E-02
ZNF192P1	rs9301	1.22E-02	1.72E-02	NA	3.16E-02
RP4-669P10.16	rs7290134	8.52E-03	1.72E-02	NA	3.17E-02
SLC39A7	rs9274558	1.04E-02	1.73E-02	NA	3.17E-02
SNX7	rs4970719	1.06E-02	1.75E-02	NA	3.19E-02
LTB	rs1003878	1.32E-02	1.78E-02	NA	3.24E-02
ELL	rs2905432	1.16E-02	1.79E-02	NA	3.24E-02
DRG2	rs9895335	7.43E-03	1.79E-02	NA	3.25E-02
PCNXL3	rs1193851	8.68E-03	1.80E-02	NA	3.25E-02
C10orf32	rs284856	8.32E-03	1.82E-02	NA	3.28E-02
ITGAL	rs4788198	1.16E-02	1.82E-02	NA	3.28E-02
FBN2	rs2764766	5.44E-03	1.88E-02	NA	3.37E-02
RP11-455F5.4	rs4788198	1.25E-02	1.92E-02	NA	3.41E-02
SUGP2	rs1054930	1.24E-02	1.92E-02	NA	3.41E-02
SLC43A2	rs9909895	1.09E-02	1.93E-02	NA	3.43E-02
RPL3P2	rs2523673	1.29E-02	1.94E-02	NA	3.43E-02
TLE3	rs2137272	7.60E-03	1.95E-02	NA	3.44E-02
APOM	rs3094014	1.61E-02	1.98E-02	NA	3.45E-02
BTN1A1	rs6456728	1.66E-02	1.99E-02	NA	3.45E-02
CS	rs324015	1.24E-02	1.98E-02	NA	3.45E-02
IBTK	rs4470825	1.08E-02	1.98E-02	NA	3.45E-02
PMM1	rs7290134	1.05E-02	1.99E-02	NA	3.45E-02
SPDYA	rs13001060	8.39E-03	1.98E-02	NA	3.45E-02
ZNF101	rs880090	1.05E-02	2.00E-02	NA	3.46E-02
PHF5A	rs5996116	1.17E-02	2.01E-02	NA	3.46E-02
RANP1	rs1737048	1.50E-02	2.01E-02	NA	3.46E-02
1-Sep	rs4788198	1.32E-02	2.01E-02	NA	3.46E-02
RP11-78O7.2	rs72843506	6.40E-03	1.70E-02	NA	3.49E-02
AC104695.3	rs4632296	8.77E-03	2.04E-02	NA	3.50E-02
ACD	rs56303487	8.12E-03	2.06E-02	NA	3.50E-02
RP11-261C10.7	rs12751210	1.06E-02	2.06E-02	NA	3.50E-02
RPRD2	rs7521783	1.21E-02	2.05E-02	NA	3.50E-02
SLC39A8	rs13107325	1.59E-02	2.07E-02	NA	3.50E-02
ZSCAN31	rs2747054	1.83E-02	2.08E-02	NA	3.51E-02
CTC-559E9.5	rs62135552	1.26E-02	2.10E-02	NA	3.54E-02
CLK1	rs281771	1.61E-02	2.12E-02	NA	3.57E-02
DXO	rs9276905	1.13E-02	2.13E-02	NA	3.58E-02
RP1-86C11.7	rs16897515	1.90E-02	2.14E-02	NA	3.59E-02
CYP17A1	rs10883823	1.62E-02	2.17E-02	NA	3.61E-02
TVP23B	rs11078400	9.26E-03	2.17E-02	NA	3.61E-02
VARS	rs7195	1.47E-02	2.17E-02	NA	3.61E-02
RP11-455F5.3	rs4788198	1.46E-02	2.18E-02	NA	3.61E-02
ZNF14	rs7249692	1.36E-02	2.18E-02	NA	3.61E-02

Gene	topSNP	p_eQTL	p_SMR	p_HEIDI	adjp_SMR
MADD	rs61882682	1.53E-02	2.21E-02	NA	3.65E-02
ALPK3	rs7237	9.57E-03	2.24E-02	NA	3.65E-02
ATAT1	rs2523673	1.56E-02	2.26E-02	NA	3.65E-02
EIF3B	rs6975354	1.35E-02	2.26E-02	NA	3.65E-02
GLS2	rs61937595	1.49E-02	2.23E-02	NA	3.65E-02
HARBI1	rs35324223	1.60E-02	2.25E-02	NA	3.65E-02
HCG4B	rs1610721	1.70E-02	2.29E-02	NA	3.65E-02
HIST1H2AG	rs45527431	2.06E-02	2.27E-02	NA	3.65E-02
HSPA1L	rs616187	1.50E-02	2.28E-02	NA	3.65E-02
NUDT3	rs9394145	1.26E-02	2.25E-02	NA	3.65E-02
PITPNA	rs9893573	1.21E-02	2.24E-02	NA	3.65E-02
RP3-425P12.4	rs198811	1.49E-02	2.28E-02	NA	3.65E-02
TOP3A	rs4584886	1.03E-02	2.28E-02	NA	3.65E-02
ZDHHC20P1	rs3129055	1.56E-02	2.26E-02	NA	3.65E-02
ALAS1	rs2581819	1.59E-02	2.31E-02	NA	3.67E-02
GDPGP1	rs6227	1.42E-02	2.30E-02	NA	3.67E-02
TNF	rs1800629	1.85E-02	2.32E-02	NA	3.69E-02
C1orf132	rs2745977	1.11E-02	2.33E-02	NA	3.69E-02
GPR146	rs12671113	1.61E-02	2.34E-02	NA	3.69E-02
RPS3P2	rs208818	1.55E-02	2.35E-02	NA	3.69E-02
SLC39A5	rs324015	1.55E-02	2.35E-02	NA	3.69E-02
XXbac-BPG157A10.21	rs3129877	1.51E-02	2.35E-02	NA	3.69E-02
HIST1H2BL	rs200983	2.10E-02	2.36E-02	NA	3.70E-02
Y_RNA	rs2428498	1.47E-02	2.41E-02	NA	3.76E-02
RTN4RL1	rs9893573	1.38E-02	2.45E-02	NA	3.82E-02
CTB-152G17.6	rs6968335	1.44E-02	2.47E-02	NA	3.83E-02
RP11-73M18.6	rs58033365	1.78E-02	2.47E-02	NA	3.83E-02
GYS1	rs10417980	1.40E-02	2.48E-02	NA	3.84E-02
CETN3	rs6891794	1.75E-02	2.50E-02	NA	3.84E-02
FLCN	rs6826	1.22E-02	2.52E-02	NA	3.84E-02
NFAM1	rs1807494	1.58E-02	2.51E-02	NA	3.84E-02
SBP1	rs2858310	1.58E-02	2.49E-02	NA	3.84E-02
TMEM243	rs73206909	1.58E-02	2.51E-02	NA	3.84E-02
TNXB	rs2858328	1.65E-02	2.52E-02	NA	3.84E-02
CTSS	rs7521783	1.61E-02	2.54E-02	NA	3.87E-02
SPOCK1	rs256013	1.48E-02	2.55E-02	NA	3.87E-02
ASPDH	rs57940349	1.46E-02	2.56E-02	NA	3.87E-02
ADIPOR2	rs68089646	1.55E-02	2.59E-02	NA	3.88E-02
BAP1	rs11235	1.62E-02	2.57E-02	NA	3.88E-02
CDC25C	rs256013	1.52E-02	2.59E-02	NA	3.88E-02
KCTD9	rs1042992	1.61E-02	2.59E-02	NA	3.88E-02
TIMM10	rs708228	1.66E-02	2.60E-02	NA	3.88E-02
HIST1H2BJ	rs198811	1.78E-02	2.61E-02	NA	3.89E-02

Gene	topSNP	p_eQTL	p_SMR	p_HEIDI	adjp_SMR
HIST1H3J	rs1225715	1.97E-02	2.61E-02	NA	3.89E-02
DIS3L2	rs974321	2.10E-02	2.66E-02	NA	3.94E-02
LY6G6E	rs6457375	1.95E-02	2.65E-02	NA	3.94E-02
STAB1	rs352139	9.81E-03	2.66E-02	NA	3.94E-02
IDH2	rs4702	1.95E-02	2.68E-02	NA	3.94E-02
NGRN	rs35346340	1.70E-02	2.68E-02	NA	3.94E-02
RING1	rs511515	1.58E-02	2.68E-02	NA	3.94E-02
C1orf51	rs72694955	1.64E-02	2.70E-02	NA	3.95E-02
DBP	rs10417980	1.59E-02	2.71E-02	NA	3.95E-02
HIST1H3B	rs9393716	2.10E-02	2.72E-02	NA	3.95E-02
NPTXR	rs1883123	1.87E-02	2.71E-02	NA	3.95E-02
SUMO2P1	rs9885928	1.95E-02	2.73E-02	NA	3.95E-02
TAP1	rs9276905	1.59E-02	2.72E-02	NA	3.95E-02
UBE2Q2P1	rs11633450	1.59E-02	2.71E-02	NA	3.95E-02
ZFYVE21	rs4906336	2.03E-02	2.74E-02	NA	3.96E-02
HIST1H1PS1	rs198811	1.89E-02	2.75E-02	NA	3.96E-02
EIF4EBP1	rs16887340	1.78E-02	2.76E-02	NA	3.97E-02
RP11-209D14.4	rs72843506	1.09E-02	2.36E-02	NA	3.98E-02
FAM53C	rs1811371	1.64E-02	2.79E-02	NA	3.99E-02
HCG11	rs2073526	1.81E-02	2.79E-02	NA	3.99E-02
SEMA6C	rs12124898	1.71E-02	2.80E-02	NA	4.00E-02
SREBF2	rs5751220	1.72E-02	2.82E-02	NA	4.03E-02
GBF1	rs284856	1.62E-02	2.86E-02	NA	4.08E-02
OBFC1	rs4917994	2.29E-02	2.93E-02	NA	4.16E-02
ABHD14B	rs9836499	1.99E-02	2.94E-02	NA	4.16E-02
BRD2	rs9275109	1.89E-02	2.96E-02	NA	4.18E-02
HLA-DMB	rs1003878	2.39E-02	2.96E-02	NA	4.18E-02
ADAP1	rs7783715	1.82E-02	2.97E-02	NA	4.18E-02
HIST1H3G	rs501220	2.46E-02	2.98E-02	NA	4.18E-02
KHK	rs12474906	1.71E-02	2.98E-02	NA	4.18E-02
HIST1H2BM	rs9468350	2.63E-02	2.99E-02	NA	4.18E-02
SNHG17	rs12625702	1.95E-02	3.00E-02	NA	4.18E-02
KCNIP2	rs284857	1.79E-02	3.01E-02	NA	4.19E-02
CLCN3	rs4434205	1.52E-02	3.03E-02	NA	4.20E-02
PEX16	rs11038864	2.09E-02	3.04E-02	NA	4.20E-02
SLC35E2B	rs942820	2.11E-02	3.03E-02	NA	4.20E-02
SV2A	rs72694962	1.96E-02	3.04E-02	NA	4.20E-02
GLT8D1	rs2276834	1.92E-02	3.06E-02	NA	4.22E-02
GNL1	rs9501082	2.06E-02	3.08E-02	NA	4.23E-02
AKT3	rs3006916	1.93E-02	3.14E-02	NA	4.25E-02
CBFB	rs56303487	1.57E-02	3.11E-02	NA	4.25E-02
FCGRT	rs7047	2.20E-02	3.11E-02	NA	4.25E-02
HIC1	rs11078018	2.07E-02	3.12E-02	NA	4.25E-02

Gene	topSNP	p_eQTL	p_SMR	p_HEIDI	adjp_SMR
HIST1H2AE	rs66972160	2.38E-02	3.14E-02	NA	4.25E-02
RBKS	rs12474906	1.84E-02	3.14E-02	NA	4.25E-02
RP11-196G18.22	rs72694955	2.00E-02	3.13E-02	NA	4.25E-02
RP11-54A4.2	rs72694955	2.01E-02	3.14E-02	NA	4.25E-02
WDR46	rs1003878	2.52E-02	3.10E-02	NA	4.25E-02
DCP1A	rs2019065	1.74E-02	3.16E-02	NA	4.26E-02
BTBD1	rs2135551	1.98E-02	3.17E-02	NA	4.26E-02
HYAL3	rs9852677	7.82E-04	3.17E-02	NA	4.26E-02
TAC3	rs324015	2.28E-02	3.19E-02	NA	4.27E-02
ZBTB9	rs73409643	2.71E-02	3.20E-02	NA	4.29E-02
FAM213B	rs942820	2.27E-02	3.22E-02	NA	4.29E-02
HLA-DOA	rs9394145	2.10E-02	3.27E-02	NA	4.35E-02
BTN2A3P	rs66972160	2.51E-02	3.28E-02	NA	4.36E-02
ACO2	rs9607850	2.15E-02	3.33E-02	NA	4.42E-02
MIR4519	rs4424923	2.39E-02	3.38E-02	NA	4.46E-02
FUT1	rs57940349	2.15E-02	3.38E-02	NA	4.46E-02
PPP1R11	rs9257834	2.21E-02	3.45E-02	NA	4.54E-02
EPC2	rs76355118	2.03E-02	3.22E-02	NA	4.58E-02
PITPNM2	rs7296418	2.57E-02	3.50E-02	NA	4.59E-02
SLC5A6	rs12474906	2.15E-02	3.50E-02	NA	4.59E-02
HFE	rs12199613	2.89E-02	3.54E-02	NA	4.62E-02
HMGA1	rs10947436	2.54E-02	3.54E-02	NA	4.62E-02
KRT8P26	rs1193851	2.26E-02	3.53E-02	NA	4.62E-02
TTLL1	rs134885	2.66E-02	3.59E-02	NA	4.67E-02
PBX4	rs62135552	2.55E-02	3.61E-02	NA	4.68E-02
HIST2H2AA4	rs72694962	2.46E-02	3.62E-02	NA	4.69E-02
HNRNPA0	rs3756766	2.27E-02	3.65E-02	NA	4.71E-02
TSPAN9	rs3829331	3.08E-02	3.67E-02	NA	4.73E-02
CTA-223H9.9	rs133379	2.65E-02	3.69E-02	NA	4.74E-02
RP4-669P10.19	rs9607850	2.47E-02	3.71E-02	NA	4.76E-02
RP11-561C5.4	rs17598603	2.62E-02	3.72E-02	NA	4.76E-02
WDR73	rs12905223	2.46E-02	3.72E-02	NA	4.76E-02
PSMG3-AS1	rs4719366	2.84E-02	3.74E-02	NA	4.77E-02
SLC35G2	rs7349597	2.71E-02	3.75E-02	NA	4.78E-02
NKAPL	rs11752496	3.24E-02	3.79E-02	NA	4.82E-02
CYCS	rs79210963	2.55E-02	3.84E-02	NA	4.87E-02
RP11-245J9.5	rs13272	2.80E-02	3.91E-02	NA	4.95E-02
RP11-47A8.5	rs3934495	2.87E-02	3.91E-02	NA	4.95E-02
RRP7B	rs9607850	2.66E-02	3.92E-02	NA	4.95E-02
VRK3	rs7047	2.98E-02	3.97E-02	NA	5.00E-02

(2) The most significant SMR analyses results in brain tissues from GTEx.

Gene	topSNP	p_eQTL	p_SMR	p_HEIDI	adjp_SMR
BTN3A2	rs3047288	1.47E-14	3.86E-10	0.34	2.26E-07
HLA-DMA	rs73409643	8.12E-18	7.02E-10	NA	4.72E-07
HCG4	rs1610645	6.50E-22	5.58E-09	0.06	1.63E-06
C4A	rs3129950	2.55E-11	1.01E-08	0.05	3.38E-06
LY6G5C	rs1046089	5.06E-14	2.70E-08	NA	6.05E-06
RP11-753C18.8	rs7911789	5.03E-13	1.25E-07	NA	2.10E-05
CYP21A1P	rs3129950	5.33E-10	5.66E-08	0.07	2.24E-05
PRSS16	rs13205911	5.09E-09	8.55E-08	0.19	2.91E-05
AS3MT	rs35525740	5.87E-13	1.80E-07	NA	4.09E-05
MICE	rs1737082	1.65E-13	3.38E-07	0.06	4.70E-05
RNF5	rs3130281	3.15E-09	3.44E-07	0.19	4.70E-05
HLA-C	rs6457375	6.70E-13	4.44E-07	0.23	5.86E-05
RP11-724N1.1	rs7911789	5.38E-11	5.49E-07	NA	6.24E-05
HLA-F	rs1610720	3.16E-11	5.36E-07	0.07	7.21E-05
AC103965.1	rs12905223	2.06E-23	1.38E-07	0.36	7.94E-05
NAGA	rs6519298	4.42E-16	7.11E-07	0.07	7.96E-05
HLA-DMB	rs73409643	4.77E-09	1.05E-06	0.22	8.93E-05
VARS2	rs3131787	1.40E-08	9.89E-07	0.47	8.93E-05
BAG6	rs2736176	1.77E-10	1.31E-06	0.27	8.93E-05
WASF5P	rs9266231	3.34E-10	1.25E-06	0.05	8.93E-05
HLA-DQB2	rs1130145	1.43E-14	3.22E-07	0.43	9.28E-05
INO80E	rs4583255	7.45E-11	2.01E-06	0.21	1.14E-04
NDUFA6-AS1	rs8135801	3.67E-15	1.95E-06	0.12	1.14E-04
C10orf32	rs35525740	1.27E-09	2.17E-06	NA	1.14E-04
HLA-V	rs1610720	3.95E-10	1.29E-06	0.26	1.19E-04
XXbac-BPG154L12.4	rs6457513	3.60E-09	9.61E-07	NA	1.19E-04
PITPNM2	rs1051434	1.69E-10	2.60E-06	0.13	1.27E-04
CYP21A2	rs1265754	2.81E-07	3.03E-06	0.46	1.29E-04
ZSCAN9	rs3131336	6.20E-07	2.85E-06	0.31	1.29E-04
ITIH4	rs13072536	1.05E-17	3.61E-06	NA	1.38E-04
RP4-669P10.19	rs8135801	2.39E-13	3.65E-06	0.12	1.38E-04
ALMS1P	rs73947808	5.25E-15	4.34E-06	NA	1.56E-04
ZSCAN31	rs213238	6.91E-13	6.15E-07	0.08	1.67E-04
CYP2D6	rs5758605	2.49E-14	8.21E-07	0.51	1.67E-04
DDAH2	rs1046089	4.36E-08	5.09E-06	0.14	1.74E-04
ZNF184	rs45527431	5.84E-08	5.87E-07	0.41	1.77E-04
HCG27	rs1130592	3.38E-09	5.98E-06	0.33	1.94E-04
TYW5	rs281771	5.57E-09	2.38E-06	0.98	2.00E-04
CACNA1C	rs11062170	4.08E-08	3.30E-06	0.53	2.09E-04
RP13-98N21.3	rs75884951	6.06E-18	3.43E-06	0.11	2.09E-04
HLA-F-AS1	rs416571	7.33E-14	2.05E-06	NA	2.17E-04

Gene	topSNP	p_eQTL	p_SMR	p_HEIDI	adjp_SMR
TM6SF2	rs4808203	3.54E-09	7.70E-06	0.83	2.39E-04
MSH5	rs3115674	8.53E-08	1.33E-06	0.37	2.59E-04
RP11-806K15.1	rs7673823	7.64E-11	5.46E-06	NA	2.82E-04
FTSJ2	rs4719431	8.14E-08	9.54E-06	0.30	2.83E-04
CD46	rs2745977	9.49E-20	1.04E-05	NA	2.84E-04
BRD2	rs73409643	2.91E-08	2.61E-06	NA	3.10E-04
RP11-102M11.2	rs696520	9.37E-12	1.98E-06	0.37	3.18E-04
C2orf82	rs2675956	8.09E-09	4.22E-06	0.30	3.41E-04
RPL23AP1	rs1610720	3.25E-08	7.48E-06	0.10	3.59E-04
HLA-DRB5	rs72849267	4.58E-11	6.11E-06	0.07	3.90E-04
RP11-890B15.3	rs62621284	6.92E-16	5.60E-06	NA	3.90E-04
RP4-669P10.16	rs8135801	2.28E-20	1.03E-05	0.12	4.50E-04
PPAPDC1B	rs1488936	1.61E-09	2.03E-05	0.70	4.94E-04
PRR12	rs57940349	1.99E-11	2.03E-05	NA	4.94E-04
ZKSCAN8	rs34130214	3.10E-06	2.12E-05	0.13	5.00E-04
ARL3	rs7911789	4.83E-07	2.42E-05	0.27	5.51E-04
RP5-874C20.3	rs213238	1.06E-09	4.68E-06	0.15	5.54E-04
HCG4P3	rs1061815	2.78E-08	2.80E-06	0.11	5.72E-04
HLA-K	rs1264352	2.07E-07	3.59E-06	0.28	5.72E-04
SF3B1	rs3792159	1.38E-07	2.64E-05	0.72	5.80E-04
THOC7	rs7615475	7.00E-09	2.85E-05	0.83	6.08E-04
DDHD2	rs9198	5.31E-09	3.03E-05	0.86	6.14E-04
GLYCTK	rs1060330	7.36E-10	3.32E-05	0.16	6.14E-04
NAT8	rs11903916	5.13E-10	3.18E-05	NA	6.14E-04
DNM1P51	rs2135551	1.02E-12	2.77E-06	0.48	6.18E-04
PACSIN3	rs12272795	2.01E-07	3.56E-05	0.51	6.23E-04
CENPM	rs1894713	6.93E-11	4.10E-06	0.34	6.58E-04
CHRNA2	rs2565065	1.77E-08	3.89E-05	NA	6.63E-04
HLA-DRB6	rs7195	6.38E-09	1.04E-05	0.56	6.71E-04
HCG23	rs1003878	1.80E-07	1.16E-05	0.06	6.83E-04
RP11-73M18.8	rs3212090	7.09E-07	4.34E-05	0.53	7.05E-04
HLA-DQB1	rs1130126	9.79E-07	4.50E-05	0.57	7.12E-04
STK19P	rs3129950	1.21E-05	4.59E-05	0.51	7.12E-04
ZNF192P2	rs9301	2.11E-08	1.42E-05	0.09	7.13E-04
C6orf48	rs2001097	9.64E-06	4.76E-05	0.30	7.21E-04
ZNF192P1	rs760587	3.23E-07	1.11E-05	0.71	7.76E-04
ZSCAN23	rs1416920	1.29E-06	1.72E-05	0.24	8.37E-04
C4B	rs2233974	1.28E-06	2.09E-05	0.08	8.49E-04
HCG17	rs1634721	2.64E-06	1.42E-05	0.38	8.49E-04
NOTCH4	rs204994	1.92E-06	2.17E-05	0.05	8.49E-04
RP11-275H4.1	rs1871355	1.00E-10	2.42E-05	NA	8.49E-04
RP11-457M11.5	rs13217675	4.02E-09	1.99E-05	0.10	8.49E-04
XXbac-BPG299F13.17	rs9266244	2.73E-07	1.75E-05	0.55	8.49E-04

Gene	topSNP	p_eQTL	p_SMR	p_HEIDI	adjp_SMR
GPSM3	rs204994	9.62E-06	6.27E-05	0.17	8.49E-04
PPM1M	rs7629072	1.68E-08	6.48E-05	0.62	8.49E-04
CSPG4P11	rs2135551	6.11E-08	4.41E-05	0.26	9.09E-04
SNX19	rs4936123	4.44E-12	2.25E-05	0.11	9.27E-04
WBP2NL	rs6519298	2.69E-09	1.62E-05	0.13	9.76E-04
BAK1	rs210142	2.13E-09	3.26E-05	NA	9.87E-04
GNL3	rs11235	9.19E-09	3.54E-05	0.32	9.92E-04
IFITM4P	rs1633081	2.48E-07	3.28E-05	0.19	9.93E-04
FAM86B3P	rs2948286	3.51E-19	6.77E-05	NA	1.12E-03
ATAT1	rs1264352	2.22E-06	1.73E-05	0.39	1.18E-03
FAM85B	rs2948286	5.83E-18	7.65E-05	NA	1.19E-03
CFB	rs3132935	3.14E-06	3.00E-05	0.48	1.19E-03
FTCDNL1	rs796364	5.81E-07	1.84E-05	0.45	1.20E-03
LY6G5B	rs1046089	1.19E-05	1.10E-04	NA	1.29E-03
RP11-282O18.3	rs585522	7.67E-07	1.10E-04	0.35	1.29E-03
RPS20P22	rs2411256	4.05E-07	1.07E-04	0.72	1.29E-03
HLA-DRB1	rs7195	4.01E-07	4.94E-05	0.65	1.37E-03
HLA-U	rs112863520	4.54E-07	5.32E-05	0.05	1.41E-03
GOLGA6L10	rs1269134	3.60E-07	1.29E-04	0.83	1.48E-03
IRF3	rs7047	1.72E-06	1.30E-04	NA	1.48E-03
HLA-J	rs1061815	1.76E-06	2.78E-05	0.07	1.48E-03
HLA-DQB1-AS1	rs2858310	4.60E-08	4.97E-05	NA	1.54E-03
XXbac-BPG181B23.7	rs9266797	1.85E-08	5.02E-05	NA	1.54E-03
POU5F1	rs3925616	1.35E-06	1.43E-04	0.56	1.57E-03
SFMBT1	rs4687680	2.10E-06	1.42E-04	0.33	1.57E-03
IGSF9B	rs73036062	7.73E-06	1.45E-04	NA	1.57E-03
SMDT1	rs6002597	5.05E-08	5.66E-05	0.12	1.66E-03
CYP2D8P1	rs134885	3.88E-06	1.68E-04	0.46	1.71E-03
HCG4P7	rs416571	8.24E-07	1.21E-04	NA	1.71E-03
C6orf100	rs3116856	1.32E-06	3.85E-05	0.48	1.72E-03
YPEL3	rs4788211	8.83E-08	9.04E-05	0.08	1.77E-03
XRCC3	rs35498576	1.19E-05	2.00E-04	0.83	2.01E-03
CSPG4P12	rs12905223	3.08E-08	3.61E-05	0.37	2.05E-03
PGBD1	rs34130214	3.50E-05	1.22E-04	NA	2.16E-03
HLA-B	rs2523599	6.48E-07	7.44E-05	0.52	2.17E-03
HLA-DQA2	rs2858310	3.46E-06	2.30E-04	NA	2.27E-03
GOLGA6L5	rs950169	1.73E-07	5.18E-05	0.48	2.34E-03
CPT1C	rs7047	7.32E-06	2.45E-04	NA	2.39E-03
PCCB	rs696520	1.68E-06	1.03E-04	0.64	2.43E-03
RNASEH2C	rs1193851	3.95E-08	1.07E-04	NA	2.44E-03
SMCR5	rs9907287	5.63E-08	2.58E-04	0.42	2.48E-03
HCG4B	rs1629068	9.53E-06	9.36E-05	0.38	2.52E-03
RP1-265C24.5	rs200969	2.96E-06	6.05E-05	0.10	2.52E-03

Gene	topSNP	p_eQTL	p_SMR	p_HEIDI	adjp_SMR
BTN2A2	rs3208733	1.79E-07	1.04E-04	NA	2.55E-03
OLA1P1	rs5758645	1.48E-06	1.07E-04	0.19	2.55E-03
TOM1L2	rs4584886	1.89E-07	1.97E-04	0.53	2.57E-03
LEMD2	rs487835	3.64E-06	2.75E-04	0.38	2.61E-03
TOB2P1	rs1416920	2.17E-05	1.11E-04	0.19	2.63E-03
TSNARE1	rs7832163	7.77E-07	1.69E-04	NA	2.74E-03
GDPD3	rs4424923	8.86E-06	3.00E-04	NA	2.76E-03
RP11-731C17.2	rs9845788	9.85E-07	1.89E-04	0.45	2.77E-03
TMEM180	rs284854	2.80E-07	1.25E-04	NA	2.78E-03
SDAD1P1	rs3808581	4.32E-06	3.12E-04	NA	2.84E-03
ZNF391	rs35555795	3.58E-05	9.84E-05	0.26	2.88E-03
SERBP1P3	rs4687680	4.85E-06	2.04E-04	0.30	2.91E-03
FAM154B	rs75884951	5.49E-07	2.11E-04	0.26	2.95E-03
RCCD1	rs4702	1.54E-05	2.36E-04	NA	3.18E-03
FLOT1	rs3129984	1.01E-04	2.45E-04	0.46	3.23E-03
BTBD1	rs4779046	7.07E-07	1.91E-04	NA	3.26E-03
TCF19	rs9262696	1.03E-06	2.15E-04	0.21	3.26E-03
RP11-425L10.1	rs6485685	4.03E-06	1.63E-04	NA	3.29E-03
ZKSCAN3	rs9301	1.23E-05	1.44E-04	0.40	3.42E-03
FAM109B	rs6002597	3.36E-06	2.44E-04	0.30	3.62E-03
NT5C2	rs79237883	3.59E-05	2.03E-04	0.84	3.70E-03
RFT1	rs4687680	8.21E-06	2.58E-04	NA	3.74E-03
AC091969.1	rs17489682	4.49E-06	2.18E-04	0.67	3.74E-03
CCHCR1	rs887465	5.75E-05	2.31E-04	NA	3.74E-03
NMB	rs17598603	2.15E-06	2.07E-04	0.94	3.74E-03
WDR73	rs11631921	2.00E-07	1.72E-04	0.10	3.77E-03
NMUR2	rs2961756	3.79E-07	1.78E-04	0.25	3.79E-03
TSNAXIP1	rs1971546	1.75E-07	1.67E-04	NA	3.83E-03
PSORS1C3	rs3132564	2.85E-05	3.06E-04	NA	3.85E-03
PSORS1C1	rs1049709	2.86E-05	1.09E-04	0.44	3.89E-03
APOPT1	rs56168984	5.32E-06	1.22E-04	0.54	3.95E-03
BTN3A3	rs6940638	5.73E-05	3.18E-04	0.15	3.97E-03
HLA-DOA	rs1003878	5.65E-05	3.13E-04	NA	3.97E-03
RERE	rs302719	3.82E-06	3.07E-04	NA	3.97E-03
SRR	rs2224770	4.22E-07	1.84E-04	NA	4.05E-03
RP5-874C20.6	rs34546986	2.11E-04	3.56E-04	NA	4.27E-03
TDRD9	rs10132641	1.46E-05	3.00E-04	0.15	4.40E-03
ABHD16A	rs1046089	6.79E-05	3.35E-04	NA	4.50E-03
PRMT7	rs1971546	1.57E-06	3.22E-04	NA	4.50E-03
HLA-G	rs3129973	1.80E-04	3.85E-04	0.22	4.54E-03
U91328.19	rs198811	2.48E-05	4.21E-04	NA	4.70E-03
ENPP7P1	rs2948286	2.64E-08	5.11E-04	NA	4.80E-03
PLEKHO1	rs72694957	3.08E-05	5.93E-04	NA	4.87E-03

Gene	topSNP	p_eQTL	p_SMR	p_HEIDI	adjp_SMR
SKIV2L	rs9266244	1.36E-04	6.08E-04	0.25	4.94E-03
CDK2AP1	rs1051434	4.13E-05	4.15E-04	NA	4.96E-03
CUL3	rs3768886	7.11E-06	4.53E-04	NA	4.96E-03
CYP2D7P1	rs2143139	3.02E-05	4.49E-04	NA	4.96E-03
MPHOSPH9	rs4553407	2.36E-05	4.13E-04	0.27	4.96E-03
SETD8	rs11057238	1.25E-05	4.01E-04	0.14	4.96E-03
VPS29	rs12305489	1.08E-05	4.33E-04	NA	4.96E-03
VSIG2	rs12283429	1.95E-05	4.47E-04	NA	4.96E-03
HAPLN4	rs8101938	1.57E-05	6.28E-04	0.28	5.04E-03
LINC00942	rs11062170	6.51E-05	2.79E-04	NA	5.06E-03
MICA	rs9262617	8.13E-06	5.05E-04	0.23	5.11E-03
PBX4	rs880090	1.66E-05	6.52E-04	0.35	5.12E-03
CTSS	rs72694955	1.20E-05	5.26E-04	NA	5.19E-03
INA	rs10430665	1.38E-04	5.06E-04	NA	5.37E-03
TMTC1	rs302317	8.90E-06	6.15E-04	NA	5.38E-03
HIST1H3E	rs34525648	6.84E-05	2.35E-04	NA	5.47E-03
HCG22	rs2233974	2.55E-04	7.17E-04	NA	5.51E-03
HLA-L	rs2523898	4.96E-05	6.04E-04	0.52	5.57E-03
ABCB9	rs10846491	2.14E-05	5.43E-04	0.47	5.67E-03
CILP2	rs8101938	1.57E-05	6.27E-04	0.21	5.70E-03
CTA-250D10.23	rs5996096	2.12E-05	7.82E-04	0.16	5.85E-03
TNFRSF13C	rs1894713	8.92E-05	7.90E-04	0.62	5.85E-03
MYO15A	rs2955365	6.40E-06	7.36E-04	0.63	5.96E-03
MAPK3	rs4424923	1.39E-05	3.68E-04	NA	6.03E-03
GUSBP2	rs2747054	5.16E-05	1.33E-04	NA	6.09E-03
HIST1H2BF	rs6902389	1.55E-04	3.40E-04	0.45	6.13E-03
RP5-966M1.5	rs678	6.97E-06	6.35E-04	0.35	6.14E-03
ABCF1	rs886424	4.70E-04	8.69E-04	NA	6.20E-03
BTNL2	rs3115674	5.03E-04	8.88E-04	0.93	6.20E-03
LINC00240	rs200969	2.41E-04	8.90E-04	NA	6.20E-03
RP11-245J9.4	rs7615475	6.66E-05	8.67E-04	0.96	6.20E-03
ZC3H7B	rs9611488	6.74E-05	9.05E-04	NA	6.23E-03
RP11-863K10.4	rs7005874	4.23E-05	7.42E-04	NA	6.39E-03
PCNXL3	rs1193851	3.25E-05	9.42E-04	NA	6.42E-03
HCG24	rs210142	7.71E-06	4.07E-04	NA	6.44E-03
ZNRD1-AS1	rs1061815	3.78E-04	9.95E-04	NA	6.56E-03
ZNRD1	rs1061815	3.82E-04	1.00E-03	NA	6.56E-03
GLRA1	rs72801007	2.95E-05	6.94E-04	0.45	6.57E-03
EFTUD1P1	rs2135551	6.05E-06	2.53E-04	NA	6.57E-03
TAP1	rs3134940	3.59E-04	8.07E-04	NA	6.59E-03
LRRC48	rs4584886	6.89E-06	5.93E-04	0.59	6.62E-03
RP11-73M18.9	rs11623546	1.13E-05	1.60E-04	NA	6.66E-03
MICB	rs1051788	8.86E-06	4.79E-04	NA	6.73E-03

Gene	topSNP	p_eQTL	p_SMR	p_HEIDI	adjp_SMR
HLA-W	rs1736934	1.32E-04	1.09E-03	0.09	6.98E-03
ATF6B	rs2736176	2.00E-05	2.17E-04	0.41	7.04E-03
HIST1H1C	rs9348746	2.31E-04	1.12E-03	NA	7.08E-03
EP300	rs9611469	1.11E-04	1.16E-03	NA	7.23E-03
AGPAT1	rs204994	1.49E-04	4.38E-04	0.17	7.24E-03
HLA-DQA1	rs2858310	4.61E-05	7.25E-04	NA	7.33E-03
C2	rs130065	1.06E-04	3.31E-04	NA	7.47E-03
HLA-H	rs2535332	1.36E-04	3.07E-04	NA	7.47E-03
BTN1A1	rs28551159	6.97E-04	1.22E-03	0.94	7.50E-03
AGER	rs73409643	9.53E-05	3.57E-04	NA	7.52E-03
BTN3A1	rs3208733	4.01E-05	8.03E-04	NA	7.54E-03
C6orf136	rs2523898	8.54E-05	8.05E-04	NA	7.54E-03
NDUFAF2	rs4583848	7.87E-05	4.87E-04	0.86	7.62E-03
ZBTB42	rs2223937	5.52E-05	5.00E-04	NA	7.67E-03
GPANK1	rs1046089	3.29E-04	9.93E-04	NA	7.71E-03
PSMG3-AS1	rs3778970	6.90E-05	5.22E-04	0.23	7.81E-03
RP11-182J1.14	rs2135551	3.29E-05	5.53E-04	NA	7.93E-03
HSPA1L	rs1264318	3.93E-04	9.72E-04	NA	7.97E-03
ACO2	rs9611488	1.44E-04	1.33E-03	NA	8.02E-03
MDK	rs12574918	7.11E-05	9.43E-04	NA	8.12E-03
LPAR2	rs8101938	5.03E-05	1.03E-03	NA	8.20E-03
COL11A2	rs73409626	4.51E-04	1.11E-03	NA	8.28E-03
CDIPT-AS1	rs4583255	8.56E-05	6.26E-04	NA	8.47E-03
POC1A	rs11177	2.95E-06	6.43E-04	NA	8.54E-03
RP11-490G2.2	rs1702294	2.53E-04	7.36E-04	0.57	8.57E-03
ZNF204P	rs6940638	2.16E-04	7.72E-04	NA	8.68E-03
XXbac-BPG252P9.10	rs3095153	3.95E-04	7.14E-04	NA	8.69E-03
SLC44A4	rs2233974	4.54E-04	1.10E-03	NA	8.73E-03
RP1-86C11.7	rs6940638	1.12E-04	4.92E-04	NA	8.75E-03
CPEB1	rs1145173	9.34E-05	1.12E-03	NA	8.76E-03
DDX39B	rs74618856	2.99E-04	1.20E-03	NA	8.97E-03
LINC00634	rs9611488	9.73E-05	1.09E-03	NA	9.01E-03
RP5-1157M23.2	rs7622851	6.38E-05	1.12E-03	NA	9.01E-03
HIST1H4I	rs35345226	5.01E-04	7.58E-04	0.53	9.29E-03
SLCO4C1	rs3114661	4.17E-06	1.22E-03	NA	9.38E-03
HCG20	rs2523898	2.14E-04	1.34E-03	NA	9.41E-03
DPYD	rs7415086	2.09E-04	8.43E-04	NA	9.47E-03
C6orf25	rs2736176	4.21E-04	1.45E-03	NA	9.62E-03
CD2BP2	rs4424923	2.07E-04	1.44E-03	NA	9.62E-03
USP8P1	rs1960278	6.27E-04	1.70E-03	NA	9.68E-03
LINC00606	rs61432137	2.57E-05	8.58E-04	NA	9.68E-03
RPP21	rs1629068	1.65E-04	6.01E-04	0.23	9.71E-03
CTCF	rs1971546	3.90E-05	1.04E-03	NA	9.84E-03

Gene	topSNP	p_eQTL	p_SMR	p_HEIDI	adjp_SMR
SAPCD1-AS1	rs3094605	1.16E-03	1.75E-03	NA	9.88E-03
MED19	rs708228	2.67E-04	1.80E-03	NA	9.90E-03
PTK2B	rs2565065	2.62E-04	1.80E-03	NA	9.90E-03
SREBF2	rs9611488	2.60E-04	1.82E-03	NA	9.90E-03
HLA-T	rs1063320	1.61E-04	1.84E-03	0.28	9.94E-03
HLA-DPB1	rs1003878	5.21E-04	1.43E-03	NA	1.01E-02
C6orf47	rs1046089	3.97E-04	1.14E-03	NA	1.02E-02
CNNM2	rs77420391	5.88E-04	1.46E-03	NA	1.02E-02
RP11-347C12.2	rs4424923	8.53E-05	8.95E-04	NA	1.03E-02
NRM	rs1737082	5.61E-04	1.94E-03	NA	1.04E-02
VPS45	rs1824850	6.22E-05	8.43E-04	NA	1.04E-02
PPT2	rs3132451	9.18E-04	1.63E-03	NA	1.06E-02
RP11-631M6.2	rs4583848	6.77E-04	1.99E-03	NA	1.06E-02
OVOL1	rs1193851	1.82E-04	2.02E-03	NA	1.07E-02
ZSCAN12P1	rs2275508	7.03E-04	2.03E-03	NA	1.07E-02
STAT2	rs324015	1.22E-04	9.79E-04	NA	1.08E-02
PPP1R18	rs9266231	5.07E-04	1.56E-03	0.97	1.09E-02
PAIP1P1	rs9262696	3.02E-05	7.80E-04	NA	1.09E-02
HCG21	rs2596473	2.85E-05	6.16E-04	NA	1.09E-02
MKLN1-AS1	rs10954343	2.17E-04	2.15E-03	NA	1.10E-02
PACSIN1	rs942496	3.08E-04	2.14E-03	NA	1.10E-02
XXbac-BPG157A10.21	rs5021453	8.54E-04	2.14E-03	NA	1.10E-02
LY6G6C	rs130079	7.49E-04	1.60E-03	NA	1.11E-02
DGKZ	rs12576115	6.62E-04	2.21E-03	NA	1.12E-02
DBF4	rs76349903	3.04E-04	2.24E-03	NA	1.12E-02
RP11-524F11.1	rs9890563	8.72E-05	2.23E-03	NA	1.12E-02
RPRD2	rs72694965	4.05E-04	2.26E-03	NA	1.12E-02
LY6E	rs7832163	1.99E-04	1.74E-03	NA	1.12E-02
C11orf49	rs12574918	4.75E-05	7.77E-04	0.06	1.13E-02
LINC00933	rs12905223	1.59E-04	1.80E-03	0.85	1.14E-02
CDIPT	rs4583255	4.39E-04	1.71E-03	NA	1.14E-02
AL049840.1	rs2368560	9.58E-05	6.71E-04	0.87	1.15E-02
AIF1	rs1264352	1.14E-03	1.92E-03	NA	1.15E-02
ZFYVE21	rs2368560	5.26E-04	1.92E-03	NA	1.15E-02
KIF26A	rs56168984	5.15E-04	1.78E-03	NA	1.16E-02
RP13-467H17.1	rs7462788	2.76E-04	1.11E-03	NA	1.16E-02
HLA-DPA1	rs5021453	9.89E-04	2.38E-03	NA	1.17E-02
DNAJB7	rs9607850	7.45E-05	1.21E-03	NA	1.18E-02
RP11-18I14.10	rs284856	1.50E-04	2.07E-03	NA	1.18E-02
RP11-455F5.3	rs4583255	5.79E-04	2.04E-03	NA	1.18E-02
BTN2A1	rs3208733	2.82E-04	2.09E-03	NA	1.18E-02
ARTN	rs3001723	4.17E-04	1.78E-03	NA	1.18E-02
CTD-3051D23.4	rs10132641	5.03E-04	2.12E-03	NA	1.18E-02

Gene	topSNP	p_eQTL	p_SMR	p_HEIDI	adjp_SMR
PRRT1	rs1049709	1.27E-03	2.13E-03	NA	1.18E-02
VWA7	rs1041885	1.30E-03	2.17E-03	NA	1.20E-02
ALMS1	rs73947808	1.21E-04	1.45E-03	NA	1.20E-02
DUS2	rs1971546	2.07E-04	2.19E-03	NA	1.20E-02
RP11-182J1.5	rs12905223	1.79E-04	1.34E-03	NA	1.20E-02
FKBPL	rs204994	7.31E-04	1.48E-03	NA	1.20E-02
BRF2	rs7005874	1.16E-04	1.22E-03	NA	1.21E-02
CKB	rs34633710	9.78E-05	1.19E-03	0.23	1.21E-02
CTD-3065B20.1	rs4702	2.43E-04	1.16E-03	NA	1.21E-02
HIST1H2BK	rs6940638	3.87E-04	1.16E-03	NA	1.21E-02
SKI	rs2477686	1.52E-04	1.19E-03	NA	1.21E-02
TBX6	rs4788211	8.82E-05	1.20E-03	NA	1.21E-02
RP1-97D16.1	rs760587	8.73E-04	1.90E-03	NA	1.22E-02
3-Sep	rs5996096	1.55E-04	1.85E-03	0.15	1.22E-02
GATAD2A	rs4808203	3.11E-04	1.42E-03	NA	1.22E-02
SPATS2L	rs59695400	2.05E-04	1.45E-03	0.10	1.22E-02
RP11-600F24.7	rs4906379	2.16E-04	1.06E-03	0.64	1.23E-02
ATP13A1	rs880090	3.35E-04	2.55E-03	NA	1.23E-02
RP13-98N21.2	rs75884951	1.11E-04	1.55E-03	NA	1.23E-02
HSPA1B	rs1264318	4.63E-04	1.10E-03	NA	1.25E-02
GLT8D1	rs11177	1.82E-05	1.14E-03	NA	1.26E-02
MDC1	rs1633081	8.72E-04	2.65E-03	NA	1.26E-02
TRIM26	rs3094125	1.29E-03	2.02E-03	NA	1.27E-02
GPM6A	rs7673823	6.62E-04	2.69E-03	NA	1.27E-02
SDCCAG8	rs10803138	3.28E-04	2.06E-03	NA	1.28E-02
HCG18	rs1049709	6.21E-04	1.18E-03	NA	1.28E-02
DEPDC1B	rs4583848	7.86E-04	2.21E-03	NA	1.28E-02
HCG11	rs2235251	1.45E-03	2.21E-03	NA	1.28E-02
HLA-DPA3	rs210142	3.02E-04	2.20E-03	NA	1.28E-02
GNL1	rs1264352	7.53E-04	1.37E-03	NA	1.29E-02
RFTN2	rs12693816	2.08E-04	1.37E-03	NA	1.29E-02
TMEM110	rs13072536	8.07E-05	1.35E-03	NA	1.29E-02
RAI1	rs4584886	1.85E-04	2.22E-03	NA	1.31E-02
PLCL1	rs4850812	5.56E-04	2.85E-03	0.81	1.32E-02
RP11-345P4.6	rs10797426	7.21E-04	2.81E-03	NA	1.32E-02
VPS52	rs1048645	9.98E-04	2.87E-03	NA	1.32E-02
DCTPP1	rs4424923	4.26E-04	2.17E-03	NA	1.32E-02
PAIP2	rs256013	2.54E-04	2.20E-03	NA	1.32E-02
GPER1	rs3800882	7.41E-04	2.47E-03	0.25	1.33E-02
RPL3P2	rs3134900	8.69E-04	1.46E-03	NA	1.33E-02
EIF5	rs3212090	3.31E-04	1.30E-03	0.83	1.33E-02
USMG5	rs10883798	4.42E-04	1.25E-03	NA	1.33E-02
PPP1R11	rs112863520	9.50E-04	2.99E-03	NA	1.34E-02

Gene	topSNP	p_eQTL	p_SMR	p_HEIDI	adjp_SMR
SEMA6C	rs72694955	5.00E-04	3.00E-03	NA	1.34E-02
GTF2H4	rs74618856	9.08E-04	2.54E-03	NA	1.34E-02
HSD17B8	rs210142	4.14E-04	2.60E-03	NA	1.34E-02
ALKBH5	rs9907287	2.15E-04	3.06E-03	NA	1.35E-02
XXbac-BPG299F13.14	rs1131165	7.55E-04	2.33E-03	0.99	1.35E-02
ACTR5	rs4812317	1.36E-03	3.14E-03	NA	1.36E-02
ALPK3	rs17598603	6.91E-04	3.18E-03	0.64	1.36E-02
ATP6V1G2	rs9264640	1.14E-03	3.18E-03	NA	1.36E-02
RP11-77H9.6	rs8058276	5.77E-05	1.44E-03	0.26	1.36E-02
OGFOD2	rs4275659	7.49E-04	2.66E-03	NA	1.36E-02
RP11-182J1.16	rs2135551	3.62E-04	2.70E-03	0.83	1.36E-02
RP1-257I20.14	rs8135801	7.21E-04	3.25E-03	NA	1.38E-02
TRIM27	rs1610721	1.11E-03	2.76E-03	NA	1.38E-02
RP11-182J1.1	rs1051168	2.63E-04	1.84E-03	0.90	1.40E-02
REEP2	rs55985421	8.44E-04	3.35E-03	NA	1.40E-02
C7orf50	rs6950330	9.37E-04	2.59E-03	NA	1.41E-02
HSPA1A	rs2075800	9.60E-04	2.61E-03	NA	1.41E-02
TCF4	rs784255	2.17E-04	1.16E-03	NA	1.41E-02
HIST2H2BF	rs7555080	6.77E-04	3.43E-03	NA	1.41E-02
LPXN	rs708228	1.85E-04	1.48E-03	NA	1.41E-02
SCAND3	rs7750526	7.60E-04	1.53E-03	0.68	1.41E-02
KRT8P26	rs1193851	5.55E-04	3.52E-03	NA	1.42E-02
TULP3	rs10774033	1.65E-03	3.50E-03	NA	1.42E-02
RP1-85F18.5	rs9611469	7.78E-05	9.65E-04	NA	1.42E-02
ZSCAN16	rs1150670	3.36E-04	1.23E-03	0.45	1.43E-02
CACNA1I	rs1008677	4.01E-04	1.62E-03	NA	1.44E-02
MARK3	rs12891288	1.42E-05	6.88E-04	0.07	1.44E-02
HYI	rs3001723	1.71E-04	1.03E-03	NA	1.44E-02
PHF5A	rs6519298	6.22E-04	2.68E-03	NA	1.45E-02
ASPHD1	rs4424923	2.60E-04	1.64E-03	NA	1.45E-02
NEK4	rs11717383	7.09E-04	3.64E-03	NA	1.45E-02
MAD1L1	rs13227554	1.56E-03	2.97E-03	NA	1.45E-02
OTUD7B	rs72694965	6.39E-04	2.94E-03	NA	1.45E-02
GOLGA6L9	rs75884951	1.44E-04	1.75E-03	NA	1.47E-02
CSDC2	rs5758268	2.36E-04	1.98E-03	NA	1.50E-02
CTD-2589M5.5	rs61884292	4.04E-04	1.83E-03	NA	1.50E-02
GPIHBP1	rs7462788	6.60E-04	2.00E-03	NA	1.50E-02
TNXA	rs1142323	6.83E-04	1.91E-03	NA	1.50E-02
CISD2	rs13107325	1.37E-03	2.83E-03	NA	1.50E-02
LINC00336	rs210142	4.81E-04	2.82E-03	NA	1.50E-02
MADD	rs10838634	1.11E-03	3.82E-03	NA	1.51E-02
MSL2	rs7349597	1.73E-04	1.35E-03	NA	1.51E-02
FSD2	rs75884951	8.57E-05	1.38E-03	NA	1.52E-02

Gene	topSNP	p_eQTL	p_SMR	p_HEIDI	adjp_SMR
FANCL	rs2678915	8.91E-04	3.89E-03	NA	1.52E-02
RGL2	rs3129950	2.05E-03	2.95E-03	NA	1.53E-02
WDR46	rs5021453	1.36E-03	3.02E-03	NA	1.53E-02
NOL5BP	rs9468350	1.19E-03	2.00E-03	NA	1.54E-02
SPDEF	rs2395401	3.86E-04	1.99E-03	NA	1.54E-02
SLC35G2	rs7349597	3.90E-04	2.12E-03	NA	1.55E-02
MRPS18B	rs1610721	1.39E-03	3.26E-03	NA	1.56E-02
EHMT2	rs2001097	1.09E-03	1.84E-03	NA	1.56E-02
hsa-mir-8072	rs7304782	1.38E-03	3.15E-03	NA	1.57E-02
BAP1	rs6769789	9.51E-04	3.43E-03	NA	1.58E-02
VPS51	rs1193851	5.25E-04	3.42E-03	NA	1.58E-02
FIBP	rs1193851	4.67E-04	3.22E-03	NA	1.59E-02
IER3	rs3129984	1.17E-03	1.84E-03	NA	1.59E-02
PPP1R13B	rs2368560	5.45E-04	1.97E-03	NA	1.59E-02
SULT1A4	rs4424923	3.57E-04	1.96E-03	NA	1.59E-02
AC110781.3	rs872465	4.64E-04	2.48E-03	NA	1.60E-02
SLK	rs79237883	1.21E-03	2.50E-03	NA	1.60E-02
RP5-1104E15.6	rs9306336	1.77E-04	1.99E-03	NA	1.60E-02
FAM65B	rs34525648	1.49E-03	2.56E-03	NA	1.61E-02
FXR1	rs1805589	7.87E-04	2.58E-03	NA	1.61E-02
SAP30L-AS1	rs11740474	4.67E-04	3.01E-03	NA	1.61E-02
PHF7	rs13072536	2.07E-04	2.09E-03	NA	1.61E-02
RP11-661D19.3	rs34633710	3.04E-04	2.12E-03	NA	1.61E-02
SLC25A5P1	rs133347	1.23E-03	4.15E-03	NA	1.62E-02
MIR137HG	rs4506495	1.63E-03	3.59E-03	NA	1.62E-02
VRK3	rs7047	3.99E-04	1.97E-03	NA	1.62E-02
AC156455.1	rs883563	1.03E-03	4.19E-03	NA	1.62E-02
UBE2Q2P1	rs3803405	4.99E-04	4.25E-03	0.41	1.63E-02
PDCD11	rs284854	5.72E-04	3.10E-03	NA	1.63E-02
DNAJC19	rs16832165	6.36E-04	2.31E-03	NA	1.64E-02
C1QTNF4	rs10838634	5.05E-04	2.35E-03	NA	1.65E-02
RP11-894P9.1	rs11623546	2.07E-03	4.47E-03	NA	1.65E-02
SEMA3G	rs1029871	6.32E-04	4.45E-03	NA	1.65E-02
VARS	rs2596473	5.25E-04	2.73E-03	NA	1.65E-02
RN7SL471P	rs3131336	2.71E-03	3.40E-03	NA	1.67E-02
CSNK2B	rs2075800	1.10E-03	2.88E-03	NA	1.67E-02
CTD-2126E3.3	rs7047	7.64E-04	2.92E-03	NA	1.67E-02
HIST1H2AC	rs3208733	5.17E-04	2.89E-03	NA	1.67E-02
ATXN7L1	rs7807853	7.07E-04	3.32E-03	NA	1.68E-02
DTX3	rs73115999	1.87E-04	2.27E-03	NA	1.69E-02
ZNF101	rs880090	7.22E-04	3.83E-03	NA	1.69E-02
AC093590.1	rs281771	8.75E-04	2.11E-03	NA	1.70E-02
APOM	rs1049709	1.28E-03	2.14E-03	NA	1.70E-02

Gene	topSNP	p_eQTL	p_SMR	p_HEIDI	adjp_SMR
ARFGAP3	rs5758659	5.90E-04	2.03E-03	NA	1.70E-02
C8orf86	rs2016875	2.51E-04	1.87E-03	NA	1.70E-02
GRAP2	rs9306336	2.37E-04	2.29E-03	NA	1.70E-02
HIST1H4C	rs198811	4.56E-04	2.08E-03	NA	1.70E-02
LTA	rs2001097	1.34E-03	2.20E-03	NA	1.70E-02
RP11-73M18.6	rs10132641	5.71E-04	2.30E-03	NA	1.70E-02
ZKSCAN4	rs34130214	1.34E-03	2.19E-03	NA	1.70E-02
GABBR1	rs1611204	1.81E-03	3.88E-03	NA	1.70E-02
HLA-DOB	rs1003878	1.67E-03	3.40E-03	NA	1.70E-02
POLR3H	rs9611488	1.34E-03	4.68E-03	NA	1.70E-02
STK19	rs3132935	3.02E-03	4.69E-03	NA	1.70E-02
XRCC6	rs9611488	1.35E-03	4.70E-03	NA	1.70E-02
ALG1L13P	rs2948286	4.62E-05	2.66E-03	NA	1.72E-02
DDX39B-AS1	rs1265092	3.03E-04	2.45E-03	NA	1.72E-02
GPR146	rs56727870	1.18E-03	2.51E-03	NA	1.72E-02
KNTC1	rs1051434	7.12E-04	2.35E-03	NA	1.72E-02
LEPRE1	rs3001723	7.16E-04	2.50E-03	NA	1.72E-02
NCR3	rs3094605	1.73E-03	2.48E-03	NA	1.72E-02
BTN2A3P	rs6910549	1.89E-03	3.19E-03	NA	1.72E-02
HFE	rs34525648	1.88E-03	3.10E-03	NA	1.72E-02
RP11-73M18.7	rs3212024	3.98E-04	1.45E-03	NA	1.74E-02
PNOC	rs111659883	9.86E-04	3.61E-03	NA	1.74E-02
ROBO3	rs12283429	9.99E-04	3.70E-03	NA	1.76E-02
SCGN	rs9393716	2.48E-03	4.95E-03	NA	1.76E-02
PPP1R16B	rs12625702	7.99E-04	3.57E-03	NA	1.78E-02
RNF39	rs112863520	1.34E-03	3.77E-03	NA	1.78E-02
CYP17A1	rs17114641	6.72E-04	2.79E-03	NA	1.79E-02
F2	rs6485685	7.93E-04	2.82E-03	NA	1.79E-02
CTB-152G17.6	rs3801281	6.78E-04	2.77E-03	NA	1.79E-02
PSORS1C2	rs3132564	8.35E-04	2.48E-03	NA	1.79E-02
NRIP2	rs10774033	1.23E-03	2.81E-03	NA	1.80E-02
CEP170	rs14403	7.30E-04	2.83E-03	NA	1.81E-02
MEI1	rs9611469	5.70E-04	2.80E-03	NA	1.81E-02
NAPSB	rs7047	1.87E-03	5.18E-03	NA	1.81E-02
NDUFA6	rs5758268	1.39E-03	5.27E-03	NA	1.82E-02
RP1-313I6.12	rs9295759	2.62E-03	5.24E-03	NA	1.82E-02
EIF4BP6	rs7807853	5.72E-04	2.94E-03	NA	1.82E-02
CNTN4	rs9845120	5.36E-04	3.04E-03	NA	1.83E-02
LRTM2	rs11062157	5.50E-04	3.04E-03	NA	1.83E-02
C1orf86	rs10797426	8.55E-04	3.13E-03	NA	1.84E-02
UQCC2	rs2395401	7.76E-04	3.03E-03	0.92	1.84E-02
DHFRP2	rs1131165	1.54E-03	3.81E-03	NA	1.85E-02
ZBTB22	rs1048645	1.51E-03	3.81E-03	NA	1.85E-02

Gene	topSNP	p_eQTL	p_SMR	p_HEIDI	adjp_SMR
HIST1H4K	rs1005127	2.32E-03	4.07E-03	NA	1.85E-02
SLC39A7	rs2395401	1.23E-03	4.05E-03	NA	1.85E-02
PABPC1P7	rs13107325	3.21E-03	5.49E-03	NA	1.85E-02
CYP17A1-AS1	rs35525740	1.93E-03	4.11E-03	NA	1.86E-02
DNAH1	rs11717383	8.92E-04	4.14E-03	NA	1.86E-02
RP11-350N15.4	rs9198	1.74E-03	5.56E-03	NA	1.86E-02
CRY2	rs61882709	1.75E-03	4.19E-03	NA	1.86E-02
RP11-245J9.6	rs7627690	4.47E-04	2.56E-03	NA	1.88E-02
TEX264	rs7622851	9.12E-04	4.28E-03	NA	1.88E-02
RP11-664I21.6	rs12283429	1.32E-03	4.39E-03	NA	1.89E-02
ALDH16A1	rs7047	2.16E-03	5.71E-03	NA	1.90E-02
RP11-62H7.2	rs2948286	6.16E-05	2.91E-03	NA	1.90E-02
OR2H5P	rs1737082	1.43E-03	3.66E-03	NA	1.91E-02
L3MBTL2	rs5758268	6.39E-04	3.37E-03	NA	1.91E-02
SNRNP35	rs4148862	1.65E-03	4.50E-03	NA	1.92E-02
CTD-2574D22.2	rs4424923	1.36E-03	4.41E-03	NA	1.92E-02
SBNO1	rs7139321	1.70E-03	4.53E-03	NA	1.92E-02
POM121L2	rs9348746	1.86E-03	4.47E-03	NA	1.93E-02
RP11-12M9.3	rs12158335	1.03E-03	4.48E-03	NA	1.93E-02
RP11-214K3.21	rs655293	3.00E-04	1.73E-03	NA	1.93E-02
THYN1	rs11223651	1.53E-03	3.52E-03	NA	1.94E-02
NAB2	rs324015	1.06E-03	3.54E-03	NA	1.94E-02
FCGRT	rs57940349	9.87E-04	4.68E-03	NA	1.95E-02
CLIC1	rs2596473	1.99E-03	6.06E-03	NA	1.96E-02
GOLGA6L3	rs17600551	5.60E-04	3.42E-03	NA	1.97E-02
AC005076.5	rs73210240	1.38E-03	4.84E-03	NA	1.97E-02
FAM63A	rs72694955	1.11E-03	4.69E-03	NA	1.98E-02
HLA-DPB2	rs210142	1.28E-03	4.91E-03	NA	1.98E-02
ZSCAN16-AS1	rs34546986	3.40E-03	4.21E-03	NA	1.98E-02
KCNK7	rs1193851	3.41E-04	2.74E-03	NA	1.98E-02
CTB-134H23.2	rs4583255	1.39E-03	3.68E-03	NA	1.99E-02
RP11-68I18.10	rs72694955	7.50E-04	3.75E-03	NA	1.99E-02
TNF	rs9266797	8.32E-04	3.71E-03	NA	1.99E-02
WDR82	rs11235	8.97E-04	3.72E-03	NA	1.99E-02
HMGN4	rs3757150	4.60E-03	6.39E-03	NA	2.00E-02
TRIM35	rs9331950	5.70E-04	2.83E-03	NA	2.00E-02
STAT6	rs324015	1.23E-03	3.91E-03	NA	2.00E-02
PANX3	rs12283429	1.67E-03	5.10E-03	NA	2.01E-02
EXOC6B	rs11903916	1.83E-03	6.46E-03	NA	2.01E-02
CNTF	rs708228	1.46E-03	4.86E-03	NA	2.01E-02
RP11-350N15.5	rs9198	9.37E-04	3.81E-03	NA	2.03E-02
CTD-3065B20.3	rs6227	6.64E-04	2.99E-03	NA	2.03E-02
ZSCAN12	rs6456825	3.74E-03	6.56E-03	NA	2.03E-02

Gene	topSNP	p_eQTL	p_SMR	p_HEIDI	adjp_SMR
CHRM4	rs11038906	2.23E-03	4.98E-03	NA	2.04E-02
SCARA3	rs73229093	2.02E-03	5.01E-03	NA	2.04E-02
INPP5K	rs12603592	9.17E-04	4.17E-03	NA	2.06E-02
RP1-178F10.1	rs2955367	1.92E-04	3.94E-03	NA	2.06E-02
RP11-469M7.1	rs796364	2.51E-03	4.50E-03	NA	2.07E-02
RPL3P4	rs11624408	9.09E-04	4.29E-03	NA	2.08E-02
RP11-541N10.3	rs7911789	2.31E-03	4.56E-03	NA	2.08E-02
XPNPEP3	rs9611469	1.30E-03	4.59E-03	NA	2.08E-02
ADAMTS8	rs4936123	7.73E-04	4.40E-03	NA	2.09E-02
RP11-85F14.5	rs9845788	2.22E-03	6.87E-03	NA	2.09E-02
GTF3C3	rs11900232	3.23E-03	6.94E-03	NA	2.09E-02
NFKBIL1	rs113239947	1.02E-03	4.02E-03	NA	2.10E-02
RP11-47A8.5	rs10430665	3.26E-03	5.43E-03	NA	2.10E-02
STT3A	rs12541	1.05E-03	4.14E-03	NA	2.10E-02
ABHD14A	rs1060330	6.55E-05	1.14E-03	NA	2.11E-02
SMIM4	rs7629072	1.44E-03	5.51E-03	NA	2.11E-02
CTSK	rs72694955	5.96E-04	3.30E-03	NA	2.12E-02
SCARNA6	rs6710294	2.19E-03	4.53E-03	NA	2.12E-02
LST1	rs3130628	3.12E-03	4.58E-03	NA	2.13E-02
RORA	rs11071612	8.63E-04	4.23E-03	NA	2.13E-02
AC007238.1	rs1518394	1.40E-03	4.83E-03	NA	2.14E-02
RANP1	rs2233960	2.34E-03	7.12E-03	NA	2.14E-02
LINC00167	rs10791098	1.74E-03	4.68E-03	NA	2.14E-02
RP11-455F5.4	rs4583255	3.65E-03	7.32E-03	NA	2.16E-02
TRIM8	rs12573474	3.12E-03	7.33E-03	NA	2.16E-02
TTYH3	rs1476887	4.22E-03	7.38E-03	NA	2.16E-02
CTA-150C2.13	rs55898159	1.47E-03	5.14E-03	NA	2.17E-02
EMX1	rs11903916	1.21E-03	5.08E-03	NA	2.17E-02
HLA-A	rs1610721	2.58E-03	5.14E-03	NA	2.17E-02
MUSTN1	rs11235	1.49E-03	5.06E-03	NA	2.17E-02
SCARNA5	rs11535	2.51E-03	4.98E-03	NA	2.17E-02
PPP1R2P1	rs2395401	1.52E-03	4.64E-03	NA	2.19E-02
NFAM1	rs5758645	2.01E-03	5.23E-03	NA	2.20E-02
CTA-14H9.5	rs13204572	2.61E-03	3.49E-03	NA	2.22E-02
HIST2H3PS2	rs7555080	1.26E-03	4.91E-03	NA	2.22E-02
ZNF764	rs4424923	1.61E-03	4.91E-03	NA	2.22E-02
TAP2	rs3129950	4.05E-03	5.37E-03	NA	2.23E-02
RP11-671M22.4	rs2135551	8.96E-04	4.38E-03	NA	2.23E-02
RP11-365N19.2	rs12891288	7.21E-04	4.53E-03	NA	2.24E-02
PNMA2	rs3808573	1.48E-03	5.95E-03	NA	2.24E-02
RPS18	rs1130126	3.05E-03	5.97E-03	NA	2.24E-02
AKT3	rs3006916	1.06E-03	4.96E-03	NA	2.24E-02
RP11-1246C19.1	rs4719431	4.67E-03	7.79E-03	NA	2.24E-02

Gene	topSNP	p_eQTL	p_SMR	p_HEIDI	adjp_SMR
SLC43A1	rs708228	3.02E-03	7.77E-03	NA	2.24E-02
BAG5	rs12891288	7.45E-04	4.61E-03	NA	2.26E-02
LTB	rs1264318	1.26E-03	2.39E-03	NA	2.26E-02
EIF2B4	rs12474906	9.73E-04	5.17E-03	NA	2.27E-02
SOX2-OT	rs1871355	1.21E-03	5.13E-03	NA	2.27E-02
PRRC2A	rs2736176	1.49E-03	3.50E-03	NA	2.28E-02
AKT1	rs2223937	2.27E-03	5.26E-03	NA	2.28E-02
CTA-254O6.1	rs56936192	1.37E-03	5.33E-03	NA	2.28E-02
CUTA	rs8084	2.19E-03	5.35E-03	NA	2.28E-02
ELFN1	rs6945719	2.30E-03	4.88E-03	NA	2.28E-02
RP11-272L13.3	rs4506495	2.44E-03	4.85E-03	NA	2.28E-02
ARNT	rs72694957	2.08E-03	6.04E-03	NA	2.28E-02
SH2B3	rs12305489	4.11E-04	2.48E-03	NA	2.29E-02
C10orf95	rs284856	2.44E-04	2.58E-03	NA	2.29E-02
RP11-697E2.4	rs4702	1.37E-03	3.62E-03	NA	2.31E-02
GABARAPL3	rs4932178	1.21E-03	4.77E-03	NA	2.31E-02
RP11-547D24.1	rs2494631	2.39E-03	6.33E-03	NA	2.32E-02
ACTG2	rs11903916	1.39E-03	5.51E-03	NA	2.32E-02
TBC1D5	rs2033379	1.46E-03	5.49E-03	NA	2.32E-02
TMEM219	rs4788211	1.47E-03	5.40E-03	NA	2.32E-02
TMEM240	rs10797426	2.04E-03	5.49E-03	NA	2.32E-02
GLS2	rs324017	1.74E-03	4.94E-03	NA	2.32E-02
PSMG3	rs4721302	1.51E-03	5.04E-03	NA	2.33E-02
RP11-752G15.4	rs4779046	2.79E-03	8.19E-03	NA	2.33E-02
RP11-383B4.4	rs2148182	1.53E-03	5.71E-03	NA	2.33E-02
C10orf76	rs10883798	3.35E-03	5.77E-03	NA	2.33E-02
DHX16	rs3131787	4.01E-03	5.81E-03	NA	2.33E-02
HCG9	rs1063320	1.49E-03	5.90E-03	NA	2.33E-02
MUC21	rs2596473	1.92E-03	5.92E-03	NA	2.33E-02
RP11-839D17.3	rs17602038	2.11E-03	5.74E-03	NA	2.33E-02
RPL5P34	rs6002597	1.92E-03	5.89E-03	NA	2.33E-02
XXbac-BPG308K3.5	rs34546986	4.93E-03	5.93E-03	NA	2.33E-02
MIB2	rs16824398	2.20E-04	1.60E-03	0.95	2.33E-02
UNCX	rs56727870	7.52E-04	1.78E-03	NA	2.33E-02
ZNF165	rs7750526	1.06E-03	1.99E-03	NA	2.33E-02
ZNF322	rs13217675	2.67E-04	1.68E-03	0.46	2.33E-02
DUSP11	rs73947808	2.89E-03	8.34E-03	NA	2.34E-02
TCHP	rs3026445	2.95E-03	8.35E-03	NA	2.34E-02
AC136289.1	rs832195	6.57E-04	2.89E-03	NA	2.35E-02
CDK11A	rs942820	9.38E-04	3.39E-03	NA	2.35E-02
LSM2	rs7195	1.19E-03	3.45E-03	NA	2.35E-02
ADAMTSL4	rs72694955	7.24E-04	3.68E-03	NA	2.37E-02
RP11-629G13.1	rs73001688	2.12E-03	6.77E-03	NA	2.37E-02

Gene	topSNP	p_eQTL	p_SMR	p_HEIDI	adjp_SMR
C2orf69	rs3769456	2.82E-03	6.83E-03	NA	2.37E-02
PRR3	rs3095153	7.00E-03	8.68E-03	NA	2.41E-02
ANKK1	rs10750025	2.68E-03	7.04E-03	NA	2.41E-02
RP13-608F4.1	rs4779046	3.17E-03	8.88E-03	NA	2.42E-02
HLA-DRA	rs3130628	4.53E-03	6.29E-03	NA	2.43E-02
CDH3	rs1971546	1.72E-03	6.63E-03	NA	2.44E-02
NDUFA4L2	rs324017	2.52E-03	6.33E-03	NA	2.45E-02
MOG	rs1059535	5.88E-03	9.01E-03	NA	2.45E-02
SHISA8	rs5758268	2.33E-03	7.19E-03	NA	2.45E-02
TNXB	rs7195	2.62E-03	5.96E-03	NA	2.46E-02
NCAM1-AS1	rs10736470	2.40E-03	6.53E-03	NA	2.47E-02
CTD-3064M3.7	rs11778057	5.23E-03	9.17E-03	NA	2.48E-02
TTC34	rs942820	2.04E-03	5.59E-03	NA	2.48E-02
SIAE	rs12541	3.76E-03	9.25E-03	NA	2.49E-02
B3GALT4	rs487835	9.20E-04	3.89E-03	NA	2.50E-02
TNKS	rs73191548	4.15E-04	3.92E-03	NA	2.50E-02
EFTUD1	rs1269134	2.32E-03	6.97E-03	NA	2.50E-02
RP4-756G23.5	rs1894713	3.02E-03	6.97E-03	NA	2.50E-02
DRG2	rs9907287	1.45E-03	7.43E-03	NA	2.50E-02
RP11-153M3.1	rs324017	3.17E-03	7.41E-03	NA	2.50E-02
MAFK	rs56727870	4.51E-03	7.18E-03	NA	2.52E-02
ENKD1	rs7187202	3.10E-03	9.53E-03	NA	2.53E-02
RANBP10	rs1971546	3.15E-03	9.50E-03	NA	2.53E-02
RP11-457M11.2	rs6910549	6.99E-03	9.55E-03	NA	2.53E-02
ZNF696	rs7833728	3.41E-03	9.53E-03	NA	2.53E-02
SNX32	rs1193851	2.25E-03	7.66E-03	NA	2.54E-02
TKT	rs1029871	1.80E-03	7.66E-03	NA	2.54E-02
ZNF311	rs1737082	4.00E-03	7.69E-03	NA	2.54E-02
HIST1H1E	rs13217675	2.61E-03	6.78E-03	NA	2.54E-02
ECEL1	rs6710294	4.47E-03	7.80E-03	NA	2.55E-02
PTGES3P4	rs35525740	4.16E-03	7.36E-03	NA	2.55E-02
COQ10B	rs3731570	3.70E-03	9.72E-03	NA	2.55E-02
DDR1	rs1265092	3.56E-03	9.84E-03	NA	2.55E-02
RP3-395M20.12	rs16824398	2.92E-03	7.53E-03	NA	2.57E-02
XXbac-BPG252P9.9	rs1264352	5.67E-03	7.57E-03	NA	2.57E-02
CYB5R3	rs134889	4.95E-03	9.95E-03	NA	2.57E-02
C15orf40	rs4779046	8.76E-04	4.12E-03	NA	2.58E-02
CACNA1D	rs11177	1.60E-03	7.63E-03	NA	2.58E-02
RP11-168J18.6	rs678	1.72E-03	7.08E-03	NA	2.59E-02
RYBP	rs9837383	2.12E-03	7.12E-03	NA	2.59E-02
ARL6IP4	rs7304782	6.35E-03	1.01E-02	NA	2.59E-02
FKBP4	rs1016388	3.92E-03	6.72E-03	NA	2.59E-02
CROT	rs73210260	2.33E-03	7.18E-03	NA	2.60E-02

Gene	topSNP	p_eQTL	p_SMR	p_HEIDI	adjp_SMR
GLB1L3	rs73034295	3.97E-03	7.25E-03	NA	2.60E-02
INHBE	rs324015	3.01E-03	7.09E-03	NA	2.60E-02
ENO1-AS1	rs301792	1.90E-03	6.78E-03	NA	2.60E-02
NDUFS3	rs10838634	4.98E-03	1.04E-02	NA	2.60E-02
RSRC2	rs10846491	4.49E-03	1.03E-02	NA	2.60E-02
FGF8	rs11191359	1.37E-03	4.39E-03	NA	2.60E-02
MARS2	rs1429417	2.45E-03	6.27E-03	NA	2.61E-02
DDX55	rs4275659	3.83E-03	8.04E-03	NA	2.61E-02
LRRN3	rs37716	4.30E-03	1.06E-02	NA	2.62E-02
LSM1	rs2306899	1.77E-03	6.46E-03	NA	2.63E-02
RP11-527N22.2	rs2016875	2.22E-03	6.62E-03	NA	2.63E-02
TEKT5	rs7195942	9.88E-04	6.42E-03	NA	2.63E-02
RP11-512M8.3	rs4148862	3.73E-03	7.93E-03	NA	2.64E-02
TM6SF1	rs1269134	2.82E-03	7.91E-03	NA	2.64E-02
TAF5	rs10883842	3.91E-03	6.98E-03	NA	2.64E-02
GLYCTK-AS1	rs11235	1.13E-03	4.28E-03	NA	2.64E-02
CRTC3	rs4702	4.45E-03	8.43E-03	NA	2.66E-02
RP11-197N18.7	rs655293	3.71E-03	8.45E-03	NA	2.66E-02
AMBRA1	rs10838634	3.48E-03	8.07E-03	NA	2.66E-02
RP4-740C4.6	rs942820	3.53E-03	8.10E-03	NA	2.66E-02
ACP2	rs12576115	4.36E-03	8.30E-03	NA	2.67E-02
CTD-2145A24.3	rs9907287	1.91E-03	8.60E-03	NA	2.68E-02
ORC2	rs3769446	2.81E-03	8.76E-03	NA	2.70E-02
RILPL2	rs11057238	5.10E-03	1.11E-02	NA	2.70E-02
CLUH	rs9893573	2.34E-03	7.70E-03	NA	2.71E-02
CCDC25	rs73229090	2.03E-03	5.29E-03	NA	2.71E-02
OPCML	rs11223651	2.59E-03	5.19E-03	NA	2.71E-02
RP5-1115A15.1	rs159962	1.46E-03	5.24E-03	NA	2.71E-02
1-Sep	rs4424923	3.68E-03	8.50E-03	NA	2.71E-02
HECW2	rs3731570	9.86E-04	4.36E-03	NA	2.72E-02
GABPB2	rs72694965	3.75E-03	8.95E-03	NA	2.72E-02
RP11-390K5.6	rs10838634	4.08E-03	9.01E-03	NA	2.72E-02
BRINP2	rs4650963	4.36E-04	2.75E-03	NA	2.73E-02
DESI1	rs9607850	4.07E-04	2.78E-03	NA	2.73E-02
MGAT3	rs9306336	2.77E-03	8.68E-03	NA	2.73E-02
SLC39A12-AS1	rs2148182	3.05E-03	8.69E-03	NA	2.73E-02
CYP11B1	rs4976982	2.54E-03	4.74E-03	NA	2.74E-02
HIST2H3D	rs72694955	1.10E-03	4.66E-03	NA	2.74E-02
RP3-375P9.2	rs942496	1.20E-03	4.62E-03	NA	2.74E-02
TLX2	rs11903916	3.24E-03	9.17E-03	NA	2.75E-02
ZBTB12	rs1046089	6.11E-03	9.28E-03	NA	2.75E-02
PCGF6	rs10786701	4.54E-03	9.56E-03	NA	2.75E-02
RPL29	rs678	2.89E-03	9.54E-03	NA	2.75E-02

Gene	topSNP	p_eQTL	p_SMR	p_HEIDI	adjp_SMR
Y_RNA	rs10838634	4.45E-03	9.58E-03	NA	2.75E-02
LLGL1	rs9907287	8.07E-04	5.56E-03	NA	2.75E-02
MMP23B	rs942820	1.86E-03	5.26E-03	NA	2.75E-02
SLC32A1	rs2244937	1.17E-03	5.17E-03	NA	2.75E-02
LFNG	rs3778970	4.89E-03	8.91E-03	NA	2.75E-02
NHP2L1	rs5758645	3.03E-03	6.94E-03	NA	2.75E-02
CERS2	rs72694957	5.41E-03	1.15E-02	NA	2.76E-02
FOXO3	rs4945816	2.24E-03	1.15E-02	NA	2.76E-02
SFXN2	rs284858	3.94E-03	1.14E-02	NA	2.76E-02
STMN4	rs2565065	5.38E-03	1.15E-02	NA	2.76E-02
FBXL15	rs10883798	3.46E-03	5.94E-03	NA	2.77E-02
C1orf51	rs72694965	3.80E-03	9.03E-03	NA	2.77E-02
C8orf31	rs11778057	4.48E-03	8.14E-03	NA	2.78E-02
ITIH3	rs6769789	3.59E-03	8.20E-03	NA	2.78E-02
PRC1	rs35346340	3.10E-03	8.12E-03	NA	2.78E-02
RP3-508I15.19	rs9306336	2.50E-03	8.18E-03	NA	2.78E-02
RP1-153G14.4	rs13217675	3.07E-03	7.55E-03	NA	2.79E-02
HIST1H2BE	rs7771468	7.57E-04	3.43E-03	NA	2.80E-02
EIF4E2	rs11535	4.02E-03	7.13E-03	NA	2.81E-02
NEU1	rs5021453	6.37E-03	9.95E-03	NA	2.82E-02
EFHD1	rs6710294	5.71E-03	9.44E-03	NA	2.83E-02
PBX2	rs7195	5.09E-03	9.63E-03	NA	2.85E-02
PTP4A3	rs72687376	3.72E-03	9.65E-03	NA	2.85E-02
HIST1H3D	rs77006546	5.43E-03	8.71E-03	NA	2.85E-02
RINT1	rs6968335	5.05E-03	1.20E-02	NA	2.86E-02
RANGAP1	rs9607850	4.99E-03	1.20E-02	NA	2.86E-02
TUBB	rs3130618	6.73E-03	8.83E-03	NA	2.86E-02
RP11-196G18.3	rs11589922	2.79E-03	7.85E-03	NA	2.87E-02
EPHX2	rs73229093	6.87E-03	1.21E-02	NA	2.87E-02
HIST2H2BC	rs72694965	5.86E-03	1.22E-02	NA	2.87E-02
RTKN	rs73947808	2.48E-03	7.57E-03	NA	2.87E-02
ABR	rs12603592	2.83E-03	8.18E-03	NA	2.89E-02
PHF1	rs5021453	5.03E-03	8.24E-03	NA	2.89E-02
TAPSAR1	rs204994	5.85E-03	8.18E-03	NA	2.89E-02
CTC-498M16.4	rs62378245	5.53E-03	1.03E-02	NA	2.89E-02
RAB27B	rs1011339	4.13E-03	1.03E-02	NA	2.89E-02
U91328.20	rs34525648	5.52E-03	7.68E-03	NA	2.89E-02
SMCR2	rs2955365	2.01E-03	9.15E-03	NA	2.89E-02
AC009061.1	rs1971546	2.60E-03	8.46E-03	NA	2.89E-02
RN7SKP80	rs28579115	3.11E-03	8.48E-03	NA	2.89E-02
RAB11FIP1	rs60530611	2.96E-03	9.53E-03	NA	2.89E-02
RP11-214K3.20	rs7304782	5.96E-03	9.65E-03	NA	2.89E-02
SBP1	rs942496	4.06E-03	9.91E-03	NA	2.89E-02

Gene	topSNP	p_eQTL	p_SMR	p_HEIDI	adjp_SMR
TRIM38	rs6902389	8.08E-03	9.96E-03	NA	2.89E-02
ATF4	rs9306336	3.52E-03	1.01E-02	NA	2.90E-02
MRPL33	rs4666014	2.16E-03	1.00E-02	NA	2.90E-02
TPRKB	rs73947808	1.30E-03	5.14E-03	NA	2.90E-02
ASPG	rs10132641	3.62E-03	7.83E-03	NA	2.90E-02
AC091167.3	rs4702	4.14E-03	7.98E-03	NA	2.91E-02
ADAM32	rs2411256	3.09E-03	7.94E-03	NA	2.91E-02
CDC42BPB	rs34633710	3.04E-03	8.20E-03	NA	2.91E-02
LY6K	rs7833728	2.68E-03	8.21E-03	NA	2.91E-02
HIST1H4H	rs6456728	6.48E-03	8.67E-03	NA	2.91E-02
BZW1	rs3769446	3.76E-03	1.05E-02	NA	2.91E-02
MVP	rs4424923	5.06E-03	1.06E-02	NA	2.92E-02
DDX43	rs1548335	4.49E-03	1.08E-02	NA	2.93E-02
RP11-44N22.3	rs2077586	4.08E-03	1.08E-02	NA	2.93E-02
WBP1L	rs79237883	7.51E-03	1.08E-02	NA	2.93E-02
NELFE	rs9501045	1.43E-03	5.88E-03	NA	2.93E-02
ENO1	rs159962	3.35E-03	8.79E-03	NA	2.93E-02
APOBEC3G	rs55898159	4.35E-03	1.03E-02	NA	2.93E-02
CGREF1	rs12474906	3.64E-03	1.09E-02	NA	2.94E-02
AC102953.4	rs1078112	5.43E-03	1.04E-02	NA	2.94E-02
CD34	rs12749612	2.77E-03	1.04E-02	NA	2.94E-02
FAM53C	rs55985421	3.23E-03	7.91E-03	NA	2.94E-02
BOLA2	rs4424923	5.30E-03	1.10E-02	NA	2.94E-02
RP11-499P20.2	rs4748465	4.73E-03	1.07E-02	NA	2.94E-02
TTI1	rs208818	4.91E-03	9.97E-03	NA	2.94E-02
ZFAND2A	rs3778998	6.96E-03	1.27E-02	NA	2.95E-02
PFDN6	rs1318691	4.41E-03	1.01E-02	NA	2.96E-02
TRIM39	rs3129984	4.66E-03	6.12E-03	NA	2.96E-02
CTD-2171N6.1	rs9964176	2.90E-03	8.12E-03	NA	2.96E-02
TACC1	rs17435276	4.85E-04	4.21E-03	NA	2.97E-02
CTA-989H11.1	rs133347	3.92E-03	8.90E-03	NA	2.97E-02
ESRP2	rs1971546	3.96E-03	1.09E-02	NA	2.97E-02
TP53TG1	rs13236006	4.62E-03	1.12E-02	NA	2.98E-02
TARS2	rs72694965	5.14E-03	1.11E-02	NA	2.99E-02
ADAMTSL3	rs2135551	1.92E-03	6.77E-03	NA	3.01E-02
CTD-2292P10.4	rs4976982	4.24E-03	7.09E-03	NA	3.01E-02
OR2B4P	rs1416920	4.90E-03	7.06E-03	NA	3.01E-02
PITPNA-AS1	rs12603592	2.20E-03	7.00E-03	NA	3.01E-02
TNNC1	rs4687680	2.83E-03	6.85E-03	NA	3.01E-02
GPLD1	rs77006546	7.54E-03	1.14E-02	NA	3.01E-02
TMEM161A	rs880090	9.43E-04	4.44E-03	NA	3.02E-02
HIST1H2BN	rs1150670	8.67E-03	1.33E-02	NA	3.02E-02
AC005841.1	rs1016388	3.66E-03	6.36E-03	NA	3.02E-02

Gene	topSNP	p_eQTL	p_SMR	p_HEIDI	adjp_SMR
TWF2	rs7626551	2.67E-03	6.74E-03	NA	3.02E-02
BRK1	rs61432137	4.27E-03	1.13E-02	NA	3.02E-02
ROBO4	rs12541	4.59E-03	1.06E-02	NA	3.02E-02
TBC1D28	rs9907287	3.25E-03	1.15E-02	NA	3.03E-02
RP11-397A16.2	rs784255	5.84E-03	1.06E-02	NA	3.03E-02
MOB4	rs7582536	6.68E-03	1.16E-02	NA	3.04E-02
KIAA1755	rs6093098	9.04E-03	1.34E-02	NA	3.05E-02
QPRT	rs4424923	5.90E-03	1.18E-02	NA	3.05E-02
SATB2-AS1	rs3769456	3.84E-03	8.46E-03	NA	3.05E-02
CTB-33G10.1	rs57940349	4.13E-03	1.09E-02	NA	3.07E-02
FURIN	rs4702	6.31E-03	1.10E-02	NA	3.07E-02
ARHGEF25	rs73115999	1.72E-03	6.99E-03	NA	3.07E-02
LPPR5	rs61786697	3.25E-03	7.10E-03	NA	3.07E-02
AP000662.9	rs708228	6.86E-03	1.36E-02	NA	3.07E-02
HCAR1	rs7304782	7.88E-03	1.21E-02	NA	3.08E-02
MAZ	rs4788211	2.42E-03	7.30E-03	NA	3.09E-02
GTF2H3	rs655293	5.62E-03	1.13E-02	NA	3.09E-02
RP11-390K5.1	rs7476	5.27E-03	1.13E-02	NA	3.09E-02
ZFP57	rs3131336	9.83E-03	1.12E-02	NA	3.09E-02
ANAPC7	rs12305489	5.31E-03	1.18E-02	NA	3.09E-02
TOB2	rs5758645	3.48E-03	7.66E-03	NA	3.09E-02
ORC5	rs41562	6.16E-03	1.21E-02	NA	3.09E-02
SEC63P1	rs61786697	4.91E-03	9.56E-03	NA	3.10E-02
TAPBP	rs1142323	5.90E-03	9.57E-03	NA	3.10E-02
JAM3	rs11223651	7.85E-03	1.22E-02	NA	3.10E-02
SUMO2P1	rs3116856	9.49E-03	1.38E-02	NA	3.11E-02
HIST1H4E	rs6902389	6.93E-03	8.68E-03	NA	3.11E-02
ITFG2	rs2238053	5.43E-03	8.73E-03	NA	3.11E-02
SH3GL3	rs950169	3.88E-03	9.63E-03	NA	3.11E-02
SRSF4	rs1498231	6.36E-03	1.39E-02	NA	3.11E-02
NDUFB3	rs3769456	4.51E-03	9.47E-03	NA	3.12E-02
RP11-659P15.1	rs708228	4.22E-03	9.72E-03	NA	3.12E-02
GPR62	rs13072536	1.10E-03	4.98E-03	NA	3.12E-02
RP11-243A14.1	rs7195942	5.80E-04	5.00E-03	NA	3.12E-02
GALNT10	rs11740474	3.85E-03	1.29E-02	NA	3.13E-02
HSD11B2	rs1971546	5.15E-03	1.29E-02	NA	3.13E-02
NKAPL	rs2747054	1.05E-02	1.25E-02	NA	3.13E-02
NT5DC2	rs11717383	5.73E-03	1.30E-02	NA	3.13E-02
SLC25A17	rs9611469	5.41E-03	1.16E-02	NA	3.13E-02
RP11-620J15.3	rs324015	4.68E-03	9.66E-03	NA	3.13E-02
PPRC1	rs10786736	3.97E-03	7.93E-03	NA	3.13E-02
RP11-278H7.1	rs10803138	2.12E-03	6.25E-03	NA	3.14E-02
RP11-677M14.3	rs12541	2.07E-03	6.28E-03	NA	3.14E-02

Gene	topSNP	p_eQTL	p_SMR	p_HEIDI	adjp_SMR
LINC00638	rs4906379	5.58E-03	9.91E-03	NA	3.14E-02
RP11-225H22.4	rs10883842	3.14E-03	5.91E-03	NA	3.15E-02
RP11-416N2.4	rs10786701	2.24E-03	5.88E-03	NA	3.15E-02
ATAD3A	rs942820	7.69E-03	1.41E-02	NA	3.15E-02
RP5-1186N24.3	rs1416920	7.40E-03	1.00E-02	NA	3.15E-02
NEURL	rs11191356	6.08E-03	1.26E-02	NA	3.15E-02
NABP2	rs324017	3.57E-03	8.04E-03	NA	3.16E-02
FBXW4	rs11191356	6.13E-03	1.26E-02	NA	3.16E-02
INF2	rs2223937	6.85E-03	1.18E-02	NA	3.16E-02
RXRB	rs511515	1.84E-03	6.54E-03	NA	3.16E-02
CAD	rs4666014	3.49E-03	1.29E-02	NA	3.18E-02
RP5-998N21.4	rs72694955	5.65E-03	1.29E-02	NA	3.18E-02
RING1	rs2001097	7.20E-03	9.30E-03	NA	3.18E-02
RP11-150O12.1	rs2306899	3.17E-03	9.18E-03	NA	3.18E-02
HLA-E	rs9266209	7.57E-03	1.20E-02	NA	3.19E-02
AC068831.10	rs4702	4.68E-03	8.74E-03	NA	3.19E-02
KXD1	rs1054930	4.27E-03	8.61E-03	NA	3.19E-02
NFKB2	rs284856	2.30E-03	8.27E-03	NA	3.19E-02
TMED2	rs1790094	4.27E-03	8.58E-03	NA	3.19E-02
SRPK2	rs4580973	3.73E-03	1.01E-02	NA	3.19E-02
RPL41	rs324015	3.63E-03	8.07E-03	NA	3.20E-02
GOLGA6L4	rs62021208	3.37E-03	9.74E-03	NA	3.23E-02
AP001007.1	rs12283429	5.02E-03	1.07E-02	NA	3.23E-02
WNT5B	rs1016388	5.60E-03	8.92E-03	NA	3.23E-02
TCTN1	rs12305489	1.49E-03	5.24E-03	NA	3.24E-02
CACNA2D4	rs11062157	4.34E-03	1.06E-02	NA	3.24E-02
NCAM1	rs4245150	5.14E-03	1.06E-02	NA	3.24E-02
RP11-981G7.1	rs73191548	2.96E-03	1.06E-02	NA	3.24E-02
CCDC134	rs6002597	2.30E-03	6.62E-03	NA	3.24E-02
MAU2	rs8101938	1.88E-03	6.58E-03	NA	3.24E-02
TTC14	rs1871355	1.85E-03	6.57E-03	NA	3.24E-02
AC087163.2	rs4584886	2.91E-03	1.07E-02	NA	3.24E-02
DPH1	rs2224770	1.27E-03	5.31E-03	NA	3.24E-02
HIST1H2AG	rs3757150	6.83E-03	9.01E-03	NA	3.25E-02
GTPBP1	rs55898159	7.57E-03	1.50E-02	NA	3.25E-02
TOMM22	rs9306336	6.48E-03	1.48E-02	NA	3.25E-02
AC009093.1	rs4788211	4.50E-03	1.09E-02	NA	3.26E-02
LY6G6D	rs2858310	2.45E-03	6.75E-03	NA	3.27E-02
CCDC62	rs1790094	9.17E-03	1.52E-02	NA	3.27E-02
LINC00637	rs11623546	1.02E-02	1.53E-02	NA	3.27E-02
PGAP1	rs1429417	4.87E-03	1.01E-02	NA	3.28E-02
RP11-260M19.2	rs10132641	5.69E-03	1.08E-02	NA	3.30E-02
SLC45A4	rs11778057	6.55E-03	1.09E-02	NA	3.30E-02

Gene	topSNP	p_eQTL	p_SMR	p_HEIDI	adjp_SMR
ERRFI1	rs302719	3.29E-03	8.79E-03	NA	3.31E-02
AC007092.1	rs11688767	6.17E-03	1.12E-02	NA	3.31E-02
SERHL2	rs1047997	5.78E-03	1.11E-02	NA	3.31E-02
AC004840.9	rs3800869	2.10E-03	7.33E-03	NA	3.32E-02
EIF2B1	rs941306	2.84E-03	7.40E-03	NA	3.32E-02
RP11-981G7.6	rs73191548	1.44E-03	7.21E-03	NA	3.32E-02
RP1-46F2.3	rs12305489	2.58E-03	7.36E-03	NA	3.32E-02
CTD-2210P24.4	rs7476	7.42E-03	1.44E-02	NA	3.33E-02
NGEF	rs6710294	9.69E-03	1.44E-02	NA	3.33E-02
RPL3	rs55898159	7.12E-03	1.44E-02	NA	3.33E-02
ZNF826P	rs880090	6.47E-03	1.44E-02	NA	3.33E-02
RP1-34B20.4	rs28551159	7.79E-03	9.78E-03	NA	3.34E-02
VRK2	rs1518394	4.11E-03	9.68E-03	NA	3.34E-02
LY6D	rs7462788	6.34E-03	1.05E-02	NA	3.34E-02
METTL21B	rs73115999	3.49E-03	1.05E-02	NA	3.34E-02
ALS2CR12	rs3769446	5.31E-03	1.30E-02	NA	3.34E-02
TRMT61A	rs11623546	8.37E-03	1.30E-02	NA	3.34E-02
AP3B2	rs1145173	4.59E-03	1.06E-02	NA	3.34E-02
CRK	rs9893573	4.42E-03	1.14E-02	NA	3.35E-02
RN7SL417P	rs11631921	3.61E-03	1.15E-02	NA	3.35E-02
SNX8	rs3800869	4.47E-03	1.16E-02	NA	3.35E-02
MIR22HG	rs9893573	5.99E-03	1.39E-02	NA	3.36E-02
RFXANK	rs2315022	6.37E-03	1.39E-02	NA	3.36E-02
RP11-430H10.2	rs11038864	5.85E-03	1.18E-02	NA	3.36E-02
DXO	rs9262696	4.66E-03	1.14E-02	NA	3.37E-02
HIST1H2BG	rs4711107	7.14E-03	1.08E-02	NA	3.37E-02
ANKRD44	rs12693816	5.99E-03	1.17E-02	NA	3.37E-02
C14orf144	rs35498576	3.96E-03	7.67E-03	NA	3.37E-02
SEPHS2	rs4583255	3.91E-03	7.70E-03	NA	3.37E-02
ARRDC3	rs2247870	1.33E-03	8.91E-03	NA	3.37E-02
CEP57L1	rs4945816	1.48E-03	9.46E-03	NA	3.38E-02
VPS33A	rs4553407	4.50E-03	9.37E-03	NA	3.38E-02
HIST1H2BH	rs34525648	1.11E-02	1.40E-02	NA	3.38E-02
CENPE	rs13107325	1.10E-02	1.51E-02	NA	3.38E-02
RP11-102M11.1	rs9845788	7.33E-03	1.50E-02	NA	3.38E-02
MATR3	rs55985421	5.13E-03	1.09E-02	NA	3.38E-02
RP11-486F17.1	rs59695400	5.97E-03	1.20E-02	NA	3.39E-02
KHK	rs4666014	2.63E-03	1.11E-02	NA	3.39E-02
RP11-1167A19.6	rs1193851	4.13E-03	1.11E-02	NA	3.39E-02
ZBTB9	rs1130126	6.90E-03	1.12E-02	NA	3.39E-02
CDC42SE1	rs72694957	2.05E-03	5.99E-03	NA	3.39E-02
DDX49	rs880090	1.73E-03	6.29E-03	NA	3.39E-02
PPP1R10	rs3094125	4.46E-03	5.92E-03	NA	3.39E-02

Gene	topSNP	p_eQTL	p_SMR	p_HEIDI	adjp_SMR
U91328.22	rs3734528	4.22E-03	5.95E-03	NA	3.39E-02
DENR	rs10846491	8.43E-03	1.60E-02	NA	3.40E-02
CASP8	rs3769446	7.47E-03	1.63E-02	NA	3.40E-02
DMTF1	rs76349903	8.18E-03	1.63E-02	NA	3.40E-02
LINC00051	rs72687376	8.15E-03	1.63E-02	NA	3.40E-02
HIST1H2BJ	rs6940638	1.01E-02	1.42E-02	NA	3.40E-02
FAM216A	rs11065628	4.77E-03	1.20E-02	NA	3.41E-02
GIT2	rs12305489	5.44E-03	1.20E-02	NA	3.41E-02
TRPM4	rs57940349	7.01E-03	1.54E-02	NA	3.42E-02
ALMS1-IT1	rs73947808	5.29E-03	1.23E-02	NA	3.43E-02
CKAP5	rs10838634	6.31E-03	1.23E-02	NA	3.43E-02
LINC00533	rs7764642	1.08E-02	1.23E-02	NA	3.43E-02
RP11-253E3.1	rs11062157	5.16E-03	1.18E-02	NA	3.44E-02
ADAM9	rs60530611	2.26E-03	8.15E-03	NA	3.44E-02
FAM83D	rs2263750	4.55E-03	8.72E-03	NA	3.44E-02
METTL16	rs216221	3.25E-03	8.44E-03	NA	3.44E-02
NISCH	rs7626551	3.47E-03	8.07E-03	NA	3.44E-02
RP11-154H23.3	rs7610856	2.77E-03	8.57E-03	NA	3.44E-02
TUSC5	rs9893573	2.93E-03	8.84E-03	NA	3.44E-02
HIST2H2AC	rs11589922	5.65E-03	1.25E-02	NA	3.44E-02
CALHM2	rs79237883	9.10E-03	1.27E-02	NA	3.44E-02
CS	rs324017	8.18E-03	1.46E-02	NA	3.44E-02
C12orf65	rs585522	8.12E-03	1.49E-02	NA	3.45E-02
MICF	rs1610645	9.81E-03	1.49E-02	NA	3.45E-02
PRKCZ	rs16824398	6.17E-03	1.26E-02	NA	3.46E-02
ACRV1	rs12283429	4.58E-03	9.99E-03	NA	3.46E-02
AC018766.5	rs7047	3.18E-03	7.43E-03	NA	3.46E-02
CTB-12O2.1	rs11167584	3.47E-03	8.37E-03	NA	3.46E-02
PLCH2	rs942820	3.87E-03	8.63E-03	NA	3.46E-02
PROK2	rs7610856	2.76E-03	8.57E-03	NA	3.46E-02
PSMB9	rs1142323	4.55E-03	7.81E-03	NA	3.46E-02
RP11-264B17.4	rs4583255	4.51E-03	8.56E-03	NA	3.46E-02
SAPCD1	rs3130281	6.05E-03	8.11E-03	NA	3.46E-02
ARFGAP2	rs61882709	1.03E-02	1.58E-02	NA	3.46E-02
CHST1	rs61882709	1.03E-02	1.58E-02	NA	3.46E-02
RPL39P3	rs1548335	7.89E-03	1.58E-02	NA	3.46E-02
SEZ6L2	rs4424923	2.47E-03	6.50E-03	NA	3.47E-02
H6PD	rs159962	7.49E-03	1.51E-02	NA	3.48E-02
FAM65A	rs1971546	5.12E-03	1.29E-02	NA	3.48E-02
FZD3	rs9331950	6.28E-03	1.29E-02	NA	3.48E-02
GLTP	rs12305489	6.10E-03	1.30E-02	NA	3.48E-02
RP11-54C4.2	rs72801007	5.98E-03	1.29E-02	NA	3.48E-02
ATF4P4	rs17602038	1.01E-02	1.72E-02	NA	3.49E-02

Gene	topSNP	p_eQTL	p_SMR	p_HEIDI	adjp_SMR
HIST1H1D	rs4711107	4.01E-03	6.78E-03	NA	3.50E-02
TGM2	rs12625702	2.36E-03	6.89E-03	NA	3.50E-02
RP11-586K2.1	rs10955542	7.86E-03	1.61E-02	NA	3.50E-02
SPCS1	rs34017441	4.23E-03	1.29E-02	NA	3.50E-02
MEN1	rs1193851	4.85E-03	1.23E-02	NA	3.50E-02
RNU1-60P	rs35065479	5.23E-03	1.73E-02	NA	3.50E-02
LRRC25	rs8101938	5.75E-03	1.33E-02	NA	3.51E-02
RP11-261C10.7	rs3006916	6.15E-03	1.43E-02	NA	3.51E-02
B3GNT4	rs7957096	7.15E-03	1.24E-02	NA	3.52E-02
TMEM183AP1	rs1633081	7.98E-03	1.31E-02	NA	3.53E-02
AL512428.1	rs7771468	8.57E-03	1.63E-02	NA	3.54E-02
DGKI	rs1647181	8.69E-03	1.58E-02	NA	3.54E-02
RP11-58A17.3	rs324017	9.09E-03	1.58E-02	NA	3.54E-02
SUGP1	rs747050	1.00E-02	1.63E-02	NA	3.54E-02
UQCRFS1P1	rs5757754	8.31E-03	1.59E-02	NA	3.54E-02
SLC35E2	rs2494635	2.54E-03	7.12E-03	NA	3.54E-02
CTD-3064M3.4	rs72687376	4.64E-03	1.12E-02	NA	3.55E-02
SERHL	rs12158335	4.51E-03	1.11E-02	NA	3.55E-02
ARHGAP40	rs4812320	8.17E-03	1.36E-02	NA	3.55E-02
HIST1H2AE	rs28360595	1.07E-02	1.35E-02	NA	3.55E-02
MCFD2P1	rs9301	8.94E-03	1.33E-02	NA	3.55E-02
MMP23A	rs16824398	6.64E-03	1.32E-02	NA	3.55E-02
RP11-430H10.4	rs61882709	7.71E-03	1.26E-02	NA	3.55E-02
RP11-752G15.6	rs1269134	6.49E-03	1.37E-02	NA	3.55E-02
SEPHS1P6	rs11688168	9.20E-03	1.36E-02	NA	3.55E-02
PPP4C	rs4583255	8.08E-03	1.33E-02	NA	3.55E-02
GGNBP1	rs210142	3.65E-03	9.43E-03	NA	3.55E-02
RP5-1042K10.13	rs9611488	3.96E-03	9.41E-03	NA	3.55E-02
CCDC68	rs72936313	8.22E-03	1.37E-02	NA	3.55E-02
EHD1	rs1193851	5.72E-03	1.37E-02	NA	3.55E-02
YJEFN3	rs880090	5.97E-03	1.36E-02	NA	3.55E-02
RP11-338N10.2	rs159962	5.93E-03	1.29E-02	NA	3.55E-02
MRPS23	rs35065479	1.61E-03	9.55E-03	NA	3.55E-02
PTPMT1	rs10838634	5.88E-03	1.17E-02	NA	3.56E-02
TXNL1	rs72936329	6.13E-03	1.13E-02	NA	3.56E-02
RP11-159D12.2	rs35065479	3.92E-03	1.49E-02	NA	3.56E-02
FLCN	rs9890563	6.66E-03	1.77E-02	NA	3.57E-02
KLC1	rs3212090	8.21E-03	1.31E-02	NA	3.57E-02
SLC45A1	rs159962	6.11E-03	1.31E-02	NA	3.58E-02
HIST1H2AK	rs2275508	1.18E-02	1.71E-02	NA	3.58E-02
4-Sep	rs35065479	3.48E-03	1.40E-02	NA	3.58E-02
RASAL2-AS1	rs4650963	4.76E-03	1.14E-02	NA	3.59E-02
NCK1	rs9845788	9.59E-03	1.81E-02	NA	3.59E-02

Gene	topSNP	p_eQTL	p_SMR	p_HEIDI	adjp_SMR
FGFR1	rs16887244	7.93E-03	1.82E-02	NA	3.60E-02
CCDC15	rs36053597	6.56E-03	1.34E-02	NA	3.61E-02
HIST1H3H	rs13217675	7.71E-03	1.44E-02	NA	3.62E-02
ATXN7	rs13272	1.04E-02	1.84E-02	NA	3.62E-02
HIST1H1PS1	rs3208733	9.98E-03	1.85E-02	NA	3.62E-02
TRIM26BP	rs887465	1.48E-02	1.85E-02	NA	3.62E-02
STAR	rs60530611	6.36E-03	1.53E-02	NA	3.63E-02
HIST1H2BD	rs34525648	1.23E-02	1.54E-02	NA	3.63E-02
RASD1	rs9907287	2.28E-03	9.43E-03	NA	3.64E-02
NUDT1	rs1476887	1.35E-02	1.88E-02	NA	3.64E-02
ATAD3B	rs942820	5.99E-03	1.18E-02	NA	3.65E-02
TLR9	rs11717383	5.02E-03	1.19E-02	NA	3.65E-02
C19orf73	rs57940349	7.09E-03	1.55E-02	NA	3.65E-02
MIEF1	rs9306336	7.16E-03	1.58E-02	NA	3.65E-02
RP11-282O18.7	rs10846491	8.18E-03	1.57E-02	NA	3.65E-02
IP6K3	rs487835	9.28E-03	1.74E-02	NA	3.65E-02
ADRA1A	rs9331950	8.48E-03	1.60E-02	NA	3.66E-02
FAM13B	rs188731	5.34E-03	1.25E-02	NA	3.66E-02
LINC01023	rs4388249	3.90E-03	1.25E-02	NA	3.66E-02
TMEM52	rs942820	6.56E-03	1.26E-02	NA	3.66E-02
CALHM1	rs79237883	1.08E-02	1.46E-02	NA	3.66E-02
POLR3G	rs2247870	3.38E-03	1.40E-02	NA	3.67E-02
NUBP1	rs7196708	8.66E-03	1.91E-02	NA	3.67E-02
CNOT8	rs11740474	4.99E-03	1.49E-02	NA	3.67E-02
FOXM1	rs11062170	1.12E-02	1.48E-02	NA	3.67E-02
RP11-977G19.11	rs324015	7.82E-03	1.40E-02	NA	3.68E-02
KIFC1	rs511515	6.33E-03	1.42E-02	NA	3.68E-02
STAG1	rs7349597	7.42E-03	1.41E-02	NA	3.68E-02
RP11-381K20.5	rs187653	7.47E-03	1.53E-02	NA	3.68E-02
AC145123.2	rs7462788	1.23E-02	1.78E-02	NA	3.69E-02
APLP2	rs62621284	6.64E-03	1.80E-02	NA	3.69E-02
EGFL8	rs1265754	1.56E-02	1.80E-02	NA	3.69E-02
RP11-54A4.2	rs72694955	9.26E-03	1.80E-02	NA	3.69E-02
ZFP41	rs7832163	9.52E-03	1.79E-02	NA	3.69E-02
UBE2D3	rs13107325	8.98E-03	1.27E-02	NA	3.69E-02
RP11-736N17.4	rs10132641	4.90E-03	9.74E-03	NA	3.69E-02
SPA17	rs12283429	8.48E-03	1.55E-02	NA	3.70E-02
IMMP2L	rs37716	4.31E-03	1.06E-02	NA	3.70E-02
KCTD18	rs3769446	3.64E-03	1.03E-02	NA	3.70E-02
KRTCAP3	rs4666014	2.41E-03	1.06E-02	NA	3.70E-02
VPS37B	rs4148862	5.40E-03	1.03E-02	NA	3.70E-02
HNRNPA1P35	rs3769446	5.40E-03	1.32E-02	NA	3.71E-02
KRT17P2	rs9890563	4.06E-03	1.33E-02	NA	3.71E-02

Gene	topSNP	p_eQTL	p_SMR	p_HEIDI	adjp_SMR
CTC-228N24.3	rs2764766	4.31E-03	1.67E-02	NA	3.71E-02
GMIP	rs880090	8.11E-03	1.67E-02	NA	3.71E-02
B3GAT1	rs73034295	1.39E-02	1.95E-02	NA	3.73E-02
BOLA1	rs72694955	1.05E-02	1.96E-02	NA	3.73E-02
HCP5	rs1046089	5.09E-03	8.00E-03	NA	3.73E-02
KCNJ13	rs6710294	1.32E-02	1.85E-02	NA	3.73E-02
RP1-283E3.4	rs2477686	1.13E-02	1.88E-02	NA	3.74E-02
SGOL2	rs59695400	1.11E-02	1.88E-02	NA	3.74E-02
ALDH5A1	rs77006546	1.06E-02	1.50E-02	NA	3.74E-02
ACTR1A	rs7911789	1.01E-02	1.46E-02	NA	3.74E-02
CNPY2	rs324015	8.89E-03	1.54E-02	NA	3.74E-02
EMC10	rs57940349	6.97E-03	1.54E-02	NA	3.74E-02
RP11-706C16.7	rs7832163	7.43E-03	1.51E-02	NA	3.74E-02
ZBTB39	rs324015	8.85E-03	1.54E-02	NA	3.74E-02
LRP4	rs6485685	1.27E-02	1.99E-02	NA	3.74E-02
RP11-665N17.4	rs1193851	1.01E-02	1.99E-02	NA	3.74E-02
ABHD14B	rs13072536	7.50E-03	1.62E-02	NA	3.75E-02
ITGAL	rs4788211	8.09E-03	1.62E-02	NA	3.75E-02
RP5-892K4.1	rs2494635	8.51E-03	1.61E-02	NA	3.75E-02
TMEM218	rs12541	8.52E-03	1.62E-02	NA	3.75E-02
C12orf76	rs12305489	9.00E-03	1.71E-02	NA	3.76E-02
HIST1H2BC	rs3208733	7.32E-03	1.49E-02	NA	3.76E-02
SPN	rs4788211	1.10E-02	2.01E-02	NA	3.76E-02
CAPRIN2	rs302317	5.48E-03	1.71E-02	NA	3.76E-02
RP11-770G2.2	rs1193851	8.12E-03	1.72E-02	NA	3.77E-02
GLI4	rs7832163	3.43E-03	8.99E-03	NA	3.77E-02
AC018766.6	rs7047	7.54E-03	1.37E-02	NA	3.78E-02
MRPL44	rs3768886	1.08E-02	2.03E-02	NA	3.78E-02
CTRL	rs1971546	9.35E-03	1.91E-02	NA	3.78E-02
XXbac-BPG308J9.3	rs1611209	1.37E-02	1.82E-02	NA	3.79E-02
PODXL	rs10954343	8.55E-03	1.77E-02	NA	3.79E-02
EPC2	rs76355118	4.79E-03	1.49E-02	NA	3.80E-02
SLC35E2B	rs2494635	7.96E-03	1.54E-02	NA	3.80E-02
SYBU	rs7007361	1.35E-02	2.05E-02	NA	3.80E-02
TNFAIP2	rs12880077	8.26E-03	1.78E-02	NA	3.80E-02
THEM6	rs7462788	1.00E-02	1.50E-02	NA	3.81E-02
ARMC6	rs880090	7.58E-03	1.60E-02	NA	3.82E-02
C2orf47	rs4270295	9.48E-03	1.61E-02	NA	3.82E-02
MIEF2	rs11078410	5.18E-03	1.60E-02	NA	3.82E-02
RP11-403I13.7	rs72694955	7.60E-03	1.57E-02	NA	3.82E-02
RP11-982M15.8	rs2368560	1.02E-02	1.59E-02	NA	3.82E-02
SUFU	rs284854	5.85E-03	1.55E-02	NA	3.82E-02
TLCD2	rs9893573	7.44E-03	1.60E-02	NA	3.82E-02

Gene	topSNP	p_eQTL	p_SMR	p_HEIDI	adjp_SMR
TTLL1	rs6002598	7.34E-03	1.61E-02	NA	3.82E-02
ZBTB18	rs884328	9.30E-03	1.79E-02	NA	3.82E-02
FBXL19	rs4424923	5.91E-03	1.18E-02	NA	3.82E-02
PTGES3	rs324017	5.74E-03	1.13E-02	NA	3.82E-02
TEF	rs134869	6.93E-03	1.19E-02	NA	3.82E-02
EXOC3L4	rs11623546	1.37E-02	1.94E-02	NA	3.83E-02
RP11-251G23.5	rs4730072	6.24E-03	1.22E-02	NA	3.83E-02
CCDC155	rs7047	8.33E-03	1.48E-02	NA	3.84E-02
LDB1	rs11191356	7.35E-03	1.43E-02	NA	3.84E-02
RP11-260M19.1	rs10132641	8.55E-03	1.47E-02	NA	3.84E-02
ZNF768	rs4788211	7.01E-03	1.47E-02	NA	3.84E-02
CTD-3074O7.12	rs1193851	4.93E-03	1.24E-02	NA	3.85E-02
PRDM16	rs2494635	4.60E-03	1.05E-02	NA	3.85E-02
SNX15	rs1193851	9.89E-03	1.97E-02	NA	3.85E-02
B4GALNT1	rs324015	5.56E-03	1.09E-02	NA	3.86E-02
CCS	rs1193851	4.03E-03	1.09E-02	NA	3.86E-02
OSTM1	rs4945816	2.03E-03	1.10E-02	NA	3.86E-02
ATP6V1E1P1	rs3001723	1.01E-02	1.63E-02	NA	3.86E-02
GIGYF2	rs709937	1.15E-02	1.65E-02	NA	3.86E-02
LETM2	rs17435276	6.32E-03	1.65E-02	NA	3.86E-02
TCF20	rs28579115	8.56E-03	1.67E-02	NA	3.86E-02
FAM72C	rs12124898	1.07E-02	1.99E-02	NA	3.87E-02
IZUMO1	rs7047	1.24E-02	1.99E-02	NA	3.87E-02
PBK	rs111659883	1.21E-02	2.00E-02	NA	3.87E-02
C6orf62	rs77006546	1.38E-02	1.88E-02	NA	3.87E-02
FOXP1	rs9837383	8.51E-03	1.73E-02	NA	3.87E-02
GPR124	rs60530611	7.82E-03	1.74E-02	NA	3.87E-02
IPO11	rs10041978	1.15E-02	1.71E-02	NA	3.87E-02
RNF43	rs35065479	5.25E-03	1.74E-02	NA	3.87E-02
RP1-140A9.1	rs16824398	9.83E-03	1.75E-02	NA	3.87E-02
FUT1	rs57940349	1.07E-02	2.05E-02	NA	3.87E-02
PACS1	rs1193851	1.06E-02	2.06E-02	NA	3.87E-02
SYPL1	rs2057884	1.31E-02	2.07E-02	NA	3.87E-02
ZNF48	rs4424923	1.26E-02	2.06E-02	NA	3.87E-02
VPS33B	rs4702	1.44E-02	2.10E-02	NA	3.88E-02
APOBEC3F	rs9306336	6.77E-03	1.53E-02	NA	3.88E-02
AP000708.1	rs12541	1.06E-02	1.89E-02	NA	3.88E-02
TM2D2	rs2016875	9.52E-03	1.76E-02	NA	3.88E-02
RP11-426C22.5	rs4424923	1.31E-02	2.12E-02	NA	3.89E-02
AMZ1	rs10950456	1.20E-02	1.73E-02	NA	3.89E-02
METTL1	rs324015	1.35E-02	2.11E-02	NA	3.90E-02
RP11-231C14.3	rs4424923	4.42E-03	9.64E-03	NA	3.90E-02
C10orf112	rs2148182	8.99E-03	1.77E-02	NA	3.91E-02

Gene	topSNP	p_eQTL	p_SMR	p_HEIDI	adjp_SMR
MEF2BNB	rs4808203	1.14E-02	1.76E-02	NA	3.91E-02
TNFRSF14	rs16824398	9.95E-03	1.77E-02	NA	3.91E-02
CCDC142	rs11903916	9.75E-03	1.89E-02	NA	3.91E-02
NXPH4	rs73115999	6.62E-03	1.57E-02	NA	3.91E-02
STAB1	rs7629072	1.19E-02	2.16E-02	NA	3.91E-02
CDC42P3	rs7833728	7.93E-03	1.65E-02	NA	3.92E-02
AL589739.1	rs16824398	1.11E-02	1.91E-02	NA	3.93E-02
AC005009.2	rs73210240	7.66E-03	1.50E-02	NA	3.93E-02
CBY1	rs55898159	6.29E-03	1.32E-02	NA	3.93E-02
FLII	rs6826	4.97E-03	1.45E-02	NA	3.93E-02
FUT2	rs57940349	5.91E-03	1.38E-02	NA	3.93E-02
KMT2E-AS1	rs4580973	6.40E-03	1.44E-02	NA	3.93E-02
PRSS56	rs2675956	9.55E-03	1.41E-02	NA	3.93E-02
LRP4-AS1	rs7476	7.96E-03	1.52E-02	NA	3.93E-02
ZNF286B	rs9890563	7.74E-03	1.94E-02	NA	3.94E-02
RP11-17G12.3	rs11038864	1.33E-02	2.15E-02	NA	3.94E-02
CHADL	rs5758659	1.55E-02	2.21E-02	NA	3.94E-02
AKAP13	rs12905223	5.73E-03	1.34E-02	NA	3.95E-02
AC074091.13	rs4666014	2.89E-03	1.17E-02	NA	3.95E-02
COPE	rs1054930	6.34E-03	1.15E-02	NA	3.95E-02
DPCR1	rs2596473	5.33E-03	1.16E-02	NA	3.95E-02
BAZ2A	rs73115999	7.17E-03	1.65E-02	NA	3.95E-02
NTM	rs73024809	5.90E-03	1.64E-02	NA	3.95E-02
OR2H2	rs3116856	1.15E-02	1.62E-02	NA	3.95E-02
RP11-367G6.3	rs6910549	1.28E-02	1.61E-02	NA	3.95E-02
GCKR	rs4666014	3.85E-03	1.36E-02	NA	3.95E-02
RP11-297M9.1	rs9926303	5.51E-03	1.36E-02	NA	3.95E-02
ZNF804A	rs62176172	8.14E-03	1.66E-02	NA	3.95E-02
AP003039.3	rs10894268	1.19E-02	1.95E-02	NA	3.96E-02
GRM2	rs1060330	9.38E-03	1.84E-02	NA	3.96E-02
RP11-214K3.19	rs4148862	1.15E-02	1.82E-02	NA	3.96E-02
RP1-92O14.3	rs3001723	1.17E-02	1.82E-02	NA	3.96E-02
BIK	rs134891	1.13E-02	1.84E-02	NA	3.96E-02
SAP30L	rs7727515	9.47E-03	1.83E-02	NA	3.96E-02
C1orf100	rs12751210	5.81E-03	1.37E-02	NA	3.96E-02
COL17A1	rs10883842	1.43E-02	1.97E-02	NA	3.96E-02
AC018766.4	rs7047	1.12E-02	1.84E-02	NA	3.97E-02
RP11-269F20.1	rs1058305	9.06E-03	1.70E-02	NA	3.98E-02
ZNF664	rs655293	8.62E-03	1.54E-02	NA	3.98E-02
SYNGR1	rs5757754	1.12E-02	1.98E-02	NA	3.98E-02
PSMB8	rs1003878	1.31E-02	1.77E-02	NA	3.98E-02
ADAMTS15	rs10894268	1.22E-02	1.99E-02	NA	3.98E-02
USP28	rs4245150	7.59E-03	1.40E-02	NA	3.98E-02

Gene	topSNP	p_eQTL	p_SMR	p_HEIDI	adjp_SMR
ETF1	rs55985421	9.80E-03	1.73E-02	NA	3.98E-02
RRP7B	rs2143139	1.02E-02	1.70E-02	NA	3.99E-02
AC068831.3	rs4932178	8.02E-03	1.60E-02	NA	4.01E-02
CARD11	rs11768637	1.11E-02	2.01E-02	NA	4.01E-02
PSMB10	rs1971546	1.15E-02	2.20E-02	NA	4.01E-02
CREB3L1	rs12574918	5.66E-03	1.21E-02	NA	4.01E-02
MMP16	rs10955542	5.08E-03	1.20E-02	NA	4.01E-02
SREBF1	rs9907287	3.58E-03	1.21E-02	NA	4.01E-02
DRD2	rs10736470	9.12E-03	1.64E-02	NA	4.01E-02
MRPS21	rs11589922	9.66E-03	1.81E-02	NA	4.01E-02
LHFPL3	rs4580973	1.06E-02	2.02E-02	NA	4.01E-02
RP1-37N7.3	rs4584886	7.56E-03	1.87E-02	NA	4.01E-02
TBC1D17	rs57940349	1.24E-02	2.27E-02	NA	4.03E-02
FAM180B	rs10838634	1.41E-02	2.22E-02	NA	4.03E-02
SOX2	rs1805589	1.15E-02	1.77E-02	NA	4.04E-02
HCG25	rs3130340	7.90E-03	1.09E-02	NA	4.04E-02
POLD1	rs7047	5.35E-03	1.07E-02	NA	4.04E-02
SHMT1	rs9907287	3.03E-03	1.10E-02	NA	4.04E-02
NFATC3	rs1971546	1.23E-02	2.30E-02	NA	4.04E-02
SCG2	rs3768886	1.29E-02	2.30E-02	NA	4.04E-02
RP11-17G12.2	rs12272795	1.53E-02	2.24E-02	NA	4.05E-02
ABT1	rs9348746	1.18E-02	1.78E-02	NA	4.05E-02
TAOK2	rs4583255	1.21E-02	1.83E-02	NA	4.05E-02
TRAF3	rs35498576	1.22E-02	1.82E-02	NA	4.05E-02
ZNF395	rs9331950	9.96E-03	1.79E-02	NA	4.05E-02
BDH2	rs13107325	1.82E-02	2.31E-02	NA	4.05E-02
COQ10A	rs324017	1.18E-02	1.92E-02	NA	4.05E-02
RP11-426C22.4	rs4788211	1.03E-02	1.92E-02	NA	4.05E-02
ABCB1	rs76349903	6.99E-03	1.47E-02	NA	4.05E-02
PANK4	rs2494631	8.06E-03	1.47E-02	NA	4.05E-02
C1orf222	rs2494635	1.01E-02	1.82E-02	NA	4.06E-02
PART1	rs4604142	1.20E-02	1.72E-02	NA	4.07E-02
RP3-395M20.8	rs16824398	9.65E-03	1.73E-02	NA	4.07E-02
CTD-2373J6.1	rs6891794	1.20E-02	1.85E-02	NA	4.07E-02
LINC01004	rs2299319	1.53E-02	2.33E-02	NA	4.07E-02
AL022476.2	rs2071711	1.39E-02	2.29E-02	NA	4.08E-02
CTC-467M3.3	rs62378245	1.56E-02	2.29E-02	NA	4.08E-02
NDUFA13	rs880090	1.12E-02	2.10E-02	NA	4.08E-02
G3BP1	rs17454953	9.93E-03	1.91E-02	NA	4.08E-02
RP3-508I15.22	rs55898159	1.08E-02	1.94E-02	NA	4.08E-02
OR2H1	rs1063320	8.55E-03	1.76E-02	NA	4.08E-02
RP11-258F1.1	rs11078410	6.37E-03	1.80E-02	NA	4.08E-02
MYH14	rs57940349	9.34E-03	1.87E-02	NA	4.08E-02

Gene	topSNP	p_eQTL	p_SMR	p_HEIDI	adjp_SMR
RNF5P1	rs2016875	1.42E-02	2.35E-02	NA	4.09E-02
JAGN1	rs61432137	1.10E-02	2.12E-02	NA	4.09E-02
PYGM	rs1193851	1.24E-02	2.30E-02	NA	4.09E-02
RP3-395M20.9	rs2494635	5.94E-03	1.25E-02	NA	4.10E-02
AC005197.2	rs880090	1.11E-02	2.08E-02	NA	4.11E-02
SLX1A	rs4424923	1.15E-02	1.92E-02	NA	4.11E-02
MATN1-AS1	rs1498231	1.14E-02	2.08E-02	NA	4.11E-02
C1orf233	rs2477686	1.18E-02	1.95E-02	NA	4.11E-02
PRRT3-AS1	rs61432137	9.96E-03	1.98E-02	NA	4.11E-02
NUDT3	rs210142	1.07E-02	1.96E-02	NA	4.12E-02
MAN2A2	rs4702	1.45E-02	2.10E-02	NA	4.12E-02
ARHGAP9	rs324017	6.03E-03	1.17E-02	NA	4.12E-02
XXbac-BPG308K3.6	rs3131336	1.73E-02	1.90E-02	NA	4.13E-02
RER1	rs942820	1.24E-02	2.01E-02	NA	4.13E-02
CD37	rs57940349	5.27E-03	1.28E-02	NA	4.14E-02
CALML6	rs10797426	6.29E-03	1.20E-02	NA	4.14E-02
RP1-265C24.8	rs7750526	2.04E-02	2.44E-02	NA	4.15E-02
LRRC36	rs1971546	4.81E-03	1.23E-02	NA	4.16E-02
NOLC1	rs7911789	1.88E-02	2.46E-02	NA	4.16E-02
AC005932.1	rs880090	1.09E-02	2.05E-02	NA	4.16E-02
ALDOA	rs4583255	1.25E-02	1.87E-02	NA	4.16E-02
CTA-223H9.9	rs5758527	1.07E-02	1.87E-02	NA	4.16E-02
ST13	rs9611469	1.05E-02	1.86E-02	NA	4.16E-02
HCG15	rs1063320	1.16E-02	2.16E-02	NA	4.17E-02
DGUOK	rs73947808	1.19E-02	2.15E-02	NA	4.17E-02
MRPL11	rs1193851	1.38E-02	2.48E-02	NA	4.17E-02
NR1H2	rs7047	1.64E-02	2.48E-02	NA	4.17E-02
KCTD13	rs4424923	6.73E-03	1.30E-02	NA	4.18E-02
RP11-45M22.3	rs6826	8.31E-03	1.98E-02	NA	4.18E-02
RRP7A	rs5758645	1.27E-02	1.99E-02	NA	4.18E-02
AL358813.2	rs11589922	7.95E-03	1.58E-02	NA	4.19E-02
BPI	rs6093098	1.93E-02	2.52E-02	NA	4.19E-02
RP11-314A5.3	rs11717383	1.49E-02	2.52E-02	NA	4.19E-02
TCTN2	rs655293	1.63E-02	2.49E-02	NA	4.19E-02
AC122129.1	rs2955365	1.20E-02	2.56E-02	NA	4.19E-02
IQCE	rs3800882	1.81E-02	2.55E-02	NA	4.19E-02
RP11-568K15.1	rs4650963	1.53E-02	2.56E-02	NA	4.19E-02
MUS81	rs1193851	1.02E-02	2.01E-02	NA	4.19E-02
CORO1A	rs4583255	1.32E-02	1.96E-02	NA	4.21E-02
AOX1	rs11688168	9.42E-03	1.38E-02	NA	4.21E-02
CTD-2008L17.1	rs72936329	8.09E-03	1.39E-02	NA	4.21E-02
RP11-231C14.4	rs4424923	7.78E-03	1.44E-02	NA	4.21E-02
SYNGAP1	rs176249	6.02E-03	1.36E-02	NA	4.21E-02

Gene	topSNP	p_eQTL	p_SMR	p_HEIDI	adjp_SMR
KIAA0319	rs77006546	1.42E-02	1.91E-02	NA	4.21E-02
ACY1	rs13072536	1.01E-02	1.98E-02	NA	4.21E-02
APLNR	rs708228	1.22E-02	2.06E-02	NA	4.21E-02
CTA-250D10.19	rs9611469	1.20E-02	2.04E-02	NA	4.21E-02
FEZ1	rs12283429	1.18E-02	1.97E-02	NA	4.21E-02
RP11-347C12.3	rs4788211	1.10E-02	2.01E-02	NA	4.21E-02
RP5-951N9.1	rs11062170	1.64E-02	2.06E-02	NA	4.21E-02
SNX3	rs4945816	6.80E-03	2.04E-02	NA	4.21E-02
SULT1A3	rs4424923	1.23E-02	2.02E-02	NA	4.21E-02
ZNF486	rs880090	1.05E-02	2.00E-02	NA	4.21E-02
CHRND	rs6710294	1.14E-02	1.64E-02	NA	4.21E-02
RP3-395M20.3	rs2494635	8.67E-03	1.63E-02	NA	4.21E-02
RP11-473I1.5	rs7196708	6.99E-03	1.66E-02	NA	4.21E-02
PARP3	rs1060330	1.36E-02	2.39E-02	NA	4.22E-02
CTC-559E9.6	rs8101938	1.07E-02	2.02E-02	NA	4.22E-02
KMT2E	rs2299319	1.27E-02	2.03E-02	NA	4.22E-02
MARCKSL1P1	rs11191356	1.49E-02	2.40E-02	NA	4.23E-02
INPP5D	rs6710294	2.01E-02	2.63E-02	NA	4.23E-02
SEMA3B-AS1	rs9852677	2.79E-04	2.68E-02	NA	4.23E-02
SFXN5	rs2077586	1.56E-02	2.66E-02	NA	4.23E-02
LRRC16A	rs3734528	1.89E-02	2.23E-02	NA	4.24E-02
NPM3	rs2031604	1.40E-02	2.22E-02	NA	4.24E-02
SCARA5	rs111659883	1.79E-02	2.70E-02	NA	4.24E-02
TRPV4	rs11065628	1.10E-02	2.09E-02	NA	4.25E-02
MEF2C-AS1	rs62378245	1.68E-02	2.43E-02	NA	4.25E-02
C6orf106	rs1318691	1.37E-02	2.27E-02	NA	4.26E-02
ADAM22	rs73210240	1.25E-02	2.13E-02	NA	4.26E-02
FAT2	rs2961756	1.14E-02	2.13E-02	NA	4.26E-02
RP11-677M14.5	rs12283429	1.35E-02	2.19E-02	NA	4.26E-02
SNRPGP2	rs9964176	1.24E-02	2.18E-02	NA	4.26E-02
LRP1	rs73115999	1.11E-02	2.20E-02	NA	4.27E-02
NNMT	rs4245150	1.39E-02	2.20E-02	NA	4.27E-02
RP11-958J22.1	rs61882712	1.38E-02	2.07E-02	NA	4.27E-02
KIAA1324L	rs73206909	1.54E-02	2.46E-02	NA	4.27E-02
PLEKHG4	rs1971546	1.36E-02	2.46E-02	NA	4.27E-02
CYP2W1	rs34922657	1.51E-02	2.13E-02	NA	4.27E-02
MORN1	rs942820	1.34E-02	2.13E-02	NA	4.27E-02
PDE6D	rs6710294	1.67E-02	2.26E-02	NA	4.28E-02
RP11-603J24.5	rs324015	1.47E-02	2.26E-02	NA	4.28E-02
NDUFA3P4	rs4932178	9.31E-03	1.78E-02	NA	4.29E-02
RP11-382A20.6	rs1145173	1.39E-02	2.32E-02	NA	4.29E-02
RPS11	rs7047	1.67E-02	2.50E-02	NA	4.29E-02
SNORA60	rs6093098	1.60E-02	2.15E-02	NA	4.29E-02

Gene	topSNP	p_eQTL	p_SMR	p_HEIDI	adjp_SMR
PRRC1	rs2764766	7.07E-03	2.17E-02	NA	4.30E-02
SEMA4B	rs4702	1.51E-02	2.17E-02	NA	4.30E-02
CTD-2008L17.2	rs72930740	7.32E-03	1.53E-02	NA	4.30E-02
MYL6	rs324015	9.96E-03	1.68E-02	NA	4.30E-02
RP11-657O9.1	rs696520	9.23E-03	1.54E-02	NA	4.30E-02
SUN2	rs1008677	1.07E-02	1.66E-02	NA	4.30E-02
VSTM2L	rs208818	9.80E-03	1.65E-02	NA	4.30E-02
ACD	rs1971546	1.20E-02	2.26E-02	NA	4.30E-02
SATB2	rs796364	1.81E-02	2.31E-02	NA	4.32E-02
RP11-752G15.9	rs1269134	1.37E-02	2.33E-02	NA	4.33E-02
RDH16	rs324017	1.91E-02	2.78E-02	NA	4.34E-02
RP11-214K3.23	rs11057238	8.17E-03	1.54E-02	NA	4.34E-02
SPRYD4	rs324017	1.44E-02	2.24E-02	NA	4.35E-02
AF131215.9	rs73191548	9.90E-03	2.19E-02	NA	4.35E-02
WDR65	rs3001723	1.47E-02	2.18E-02	NA	4.35E-02
APOBEC3B	rs1008677	1.66E-02	2.36E-02	NA	4.35E-02
BRD8	rs256013	1.35E-02	2.39E-02	NA	4.36E-02
ZSWIM6	rs4604142	1.79E-02	2.40E-02	NA	4.36E-02
NOTO	rs2077586	1.21E-02	2.23E-02	NA	4.36E-02
SLC4A1AP	rs12474906	1.11E-02	2.21E-02	NA	4.36E-02
SSU72	rs16824398	1.36E-02	2.23E-02	NA	4.36E-02
AC004447.2	rs4808203	1.64E-02	2.36E-02	NA	4.36E-02
HNRNPA1P38	rs56055628	1.37E-02	2.37E-02	NA	4.36E-02
MNT	rs9893573	1.32E-02	2.38E-02	NA	4.36E-02
SLC9B1	rs13107325	1.91E-02	2.41E-02	NA	4.36E-02
SV2A	rs7555080	1.32E-02	2.29E-02	NA	4.36E-02
APOBEC3D	rs5757754	1.30E-02	2.20E-02	NA	4.36E-02
RP5-1107A17.4	rs35065479	6.93E-03	2.02E-02	NA	4.36E-02
KCNIP2-AS1	rs11191356	1.63E-02	2.57E-02	NA	4.36E-02
SGSM3	rs55898159	1.60E-02	2.57E-02	NA	4.36E-02
FBXO41	rs2077586	1.08E-02	2.05E-02	NA	4.36E-02
BOLA2B	rs4424923	1.57E-02	2.43E-02	NA	4.37E-02
ADSSL1	rs2368560	1.74E-02	2.45E-02	NA	4.37E-02
AC010524.2	rs7047	1.34E-02	2.11E-02	NA	4.38E-02
RP11-342A23.1	rs17601029	1.02E-02	2.11E-02	NA	4.38E-02
ERI3	rs3001723	1.58E-02	2.31E-02	NA	4.38E-02
RP11-45P15.4	rs34633710	1.46E-02	2.42E-02	NA	4.39E-02
TMEM200B	rs1498231	9.23E-03	1.79E-02	NA	4.39E-02
MAN2A1	rs2269201	1.10E-02	2.50E-02	NA	4.40E-02
DOCK4-AS1	rs37716	1.59E-02	2.62E-02	NA	4.40E-02
NPY6R	rs55985421	1.73E-02	2.65E-02	NA	4.40E-02
WDR17	rs1106568	1.83E-02	2.63E-02	NA	4.40E-02
RBKS	rs12474906	1.05E-02	2.13E-02	NA	4.40E-02

Gene	topSNP	p_eQTL	p_SMR	p_HEIDI	adjp_SMR
ASS1P1	rs6910549	2.10E-02	2.51E-02	NA	4.41E-02
CTSW	rs1193851	1.39E-02	2.49E-02	NA	4.41E-02
HNRNPKP1	rs2764766	9.32E-03	2.53E-02	NA	4.41E-02
HSD17B14	rs7047	1.69E-02	2.53E-02	NA	4.41E-02
ZNF691	rs3001723	1.76E-02	2.52E-02	NA	4.41E-02
AC135048.1	rs4788211	1.32E-02	2.28E-02	NA	4.41E-02
SCNM1	rs12124898	1.77E-02	2.87E-02	NA	4.41E-02
RP1-178F10.3	rs11078410	1.14E-02	2.55E-02	NA	4.42E-02
RP13-582O9.7	rs7832163	1.30E-02	2.24E-02	NA	4.42E-02
PRSS55	rs73191548	1.07E-02	2.30E-02	NA	4.42E-02
ZCCHC8	rs10846491	1.36E-02	2.26E-02	NA	4.42E-02
KB-1836B5.4	rs17435276	1.22E-02	2.50E-02	NA	4.43E-02
CNIH2	rs1193851	1.46E-02	2.58E-02	NA	4.44E-02
NOSIP	rs7047	1.74E-02	2.58E-02	NA	4.44E-02
SEC13	rs61432137	1.22E-02	2.27E-02	NA	4.44E-02
RP11-245J9.5	rs13272	1.91E-02	2.90E-02	NA	4.45E-02
FBXO16	rs9331950	1.39E-02	2.30E-02	NA	4.45E-02
TMEM59L	rs4808203	1.58E-02	2.28E-02	NA	4.45E-02
RP11-12M9.4	rs9607850	1.31E-02	2.32E-02	NA	4.45E-02
AP000769.1	rs1193851	1.32E-02	2.40E-02	NA	4.45E-02
SGSM2	rs2224770	1.35E-02	2.39E-02	NA	4.45E-02
SRSF1	rs35065479	9.28E-03	2.39E-02	NA	4.45E-02
ADIG	rs6093098	1.78E-02	2.36E-02	NA	4.46E-02
GRM4	rs9394145	1.33E-02	2.35E-02	NA	4.46E-02
OBFC1	rs10786736	1.65E-02	2.36E-02	NA	4.46E-02
RP11-353N4.4	rs72694957	1.47E-02	2.36E-02	NA	4.46E-02
LINC00663	rs4808203	1.21E-02	1.84E-02	NA	4.46E-02
RP11-121M22.1	rs73024809	7.13E-03	1.83E-02	NA	4.46E-02
ITIH1	rs6769789	1.50E-02	2.32E-02	NA	4.46E-02
ACTR8	rs7626551	1.22E-02	2.00E-02	NA	4.47E-02
CA14	rs72694955	1.06E-02	1.98E-02	NA	4.47E-02
RPS2P4	rs10132641	1.28E-02	2.00E-02	NA	4.47E-02
RP11-181B11.1	rs2565065	1.51E-02	2.43E-02	NA	4.47E-02
PSCA	rs11778057	1.97E-02	2.65E-02	NA	4.47E-02
RP11-353N4.5	rs12124898	1.57E-02	2.63E-02	NA	4.48E-02
BANK1	rs13107325	2.40E-02	2.94E-02	NA	4.50E-02
IL4I1	rs57940349	7.89E-03	1.67E-02	NA	4.50E-02
LINC00568	rs72694950	9.03E-03	1.70E-02	NA	4.50E-02
USP40	rs6710294	1.22E-02	1.74E-02	NA	4.50E-02
RP11-752G15.3	rs75884951	8.45E-03	1.75E-02	NA	4.50E-02
DUSP7	rs1060330	1.42E-02	2.46E-02	NA	4.50E-02
ZNF682	rs4808203	1.74E-02	2.46E-02	NA	4.50E-02
ATG13	rs60722866	2.01E-02	2.74E-02	NA	4.50E-02

Gene	topSNP	p_eQTL	p_SMR	p_HEIDI	adjp_SMR
CCDC39	rs1871355	1.35E-02	2.39E-02	NA	4.50E-02
RCN3	rs57940349	8.85E-03	1.80E-02	NA	4.50E-02
HSPE1	rs11900232	1.97E-02	2.76E-02	NA	4.51E-02
WDR81	rs216221	1.76E-02	2.76E-02	NA	4.51E-02
CTC-260E6.10	rs15622	1.52E-02	2.40E-02	NA	4.51E-02
RUNDC3B	rs56936192	1.63E-02	2.71E-02	NA	4.52E-02
CCDC92	rs10846491	1.70E-02	2.67E-02	NA	4.52E-02
DCP1B	rs2238053	1.41E-02	1.91E-02	NA	4.52E-02
CTC-332L22.1	rs4388249	1.11E-02	2.38E-02	NA	4.52E-02
RP11-73M18.11	rs35498576	1.72E-02	2.41E-02	NA	4.53E-02
SPATA4	rs6846161	1.63E-02	2.39E-02	NA	4.53E-02
RASGRP2	rs1193851	1.35E-02	2.44E-02	NA	4.53E-02
C16orf93	rs4583255	1.31E-02	1.94E-02	NA	4.53E-02
HLA-DRB9	rs176249	9.65E-03	1.87E-02	NA	4.53E-02
SLURP1	rs4976982	1.88E-02	2.42E-02	NA	4.54E-02
A4GALT	rs5758605	1.20E-02	1.91E-02	NA	4.54E-02
KHDC1	rs1548335	1.03E-02	1.91E-02	NA	4.54E-02
RP13-582O9.5	rs72687376	1.74E-02	2.80E-02	NA	4.54E-02
SLC12A4	rs1971546	1.63E-02	2.81E-02	NA	4.54E-02
BCL11B	rs11624408	1.15E-02	2.12E-02	NA	4.54E-02
GET4	rs6957894	1.19E-02	1.97E-02	NA	4.54E-02
PRADC1	rs2077586	1.12E-02	2.11E-02	NA	4.54E-02
RP11-670E13.2	rs35065479	7.34E-03	2.09E-02	NA	4.54E-02
SCAF1	rs57940349	1.05E-02	2.03E-02	NA	4.54E-02
RP11-305O4.3	rs9845788	1.54E-02	2.54E-02	NA	4.55E-02
FAM227A	rs9306336	1.34E-02	2.43E-02	NA	4.55E-02
CLK1	rs3769446	1.54E-02	2.68E-02	NA	4.55E-02
NR1H3	rs7476	1.17E-02	2.00E-02	NA	4.55E-02
PLXNA4	rs10954343	1.01E-02	1.99E-02	NA	4.55E-02
RNU6-312P	rs3769446	1.60E-02	2.75E-02	NA	4.56E-02
PEMT	rs9907287	1.60E-02	3.04E-02	NA	4.56E-02
RP11-1167A19.2	rs1193851	1.84E-02	3.05E-02	NA	4.56E-02
AC013472.4	rs12474906	1.50E-02	2.71E-02	NA	4.56E-02
BZRAP1-AS1	rs35065479	1.14E-02	2.71E-02	NA	4.56E-02
RP11-503P10.1	rs12474906	1.48E-02	2.70E-02	NA	4.56E-02
YWHAE	rs216176	1.72E-02	2.73E-02	NA	4.56E-02
RP5-821D11.7	rs5996096	1.36E-02	2.45E-02	NA	4.57E-02
TMEM184A	rs6945719	2.37E-02	3.06E-02	NA	4.57E-02
RPS10	rs942496	1.49E-02	2.45E-02	NA	4.57E-02
STAC3	rs324015	2.17E-02	3.07E-02	NA	4.57E-02
ZNF90	rs4808203	1.84E-02	2.58E-02	NA	4.58E-02
RP11-430H10.1	rs876701	1.76E-02	2.48E-02	NA	4.58E-02
ELOVL1	rs3001723	1.37E-02	2.06E-02	NA	4.58E-02

Gene	topSNP	p_eQTL	p_SMR	p_HEIDI	adjp_SMR
DIABLO	rs655293	1.86E-02	2.76E-02	NA	4.59E-02
RP11-977G19.14	rs324017	1.91E-02	2.78E-02	NA	4.59E-02
RRAS	rs57940349	1.67E-02	2.81E-02	NA	4.59E-02
PAM	rs1823694	1.13E-02	2.52E-02	NA	4.59E-02
RP11-225H22.5	rs10883798	2.02E-02	2.55E-02	NA	4.59E-02
SF3B4	rs7555080	1.75E-02	2.81E-02	NA	4.59E-02
C14orf2	rs35498576	1.87E-02	2.58E-02	NA	4.60E-02
CACNB2	rs2148182	1.19E-02	2.15E-02	NA	4.60E-02
KIF5C	rs76355118	1.22E-02	2.62E-02	NA	4.61E-02
PTP4A2P2	rs73036062	1.95E-02	2.63E-02	NA	4.61E-02
TSPO	rs2143139	2.23E-02	3.12E-02	NA	4.62E-02
RP11-561C5.5	rs11631921	1.36E-02	2.64E-02	NA	4.62E-02
HOMER2	rs4779046	1.74E-02	2.84E-02	NA	4.62E-02
RP11-109N23.6	rs2332704	1.24E-02	2.17E-02	NA	4.63E-02
RP11-522N14.2	rs7304782	1.60E-02	2.15E-02	NA	4.63E-02
JOSD1	rs1008677	2.02E-02	2.77E-02	NA	4.63E-02
IFT81	rs11065628	1.13E-02	2.13E-02	NA	4.64E-02
LINC00852	rs61432137	1.93E-02	3.15E-02	NA	4.64E-02
LINC00222	rs13217795	1.20E-02	2.88E-02	NA	4.64E-02
RP11-458I7.1	rs72694965	1.84E-02	2.81E-02	NA	4.65E-02
PLD5	rs10803138	1.32E-02	2.20E-02	NA	4.65E-02
RBMS2	rs324015	1.42E-02	2.20E-02	NA	4.66E-02
NIF3L1	rs3769446	1.53E-02	2.66E-02	NA	4.66E-02
AC074117.13	rs4666014	1.12E-02	2.54E-02	NA	4.66E-02
TMEM179	rs3861678	1.90E-02	2.57E-02	NA	4.66E-02
HDDC3	rs4932178	1.86E-02	2.93E-02	NA	4.67E-02
RAP1GAP2	rs2224770	1.79E-02	2.94E-02	NA	4.67E-02
TAC3	rs324015	2.05E-02	2.93E-02	NA	4.67E-02
BRMS1	rs1193851	1.60E-02	2.76E-02	NA	4.67E-02
MEF2B	rs4808203	1.99E-02	2.75E-02	NA	4.67E-02
COIL	rs35065479	8.00E-03	2.20E-02	NA	4.67E-02
RP11-345J4.8	rs4424923	1.38E-02	2.21E-02	NA	4.67E-02
RP11-10L12.4	rs13107325	2.63E-02	3.19E-02	NA	4.67E-02
PDGFB	rs55898159	1.61E-02	2.59E-02	NA	4.67E-02
RP11-697E2.7	rs4932178	1.45E-02	2.43E-02	NA	4.68E-02
DGKE	rs35065479	1.15E-02	2.72E-02	NA	4.68E-02
MSRA	rs73191548	1.43E-02	2.78E-02	NA	4.68E-02
ERCC8	rs10041978	1.93E-02	2.61E-02	NA	4.69E-02
GHRL	rs61432137	1.56E-02	2.70E-02	NA	4.69E-02
MGEA5	rs284854	1.29E-02	2.57E-02	NA	4.69E-02
PCBP4	rs4687680	1.83E-02	2.69E-02	NA	4.69E-02
PFKFB2	rs12749612	1.33E-02	2.67E-02	NA	4.69E-02
SCAND2P	rs1051168	1.74E-02	2.71E-02	NA	4.69E-02

Gene	topSNP	p_eQTL	p_SMR	p_HEIDI	adjp_SMR
TSSK6	rs1054284	1.81E-02	2.62E-02	NA	4.69E-02
HIST2H2BE	rs72694965	1.67E-02	2.61E-02	NA	4.69E-02
ARC	rs72687376	1.86E-02	2.95E-02	NA	4.69E-02
RP11-486O12.2	rs941306	1.93E-02	2.90E-02	NA	4.69E-02
PTN	rs3735026	2.33E-02	3.23E-02	NA	4.69E-02
CACNA1C-AS1	rs11062157	2.19E-02	3.31E-02	NA	4.69E-02
CALHM3	rs17114641	2.37E-02	3.33E-02	NA	4.69E-02
DIS3L2	rs11535	2.61E-02	3.27E-02	NA	4.69E-02
PPARGC1A	rs215405	1.84E-02	3.31E-02	NA	4.69E-02
TANGO6	rs1971546	2.02E-02	3.27E-02	NA	4.69E-02
WDFY1	rs3768886	2.13E-02	3.32E-02	NA	4.69E-02
CMAHP	rs77006546	2.28E-02	2.86E-02	NA	4.70E-02
IL17RE	rs61432137	1.69E-02	2.86E-02	NA	4.70E-02
COPS3	rs4584886	1.48E-02	2.88E-02	NA	4.71E-02
NRGN	rs12541	1.90E-02	2.92E-02	NA	4.71E-02
RP3-508I15.20	rs1008677	2.15E-02	2.91E-02	NA	4.71E-02
SUGP2	rs880090	1.76E-02	2.89E-02	NA	4.71E-02
CTF1	rs4424923	1.63E-02	2.50E-02	NA	4.71E-02
ATF7IP2	rs7199375	1.56E-02	2.98E-02	NA	4.71E-02
SGK223	rs2948286	2.20E-03	2.26E-02	NA	4.72E-02
CTB-113P19.3	rs2961756	1.22E-02	2.24E-02	NA	4.72E-02
C22orf46	rs5758268	1.87E-02	2.96E-02	NA	4.72E-02
EIF4E3	rs56343799	1.76E-02	2.95E-02	NA	4.72E-02
LYPD6	rs76355118	1.47E-02	2.95E-02	NA	4.72E-02
PKD2L2	rs256013	1.82E-02	2.96E-02	NA	4.73E-02
ZNF592	rs2135551	1.80E-02	2.96E-02	NA	4.73E-02
LYSMD3	rs2247870	1.06E-02	2.62E-02	NA	4.73E-02
CDK11B	rs2494635	1.60E-02	2.56E-02	NA	4.73E-02
TBC1D10B	rs4583255	1.86E-02	2.58E-02	NA	4.73E-02
TNRC6B	rs55898159	1.59E-02	2.57E-02	NA	4.73E-02
DAXX	rs1003878	1.76E-02	2.27E-02	NA	4.74E-02
BNIP3L	rs3808573	1.83E-02	3.01E-02	NA	4.75E-02
ADSL	rs9611469	2.00E-02	3.01E-02	NA	4.76E-02
RP1-46F2.2	rs11065628	1.27E-02	2.31E-02	NA	4.76E-02
AC099684.1	rs12603592	2.27E-02	3.44E-02	NA	4.76E-02
AP3S2	rs4932178	2.30E-02	3.44E-02	NA	4.76E-02
NYAP2	rs3768886	2.23E-02	3.43E-02	NA	4.76E-02
BRAT1	rs872465	1.50E-02	2.41E-02	NA	4.76E-02
HCG16	rs34130214	2.10E-02	2.43E-02	NA	4.76E-02
PLEKHA4	rs57940349	1.33E-02	2.39E-02	NA	4.76E-02
RP11-953B20.1	rs4976982	1.79E-02	2.32E-02	NA	4.76E-02
RPA1	rs2224770	1.87E-02	3.04E-02	NA	4.76E-02
PLD6	rs11078410	1.86E-02	3.50E-02	NA	4.76E-02

**Gene**	**topSNP**	**p_eQTL**	**p_SMR**	**p_HEIDI**	**adjp_SMR**
RRP9	rs13072536	2.23E-02	3.48E-02	NA	4.76E-02
RP11-1020A11.2	rs61432137	1.72E-02	2.90E-02	NA	4.77E-02
GBF1	rs35525740	1.73E-02	2.31E-02	NA	4.77E-02
PSD	rs284858	1.18E-02	2.31E-02	NA	4.77E-02
AP000662.4	rs708228	2.08E-02	3.08E-02	NA	4.78E-02
FOXO3B	rs9890563	1.60E-02	3.08E-02	NA	4.78E-02
TSGA10IP	rs1193851	1.68E-02	2.84E-02	NA	4.78E-02
DIMT1	rs10041978	2.35E-02	3.08E-02	NA	4.78E-02
HMGA1	rs511515	1.45E-02	2.52E-02	NA	4.79E-02
RP11-403I13.8	rs12124898	1.48E-02	2.51E-02	NA	4.79E-02
RP11-677M14.6	rs12283429	1.61E-02	2.50E-02	NA	4.79E-02
ARHGAP1	rs10838634	2.13E-02	3.06E-02	NA	4.79E-02
RP11-496B10.3	rs1805589	1.89E-02	2.64E-02	NA	4.80E-02
GPN1	rs12474906	1.82E-02	3.11E-02	NA	4.80E-02
C14orf180	rs4906379	2.13E-02	2.87E-02	NA	4.80E-02
RP11-158M2.3	rs3803405	1.73E-02	3.15E-02	NA	4.80E-02
RP11-723D22.3	rs17435276	1.74E-02	3.17E-02	NA	4.80E-02
MLLT11	rs12124898	1.96E-02	3.09E-02	NA	4.81E-02
AVIL	rs73115999	1.51E-02	2.72E-02	NA	4.81E-02
RP11-146F11.5	rs4583255	2.78E-02	3.59E-02	NA	4.81E-02
AF131215.2	rs73191548	1.40E-02	2.75E-02	NA	4.82E-02
AP006547.3	rs28460883	2.09E-02	2.99E-02	NA	4.82E-02
RP11-297M9.2	rs1420042	1.79E-02	3.11E-02	NA	4.82E-02
SAG	rs11535	2.57E-02	3.24E-02	NA	4.83E-02
TTC12	rs10750025	2.24E-02	3.24E-02	NA	4.83E-02
AC109829.1	rs12474906	1.66E-02	2.92E-02	NA	4.83E-02
MBLAC2	rs2247870	9.72E-03	2.98E-02	NA	4.83E-02
NCAN	rs8101938	1.83E-02	2.97E-02	NA	4.83E-02
SIVA1	rs4906379	2.17E-02	2.91E-02	NA	4.83E-02
SLC25A40	rs73210260	1.82E-02	2.90E-02	NA	4.83E-02
SZT2	rs3001723	2.19E-02	3.00E-02	NA	4.83E-02
ATPAF2	rs2955367	1.46E-02	3.14E-02	NA	4.84E-02
SLC5A6	rs12474906	1.80E-02	3.09E-02	NA	4.84E-02
ARL5B	rs4748465	2.00E-02	3.01E-02	NA	4.84E-02
CDC42EP2	rs1193851	1.93E-02	3.15E-02	NA	4.85E-02
ST3GAL3	rs3001723	2.32E-02	3.15E-02	NA	4.85E-02
FAM222A-AS1	rs3026445	2.00E-02	3.12E-02	NA	4.85E-02
AC009245.3	rs1647181	2.19E-02	3.17E-02	NA	4.86E-02
BAI1	rs28460883	2.29E-02	3.21E-02	NA	4.86E-02
RP11-22L13.1	rs2477686	2.29E-02	3.24E-02	NA	4.86E-02
RP11-543P15.1	rs1016388	2.65E-02	3.27E-02	NA	4.86E-02
NPTXR	rs1008677	2.12E-02	2.87E-02	NA	4.86E-02
PRSS51	rs73191548	1.50E-02	2.86E-02	NA	4.86E-02

Gene	topSNP	p_eQTL	p_SMR	p_HEIDI	adjp_SMR
RP11-261C10.3	rs12751210	1.65E-02	2.80E-02	NA	4.86E-02
STAMBP	rs73947808	1.66E-02	2.73E-02	NA	4.86E-02
ZNF689	rs4424923	1.87E-02	2.79E-02	NA	4.86E-02
RPL7P4	rs3132564	2.43E-02	3.17E-02	NA	4.86E-02
CTD-2012K14.7	rs1971546	1.91E-02	3.14E-02	NA	4.87E-02
RP11-499E18.1	rs13107325	1.93E-02	2.43E-02	NA	4.87E-02
OS9	rs73115999	1.41E-02	2.59E-02	NA	4.87E-02
PEX16	rs10838634	2.27E-02	3.22E-02	NA	4.87E-02
ST14	rs4936123	1.96E-02	3.24E-02	NA	4.88E-02
IL17RC	rs61432137	2.08E-02	3.32E-02	NA	4.89E-02
MFHAS1	rs2948286	1.05E-02	2.66E-02	NA	4.89E-02
FBXL19-AS1	rs4583255	2.22E-02	2.98E-02	NA	4.90E-02
EDC4	rs7187202	2.00E-02	3.27E-02	NA	4.91E-02
FOSL2	rs4666014	1.69E-02	3.29E-02	NA	4.91E-02
NUCB1	rs7047	2.36E-02	3.29E-02	NA	4.91E-02
RP4-564F22.5	rs6093097	2.62E-02	3.28E-02	NA	4.91E-02
FES	rs4702	1.93E-02	2.65E-02	NA	4.91E-02
FCGR1C	rs72694957	2.17E-02	3.18E-02	NA	4.91E-02
EIF4EBP1	rs9198	2.23E-02	3.28E-02	NA	4.91E-02
JRK	rs7462788	2.57E-02	3.28E-02	NA	4.91E-02
PRKCD	rs13072536	2.07E-02	3.29E-02	NA	4.91E-02
RP11-567M21.3	rs4936123	2.02E-02	3.31E-02	NA	4.91E-02
C16orf92	rs4424923	2.23E-02	3.20E-02	NA	4.92E-02
INTS1	rs1476887	2.19E-02	2.81E-02	NA	4.93E-02
NSUN6	rs1409203	2.37E-02	3.35E-02	NA	4.93E-02
DDB2	rs10838634	2.52E-02	3.50E-02	NA	4.94E-02
EIF1AD	rs1193851	2.10E-02	3.35E-02	NA	4.95E-02
IPO13	rs3001723	2.50E-02	3.34E-02	NA	4.95E-02
GIN1	rs1823694	1.51E-02	3.03E-02	NA	4.95E-02
IL20RB	rs9845788	1.99E-02	3.08E-02	NA	4.95E-02
REXO2	rs10736470	2.06E-02	3.03E-02	NA	4.95E-02
RP11-619L19.2	rs784255	2.27E-02	3.08E-02	NA	4.95E-02
GYLTL1B	rs10838634	2.54E-02	3.52E-02	NA	4.96E-02
ZNF710	rs35346340	2.43E-02	3.53E-02	NA	4.96E-02
PDE4D	rs4604142	2.47E-02	3.13E-02	NA	4.96E-02
RP11-375N15.2	rs73191548	1.74E-02	3.17E-02	NA	4.96E-02
RPL35P2	rs511515	1.95E-02	3.12E-02	NA	4.96E-02
RP11-234N17.1	rs1702294	2.91E-02	3.43E-02	NA	4.96E-02
AC078842.3	rs3735025	2.53E-02	3.44E-02	NA	4.96E-02
BLM	rs6227	1.54E-02	2.44E-02	NA	4.96E-02
HNRNPA1P9	rs76349903	1.44E-02	2.44E-02	NA	4.96E-02
SNX7	rs9662719	2.28E-02	3.19E-02	NA	4.96E-02
ZNF771	rs4788211	2.08E-02	3.19E-02	NA	4.96E-02

Gene	topSNP	p_eQTL	p_SMR	p_HEIDI	adjp_SMR
AL590822.1	rs2494631	2.15E-02	3.10E-02	NA	4.96E-02
BHLHA9	rs216176	2.07E-02	3.14E-02	NA	4.96E-02
CASP10	rs6708345	1.98E-02	3.14E-02	NA	4.96E-02
PEX10	rs10797426	2.24E-02	3.15E-02	NA	4.96E-02
CTNNA1	rs187653	1.68E-02	2.72E-02	NA	4.97E-02
SCML4	rs9400240	2.04E-02	3.77E-02	NA	4.98E-02
CEP170B	rs10132641	2.36E-02	3.24E-02	NA	4.98E-02
CTXN3	rs2764766	1.44E-02	3.25E-02	NA	4.98E-02
ATG7	rs61432137	2.15E-02	3.41E-02	NA	4.99E-02
RP11-675F6.4	rs9198	2.32E-02	3.39E-02	NA	4.99E-02
RP11-3P17.4	rs536964	2.14E-02	3.31E-02	NA	5.00E-02
RP6-91H8.5	rs2332704	2.20E-02	3.32E-02	NA	5.00E-02

(3) The most significant eQTL analyses results in brain from CMC.

Gene	SNP	P_value
AC103965	rs11630887	<0.01
AL022393	rs111770007	<0.01
ALMS1P	rs11903916	<0.01
ALMS1	rs11903916	<0.01
AP3B2	rs1145173	<0.01
ARL3	rs10786701	<0.01
AS3MT	rs10883842	<0.01
ATP6V0D1	rs1971546	<0.01
ATP6V1G2	rs1265092	<0.01
BAG4	rs16887244	<0.01
BAG6	rs433061	<0.01
BAK1	rs210142	<0.01
BRD2	rs433061	<0.01
BTN2A1	rs6940638	<0.01
BTN3A2	rs13216828	<0.01
BTN3A3	rs2235251	<0.01
C10orf32	rs10883842	<0.01
C2orf47	rs11688168	<0.01
C2orf82	rs1083522	<0.01
C4A	rs1265092	<0.01
CD46	rs12749612	<0.01
CNNM2	rs10883842	<0.01
CNTN4	rs9845120	<0.01
CPEB1	rs1145173	<0.01
CSDC2	rs12158335	<0.01
CSPG4P11	rs11630887	<0.01
CSPG4P12	rs11630887	<0.01
CTSS	rs72694955	<0.01
DDAH2	rs433061	<0.01
DDHD2	rs11779986	<0.01
DNM1P51	rs57489559	<0.01
DOC2A	rs11644809	<0.01
DRG2	rs11078410	<0.01
EFTUD1P1	rs11630887	<0.01
ELOVL7	rs10041978	<0.01
ERI1	rs2948286	<0.01
F8	rs2266886	<0.01
FAM109B	rs133382	<0.01
FAM154B	rs4779046	<0.01
FAM66A	rs2948286	<0.01
FAM66D	rs2948286	<0.01

Gene	SNP	P_value
FAM86B3P	rs2948286	<0.01
FANCL	rs11688767	<0.01
FES	rs11372849	<0.01
FTCDNL1	rs11333159	<0.01
FTSJ2	rs12699453	<0.01
FURIN	rs11372849	<0.01
GATAD2A	rs10282	<0.01
GLT8D1	rs11717383	<0.01
GLYCTK	rs11717383	<0.01
GOLGA6L4	rs11630887	<0.01
GUSBP2	rs6932590	<0.01
HIST1H2BC	rs198811	<0.01
HIST1H4B	rs198811	<0.01
HLA-B	rs1131156	<0.01
HLA-C	rs34546986	<0.01
HLA-DMA	rs433061	<0.01
HLA-DQA1	rs1048645	<0.01
HLA-DRB1	rs9270358	<0.01
HSPA1L	rs2075800	<0.01
INO80E	rs11644809	<0.01
IP6K3	rs2395401	<0.01
IRAK1	rs2266886	<0.01
KLC1	rs10132641	<0.01
LINC00634	rs133382	<0.01
MAN2A1	rs2249413	<0.01
MAPK3	rs11644809	<0.01
MAU2	rs13964	<0.01
MDK	rs11038906	<0.01
MED8	rs3001723	<0.01
MICA	rs2534675	<0.01
NAGA	rs12158335	<0.01
NEK4	rs1029871	<0.01
NOTCH4	rs2534675	<0.01
NT5C2	rs11191560	<0.01
OGFOD2	rs10846491	<0.01
PCCB	rs7349597	<0.01
PGBD1	rs1150670	<0.01
PLXNA3	rs2266886	<0.01
POLR3H	rs1894713	<0.01
PPM1M	rs4687680	<0.01
PPP1R13B	rs10132641	<0.01
PRMT7	rs1971546	<0.01
REEP2	rs16373	<0.01

**Gene**	**SNP**	**P_value**
RFT1	rs7620706	<0.01
RP11-18I14	rs10786701	<0.01
RP11-275H4	rs16832165	<0.01
RP11-347C12	rs11644809	<0.01
RP11-490G2	rs12072739	<0.01
RP11-586K2	rs10955542	<0.01
RP11-677M14	rs36053597	<0.01
RP11-890B15	rs11600182	<0.01
RP1-265C24	rs111770007	<0.01
RP5-874C20	rs1150670	<0.01
RP5-966M1	rs4687680	<0.01
SDAD1P1	rs3808573	<0.01
SF3B1	rs10931779	<0.01
SFMBT1	rs1961958	<0.01
SLC45A1	rs301792	<0.01
SLCO4C1	rs1823694	<0.01
SNX19	rs10791098	<0.01
SNX29P2	rs11644809	<0.01
SRR	rs12603592	<0.01
STAT6	rs324015	<0.01
TAP2	rs433061	<0.01
TDRD9	rs10132641	<0.01
THOC7	rs7615475	<0.01
TMEM187	rs2266886	<0.01
TMEM219	rs4583255	<0.01
TOM1L2	rs9907287	<0.01
TRIM35	rs2565065	<0.01
TSNARE1	rs28460883	<0.01
TYW5	rs11333159	<0.01
UBE2Q2P1	rs57489559	<0.01
VARS2	rs1265092	<0.01
WBP1L	rs10883842	<0.01
WBP2NL	rs12158335	<0.01
WHSC1L1	rs11779986	<0.01
XPNPEP3	rs9611469	<0.01
ZKSCAN3	rs1150670	<0.01
ZKSCAN8	rs2275508	<0.01
ZNF165	rs1150670	<0.01
ZNF192P1	rs111770007	<0.01
ZNF259P1	rs13217795	<0.01
ZSCAN16	rs111770007	<0.01
ZSCAN23	rs111770007	<0.01
ZSCAN31	rs111770007	<0.01

Gene	SNP	P_value
ZSCAN9	rs1150670	<0.01
AC005477	rs2332703	<0.05
AC007163	rs281771	<0.05
ACTR1A	rs3834416	<0.05
ACTR5	rs2244937	<0.05
ANAPC7	rs3026445	<0.05
BTBD1	rs4779046	<0.05
BTN2A2	rs2072803	<0.05
CPT1C	rs57940349	<0.05
CTNNA1	rs154069	<0.05
DHX35	rs208818	<0.05
ERCC8	rs10041978	<0.05
FAM167A	rs2948286	<0.05
FCGRT	rs57940349	<0.05
HIST1H4F	rs198811	<0.05
HLA-E	rs1265092	<0.05
HYAL3	rs9852677	<0.05
IMMP2L	rs37716	<0.05
INA	rs11191560	<0.05
KMT2E	rs10281886	<0.05
L3MBTL2	rs5758268	<0.05
LY6G5B	rs433061	<0.05
MARK3	rs12147596	<0.05
MPHOSPH9	rs1051434	<0.05
NISCH	rs1961958	<0.05
NMB	rs1051168	<0.05
PACSIN3	rs10838634	<0.05
PJA1	rs5937159	<0.05
PLCL1	rs4389332	<0.05
PPT2	rs72849267	<0.05
PSMG3	rs12531315	<0.05
RAB27B	rs12327270	<0.05
RERE	rs159962	<0.05
RP4-756G23	rs5758268	<0.05
SDCCAG8	rs10803138	<0.05
SETD8	rs3759115	<0.05
SNRNP70	rs57940349	<0.05
STAR	rs1488936	<0.05
TARS2	rs35132383	<0.05
TMEM110	rs7620706	<0.05
VPS29	rs11065628	<0.05
VSTM2L	rs208818	<0.05
WDR73	rs11630887	<0.05

**Gene**	**SNP**	**P_value**
YJEFN3	rs1054930	<0.05
ZKSCAN4	rs213238	<0.05
ZNF14	rs1469712	<0.05
ZNF204P	rs111770007	<0.05
ZSCAN2	rs11630887	<0.05

(4) The most significant eQTL analyses results in blood from NG.

Gene	SNP	P_value	FDR
AC013264.2	rs10199410	3.27E-310	0.00E+00
BAK1	rs511515	3.27E-310	0.00E+00
BTN3A2	rs12176317	3.27E-310	0.00E+00
C22orf32	rs12157344	3.27E-310	0.00E+00
C2	rs12157344	3.27E-310	0.00E+00
F2	rs9274563	3.27E-310	0.00E+00
HLA-DQB1-AS1	rs9274563	3.27E-310	0.00E+00
HLA-DQB1	rs9274563	3.27E-310	0.00E+00
HLA-DRB5	rs9271877	3.27E-310	0.00E+00
HLA-DRB6	rs9269874	3.27E-310	0.00E+00
HLA-DRB9	rs9269874	3.27E-310	0.00E+00
ITIH4	rs4687663	3.27E-310	0.00E+00
MUSTN1	rs4687663	3.27E-310	0.00E+00
SNX19	rs2276097	3.27E-310	0.00E+00
XXbac-BPG254F23.6	rs9274563	3.27E-310	0.00E+00
ZFP57	rs3117289	3.27E-310	0.00E+00
HLA-DQA1	rs9274563	3.16E-301	0.00E+00
CYP2D6	rs5751220	2.79E-281	0.00E+00
GPN3	rs11065647	2.93E-271	0.00E+00
ANKRD44	rs10931779	5.89E-255	0.00E+00
RP11-890B15.3	rs2276097	6.88E-246	0.00E+00
HLA-DQB2	rs9269874	1.28E-225	0.00E+00
AS3MT	rs284863	7.23E-223	0.00E+00
HLA-F-AS1	rs3129055	3.65E-212	0.00E+00
HLA-F	rs3129055	3.65E-212	0.00E+00
DUS2L	rs2418736	1.75E-207	0.00E+00
ITIH4-AS1	rs4687663	4.14E-204	0.00E+00
HLA-S	rs1005598	2.31E-202	0.00E+00
HLA-DQA2	rs9269874	1.75E-176	0.00E+00
TMTC1	rs302317	3.69E-173	0.00E+00
XRCC3	rs1799796	7.99E-168	0.00E+00
HLA-C	rs17197637	2.08E-164	0.00E+00
KLC1	rs11846404	7.72E-160	0.00E+00
HCG4P5	rs2571385	4.46E-155	0.00E+00
HCG4	rs2571385	4.46E-155	0.00E+00
HLA-A	rs2571385	4.46E-155	0.00E+00
NT5DC2	rs13076193	6.79E-155	0.00E+00
HLA-DRB1	rs9275109	6.30E-146	0.00E+00
PSMB9	rs9276905	7.07E-145	0.00E+00
RPL23AP1	rs885945	2.81E-135	0.00E+00
FAM109B	rs133379	5.48E-135	0.00E+00

Gene	SNP	P_value	FDR
MAPK3	rs4424923	3.54E-133	0.00E+00
ABCB9	rs883562	2.31E-132	0.00E+00
SREBF1	rs11078400	7.56E-132	0.00E+00
GLYCTK	rs2276834	2.36E-130	0.00E+00
LPAR2	rs7249692	9.85E-130	0.00E+00
INO80E	rs4788197	7.59E-119	0.00E+00
C10orf32	rs284863	9.40E-119	0.00E+00
NT5C2	rs11191560	1.57E-117	0.00E+00
HLA-J	rs1655905	1.29E-116	0.00E+00
ZNRD1-AS1	rs1655905	1.29E-116	0.00E+00
ZNRD1	rs1655905	1.29E-116	0.00E+00
LINC00243	rs886424	1.85E-115	0.00E+00
GLYCTK-AS1	rs2276834	6.68E-114	0.00E+00
MIR135A1	rs2276834	6.68E-114	0.00E+00
HCG27	rs4713446	4.75E-112	0.00E+00
RP11-73M18.10	rs3212090	2.93E-110	0.00E+00
RP11-73M18.7	rs3212090	2.93E-110	0.00E+00
CCDC134	rs9607850	2.49E-108	0.00E+00
HMGN4	rs2073526	9.75E-105	0.00E+00
TRIM27	rs209174	2.28E-104	0.00E+00
ACTR5	rs1006945	2.45E-104	0.00E+00
HLA-U	rs2571385	2.73E-104	0.00E+00
NAGA	rs133379	1.12E-102	0.00E+00
WBP2NL	rs133379	1.12E-102	0.00E+00
RP11-73M18.8	rs11846404	4.19E-101	0.00E+00
HLA-G	rs3132690	3.23E-99	0.00E+00
LY6G5C	rs805303	2.73E-98	0.00E+00
RP11-67A1.2	rs11864819	2.74E-96	0.00E+00
FES	rs35346340	7.95E-96	0.00E+00
CYP21A2	rs519417	1.61E-89	0.00E+00
SETD8	rs2337631	5.47E-86	0.00E+00
HLA-K	rs3132690	3.07E-85	0.00E+00
ALMS1P	rs11903916	3.53E-82	0.00E+00
ALMS1	rs11903916	3.53E-82	0.00E+00
PPM1M	rs11717383	8.11E-82	0.00E+00
TCF19	rs114475699	4.11E-80	0.00E+00
AKAP10	rs72843506	4.78E-79	0.00E+00
PLCL1	rs10207232	5.93E-79	0.00E+00
GDPD3	rs4424923	6.69E-79	0.00E+00
HLA-L	rs3132619	4.99E-77	0.00E+00
RP5-874C20.3	rs1225715	1.51E-76	0.00E+00
PCCB	rs7349597	2.47E-75	0.00E+00
PSORS1C3	rs9380224	1.66E-73	0.00E+00

Gene	SNP	P_value	FDR
RP5-821D11.7	rs9607850	2.45E-73	0.00E+00
SREBF2	rs9607850	2.45E-73	0.00E+00
FAM216A	rs11065647	1.17E-72	0.00E+00
RP11-478C19.5	rs11065647	1.17E-72	0.00E+00
POU5F1	rs114475699	2.81E-70	0.00E+00
IER3	rs886424	2.24E-68	0.00E+00
CCHCR1	rs130076	5.46E-68	0.00E+00
VARS2	rs1632855	6.28E-68	0.00E+00
GATAD2A	rs2905432	1.34E-65	0.00E+00
CDK2AP1	rs12817892	1.61E-65	0.00E+00
RP11-282O18.3	rs4759415	4.04E-65	0.00E+00
TMX2	rs682503	2.27E-64	0.00E+00
NMB	rs11636189	1.84E-62	0.00E+00
PRSS16	rs61240102	3.55E-62	0.00E+00
MOG	rs3117289	1.08E-61	0.00E+00
TRIM38	rs34043431	3.55E-61	0.00E+00
BTN2A2	rs4711107	5.16E-61	0.00E+00
PRMT7	rs1971546	1.37E-60	0.00E+00
YPEL3	rs4788198	2.86E-60	0.00E+00
HLA-DPB2	rs114132738	4.21E-60	0.00E+00
GOLGA6L4	rs220333	8.38E-60	0.00E+00
ZKSCAN4	rs735765	1.39E-59	0.00E+00
LRRFIP2	rs6769400	7.72E-59	0.00E+00
ITIH3	rs2710339	1.21E-58	0.00E+00
CYP2D8P1	rs12157344	6.24E-58	0.00E+00
TMEM180	rs3934495	1.38E-57	0.00E+00
RP5-1157M23.2	rs7629072	2.19E-57	0.00E+00
ANP32E	rs12124898	2.38E-57	0.00E+00
FLOT1	rs886424	1.14E-56	0.00E+00
IGSF9B	rs73034295	1.81E-55	0.00E+00
TBX6	rs4424923	3.20E-55	0.00E+00
ZSCAN12P1	rs2275508	1.35E-53	0.00E+00
RP11-325F22.2	rs6466030	2.80E-52	0.00E+00
MED19	rs682503	3.35E-52	0.00E+00
MUC22	rs114475699	3.57E-52	0.00E+00
HCG4P6	rs3129012	5.92E-52	0.00E+00
ARL6IP4	rs1727295	1.86E-51	0.00E+00
RP11-197N18.2	rs1727295	1.86E-51	0.00E+00
TNXB	rs2280774	2.17E-50	0.00E+00
TNXA	rs389883	5.71E-50	0.00E+00
PSORS1C1	rs9264587	8.60E-50	0.00E+00
PSORS1C2	rs9264587	8.60E-50	0.00E+00
RP11-458I7.4	rs72694957	2.32E-48	0.00E+00

Gene	SNP	P_value	FDR
C4B	rs2280774	2.94E-48	0.00E+00
MIR1307	rs113278154	1.06E-47	0.00E+00
USMG5	rs113278154	1.06E-47	0.00E+00
RP11-332O19.3	rs11191560	1.59E-47	0.00E+00
DPEP3	rs7204208	2.16E-47	0.00E+00
RAD9B	rs3026445	4.35E-47	0.00E+00
SRR	rs216199	2.46E-45	0.00E+00
HLA-V	rs2571385	3.62E-44	0.00E+00
DDHD2	rs2306899	4.14E-44	0.00E+00
TMEM110-MUSTN1	rs9836499	9.26E-44	0.00E+00
TMEM110	rs9836499	9.26E-44	0.00E+00
ZDHHC5	rs682503	1.04E-43	0.00E+00
FTSJ2	rs3778996	1.83E-43	0.00E+00
VPS29	rs3026445	6.75E-43	0.00E+00
DAXX	rs3135406	1.67E-42	0.00E+00
TSNARE1	rs72687376	4.65E-42	0.00E+00
U1	rs9269874	1.20E-41	0.00E+00
GOLGA6L5	rs220333	1.20E-41	0.00E+00
ZBTB22	rs3135406	1.33E-41	0.00E+00
SLC7A6OS	rs6499160	1.75E-41	0.00E+00
SLC7A6	rs6499160	1.75E-41	0.00E+00
HCG9	rs1611350	2.83E-41	0.00E+00
ZSCAN9	rs1225715	3.12E-41	0.00E+00
C4A	rs519417	1.82E-40	0.00E+00
SPECC1	rs72843506	3.70E-40	0.00E+00
ZSCAN2	rs11631921	1.95E-39	0.00E+00
TAP2	rs9271562	3.70E-39	0.00E+00
DOC2A	rs4788198	8.52E-39	0.00E+00
HLA-B	rs2394885	9.79E-39	0.00E+00
HLA-DMA	rs116131925	1.01E-38	0.00E+00
HLA-DMB	rs116131925	1.01E-38	0.00E+00
AL645922.1	rs389883	1.08E-38	0.00E+00
CYP21A1P	rs389883	1.08E-38	0.00E+00
NDRG4	rs113571835	1.44E-38	0.00E+00
BAG5	rs4906336	1.70E-38	0.00E+00
NAB2	rs324017	1.87E-38	0.00E+00
STAT6	rs324017	1.87E-38	0.00E+00
MPHOSPH9	rs7304782	2.27E-38	0.00E+00
Z83851.3	rs134869	1.43E-37	0.00E+00
DDX39BP2	rs1632910	1.47E-37	0.00E+00
DDX39B	rs1632910	1.47E-37	0.00E+00
C4B-AS1	rs2280774	1.55E-37	0.00E+00
C4A-AS1	rs2280774	2.28E-37	0.00E+00

Gene	SNP	P_value	FDR
SKIV2L	rs389883	7.54E-37	0.00E+00
RERE	rs301792	1.99E-36	0.00E+00
MAD1L1	rs6975354	3.43E-36	0.00E+00
ATPAF2	rs7219320	4.65E-36	0.00E+00
BTN2A1	rs2273558	7.45E-36	0.00E+00
NDUFA13	rs2916068	2.15E-35	0.00E+00
TSSK6	rs2916068	2.15E-35	0.00E+00
YJEFN3	rs2916068	2.15E-35	0.00E+00
MEI1	rs9620001	1.39E-34	0.00E+00
TOM1L2	rs11078400	5.86E-34	0.00E+00
LCAT	rs56303487	1.18E-33	0.00E+00
HIST1H4H	rs9358914	2.29E-33	0.00E+00
SDAD1P1	rs3808573	2.62E-33	0.00E+00
SERBP1P3	rs7638524	2.89E-33	0.00E+00
DDAH2	rs622871	3.43E-33	0.00E+00
RP1-265C24.5	rs2275508	4.99E-33	0.00E+00
MICC	rs3132619	9.00E-33	0.00E+00
OR1F12	rs2275508	1.36E-32	0.00E+00
C6orf48	rs805303	1.99E-32	0.00E+00
SNORD52	rs805303	1.99E-32	0.00E+00
DESI1	rs9607850	3.06E-32	0.00E+00
TAPBP	rs3135406	8.24E-32	0.00E+00
BRD2	rs209474	1.85E-31	0.00E+00
XXbac-BPG181M17.6	rs209474	1.85E-31	0.00E+00
CYP2D7P1	rs6002597	3.19E-31	0.00E+00
CSNK2B	rs805303	3.23E-31	0.00E+00
LY6G5B	rs805303	3.23E-31	0.00E+00
PGBD1	rs34130214	3.47E-31	0.00E+00
HLA-H	rs1655905	6.13E-31	0.00E+00
SFXN2	rs4147157	6.46E-31	0.00E+00
HLA-DPA3	rs114132738	7.18E-31	0.00E+00
PRRC2A	rs2736176	1.33E-30	0.00E+00
HCG18	rs3094064	2.44E-30	0.00E+00
RP11-18I14.7	rs2031604	4.22E-30	0.00E+00
BTN3A1	rs3208733	3.36E-29	0.00E+00
MARK3	rs12890820	3.52E-29	0.00E+00
RP11-259P6.1	rs73034295	3.60E-29	0.00E+00
CYP17A1-AS1	rs284863	2.02E-28	0.00E+00
CYP17A1	rs284863	2.02E-28	0.00E+00
ZKSCAN3	rs9468368	2.82E-28	0.00E+00
BTN2A3P	rs12176317	1.02E-27	0.00E+00
MAU2	rs62135552	1.12E-27	0.00E+00
AGER	rs204994	3.91E-27	0.00E+00

Gene	SNP	P_value	FDR
NEK1	rs4434205	5.58E-27	0.00E+00
HLA-W	rs1655905	6.46E-27	0.00E+00
RP11-455F5.4	rs4424923	6.91E-27	0.00E+00
MSL2	rs7349597	7.00E-27	0.00E+00
TRIM10	rs3132651	8.13E-27	0.00E+00
ATF6B	rs519417	9.94E-27	0.00E+00
TRIM31	rs1610752	1.50E-26	0.00E+00
ITPR3	rs756139	3.43E-26	0.00E+00
SF3B1	rs1560277	3.64E-26	0.00E+00
HLA-P	rs3132690	4.92E-26	0.00E+00
PSMD6	rs7627690	5.63E-26	0.00E+00
RP11-245J9.4	rs7627690	5.63E-26	0.00E+00
RP1-97J1.2	rs6921229	7.03E-26	0.00E+00
GPANK1	rs805303	1.22E-25	0.00E+00
ATP6V1G2-DDX39B	rs1005599	1.47E-25	0.00E+00
ATP6V1G2	rs1005599	1.47E-25	0.00E+00
MICE	rs1610622	2.79E-25	0.00E+00
HCG22	rs2246330	4.70E-25	0.00E+00
MICA	rs1005598	5.06E-25	0.00E+00
AIF1	rs805302	9.56E-25	0.00E+00
OGFOD2	rs11608811	3.12E-24	0.00E+00
XXbac-BPG248L24.11	rs2905737	3.78E-24	0.00E+00
MUC21	rs9263970	1.12E-23	0.00E+00
HLA-DPA1	rs114132738	1.88E-23	0.00E+00
HLA-DPB1	rs114132738	1.88E-23	0.00E+00
MCCD1P2	rs1633086	2.77E-23	0.00E+00
MCCD1	rs1633086	2.77E-23	0.00E+00
TDRD9	rs10132641	5.63E-23	0.00E+00
ZSCAN16	rs35345226	8.38E-23	0.00E+00
ATG13	rs55657382	1.13E-22	0.00E+00
GABBR1	rs3117289	1.54E-22	0.00E+00
RP1-265C24.8	rs9393886	1.81E-22	0.00E+00
RP1-265C24.9	rs9393886	1.81E-22	0.00E+00
ZNF165	rs9393886	3.36E-22	0.00E+00
PBX2	rs204994	6.22E-22	0.00E+00
VRK2	rs2465804	8.25E-22	0.00E+00
MCCD1P1	rs1610720	8.26E-22	0.00E+00
HCG11	rs35942482	1.26E-21	0.00E+00
AC073043.2	rs1658810	1.99E-21	0.00E+00
NOTCH4	rs389883	2.20E-21	0.00E+00
ARPC3	rs11065647	2.41E-21	0.00E+00
RP11-478C19.2	rs11065647	2.41E-21	0.00E+00
CTC-479C5.17	rs9922594	3.09E-21	0.00E+00

Gene	SNP	P_value	FDR
SLC12A4	rs9922594	3.09E-21	0.00E+00
PPP1R16B	rs12625702	5.77E-21	0.00E+00
HCG4P8	rs1655905	7.23E-21	0.00E+00
DOPEY1	rs4470825	1.02E-20	0.00E+00
IRF3	rs1045567	1.27E-20	0.00E+00
HIST1H1T	rs198811	1.54E-20	0.00E+00
MICD	rs1611350	2.54E-20	0.00E+00
ALG1L13P	rs2945247	3.33E-20	0.00E+00
AL022393.7	rs1225715	5.63E-20	0.00E+00
VSIG2	rs12293621	6.02E-20	0.00E+00
DPEP2	rs56303487	8.48E-20	0.00E+00
HLA-T	rs3129012	1.07E-19	0.00E+00
TRANK1	rs73068054	3.79E-19	0.00E+00
PSMA4	rs905739	4.21E-19	0.00E+00
CKB	rs58033365	4.36E-19	0.00E+00
SLC17A3	rs1165159	5.12E-19	0.00E+00
MRPS21	rs12124898	9.73E-19	0.00E+00
SGK223	rs2948293	1.18E-18	0.00E+00
HIST1H2BD	rs9358914	1.81E-18	0.00E+00
PCNXL3	rs1193851	3.81E-18	0.00E+00
WHSC1L1	rs11777811	6.27E-18	0.00E+00
CHRNA5	rs905739	2.32E-17	0.00E+00
RP11-650L12.2	rs905739	2.32E-17	0.00E+00
ALAS1	rs352139	4.00E-17	0.00E+00
RP11-753C18.8	rs284863	6.79E-17	0.00E+00
ESRP2	rs9922594	7.50E-17	0.00E+00
NFATC3	rs9922594	7.50E-17	0.00E+00
RP11-96D1.10	rs9922594	7.50E-17	0.00E+00
RP11-96D1.11	rs9922594	7.50E-17	0.00E+00
HLA-DOB	rs4947350	2.10E-16	0.00E+00
BTN3A3	rs6926677	3.82E-16	0.00E+00
NEK4	rs2710323	5.34E-16	0.00E+00
HSPE1	rs3792159	8.08E-16	0.00E+00
SRPK2	rs6968335	1.24E-15	0.00E+00
ZNF592	rs11636499	1.36E-15	0.00E+00
RP11-455F5.3	rs4788197	1.57E-15	0.00E+00
EGFL8	rs2280774	1.59E-15	0.00E+00
STAB1	rs2276834	1.73E-15	0.00E+00
HLA-DRA	rs2002777	2.36E-15	0.00E+00
RP4-669P10.16	rs134885	3.09E-15	0.00E+00
RP4-669P10.18	rs134885	3.09E-15	0.00E+00
APOPT1	rs35498576	3.60E-15	0.00E+00
LEMD2	rs756139	5.88E-15	0.00E+00

Gene	SNP	P_value	FDR
HCG4P11	rs3132690	8.49E-15	0.00E+00
HCG4P7	rs1655905	1.03E-14	0.00E+00
CFB	rs3134940	1.06E-14	0.00E+00
ALDH16A1	rs1045567	1.12E-14	0.00E+00
ZFYVE21	rs2368560	1.69E-14	0.00E+00
GRAP	rs66885728	2.22E-14	0.00E+00
SLC5A10	rs66885728	2.22E-14	0.00E+00
HIST1H3E	rs198811	2.62E-14	0.00E+00
ASPHD1	rs4424923	2.75E-14	0.00E+00
CTD-2574D22.4	rs4424923	2.75E-14	0.00E+00
KCTD13	rs4424923	2.75E-14	0.00E+00
ALKBH5	rs7207461	2.90E-14	0.00E+00
RP11-258F1.1	rs7207461	2.90E-14	0.00E+00
HCG4P3	rs3129012	3.43E-14	0.00E+00
AC092653.5	rs11903916	3.58E-14	0.00E+00
ZNF192P1	rs1225715	3.73E-14	0.00E+00
EPHX2	rs111659883	4.27E-14	0.00E+00
RP11-209D14.2	rs72843506	5.40E-14	0.00E+00
NDUFA6	rs5751241	6.09E-14	0.00E+00
RCN3	rs57940349	6.69E-14	0.00E+00
AC010746.3	rs10931779	7.96E-14	0.00E+00
AC103965.1	rs220333	9.28E-14	0.00E+00
AC136698.1	rs220333	9.28E-14	0.00E+00
ULK2	rs72843506	1.20E-13	0.00E+00
CTA-14H9.5	rs2073526	1.74E-13	0.00E+00
DHFRP2	rs2395476	2.17E-13	0.00E+00
PRRT1	rs3130281	2.19E-13	0.00E+00
AL049840.1	rs10132641	2.52E-13	0.00E+00
SNORD48	rs805303	2.85E-13	0.00E+00
VWA7	rs805303	3.23E-13	0.00E+00
ZSCAN23	rs9885928	3.41E-13	0.00E+00
RP11-513D5.5	rs17435276	5.96E-13	0.00E+00
XXbac-BPG299F13.16	rs2394885	7.08E-13	0.00E+00
ZNF204P	rs61240102	7.19E-13	0.00E+00
MEF2B	rs2905432	9.78E-13	0.00E+00
TSR1	rs2224770	1.14E-12	0.00E+00
DHX35	rs208810	1.18E-12	0.00E+00
APOM	rs2736425	1.36E-12	0.00E+00
TRIM8	rs284862	1.37E-12	0.00E+00
ATXN7	rs7615475	2.20E-12	0.00E+00
DFNA5	rs79210963	3.01E-12	0.00E+00
RP11-600F24.7	rs4906336	3.13E-12	0.00E+00
PITPNM2	rs655293	3.53E-12	0.00E+00

Gene	SNP	P_value	FDR
GLT8D1	rs35249778	3.56E-12	0.00E+00
LRLE1	rs1878561	4.47E-12	0.00E+00
STK19P	rs389883	4.48E-12	0.00E+00
STK19	rs389883	4.48E-12	0.00E+00
PPT2	rs3130281	4.58E-12	0.00E+00
DDX39B-AS1	rs3130621	5.16E-12	0.00E+00
SNORD84	rs3130621	5.16E-12	0.00E+00
AL021917.1	rs9358933	5.60E-12	0.00E+00
GPN1	rs4632296	9.90E-12	0.00E+00
SUPT7L	rs4632296	9.90E-12	0.00E+00
PLEKHO1	rs1824850	1.33E-11	0.00E+00
NEU1	rs1265947	1.46E-11	0.00E+00
HIST1H2BC	rs13196986	1.46E-11	0.00E+00
U6	rs284863	1.59E-11	0.00E+00
HIST1H4K	rs2056924	2.08E-11	0.00E+00
FAM86B3P	rs2948293	2.36E-11	0.00E+00
AC002306.1	rs7249692	2.66E-11	0.00E+00
TRIM26	rs1117488	2.76E-11	0.00E+00
HIST1H4J	rs2056924	3.00E-11	0.00E+00
EGLN1P1	rs17598603	3.36E-11	0.00E+00
RP11-182J1.1	rs17598603	3.36E-11	0.00E+00
SCAND2P	rs17598603	3.36E-11	0.00E+00
BTNL2	rs3132931	4.45E-11	0.00E+00
RBM26-AS1	rs8002042	4.95E-11	0.00E+00
RBM26	rs8002042	4.95E-11	0.00E+00
OR2B8P	rs2275508	6.05E-11	0.00E+00
RP11-196G18.22	rs10888569	6.39E-11	0.00E+00
AKT3	rs3006916	6.64E-11	0.00E+00
SDCCAG8	rs3006916	6.64E-11	0.00E+00
FAM213B	rs2494631	8.63E-11	0.00E+00
MMEL1	rs2494631	8.63E-11	0.00E+00
PSMD6-AS2	rs832195	8.81E-11	0.00E+00
FAM154B	rs75884951	8.91E-11	0.00E+00
PLCH2	rs2494631	8.92E-11	0.00E+00
CTA-250D10.23	rs133348	8.98E-11	0.00E+00
CHRNA2	rs111659883	1.05E-10	0.00E+00
RPL13P	rs3131094	1.25E-10	0.00E+00
XXbac-BPG308K3.6	rs3131094	1.25E-10	0.00E+00
CTD-2574D22.2	rs4424923	1.29E-10	0.00E+00
ANAPC7	rs11065647	1.36E-10	0.00E+00
MICF	rs3128992	1.37E-10	0.00E+00
TSNAXIP1	rs7204208	1.41E-10	0.00E+00
ITIH1	rs4687663	1.64E-10	0.00E+00

Gene	SNP	P_value	FDR
FCGRT	rs10417980	1.98E-10	0.00E+00
RPL13A	rs10417980	2.32E-10	0.00E+00
NRGN	rs36053597	2.63E-10	0.00E+00
PRR12	rs10417980	2.74E-10	0.00E+00
PPP2R3A	rs7349597	4.02E-10	0.00E+00
FANCL	rs2678904	4.98E-10	0.00E+00
RP11-457M11.2	rs13215020	5.04E-10	0.00E+00
ZNF322	rs13215020	5.04E-10	0.00E+00
IFITM4P	rs1610622	5.21E-10	0.00E+00
RP11-499P20.2	rs6482406	6.53E-10	0.00E+00
RFT1	rs9836499	6.58E-10	0.00E+00
ATP13A1	rs7249692	7.19E-10	0.00E+00
NELFE	rs2280774	7.92E-10	6.21E-06
SUGP1	rs2916068	8.10E-10	6.21E-06
FAM83G	rs66885728	9.73E-10	6.18E-06
CLU	rs9331950	1.22E-09	6.15E-06
DNAH1	rs7629072	1.41E-09	6.13E-06
CENPM	rs9620001	1.54E-09	6.12E-06
GULOP	rs111659883	1.56E-09	6.12E-06
ZNF311	rs2765218	1.60E-09	6.11E-06
DDX39BP1	rs1632910	1.65E-09	6.11E-06
SLC4A1AP	rs13001060	1.74E-09	6.10E-06
SLC44A4	rs622871	2.12E-09	6.07E-06
ALPK3	rs3803405	2.61E-09	6.05E-06
MICB	rs2428535	2.80E-09	6.04E-06
HARBI1	rs11038906	3.17E-09	6.02E-06
NHP2L1	rs9607850	3.36E-09	6.01E-06
C12orf65	rs10846491	3.54E-09	6.01E-06
RFTN2	rs11900232	3.57E-09	6.01E-06
ZBTB9	rs3135406	4.53E-09	5.97E-06
HCG9P5	rs1610622	4.83E-09	5.97E-06
MIR378I	rs133348	5.68E-09	1.19E-05
MVP	rs4788197	7.20E-09	1.18E-05
HIST1H3B	rs13196986	7.65E-09	1.18E-05
BCL2L12	rs1045567	7.88E-09	1.77E-05
NCR3	rs2523598	8.81E-09	1.77E-05
GIGYF2	rs2012423	1.01E-08	1.76E-05
SEC11A	rs11637728	1.18E-08	2.34E-05
RPL32P1	rs114132738	1.19E-08	2.34E-05
RPL3	rs114132738	1.19E-08	2.34E-05
PSMB8	rs9276915	1.20E-08	2.34E-05
XXbac-BPG246D15.8	rs9276915	1.20E-08	2.34E-05
HLA-DOA	rs209474	1.35E-08	2.34E-05

Gene	SNP	P_value	FDR
PGM3	rs4470825	1.52E-08	2.33E-05
GTF2H4	rs1110482	1.92E-08	3.48E-05
BAG4	rs4537271	2.58E-08	3.45E-05
LSM1	rs4537271	2.58E-08	3.45E-05
LST1	rs2523554	2.80E-08	4.02E-05
NFKBIL1	rs1800629	2.92E-08	4.02E-05
RP1-59D14.3	rs12943566	3.00E-08	4.59E-05
MGAT3	rs5757717	3.09E-08	4.59E-05
HIST1H1E	rs13196986	3.45E-08	4.57E-05
CTC-228N24.3	rs2764766	4.02E-08	4.56E-05
RP11-325F22.5	rs7807853	5.82E-08	5.08E-05
TNF	rs5996116	6.58E-08	6.20E-05
TNFRSF13C	rs5996116	6.58E-08	6.20E-05
CACNB2	rs6482406	6.85E-08	6.19E-05
MSH5	rs805303	8.33E-08	6.16E-05
MSH5-SAPCD1	rs805303	8.33E-08	6.16E-05
SAPCD1	rs805303	8.33E-08	6.16E-05
FAM53C	rs3756766	9.28E-08	8.37E-05
RP11-256P1.1	rs3756766	9.28E-08	8.37E-05
HIST1H2BO	rs760587	9.70E-08	8.37E-05
DGKZ	rs876701	1.14E-07	8.34E-05
CHRNA3	rs905739	1.25E-07	8.32E-05
YPEL4	rs682503	1.44E-07	8.84E-05
WDR82	rs11717383	1.52E-07	8.83E-05
TOP3A	rs7207461	1.59E-07	9.36E-05
HIST2H2AA4	rs7553622	1.73E-07	9.89E-05
RP11-73M18.9	rs10132641	2.13E-07	1.37E-04
DRG2	rs9895335	2.28E-07	1.58E-04
KDM3B	rs3756766	2.35E-07	1.69E-04
AC011816.1	rs73068054	2.58E-07	1.85E-04
ABT1	rs13215020	2.94E-07	2.12E-04
RP11-671M22.5	rs220333	3.31E-07	2.43E-04
XXbac-BPG116M5.15	rs389883	3.34E-07	2.43E-04
CTD-2134A5.4	rs3212024	3.37E-07	2.43E-04
STAC3	rs61937595	3.47E-07	2.48E-04
CDSN	rs1005598	3.60E-07	2.53E-04
HCG20	rs3131060	3.77E-07	2.69E-04
DDR1	rs2246330	4.01E-07	2.90E-04
HIST1H2AC	rs198811	4.31E-07	3.00E-04
RP11-282O18.6	rs10772999	4.34E-07	3.06E-04
SBNO1	rs10772999	4.34E-07	3.06E-04
RP11-677M14.2	rs36053597	4.57E-07	3.32E-04
PPP1R13B	rs3818085	4.86E-07	3.74E-04

Gene	SNP	P_value	FDR
ARHGAP1	rs12574918	4.96E-07	3.90E-04
GPSM3	rs204993	5.04E-07	4.00E-04
RP5-1115A15.1	rs11121178	5.12E-07	4.06E-04
RP4-669P10.19	rs5751220	5.34E-07	4.32E-04
GID4	rs8068175	5.47E-07	4.47E-04
HCP5	rs3094014	5.53E-07	4.53E-04
AC027228.1	rs905739	5.94E-07	5.10E-04
LRRC48	rs4584886	6.48E-07	5.30E-04
BRD2-IT1	rs209474	6.52E-07	5.30E-04
MIR4640	rs2517472	6.80E-07	5.67E-04
HIST2H2AA3	rs10888569	7.24E-07	6.24E-04
CLCN3	rs4434205	7.29E-07	6.24E-04
RP11-196G18.23	rs10888569	7.42E-07	6.34E-04
RP11-73M18.6	rs2295152	7.68E-07	6.91E-04
SAPCD1-AS1	rs805303	8.97E-07	8.04E-04
RP11-269F20.1	rs3006916	9.42E-07	8.29E-04
FKBPL	rs169504	1.22E-06	1.04E-03
HIST1H4E	rs9358914	1.24E-06	1.05E-03
C2orf69	rs1658810	1.25E-06	1.06E-03
HSPD1	rs12693815	1.26E-06	1.07E-03
CLP1	rs682503	1.31E-06	1.11E-03
LTA	rs2523554	1.39E-06	1.17E-03
ESAM	rs12541	1.40E-06	1.17E-03
PPP1R2P1	rs9275109	1.48E-06	1.22E-03
GPM6A	rs62340589	1.62E-06	1.33E-03
SLC25A5P1	rs5751241	1.62E-06	1.33E-03
GOLGA6L10	rs75884951	1.82E-06	1.43E-03
HSPA1L	rs2075800	1.82E-06	1.44E-03
OR2H2	rs3117289	1.87E-06	1.46E-03
CORO1A	rs4788198	2.03E-06	1.59E-03
TAP1	rs9276905	2.05E-06	1.59E-03
Y_RNA	rs1005598	2.52E-06	1.91E-03
ARL3	rs284862	2.53E-06	1.92E-03
RP11-96D1.5	rs1971546	2.69E-06	2.05E-03
FYN	rs1022649	2.86E-06	2.15E-03
WDR73	rs220333	2.86E-06	2.15E-03
HIST1H2AK	rs35501037	2.90E-06	2.18E-03
SMIM15	rs159538	3.27E-06	2.43E-03
XXbac-BPG16N22.5	rs1005599	4.79E-06	3.36E-03
SFMBT1	rs2581819	5.39E-06	3.82E-03
GNL3	rs2019065	6.26E-06	4.40E-03
RP11-600F24.2	rs35498576	6.41E-06	4.49E-03
SLC17A1	rs501220	6.83E-06	4.77E-03

Gene	SNP	P_value	FDR
AL645941.1	rs209474	7.36E-06	5.04E-03
XXbac-BPG299F13.15	rs17197637	7.51E-06	5.18E-03
TYW5	rs1658810	7.61E-06	5.24E-03
FGFR1	rs17435276	7.62E-06	5.26E-03
RP11-350N15.4	rs17435276	7.62E-06	5.26E-03
MEF2BNB-MEF2B	rs2916068	7.93E-06	5.47E-03
MEF2BNB	rs2916068	7.93E-06	5.47E-03
TCF20	rs134885	8.18E-06	5.63E-03
PIH1D1	rs1045567	8.46E-06	5.85E-03
SUFU	rs12573474	8.93E-06	6.12E-03
HIST1H2BE	rs66972160	9.04E-06	6.19E-03
TRIM15	rs376253	9.91E-06	6.81E-03
PPP1R11	rs2571385	1.16E-05	7.85E-03
RNF39	rs2571385	1.16E-05	7.85E-03
CTB-33G10.11	rs10417980	1.23E-05	8.32E-03
TLR9	rs352139	1.28E-05	8.65E-03
TWF2	rs352139	1.28E-05	8.65E-03
SPCS1	rs35249778	1.69E-05	1.10E-02
CTD-2589M5.5	rs11038864	1.74E-05	1.13E-02
PCGF6	rs116821290	1.74E-05	1.13E-02
RP13-942N8.1	rs10772999	2.17E-05	1.38E-02
SLX1A-SULT1A3	rs4788198	2.20E-05	1.39E-02
SULT1A3	rs4788198	2.20E-05	1.39E-02
AC110781.3	rs7783715	2.48E-05	1.55E-02
HNRNPA1P16	rs216199	2.71E-05	1.68E-02
SMIM4	rs2276834	2.73E-05	1.68E-02
CLIC1	rs805302	3.06E-05	1.87E-02
SMG6	rs8076939	3.15E-05	1.92E-02
MNT	rs9909895	3.18E-05	1.94E-02
RP1-59D14.6	rs9909895	3.18E-05	1.94E-02
CUTA	rs210133	3.38E-05	2.05E-02
PHF1	rs210133	3.38E-05	2.05E-02
CTD-3148I10.9	rs1045567	3.45E-05	2.08E-02
RPS18	rs3135406	3.63E-05	2.19E-02
RP11-457M11.5	rs13215020	3.84E-05	2.31E-02
BTN1A1	rs13215020	4.24E-05	2.52E-02
NKAPL	rs1225715	4.29E-05	2.54E-02
AC073052.1	rs72974219	4.79E-05	2.80E-02
OR2W6P	rs34130214	5.13E-05	2.98E-02
HCG14	rs369136	5.14E-05	2.99E-02
GOLGA6L9	rs75884951	5.23E-05	3.03E-02
RP13-608F4.8	rs75884951	5.23E-05	3.03E-02
HCG25	rs3135406	6.12E-05	3.50E-02

Gene	SNP	P_value	FDR
RP4-778K6.3	rs6968335	6.30E-05	3.59E-02
RILPL1	rs10772999	6.35E-05	3.61E-02
SNRNP35	rs10772999	6.35E-05	3.61E-02
RP1-257I20.13	rs2143139	6.56E-05	3.72E-02
WBP1L	rs4147157	6.86E-05	3.85E-02
HIST1H1PS1	rs198811	6.94E-05	3.90E-02
HIST2H2BD	rs7553622	7.78E-05	4.28E-02
CTC-559E9.6	rs7252981	7.80E-05	4.29E-02
GRAPL	rs66885728	7.83E-05	4.31E-02
SEMA3G	rs11717383	8.94E-05	4.87E-02
APH1A	rs72694955	9.02E-05	4.91E-02

3、pQTL analyses results.

SNP	Protein	Adjusted P
rs3129887	B2M	1.49E-02
rs2442729	C2	1.87E-02
rs3135394		5.66E-03
rs3132931		4.79E-03
rs3096680		1.42E-03
rs3129887		1.14E-03
rs9265998	C4A,C4B	1.92E-02
rs3134792		1.92E-02
rs2442729		1.63E-02
rs1639108		1.34E-02
rs9268659		6.65E-03
rs9271333		5.36E-03
rs2280774		7.54E-04
rs3094005		3.01E-04
rs616187		2.04E-04
rs983561		1.07E-05
rs3129877		1.01E-05
rs3135394		3.48E-06
rs2534679		1.76E-08
rs3129887		1.44E-08
rs389883		3.73E-02
rs9268659		3.26E-02
rs9263927		2.33E-02
rs9276351		1.33E-03
rs2858310		2.47E-05
rs4248168		4.28E-08
rs4713580		1.23E-08
rs2280774		1.08E-24
rs9271333	CD70	3.26E-02
rs2523554	EPHA5	2.75E-02
rs9263927	GZMA	1.19E-04
rs2523554		8.61E-05
rs2678905	GZMB	4.28E-02
rs501220	IFNL2	4.02E-02
rs2523886	LRPAP1	6.59E-03
rs9268659	MICA	3.21E-02
rs760587		2.36E-02
rs3129887		1.36E-02
rs3129055		1.24E-02
rs3117289		7.26E-03
rs2858310		6.46E-03

SNP	Protein	Adjusted P
rs3132505		3.35E-03
rs4248168		1.83E-05
rs3129012		1.73E-05
rs4713580		1.34E-05
rs2523886		7.23E-08
rs3135394		5.18E-08
rs389883		8.09E-09
rs9263927		5.83E-09
rs3094064		4.72E-10
rs3131060		2.93E-10
rs886424		1.96E-10
rs3129984		1.09E-10
rs3094005		1.09E-13
rs9265998		2.15E-14
rs3134792		2.15E-14
rs1639108		3.21E-16
rs2523554		3.96E-21
rs2858310	MICB	4.97E-02
rs2442729		2.22E-02
rs3129887		1.99E-02
rs2523554		1.81E-02
rs3129012		1.75E-02
rs9271333		1.32E-02
rs501220		2.65E-03
rs9276915		1.56E-05
rs9276351		1.65E-06
rs4947350		8.56E-07
rs389883		4.85E-07
rs3094064		6.11E-16
rs2534679		3.09E-17
rs3135394		4.06E-18
rs3129984		1.06E-23
rs3131060		7.09E-24
rs886424		1.08E-24
rs1639108		6.39E-25
rs9265998		4.33E-27
rs3134792		4.33E-27
rs3094005		3.05E-33
rs2280774	MMP8	9.02E-04
rs1639108	MMP9	2.36E-02
rs3094005	PRSS3	4.89E-02
rs3132505		1.73E-02
rs3135394		6.63E-03

SNP	Protein	Adjusted P
rs5004276		4.89E-03
rs2523554	RB1	3.10E-02
rs9276351	ROBO3	7.17E-03
rs4248168	SERPIND1	3.69E-02
rs2056924	TNFRSF14	4.89E-02
