# Supplementary material for: Comprehensive functional annotation of susceptibility SNPs prioritized 10 genes for schizophrenia
Source: Transl Psychiatry. 2019 Jan 31;9:56. doi: 10.1038/s41398-019-0398-5 (PMC6355777; doi:10.1038/s41398-019-0398-5)
Supplement: Supplementary file 9 — supplementary Table S7 [file 41398_2019_398_MOESM9_ESM.pdf]

Table S7. Differential expression of meta analysis between schizophrenia cases and controls.

Gene	N	Zscore	P-value	FDR	Direction
VPS29	513	6.67	2.50E-11	1.08E-08	+-+
BNIP3L	513	5.92	3.31E-09	7.16E-07	+-+
MRPS21	513	5.64	1.67E-08	2.40E-06	+-+
HCFC1	513	5.44	5.43E-08	5.88E-06	+-+
PSMA4	513	5.02	5.29E-07	4.58E-05	+-+
THOC7	513	4.95	7.36E-07	5.31E-05	+-+
SF3B1	513	4.82	1.41E-06	8.10E-05	+-+
NEK1	513	-4.81	1.50E-06	8.10E-05	---
ACTR1A	513	4.68	2.83E-06	1.36E-04	+-+
FAM216A	513	-4.60	4.22E-06	1.83E-04	---
BTBD1	513	-4.57	4.92E-06	1.94E-04	---
TCF12	513	-4.50	6.87E-06	2.48E-04	---
FXR1	513	-4.35	1.36E-05	3.97E-04	---
CHRNA2	513	-4.33	1.47E-05	3.97E-04	---
B2M	513	-4.35	1.38E-05	3.97E-04	---
GRIN2A	513	-4.38	1.21E-05	3.97E-04	---
PBX2	513	-4.32	1.56E-05	3.98E-04	---
BTN3A3	513	-4.27	1.96E-05	4.71E-04	---
TAP1	513	-4.17	3.03E-05	6.91E-04	---
TARS2	410	-4.15	3.35E-05	7.25E-04	-?-
SOX2	513	-4.13	3.63E-05	7.49E-04	---
ETF1	513	-4.07	4.71E-05	9.27E-04	---
PRRT1	410	-4.00	6.34E-05	1.19E-03	-?-
KMT2E	513	-3.98	6.84E-05	1.23E-03	---
MARK3	513	-3.96	7.52E-05	1.25E-03	---
NDUFA6	311	3.97	7.35E-05	1.25E-03	?-+
FGFR1	513	-3.94	8.14E-05	1.26E-03	---
TCF20	513	-3.94	8.00E-05	1.26E-03	---
SRPK2	513	-3.93	8.52E-05	1.27E-03	---
HLA-E	513	-3.89	9.98E-05	1.44E-03	---
BAG4	513	-3.83	1.30E-04	1.81E-03	---
CLU	513	-3.75	1.74E-04	2.35E-03	---
PPP1R11	513	-3.64	2.78E-04	3.65E-03	---
PHF1	513	-3.58	3.47E-04	4.42E-03	---
CCDC134	513	-3.53	4.11E-04	5.08E-03	---
PLCL1	513	-3.52	4.38E-04	5.27E-03	---
GIGYF2	513	-3.51	4.55E-04	5.33E-03	---
DPYD	513	-3.47	5.16E-04	5.88E-03	---
SKIV2L	513	-3.46	5.49E-04	6.09E-03	---
HLA-G	513	-3.45	5.63E-04	6.09E-03	---

Gene	N	Zscore	P-value	FDR	Direction
LRRFIP2	513	-3.44	5.89E-04	6.22E-03	---
ANKRD44	513	-3.39	6.96E-04	7.18E-03	---
DDX39B	410	-3.38	7.24E-04	7.29E-03	-?-
PLA2G15	513	-3.33	8.75E-04	8.61E-03	---
STK19	513	-3.29	9.92E-04	9.54E-03	---
PITPNM2	513	-3.27	1.09E-03	1.01E-02	---
GATAD2A	513	-3.27	1.09E-03	1.01E-02	---
TMTC1	513	-3.24	1.22E-03	1.10E-02	---
NAA10	513	-3.23	1.25E-03	1.10E-02	---
TRIM35	513	-3.22	1.30E-03	1.10E-02	---
MICB	513	-3.22	1.30E-03	1.10E-02	---
TRIM8	513	-3.20	1.38E-03	1.15E-02	---
ZNF592	513	-3.17	1.54E-03	1.26E-02	---
SLC7A6	513	-3.15	1.63E-03	1.29E-02	---
ABCB9	513	-3.15	1.63E-03	1.29E-02	---
DOPEY1	513	-3.14	1.68E-03	1.30E-02	---
HLA-B	513	-3.13	1.77E-03	1.35E-02	---
HLA-C	513	-3.11	1.89E-03	1.41E-02	---
TWF2	513	-3.08	2.06E-03	1.49E-02	---
STAB1	513	-3.08	2.05E-03	1.49E-02	---
RB1	513	-3.07	2.14E-03	1.52E-02	---
ANP32E	513	-3.05	2.31E-03	1.61E-02	---
AS3MT	513	-3.03	2.42E-03	1.63E-02	---
PRMT7	513	-3.04	2.40E-03	1.63E-02	---
HLA-DOA	513	-3.00	2.69E-03	1.79E-02	---
SPATS2L	513	-2.99	2.76E-03	1.81E-02	---
AGPAT1	513	-2.98	2.84E-03	1.84E-02	---
OR2H2	513	-2.98	2.93E-03	1.87E-02	---
FANCL	513	-2.97	3.00E-03	1.88E-02	---
TOM1L2	513	-2.95	3.17E-03	1.96E-02	---
TNF	513	-2.93	3.43E-03	2.03E-02	---
MSH5	202	-2.93	3.38E-03	2.03E-02	-??
GPM6A	513	-2.93	3.39E-03	2.03E-02	---
TNFRSF14	513	-2.91	3.57E-03	2.09E-02	---
NAGA	513	-2.88	3.95E-03	2.28E-02	---
MMP8	513	-2.88	4.03E-03	2.30E-02	---
GABBR1	513	-2.86	4.22E-03	2.35E-02	---
MED19	513	-2.85	4.32E-03	2.35E-02	---
CD46	311	-2.85	4.34E-03	2.35E-02	?--
CTSS	513	-2.86	4.30E-03	2.35E-02	---
ANAPC7	513	-2.85	4.39E-03	2.35E-02	---
BTN2A2	513	-2.81	4.97E-03	2.59E-02	---
PPP1R18	513	-2.81	4.95E-03	2.59E-02	---

Gene	N	Zscore	P-value	FDR	Direction
NISCH	513	-2.80	5.04E-03	2.60E-02	---
AKAP10	513	-2.79	5.23E-03	2.67E-02	---
ARL3	513	-2.75	5.90E-03	2.78E-02	---
DDHD2	513	-2.75	5.95E-03	2.78E-02	---
SYNGAP1	513	-2.76	5.71E-03	2.78E-02	---
SPCS1	513	-2.75	5.97E-03	2.78E-02	---
CACNA1C	513	-2.77	5.58E-03	2.78E-02	---
AIF1	513	-2.77	5.68E-03	2.78E-02	---
MPHOSPH9	513	-2.76	5.86E-03	2.78E-02	---
MAU2	513	-2.76	5.82E-03	2.78E-02	---
ZSCAN16	513	-2.74	6.10E-03	2.81E-02	---
LRPAP1	513	-2.72	6.62E-03	2.84E-02	---
APH1A	513	-2.72	6.61E-03	2.84E-02	---
TMX2	513	-2.72	6.45E-03	2.84E-02	---
HIST1H2AC	513	-2.73	6.32E-03	2.84E-02	---
ARHGAP1	513	2.72	6.51E-03	2.84E-02	+--
YPEL3	513	-2.73	6.36E-03	2.84E-02	---
TRIM26	513	2.73	6.36E-03	2.84E-02	+--
NAB2	513	-2.71	6.79E-03	2.88E-02	---
SGK223	513	2.70	6.91E-03	2.91E-02	+--
MGAT3	513	-2.69	7.08E-03	2.95E-02	---
PLEKHO1	513	-2.69	7.23E-03	2.98E-02	---
ESR2	513	-2.68	7.48E-03	3.06E-02	---
TOP3A	513	-2.66	7.92E-03	3.09E-02	---
IGSF9B	311	2.66	7.87E-03	3.09E-02	?-+
NELFE	410	-2.66	7.72E-03	3.09E-02	-?-
HSPA1A	410	2.66	7.81E-03	3.09E-02	-?+
PLAG1	513	-2.66	7.82E-03	3.09E-02	---
BTN2A1	513	-2.62	8.78E-03	3.39E-02	---
ZSCAN2	513	-2.61	9.04E-03	3.46E-02	---
RCN3	513	-2.61	9.17E-03	3.48E-02	---
CSNK2B	513	-2.60	9.42E-03	3.53E-02	---
HLA-DRB1	513	-2.59	9.62E-03	3.53E-02	---
TNXB	513	-2.59	9.59E-03	3.53E-02	---
ROBO3	513	-2.59	9.55E-03	3.53E-02	---
RNF5	513	2.59	9.73E-03	3.54E-02	+--
C12orf65	513	-2.58	9.88E-03	3.56E-02	---
HIST1H1A	513	-2.57	1.02E-02	3.65E-02	---
GPN3	513	-2.56	1.03E-02	3.67E-02	---
MEI1	513	-2.56	1.05E-02	3.71E-02	---
HIST1H2AK	513	-2.55	1.09E-02	3.82E-02	---
FURIN	513	-2.53	1.15E-02	3.93E-02	---
SNRNP35	513	-2.53	1.15E-02	3.93E-02	---

Gene	N	Zscore	P-value	FDR	Direction
LY6G5C	513	-2.53	1.14E-02	3.93E-02	---
STAT6	513	-2.52	1.17E-02	3.96E-02	---
TSSK6	513	-2.52	1.18E-02	3.97E-02	---
SEPT3	513	-2.51	1.22E-02	4.04E-02	---
ZSCAN23	311	-2.51	1.22E-02	4.04E-02	?--
ALAS1	513	-2.50	1.24E-02	4.06E-02	---
TYW5	513	-2.50	1.25E-02	4.08E-02	---
HIST1H2BD	513	-2.49	1.27E-02	4.10E-02	---
HSPA1L	513	-2.49	1.29E-02	4.15E-02	---
CTNNA1	513	-2.48	1.33E-02	4.21E-02	---
PLCH2	513	-2.48	1.33E-02	4.21E-02	---
VWA7	513	-2.47	1.35E-02	4.23E-02	---
ARL6IP4	513	-2.46	1.37E-02	4.28E-02	---
ZDHHC5	513	-2.46	1.39E-02	4.29E-02	---
BRD2	513	-2.45	1.44E-02	4.42E-02	---
MOG	513	-2.44	1.46E-02	4.44E-02	---
TMEM219	513	-2.44	1.47E-02	4.46E-02	---
PPP1R13B	513	-2.44	1.48E-02	4.46E-02	---
PRR12	513	-2.43	1.51E-02	4.49E-02	---
HLA-DQB1	513	-2.42	1.55E-02	4.57E-02	---
AGER	513	-2.42	1.55E-02	4.57E-02	---
TAP2	513	-2.40	1.63E-02	4.63E-02	---
HIST1H4J	513	-2.40	1.64E-02	4.63E-02	---
PPP1CC	513	2.40	1.63E-02	4.63E-02	+--
MAFG	513	-2.41	1.59E-02	4.63E-02	---
MYC	513	-2.41	1.60E-02	4.63E-02	---
NOTCH4	513	-2.40	1.62E-02	4.63E-02	---
L3MBTL2	513	2.39	1.67E-02	4.71E-02	+--
MAPK3	513	2.38	1.75E-02	4.75E-02	+--
NKAPL	513	-2.38	1.71E-02	4.75E-02	---
MZF1	513	-2.38	1.74E-02	4.75E-02	---
SRR	513	-2.38	1.74E-02	4.75E-02	---
TBX6	513	-2.38	1.75E-02	4.75E-02	---
ZFX	513	-2.38	1.74E-02	4.75E-02	---
C6orf47	513	-2.37	1.78E-02	4.79E-02	---
SMG6	513	-2.36	1.85E-02	4.85E-02	---
GLYCTK	513	-2.36	1.85E-02	4.85E-02	---
SLC12A4	513	-2.36	1.85E-02	4.85E-02	---
FAM213B	311	-2.36	1.85E-02	4.85E-02	?--
BACH1	513	-2.35	1.88E-02	4.90E-02	---
HSPA9	513	2.35	1.90E-02	4.92E-02	--+
ALPK3	513	-2.34	1.92E-02	4.94E-02	---
ATP6V1G2	513	-2.33	1.96E-02	5.02E-02	---

Gene	N	Zscore	P-value	FDR	Direction
APOM	513	-2.33	2.00E-02	5.09E-02	---
ITPR3	513	-2.32	2.05E-02	5.20E-02	---
HSPA1B	202	-2.31	2.07E-02	5.21E-02	-??
PAX5	513	-2.31	2.11E-02	5.27E-02	---
HLA-DPA1	513	-2.29	2.19E-02	5.44E-02	---
GLT8D1	513	-2.29	2.21E-02	5.46E-02	---
TRMT61A	513	-2.28	2.24E-02	5.47E-02	---
SDCCAG8	513	-2.29	2.23E-02	5.47E-02	---
MAN2A1	513	-2.28	2.25E-02	5.48E-02	---
KDM3B	513	-2.27	2.32E-02	5.58E-02	---
VARS2	513	-2.27	2.31E-02	5.58E-02	---
EPHA5	513	-2.26	2.41E-02	5.76E-02	---
C2	513	-2.25	2.48E-02	5.86E-02	---
DRD2	513	-2.25	2.47E-02	5.86E-02	---
KLF5	513	-2.23	2.56E-02	5.93E-02	---
BAK1	513	-2.24	2.53E-02	5.93E-02	---
SP2	513	2.23	2.58E-02	5.93E-02	+--
TNFRSF13C	513	-2.23	2.55E-02	5.93E-02	---
CYP2D6	513	-2.24	2.53E-02	5.93E-02	---
WHSC1L1	513	-2.23	2.59E-02	5.93E-02	---
CYP17A1	513	-2.22	2.68E-02	6.03E-02	---
ZNF354C	513	-2.22	2.66E-02	6.03E-02	---
CLP1	513	-2.22	2.66E-02	6.03E-02	---
C2orf69	513	-2.20	2.79E-02	6.27E-02	---
ATXN7	513	-2.18	2.90E-02	6.47E-02	---
BTN3A2	513	-2.18	2.97E-02	6.58E-02	---
SUGP1	311	-2.17	3.01E-02	6.58E-02	?--
LCAT	513	-2.17	3.01E-02	6.58E-02	---
FCGRT	513	-2.17	2.98E-02	6.58E-02	---
TRANK1	513	2.16	3.11E-02	6.76E-02	+--
ZNRD1	513	-2.14	3.21E-02	6.94E-02	---
ALDH16A1	513	-2.12	3.41E-02	7.30E-02	---
DNAH1	513	-2.12	3.39E-02	7.30E-02	---
TDRD9	513	-2.12	3.44E-02	7.33E-02	---
EHF	513	-2.11	3.51E-02	7.46E-02	---
PACSIN3	513	-2.10	3.54E-02	7.48E-02	---
RENBP	513	2.10	3.57E-02	7.49E-02	+--
SCGN	513	-2.08	3.72E-02	7.76E-02	---
NT5DC2	513	-2.08	3.76E-02	7.76E-02	---
IMMP2L	513	-2.08	3.74E-02	7.76E-02	---
GZMB	513	-2.08	3.76E-02	7.76E-02	---
HLA-DRA	513	-2.08	3.80E-02	7.80E-02	---
SREBF2	513	-2.05	4.08E-02	8.33E-02	---

Gene	N	Zscore	P-value	FDR	Direction
IRF3	513	2.03	4.19E-02	8.52E-02	+--
RPS18	513	2.03	4.26E-02	8.62E-02	+--
C16orf92	513	-2.02	4.39E-02	8.72E-02	---
ATP13A1	513	-2.02	4.37E-02	8.72E-02	---
C6orf10	513	-2.02	4.34E-02	8.72E-02	---
USF2	513	2.02	4.38E-02	8.72E-02	--+
RFT1	513	2.00	4.54E-02	8.97E-02	--+
PRRC2A	513	2.00	4.56E-02	8.97E-02	+--
NT5C2	513	-1.99	4.62E-02	9.05E-02	---
HCG27	513	-1.99	4.70E-02	9.17E-02	---
RXRA	513	-1.96	4.96E-02	9.63E-02	---
RNF39	513	-1.96	4.98E-02	9.63E-02	---
CDC25C	513	-1.95	5.08E-02	9.77E-02	---
BAG5	513	-1.95	5.18E-02	9.91E-02	---
TRIM27	513	-1.94	5.27E-02	1.00E-01	---
WDR73	513	-1.93	5.33E-02	1.01E-01	---
HIST1H2BL	513	-1.93	5.37E-02	1.02E-01	---
EMX1	513	-1.93	5.42E-02	1.02E-01	---
STAR	513	-1.93	5.40E-02	1.02E-01	---
TRIM10	513	-1.92	5.54E-02	1.03E-01	---
CACNA2D2	513	1.91	5.56E-02	1.03E-01	+--
ZSCAN31	513	-1.92	5.55E-02	1.03E-01	---
BCL11B	513	-1.91	5.61E-02	1.03E-01	---
DGKZ	513	-1.91	5.66E-02	1.04E-01	---
INO80E	513	-1.90	5.78E-02	1.05E-01	---
NEU1	513	-1.90	5.80E-02	1.05E-01	---
ATF6B	513	-1.89	5.82E-02	1.05E-01	---
PBRM1	311	-1.89	5.86E-02	1.06E-01	?--
HIST1H4H	513	1.89	5.91E-02	1.06E-01	--+
CFB	513	-1.88	5.96E-02	1.07E-01	---
NFKBIL1	513	-1.88	6.01E-02	1.07E-01	---
SFXN2	513	-1.88	6.08E-02	1.08E-01	---
ITIH1	513	-1.87	6.09E-02	1.08E-01	---
PPT2	410	-1.85	6.38E-02	1.12E-01	-?-
CCHCR1	513	-1.83	6.78E-02	1.19E-01	---
RAD9B	513	-1.82	6.86E-02	1.20E-01	---
NFE2L1	513	1.81	7.00E-02	1.22E-01	+--
CNNM2	513	-1.80	7.19E-02	1.25E-01	---
LTA	513	-1.79	7.31E-02	1.25E-01	---
HLA-DMB	513	-1.80	7.26E-02	1.25E-01	---
HLA-DPB1	513	-1.79	7.29E-02	1.25E-01	---
DPEP2	513	-1.78	7.46E-02	1.27E-01	---
PSMB8	513	-1.78	7.47E-02	1.27E-01	---

Gene	N	Zscore	P-value	FDR	Direction
DRG2	513	1.78	7.51E-02	1.27E-01	+--
ESR1	513	-1.77	7.65E-02	1.28E-01	---
HLA-F	513	-1.77	7.64E-02	1.28E-01	---
NFKB1	513	-1.76	7.78E-02	1.30E-01	---
YJEFN3	513	-1.75	8.00E-02	1.33E-01	---
CUTA	513	1.75	8.04E-02	1.33E-01	--+
LHFPL3	513	-1.73	8.32E-02	1.36E-01	---
IP6K3	513	-1.73	8.31E-02	1.36E-01	---
POLR3H	513	1.73	8.37E-02	1.36E-01	+--
FAM83G	513	-1.73	8.36E-02	1.36E-01	---
TCF19	513	-1.73	8.37E-02	1.36E-01	---
FAM53C	513	-1.73	8.45E-02	1.37E-01	---
OR2J2	513	-1.72	8.59E-02	1.39E-01	---
ZBTB9	513	-1.72	8.64E-02	1.39E-01	---
GPSM3	513	1.71	8.75E-02	1.40E-01	+--
POU5F1	202	-1.69	9.16E-02	1.45E-01	-??
HARBI1	513	-1.69	9.15E-02	1.45E-01	---
SAPCD1	410	-1.69	9.12E-02	1.45E-01	-?-
DOC2A	513	-1.68	9.31E-02	1.47E-01	---
FLI1	513	-1.68	9.34E-02	1.47E-01	---
ATPAF2	513	-1.67	9.51E-02	1.49E-01	---
TFAP2C	513	-1.67	9.53E-02	1.49E-01	---
TMEM110	513	-1.66	9.72E-02	1.51E-01	---
SATB2	513	1.65	9.91E-02	1.54E-01	--+
ZSCAN9	513	-1.64	1.01E-01	1.54E-01	---
OGFOD2	513	-1.64	1.02E-01	1.54E-01	---
CLIC1	513	1.64	1.01E-01	1.54E-01	+--
DPCR1	513	-1.65	1.00E-01	1.54E-01	---
HIST1H2AJ	513	-1.64	1.00E-01	1.54E-01	---
SREBF1	513	1.64	1.01E-01	1.54E-01	+--
HLA-DQA2	202	-1.63	1.03E-01	1.56E-01	-??
HIST1H2BJ	513	-1.63	1.04E-01	1.57E-01	---
CILP2	513	-1.62	1.04E-01	1.57E-01	---
PSORS1C1	513	-1.62	1.06E-01	1.59E-01	---
GZMA	513	-1.60	1.09E-01	1.62E-01	---
DUS2	513	-1.60	1.09E-01	1.62E-01	---
PEX10	513	-1.60	1.09E-01	1.62E-01	---
ZNF263	410	-1.60	1.10E-01	1.62E-01	-?-
CNTN4	513	-1.60	1.10E-01	1.62E-01	---
BTN1A1	513	-1.60	1.10E-01	1.62E-01	---
FLOT1	513	1.59	1.13E-01	1.64E-01	--+
WBP1L	513	1.59	1.13E-01	1.64E-01	+--
DDAH2	513	-1.56	1.18E-01	1.72E-01	---

Gene	N	Zscore	P-value	FDR	Direction
C2orf82	513	-1.56	1.19E-01	1.73E-01	---
GDPD3	513	-1.54	1.23E-01	1.78E-01	---
ZKSCAN8	513	-1.54	1.25E-01	1.79E-01	---
CPT1C	513	-1.52	1.28E-01	1.84E-01	---
TAPBP	513	1.52	1.29E-01	1.84E-01	+--
SLCO4C1	513	-1.52	1.29E-01	1.84E-01	---
TSNARE1	513	-1.52	1.29E-01	1.84E-01	---
HIST1H4E	513	1.51	1.31E-01	1.85E-01	--+
GNL3	513	1.49	1.36E-01	1.92E-01	+--
NEK4	513	-1.48	1.39E-01	1.95E-01	---
HAPLN4	513	-1.47	1.41E-01	1.97E-01	---
ZFYVE21	513	-1.47	1.42E-01	1.98E-01	---
NMUR2	513	-1.47	1.43E-01	1.99E-01	---
ACTR5	513	-1.46	1.44E-01	1.99E-01	---
GPANK1	513	-1.46	1.45E-01	2.00E-01	---
LST1	513	-1.45	1.47E-01	2.01E-01	---
CENPM	513	-1.45	1.46E-01	2.01E-01	---
HIST1H3I	513	-1.45	1.47E-01	2.01E-01	---
MAFK	513	-1.45	1.48E-01	2.02E-01	---
HIST1H1T	513	-1.45	1.48E-01	2.02E-01	---
HIST1H1B	513	-1.44	1.49E-01	2.02E-01	---
KLF1	513	-1.44	1.50E-01	2.02E-01	---
CLCN3	513	1.44	1.50E-01	2.03E-01	--+
DAXX	513	1.44	1.51E-01	2.03E-01	+--
CD70	513	-1.43	1.51E-01	2.03E-01	---
DESI1	513	-1.42	1.55E-01	2.06E-01	---
IER3	513	-1.42	1.56E-01	2.07E-01	---
GALNT10	513	-1.42	1.55E-01	2.07E-01	---
NCR3	513	1.42	1.56E-01	2.07E-01	+--
HLA-DOB	513	-1.42	1.57E-01	2.07E-01	---
FTCDNL1	311	-1.40	1.61E-01	2.12E-01	?--
ATG13	513	-1.40	1.62E-01	2.13E-01	---
CPEB1	513	-1.40	1.63E-01	2.13E-01	---
DPEP3	513	-1.39	1.65E-01	2.15E-01	---
MNT	513	1.37	1.71E-01	2.22E-01	+--
SFMBT1	513	-1.36	1.73E-01	2.24E-01	---
LSM1	513	1.35	1.77E-01	2.29E-01	+--
IFNL2	513	-1.32	1.85E-01	2.38E-01	---
HHAT	513	-1.33	1.85E-01	2.38E-01	---
MDK	513	1.31	1.89E-01	2.42E-01	+--
SNX19	513	-1.31	1.91E-01	2.43E-01	---
TMEM243	513	1.31	1.91E-01	2.43E-01	+--
FKBPL	513	-1.30	1.93E-01	2.45E-01	---

Gene	N	Zscore	P-value	FDR	Direction
ETS1	513	-1.30	1.95E-01	2.47E-01	---
HIST1H1E	513	-1.28	2.00E-01	2.53E-01	---
SLC44A4	513	1.27	2.04E-01	2.57E-01	+--
HIST1H3E	311	-1.25	2.10E-01	2.64E-01	?--
PGBD1	513	-1.24	2.14E-01	2.68E-01	---
GTF2H4	513	-1.24	2.16E-01	2.69E-01	---
ZKSCAN4	513	-1.22	2.23E-01	2.78E-01	---
AP3B2	513	-1.21	2.26E-01	2.80E-01	---
HLA-DMA	513	-1.21	2.26E-01	2.80E-01	---
ZNF165	513	-1.19	2.34E-01	2.89E-01	---
ITIH4	513	-1.18	2.38E-01	2.93E-01	---
SPECC1	513	-1.18	2.40E-01	2.94E-01	---
NR1H3	513	-1.17	2.43E-01	2.97E-01	---
USF1	513	-1.16	2.47E-01	3.00E-01	---
BTN3A1	513	1.16	2.46E-01	3.00E-01	+--
MAD1L1	513	-1.15	2.50E-01	3.03E-01	---
ABT1	513	-1.15	2.52E-01	3.03E-01	---
FAM109B	513	-1.15	2.52E-01	3.03E-01	---
PPP1R16B	513	1.14	2.54E-01	3.05E-01	+--
ERCC8	513	1.12	2.63E-01	3.16E-01	+--
KCTD13	513	1.11	2.65E-01	3.17E-01	+--
PCCB	513	1.09	2.77E-01	3.30E-01	--+
PRSS3	513	1.08	2.82E-01	3.36E-01	--+
MICA	305	-1.07	2.83E-01	3.36E-01	--?
PPP4C	311	-1.07	2.86E-01	3.38E-01	?--
HIST1H3B	513	-1.05	2.93E-01	3.45E-01	---
SLC45A1	513	-1.03	3.01E-01	3.54E-01	---
OR12D2	513	-1.01	3.14E-01	3.68E-01	---
MLN	513	-0.99	3.21E-01	3.76E-01	---
ZSCAN12	311	-0.96	3.37E-01	3.93E-01	?--
FES	513	-0.95	3.40E-01	3.96E-01	---
C4B	202	-0.94	3.45E-01	3.99E-01	-??
ZBTB22	311	-0.95	3.44E-01	3.99E-01	?--
HIST1H2BC	513	-0.94	3.46E-01	4.00E-01	---
PRRG2	513	-0.92	3.58E-01	4.12E-01	---
SHISA8	513	-0.90	3.69E-01	4.23E-01	---
CACNB2	513	0.90	3.71E-01	4.25E-01	--+
LEMD2	513	-0.89	3.75E-01	4.28E-01	---
HLA-A	513	0.88	3.81E-01	4.34E-01	+--
NGEF	513	-0.87	3.84E-01	4.37E-01	---
ZKSCAN3	513	-0.87	3.86E-01	4.37E-01	---
ARPC3	513	0.85	3.93E-01	4.44E-01	--+
UBD	202	-0.83	4.05E-01	4.57E-01	-??

Gene	N	Zscore	P-value	FDR	Direction
MYO15A	513	-0.83	4.07E-01	4.58E-01	---
CYP21A2	410	-0.81	4.16E-01	4.66E-01	-?-
TRIM38	513	0.80	4.21E-01	4.71E-01	+--
HLA-DQA1	311	-0.79	4.30E-01	4.80E-01	?--
KLF4	513	-0.78	4.34E-01	4.83E-01	---
RAB27B	513	0.76	4.46E-01	4.95E-01	+--
INA	513	-0.76	4.47E-01	4.95E-01	---
XPNPEP3	513	0.75	4.55E-01	5.02E-01	+--
C6orf15	513	-0.75	4.55E-01	5.02E-01	---
SLC4A1AP	513	0.74	4.59E-01	5.05E-01	--+
PRSS16	513	-0.73	4.65E-01	5.09E-01	---
SLC17A2	513	-0.72	4.73E-01	5.18E-01	---
CSDC2	513	-0.71	4.77E-01	5.20E-01	---
NFATC3	513	0.69	4.90E-01	5.33E-01	+--
NDUFA13	305	-0.68	4.95E-01	5.38E-01	--?
BTNL2	311	-0.68	4.98E-01	5.39E-01	?--
SERPIND1	513	-0.67	5.05E-01	5.44E-01	---
ITIH3	513	-0.67	5.05E-01	5.44E-01	---
DFNA5	513	-0.66	5.11E-01	5.49E-01	---
AKT3	513	0.65	5.16E-01	5.51E-01	--+
MMP9	513	-0.65	5.16E-01	5.51E-01	---
SFTA2	513	-0.64	5.20E-01	5.55E-01	---
WBP2NL	513	-0.62	5.33E-01	5.67E-01	---
MYOG	513	-0.60	5.48E-01	5.81E-01	---
VSTM2L	513	-0.59	5.53E-01	5.85E-01	---
KLC1	513	0.53	5.93E-01	6.26E-01	--+
HIST1H4K	202	-0.53	5.97E-01	6.29E-01	-??
NMB	513	-0.52	6.00E-01	6.31E-01	---
JUND	513	0.51	6.14E-01	6.43E-01	+--
PPM1M	513	-0.50	6.15E-01	6.43E-01	---
ZFP57	513	-0.49	6.28E-01	6.55E-01	---
REEP2	513	-0.44	6.58E-01	6.84E-01	---
POM121L2	311	-0.44	6.61E-01	6.86E-01	?--
C6orf48	513	0.43	6.67E-01	6.91E-01	+--
C4A	202	-0.39	6.97E-01	7.20E-01	-??
EGFL8	202	-0.34	7.35E-01	7.58E-01	-??
HIST1H3C	513	-0.33	7.41E-01	7.61E-01	---
RERE	513	0.33	7.41E-01	7.61E-01	+--
APOPT1	513	-0.21	8.34E-01	8.54E-01	--+
ALMS1	513	0.20	8.39E-01	8.57E-01	--+
EGR1	513	-0.20	8.41E-01	8.57E-01	--+
DDR1	410	0.17	8.63E-01	8.77E-01	+?-
C2orf47	513	0.14	8.85E-01	8.98E-01	+--

Gene	N	Zscore	P-value	FDR	Direction
FOXP1	513	-0.13	8.94E-01	9.04E-01	+--
LY6G5B	202	-0.11	9.11E-01	9.19E-01	-??
CDK2AP1	311	-0.10	9.22E-01	9.28E-01	?--
CUL3	513	0.08	9.40E-01	9.42E-01	--+
PSMG3	513	0.08	9.39E-01	9.42E-01	--+
BAG6	513	-0.04	9.72E-01	9.72E-01	+--

NOTE: N means the number of subjects in meta analyses.
