# Supplementary material for: Comprehensive functional annotation of susceptibility SNPs prioritized 10 genes for schizophrenia
Source: Transl Psychiatry. 2019 Jan 31;9:56. doi: 10.1038/s41398-019-0398-5 (PMC6355777; doi:10.1038/s41398-019-0398-5)
Supplement: Supplementary file 10 — supplementary Table S8 [file 41398_2019_398_MOESM10_ESM.doc]

Table S8. Target genes related to nervous phenotype in mouse models.

| **Gene** | **Phenotype** |
| --- | --- |
| *GPSM3* | nervous; nervous system phenotype |
| *KLC1* | nervous; nervous system phenotype |
| *ESR1* | nervous; nervous system phenotype |
| *ESR2* | nervous; nervous system phenotype |
| *RAB27B* | nervous; nervous system phenotype |
| *SATB2* | nervous; nervous system phenotype |
| *MAPK3* | nervous; nervous system phenotype |
| *DAXX* | nervous; nervous system phenotype |
| *CHRNA2* | nervous; nervous system phenotype |
| *FLI1* | nervous; nervous system phenotype |
| *PLCL1* | nervous; nervous system phenotype; abnormal nervous system electrophysiology |
| *DRD2* | nervous; nervous system phenotype; abnormal nervous system electrophysiology |
| *NGEF* | nervous; nervous system phenotype |
| *MDK* | nervous; nervous system phenotype |
| *RERE* | nervous; nervous system phenotype |
| *USF1* | nervous; nervous system phenotype |
| *USF2* | nervous; nervous system phenotype |
| *APH1A* | nervous; nervous system phenotype |
| *EMX1* | nervous; nervous system phenotype; abnormal nervous system electrophysiology; abnormal nervous system tract morphology |
| *LTA* | nervous; nervous system phenotype |
| *MYC* | nervous; nervous system phenotype |
| *CLP1* | abnormal phrenic nerve innervation pattern to diaphragm; nervous; nervous system phenotype; abnormal sciatic nerve morphology; defasiculated phrenic nerve |
| *ERCC8* | nervous; nervous system phenotype |
| *GABBR1* | nervous; nervous system phenotype; abnormal somatic nervous system morphology; abnormal sciatic nerve morphology; abnormal nervous system electrophysiology |
| *DDHD2* | nervous; nervous system phenotype |
| *HHAT* | nervous; nervous system phenotype |
| *FLOT1* | nervous; nervous system phenotype |
| *CLCN3* | abnormal nervous system physiology; abnormal nervous system morphology; nervous; nervous system phenotype |
| *SREBF2* | nervous; nervous system phenotype |
| *LRPAP1* | nervous; nervous system phenotype |
| *CACNA2D2* | nervous; nervous system phenotype; abnormal nervous system electrophysiology; abnormal spinal nerve morphology |
| *MYO15A* | abnormal cochlear nerve compound action potential; nervous; nervous system phenotype |
| *FES* | nervous; nervous system phenotype |
| *NAB2* | nervous; nervous system phenotype |
| *GATAD2A* | nervous; nervous system phenotype |
| *IP6K3* | abnormal nervous system physiology; nervous; nervous system phenotype |
| *SP2* | nervous; nervous system phenotype |
| *HLA-A* | nervous; nervous system phenotype |
| *PAX5* | nervous; nervous system phenotype |
| *MAFG* | nervous; nervous system phenotype |
| *MAFK* | nervous; nervous system phenotype |
| *CPEB1* | nervous; nervous system phenotype |
| *EPHA5* | nervous; nervous system phenotype |
| *AIF1* | nervous; nervous system phenotype |
| *DOC2A* | nervous; nervous system phenotype |
| *HLA-DQB1* | nervous; nervous system phenotype |
| *BAG6* | nervous; nervous system phenotype |
| *PPT2* | nervous; nervous system phenotype |
| *NEK1* | nervous; nervous system phenotype |
| *ROBO3* | nervous; nervous system phenotype |
| *HLA-DOA* | nervous; nervous system phenotype |
| *ARHGAP1* | nervous; nervous system phenotype |
| *BAK1* | nervous; nervous system phenotype |
| *AKT3* | nervous; nervous system phenotype |
| *CNTN4* | abnormal olfactory nerve morphology; nervous; nervous system phenotype |
| *NOTCH4* | nervous; nervous system phenotype |
| *TBX6* | nervous; nervous system phenotype |
| *FURIN* | nervous; nervous system phenotype |
| *SYNGAP1* | nervous; nervous system phenotype |
| *NR1H3* | nervous; nervous system phenotype |
| *GNL3* | nervous; nervous system phenotype |
| *RB1* | increased nervous system tumor incidence; nervous; nervous system phenotype |
| *EGFL8* | nervous; nervous system phenotype |
| *PEX10* | nervous; nervous system phenotype |
| *CD46* | nervous; nervous system phenotype |
| *SRR* | abnormal nervous system physiology; nervous; nervous system phenotype |
| *GLT8D1* | nervous; nervous system phenotype |
| *RXRA* | nervous; nervous system phenotype |
| *RAD9B* | nervous; nervous system phenotype |
| *MMP9* | nervous; nervous system phenotype |
| *BRD2* | nervous; nervous system phenotype |
| *NFATC3* | nervous; nervous system phenotype; abnormal trigeminal nerve morphology |
| *NEU1* | nervous; nervous system phenotype |
| *ARL3* | nervous; nervous system phenotype |
| *GIGYF2* | nervous; nervous system phenotype |
| *AP3B2* | abnormal nervous system physiology; nervous; nervous system phenotype |
| *ARPC3* | nervous; nervous system phenotype |
| *AKAP10* | nervous; nervous system phenotype |
| *ATXN7* | nervous; nervous system phenotype |
| *CPT1C* | nervous; nervous system phenotype |
| *SDCCAG8* | nervous; nervous system phenotype |
| *TNF* | abnormal nervous system physiology; nervous; nervous system phenotype |
| *SOX2* | optic nerve hypoplasia; decreased cochlear nerve compound action potential; absent cochlear nerve compound action potential; abnormal nervous system physiology; abnormal nervous system morphology; nervous; nervous system phenotype |
| *B2M* | nervous; nervous system phenotype |
| *CACNB2* | nervous; nervous system phenotype |
| *HIST1H2BJ* | nervous; nervous system phenotype |
| *TOM1L2* | nervous; nervous system phenotype |
| *NFKB1* | decreased cochlear nerve compound action potential; nervous; nervous system phenotype |
| *GPM6A* | nervous; nervous system phenotype |
| *ITPR3* | nervous; nervous system phenotype |
| *EGR1* | nervous; nervous system phenotype |
| *MOG* | abnormal nervous system morphology; nervous; nervous system phenotype; abnormal optic nerve morphology |
| *TCF12* | nervous; nervous system phenotype |
| *CSNK2B* | nervous; nervous system phenotype |
| *INA* | nervous; nervous system phenotype |
| *GRIN2A* | nervous; nervous system phenotype |
| *LEMD2* | nervous; nervous system phenotype |
| *TAP1* | nervous; nervous system phenotype |
| *CLU* | nervous; nervous system phenotype |
| *FGFR1* | abnormal cochlear nerve compound action potential; nervous; nervous system phenotype; abnormal trochlear nerve morphology |
| *HSPA1B* | nervous; nervous system phenotype |
| *CACNA1C* | abnormal nervous system physiology; nervous; nervous system phenotype |
| *CTNNA1* | nervous; nervous system phenotype |
