# Supplementary material for: Comprehensive functional annotation of susceptibility SNPs prioritized 10 genes for schizophrenia
Source: Transl Psychiatry. 2019 Jan 31;9:56. doi: 10.1038/s41398-019-0398-5 (PMC6355777; doi:10.1038/s41398-019-0398-5)
Supplement: Supplementary file 11 — supplementary Table S9 [file 41398_2019_398_MOESM11_ESM.doc]

Table S9. The list of the contributing SNPs associated with each of the prioritized genes.

1、The most significant SMR analyses results in whole blood from GTEx.

| **Gene** | **topSNP** | **p_eQTL** | **p_SMR** | **adjp_SMR** |
| --- | --- | --- | --- | --- |
| *CSNK2B* | rs2075800 | 6.08E-04 | 2.13E-03 | 9.88E-03 |
|  | rs2857694 | 1.36E-05 | 1.81E-04 | 1.72E-03 |
| *GABBR1* | rs3129055 | 3.32E-07 | 4.11E-05 | 6.19E-04 |
|  | rs885945 | 4.65E-06 | 1.17E-04 | 1.61E-03 |
| *MAPK3* | rs4424923 | 2.15E-20 | 2.44E-07 | 1.82E-05 |
| *SRR* | rs11078018 | 1.16E-03 | 4.40E-03 | 1.47E-02 |
| *SYNGAP1* | rs9276915 | 3.68E-03 | 9.28E-03 | 2.29E-02 |
|  | rs9394145 | 1.40E-02 | 2.91E-02 | 4.32E-02 |
| *TNF* | rs1800629 | 1.85E-02 | 2.32E-02 | 3.69E-02 |

2、The most significant SMR analyses results in brain tissues from GTEx.

| **Gene** | **topSNP** | **p_eQTL** | **p_SMR** | **adjp_SMR** |
| --- | --- | --- | --- | --- |
| *CACNA1C* | rs11062157 | 2.44E-04 | 1.64E-03 | 3.61E-02 |
|  | rs11062170 | 4.08E-08 | 3.30E-06 | 2.09E-04 |
| *CSNK2B* | rs1265754 | 4.43E-03 | 5.81E-03 | 1.92E-02 |
|  | rs1800629 | 1.30E-02 | 1.71E-02 | 3.98E-02 |
|  | rs204993 | 1.37E-02 | 2.02E-02 | 4.21E-02 |
|  | rs2075800 | 1.10E-03 | 2.88E-03 | 1.67E-02 |
|  | rs2736176 | 2.18E-03 | 4.61E-03 | 1.93E-02 |
|  | rs2857694 | 4.82E-03 | 8.48E-03 | 3.44E-02 |
|  | rs433061 | 4.43E-03 | 6.97E-03 | 2.45E-02 |
|  | rs9262696 | 8.07E-03 | 1.64E-02 | 4.30E-02 |
|  | rs9263473 | 3.05E-03 | 7.59E-03 | 2.87E-02 |
|  | rs9268142 | 6.96E-03 | 9.98E-03 | 3.36E-02 |
| *GABBR1* | rs1063320 | 2.30E-03 | 7.61E-03 | 2.79E-02 |
|  | rs1610720 | 1.37E-03 | 3.16E-03 | 1.72E-02 |
|  | rs1610721 | 1.09E-03 | 2.73E-03 | 1.79E-02 |
|  | rs1611204 | 1.81E-03 | 3.88E-03 | 1.70E-02 |
| *MAPK3* | rs4424923 | 1.39E-05 | 3.68E-04 | 6.03E-03 |
| *NOTCH4* | rs1003878 | 1.89E-05 | 1.57E-04 | 1.64E-03 |
|  | rs204994 | 1.92E-06 | 2.17E-05 | 8.49E-04 |
|  | rs3130281 | 3.81E-03 | 5.44E-03 | 2.75E-02 |
|  | rs433061 | 7.77E-05 | 3.40E-04 | 3.56E-03 |
|  | rs5021453 | 5.24E-03 | 8.51E-03 | 3.73E-02 |
|  | rs9266209 | 1.75E-02 | 2.37E-02 | 4.36E-02 |
|  | rs9268142 | 6.46E-03 | 9.37E-03 | 3.38E-02 |
| *SRR* | rs12603592 | 1.11E-05 | 5.03E-04 | 5.45E-03 |
|  | rs177567 | 3.11E-03 | 8.27E-03 | 2.79E-02 |
|  | rs216221 | 6.45E-03 | 1.34E-02 | 4.30E-02 |
|  | rs2224770 | 4.22E-07 | 1.84E-04 | 4.05E-03 |
| *SYNGAP1* | rs176249 | 6.02E-03 | 1.36E-02 | 4.21E-02 |
| *TNF* | rs130079 | 2.80E-02 | 3.28E-02 | 5.00E-02 |
|  | rs1800629 | 1.98E-03 | 3.65E-03 | 2.37E-02 |
|  | rs2075800 | 7.79E-03 | 1.24E-02 | 3.13E-02 |
|  | rs6457375 | 2.04E-03 | 4.68E-03 | 2.14E-02 |
|  | rs8084 | 1.83E-02 | 2.61E-02 | 4.60E-02 |
|  | rs9266797 | 8.32E-04 | 3.71E-03 | 1.99E-02 |
|  | rs9268142 | 3.94E-03 | 6.24E-03 | 3.39E-02 |

3、The most significant eQTL analyses results in brain from CMC.

| **Gene** | **SNP** | **P_value** |
| --- | --- | --- |
| *MAPK3* | rs11644809 | <0.01 |
|  | rs34855001 | <0.05 |
|  | rs4424923 | <0.01 |
|  | rs4583255 | <0.01 |
|  | rs4788211 | <0.05 |
| *NOTCH4* | rs2534675 | <0.01 |
|  | rs2855802 | <0.01 |
|  | rs433061 | <0.05 |
| *SRR* | rs12603592 | <0.01 |
|  | rs177567 | <0.01 |
|  | rs216176 | <0.01 |
|  | rs216221 | <0.01 |
|  | rs2224770 | <0.01 |
|  | rs441750 | <0.01 |
|  | rs66529059 | <0.01 |
|  | rs9893573 | <0.01 |

4、The most significant eQTL analyses results in blood from NG.

| **Gene** | **SNP** | **P_value** | **FDR** |
| --- | --- | --- | --- |
| *CLU* | rs9331950 | 1.22E-09 | 6.15E-06 |
| *CSNK2B* | rs1265947 | 2.35E-13 | 0.00E+00 |
|  | rs1800629 | 9.11E-14 | 0.00E+00 |
|  | rs2736425 | 2.48E-13 | 0.00E+00 |
|  | rs3094005 | 1.21E-11 | 0.00E+00 |
|  | rs3094014 | 5.48E-06 | 3.87E-03 |
|  | rs3128986 | 6.68E-10 | 0.00E+00 |
|  | rs3132089 | 7.22E-10 | 0.00E+00 |
|  | rs519417 | 5.52E-12 | 0.00E+00 |
|  | rs622871 | 7.48E-22 | 0.00E+00 |
|  | rs805302 | 6.20E-30 | 0.00E+00 |
|  | rs805303 | 3.23E-31 | 0.00E+00 |
| *GABBR1* | rs1233372 | 1.32E-12 | 0.00E+00 |
|  | rs1610589 | 1.70E-13 | 0.00E+00 |
|  | rs1610719 | 1.18E-09 | 6.16E-06 |
|  | rs1610720 | 4.68E-06 | 3.29E-03 |
|  | rs1610721 | 1.91E-07 | 1.21E-04 |
|  | rs1610725 | 5.27E-05 | 3.05E-02 |
|  | rs1611214 | 8.67E-05 | 4.72E-02 |
|  | rs1611296 | 1.87E-10 | 0.00E+00 |
|  | rs1611350 | 5.14E-14 | 0.00E+00 |
|  | rs1633019 | 1.64E-08 | 2.33E-05 |
|  | rs1633022 | 4.74E-07 | 3.48E-04 |
|  | rs1633086 | 1.88E-10 | 0.00E+00 |
|  | rs1737004 | 3.47E-06 | 2.53E-03 |
|  | rs1737020 | 1.14E-08 | 2.35E-05 |
|  | rs1737048 | 4.32E-07 | 3.00E-04 |
|  | rs2523432 | 3.50E-11 | 0.00E+00 |
|  | rs3094724 | 2.24E-17 | 0.00E+00 |
|  | rs3117289 | 1.54E-22 | 0.00E+00 |
|  | rs3129055 | 7.36E-16 | 0.00E+00 |
|  | rs3129056 | 7.36E-16 | 0.00E+00 |
|  | rs3131864 | 2.11E-13 | 0.00E+00 |
|  | rs885945 | 4.48E-17 | 0.00E+00 |
|  | rs9258289 | 1.89E-07 | 1.21E-04 |
| *MAPK3* | rs4424923 | 3.54E-133 | 0.00E+00 |
|  | rs4788197 | 5.53E-70 | 0.00E+00 |
|  | rs4788198 | 7.61E-69 | 0.00E+00 |
| *NOTCH4* | rs204993 | 1.48E-07 | 8.83E-05 |
|  | rs204994 | 6.84E-05 | 3.85E-02 |
|  | rs2280774 | 8.75E-08 | 7.27E-05 |
|  | rs3096675 | 1.50E-07 | 8.83E-05 |
|  | rs3096680 | 1.79E-07 | 1.04E-04 |
|  | rs3096683 | 6.09E-08 | 5.64E-05 |
|  | rs3096690 | 8.26E-07 | 7.38E-04 |
|  | rs3115561 | 1.50E-07 | 8.83E-05 |
|  | rs3132930 | 1.50E-07 | 8.83E-05 |
|  | rs3132931 | 5.69E-07 | 4.74E-04 |
|  | rs3132945 | 1.50E-07 | 8.83E-05 |
|  | rs3134940 | 2.62E-08 | 3.45E-05 |
|  | rs3135394 | 7.15E-06 | 4.93E-03 |
|  | rs3864301 | 5.69E-07 | 4.74E-04 |
|  | rs389883 | 2.20E-21 | 0.00E+00 |
|  | rs6457513 | 1.50E-07 | 8.83E-05 |
|  | rs6457516 | 7.22E-06 | 4.98E-03 |
|  | rs6908927 | 1.50E-07 | 8.83E-05 |
|  | rs7746994 | 1.50E-07 | 8.83E-05 |
|  | rs7749904 | 1.50E-07 | 8.83E-05 |
|  | rs9268083 | 1.50E-07 | 8.83E-05 |
|  | rs9268084 | 6.09E-08 | 5.64E-05 |
|  | rs9268097 | 6.09E-08 | 5.64E-05 |
|  | rs9268659 | 1.40E-13 | 0.00E+00 |
| *SRR* | rs11078018 | 2.33E-39 | 0.00E+00 |
|  | rs11078019 | 1.29E-39 | 0.00E+00 |
|  | rs1122645 | 8.77E-40 | 0.00E+00 |
|  | rs12603592 | 5.36E-38 | 0.00E+00 |
|  | rs12943566 | 2.65E-40 | 0.00E+00 |
|  | rs12950555 | 7.19E-40 | 0.00E+00 |
|  | rs177567 | 1.31E-39 | 0.00E+00 |
|  | rs216176 | 8.77E-40 | 0.00E+00 |
|  | rs216199 | 2.46E-45 | 0.00E+00 |
|  | rs216221 | 7.19E-40 | 0.00E+00 |
|  | rs2224770 | 2.46E-45 | 0.00E+00 |
|  | rs375245 | 1.60E-39 | 0.00E+00 |
|  | rs394752 | 1.60E-39 | 0.00E+00 |
|  | rs422632 | 8.77E-40 | 0.00E+00 |
|  | rs441750 | 8.77E-40 | 0.00E+00 |
|  | rs4790884 | 4.97E-38 | 0.00E+00 |
|  | rs8076939 | 5.12E-38 | 0.00E+00 |
|  | rs9893573 | 3.18E-37 | 0.00E+00 |
|  | rs9899193 | 7.19E-40 | 0.00E+00 |
|  | rs9905529 | 8.77E-40 | 0.00E+00 |
|  | rs9909895 | 6.71E-39 | 0.00E+00 |
| *TNF* | rs1800629 | 7.39E-06 | 5.07E-03 |
|  | rs2428498 | 8.52E-06 | 5.88E-03 |
|  | rs2442734 | 8.40E-06 | 5.83E-03 |
|  | rs2507963 | 8.52E-06 | 5.88E-03 |
|  | rs2516408 | 3.81E-05 | 2.30E-02 |
|  | rs2523554 | 1.50E-06 | 1.25E-03 |
|  | rs2534675 | 3.18E-05 | 1.94E-02 |
|  | rs2534679 | 3.18E-05 | 1.94E-02 |
|  | rs2855802 | 3.18E-05 | 1.94E-02 |
|  | rs2855817 | 3.18E-05 | 1.94E-02 |
|  | rs2905737 | 2.13E-06 | 1.65E-03 |
|  | rs805302 | 8.77E-05 | 4.76E-02 |
|  | rs805303 | 8.77E-05 | 4.76E-02 |

5、The most significant meQTL analyses results in blood.

| **Gene** | **SNP** | **FDR** | **beta** |
| --- | --- | --- | --- |
| *CACNA1C* | rs1016388 | 1.10E-15 | 0.02 |
|  | rs11062170 | 4.96E-04 | -0.01 |
|  | rs10774033 | 6.45E-04 | -0.01 |
|  | rs11062157 | 7.20E-04 | -0.01 |
|  | rs2238053 | 1.26E-03 | -0.01 |
|  | rs146118900 | 5.48E-03 | -0.01 |
|  | rs61909095 | 5.49E-03 | -0.01 |
| *CLU* | rs9331950 | 7.91E-12 | 0.02 |
|  | rs73229093 | 4.75E-09 | 0.02 |
|  | rs73229090 | 1.13E-08 | 0.02 |
|  | rs111659883 | 5.92E-04 | -0.02 |
| *CSNK2B* | rs805302 | 6.87E-13 | -0.07 |
|  | rs3130618 | 4.12E-03 | 0.04 |
| *GABBR1* | rs7757931 | 7.03E-90 | -0.18 |
|  | rs2076484 | 7.03E-90 | -0.18 |
| *GRIN2A* | rs9926303 | 1.71E-15 | 0.02 |
|  | rs7199375 | 4.68E-15 | -0.07 |
| *NOTCH4* | rs6457513 | 3.04E-14 | -0.03 |
|  | rs3132935 | 1.42E-08 | -0.01 |
|  | rs3130321 | 1.08E-06 | -0.02 |
|  | rs204993 | 4.23E-03 | 0.01 |
|  | rs204994 | 6.32E-03 | -0.02 |
| *SRR* | rs2224770 | 1.33E-59 | 0.14 |
|  | rs441750 | 4.94E-57 | 0.14 |
|  | rs177567 | 4.94E-57 | 0.14 |
|  | rs66529059 | 4.51E-37 | -0.12 |
| *SYNGAP1* | rs9394145 | 1.63E-03 | 0.01 |
