# Supplementary material for: Comprehensive functional annotation of susceptibility SNPs prioritized 10 genes for schizophrenia
Source: Transl Psychiatry. 2019 Jan 31;9:56. doi: 10.1038/s41398-019-0398-5 (PMC6355777; doi:10.1038/s41398-019-0398-5)
Supplement: Supplementary file 12 — supplementary Table S10 [file 41398_2019_398_MOESM12_ESM.doc]

Table S10. Pearson correlation analyses and conditional eQTL analyses for *CSNK2B* and *SYNGAP1*.

1. The P value in the Pearson correlation analyses results.

| **Tissue** | ***CSNK2B-C4A*** | ***CSNK2B-C4B*** | ***CSNK2B-SYNGAP1*** | ***SYNGAP1-C4A*** | ***SYNGAP1-C4B*** |
| --- | --- | --- | --- | --- | --- |
| Brain Amygdala | **0.483** | 0.016 | **0.430** | **0.435** | **0.466** |
| Brain Anterior cingulate cortex BA24 | **0.148** | **0.405** | **0.154** | 0.024 | **0.457** |
| Brain Caudate basal ganglia | **0.512** | **0.106** | **0.392** | **0.391** | **0.796** |
| Brain Cerebellar Hemisphere | **0.243** | **0.403** | 0.029 | **0.063** | **0.103** |
| Brain Cerebellum | **0.327** | 0.025 | **0.479** | **0.511** | **0.454** |
| Brain Cortex | **0.730** | 0.005 | 0.003 | **0.805** | **0.188** |
| Brain Frontal Cortex BA9 | **0.640** | **0.208** | 0.011 | **0.373** | **0.271** |
| Brain Hippocampus | **0.207** | **0.147** | **0.496** | **0.913** | **0.238** |
| Brain Hypothalamus | **0.283** | **0.906** | **0.158** | 0.014 | **0.368** |
| Brain Nucleus accumbens basal ganglia | 0.022 | **0.889** | **0.203** | **0.805** | **0.081** |
| Brain Putamen basal ganglia | **0.720** | **0.432** | **0.161** | **0.255** | **0.374** |
| Brain Spinal cord cervical c-1 | **0.992** | **0.563** | **0.594** | **0.550** | **0.094** |
| Brain Substantia nigra | **0.211** | **0.256** | 0.002 | **0.859** | **0.571** |
| Whole Blood | **0.868** | **0.366** | **0.786** | 0.042 | **0.095** |

Note: Bold means P > 0.05.

2. Conditional eQTL analyses of *CSNK2B*.

| **SNP** | **Tissue** | **Raw** | | **Adjust** | |
| --- | --- | --- | --- | --- | --- |
|  |  | **beta** | **P** | **beta** | **P** |
| rs1265754 | Brain Cerebellum | 0.222 | 4.94E-03 | 0.118 | 1.32E-01 |
| rs1265754 | Brain Nucleus accumbens basal ganglia | 0.264 | 2.38E-02 | 0.089 | 4.35E-01 |
| rs1800629 | Brain Cerebellar Hemisphere | 0.172 | 2.65E-02 | 0.151 | 5.05E-02 |
| rs1800629 | Brain Nucleus accumbens basal ganglia | 0.236 | 3.85E-03 | 0.160 | **4.49E-02** |
| rs204993 | Brain Anterior cingulate cortex BA24 | -0.169 | 1.47E-02 | -0.197 | **3.78E-03** |
| rs2075800 | Brain Caudate basal ganglia | -0.119 | 2.57E-02 | -0.112 | **3.45E-02** |
| rs2075800 | Brain Nucleus accumbens basal ganglia | -0.191 | 1.58E-03 | -0.154 | **8.93E-03** |
| rs2075800 | Brain Putamen basal ganglia | -0.133 | 4.60E-02 | -0.125 | 6.02E-02 |
| rs2075800 | Whole Blood | -0.042 | 1.02E-03 | -0.042 | **8.72E-04** |
| rs2736176 | Brain Amygdala | -0.137 | 3.68E-02 | -0.098 | 1.24E-01 |
| rs2736176 | Brain Caudate basal ganglia | -0.169 | 2.69E-03 | -0.158 | **4.62E-03** |
| rs2736176 | Brain Cortex | -0.169 | 7.81E-03 | -0.139 | **2.39E-02** |
| rs2736176 | Brain Frontal Cortex BA9 | -0.108 | 4.32E-02 | -0.114 | **3.17E-02** |
| rs2736176 | Brain Hippocampus | -0.173 | 1.11E-02 | -0.187 | **4.55E-03** |
| rs2736176 | Brain Nucleus accumbens basal ganglia | -0.196 | 3.18E-03 | -0.166 | **1.00E-02** |
| rs2736176 | Brain Putamen basal ganglia | -0.164 | 2.78E-02 | -0.156 | **3.58E-02** |
| rs2857694 | Brain Caudate basal ganglia | -0.108 | 3.71E-02 | -0.100 | 5.16E-02 |
| rs2857694 | Brain Cortex | -0.141 | 1.51E-02 | -0.123 | **2.83E-02** |
| rs2857694 | Brain Hippocampus | -0.174 | 4.78E-03 | -0.174 | **3.51E-03** |
| rs2857694 | Brain Nucleus accumbens basal ganglia | -0.141 | 1.82E-02 | -0.120 | **3.87E-02** |
| rs2857694 | Whole Blood | -0.054 | 2.09E-05 | -0.055 | **1.43E-05** |
| rs433061 | Brain Cerebellum | 0.222 | 4.94E-03 | 0.118 | 1.32E-01 |
| rs433061 | Brain Nucleus accumbens basal ganglia | 0.264 | 2.38E-02 | 0.089 | 4.35E-01 |
| rs9262696 | Brain Caudate basal ganglia | 0.145 | 2.63E-02 | 0.150 | **2.04E-02** |
| rs9262696 | Brain Hypothalamus | 0.141 | 2.17E-02 | 0.143 | **1.86E-02** |
| rs9262696 | Brain Substantia nigra | -0.249 | 7.91E-03 | -0.244 | **8.11E-03** |
| rs9263473 | Brain Hypothalamus | 0.127 | 1.21E-02 | 0.127 | **1.16E-02** |
| rs9263473 | Brain Putamen basal ganglia | 0.195 | 2.93E-03 | 0.203 | **1.89E-03** |
| rs9263473 | Brain Substantia nigra | -0.162 | 3.26E-02 | -0.147 | 5.05E-02 |
| rs9268142 | Brain Hypothalamus | 0.152 | 1.34E-02 | 0.127 | **3.87E-02** |

Note: Bold means P < 0.05. Adjust means after adjusting the residual effect of *C4A* and *C4B*.

*3. Conditional eQTL analyses of SYNGAP1.*

| **SNP** | **Tissue** | **Raw** | | **Adjust** | |
| --- | --- | --- | --- | --- | --- |
|  |  | **beta** | **P** | **beta** | **P** |
| rs9276915 | Whole Blood | -0.058 | 4.24E-03 | -0.048 | **1.65E-02** |
| rs9394145 | Whole Blood | 0.047 | 1.59E-02 | 0.045 | **2.08E-02** |
| rs176249 | Brain Hypothalamus | 0.094 | 4.78E-02 | 0.076 | 1.03E-01 |

Note: Bold means P < 0.05. Adjust means after adjusting the residual effect of *C4A* and *C4B*.
